# Chemical risk analysis competence in the Nordics is at stake

**DOI:** 10.1007/s11356-025-36318-2

**Published:** 2025-04-02

**Authors:** Åke Bergman, Hrönn Jörundsdóttir, Lisbeth E. Knudsen, Matti Viluksela, Jan Alexander, Åse Krøkje, Kimmo Peltonen, Jaana Rysä, Anette Schnipper, Per Sporrong, Ingrid Ericson Jogsten, Halldór P. Halldórsson, Annika Hanberg, Karin Sørig Hougaard, Gunilla Sandström, Johan Øvrevik, Hubert Dirven

**Affiliations:** 1 School of Science and Technology, Örebro University, 701 82 Örebro, Sweden; 2 Norwegian Institute of Public Health, Skøyen, P.O. Box 222, 0213 Oslo, Norway; 3 Icelandic Food and Veterinary Authority, Austurvegur 64, 800 Selfoss, Iceland; 4 Department of Public Health, University of Copenhagen, 1315 Copenhagen, Denmark; 5 School of Pharmacy, University of Eastern Finland, P.O. Box 1627, 70211 Kuopio, Finland; 6 Environmental Health Unit, The Finnish Institute for Health and Welfare, 70701 Kuopio, Finland; 7 Department of Biology, Norwegian University of Science and Technology, Høgskoleringen 5, 7491 Trondheim, Norway; 8 The Finnish Safety and Chemicals Agency, PL 66 (Opastinsilta 12 B), 00521 Helsinki, Finland; 9 Kornvej 19, 4040 Jyllinge Denmark; 10 University of Iceland’S Research Centre in Sudurnes, Gardvegi 1, 245 Sandgerdi, Iceland; 11 Institute of Environmental Medicine, Karolinska Institutet, PO Box 210, 17177 Stockholm, Sweden; 12 The National Research Center for the Working Environment, Lersø Parkallé 105, 2100 Copenhagen Ø, Denmark; 13 Ahlsell AB, 117 98 Stockholm, Sweden; 14 Ahlsell AB, Adolfsbergsvägen 5, 702 27 Örebro, Sweden; 15 Department of Biosciences (Human Toxicology), University of Oslo, Postboks 1066, Blindern, 0316 Oslo, Norway

**Keywords:** Risk assessment, Risk communication, Chemicals, Competence needs, Training, Education

## Abstract

Toxicology-related experts are on a daily basis working with the safety assessment of chemicals for human health and the environment, providing knowledge applied for management and regulation of chemicals. The field of toxicology is undergoing continuous transition away from traditional safety evaluation studies in experimental animals to application of new approach methodologies (NAMs), the use of omics-related technologies, and concepts like next-generation risk assessment. This requires expertise in the new technologies but does not dismiss the need of knowledge for interpretation of in vivo studies and full understanding of chemical exposure data. A survey was initiated in 2022 in the Nordic countries to assess current and future needs for competences in risk analysis. In total, 40 replies were received from Denmark, Finland, Norway and Sweden, while an expert assessment was performed in Iceland. The responses primarily (87.5%) came from national authorities, research institutes, industry/business, and consultants, less from the hospital system, NGOs, and others. The survey shows obvious difficulties in finding competent and trained personnel in all areas in risk analysis. Since the individual Nordic countries lack critical mass, a Nordic initiative for training and education is recommended to counteract loss of competences within chemical risk analysis in the future.

**Supplementary Information:**

The online version contains supplementary material available at 10.1007/s11356-025-36318-2.

## Introduction

The global One Health situation is threatened with crises due to climate change, biodiversity changes chemical pollution, and waste, known as the Triple Crises (WHO 2023a). Chemicals of anthropogenic origin constitute an important element in all of these crises, directly or indirectly, by refining natural resources (e.g., crude oil and mining), in consumer products and production of waste with a profound impact on all the 17 United Nations Sustainable Development Goals (UN 2015). Accordingly, risk assessment and management of chemicals in use, development, and their environmental occurrence need advanced competencies for decision making and communication.

Expertise is needed to prevent regrettable introduction of persistent, bioaccumulative, and toxic chemicals, as well as mobile chemicals with shorter half-lives, to the environment. Important to avoid are also substitutions for regulated substances that are as harmful as the substituted chemicals. Further, the ongoing industrial green transition, e.g., the energy sector, will require new competent personnel in chemical risk analyses. The incidences of non-communicable diseases (NCDs) like cardiovascular diseases, cancers, chronic respiratory diseases, obesity, diabetes, and mental health continue to increase. The global share of NCD deaths among all deaths increased from 61% in 2000 to 74% in 2019 (WHO 2023b). The WHO has recognised that changing lifestyle and environmental factors, including exposure to chemicals, play an important role in prevention of NCDs.

In order to improve and accelerate the safety assessment of chemicals, the field of toxicology is advancing, leveraging new technologies, e.g., omics-related data in the hazard assessment, use of non-animal data by introducing concepts as new approach methodologies (NAMs), integrated approaches to testing and assessment (IATA), and next-generation risk assessments (NGRA). Furthermore, the use of computer-based learning and artificial intelligence to assess the safety of chemicals will advance the science beyond traditional hazard identification, hazard characterisations, exposure assessment, and risk characterisation. The scientific development will promote both societal and individual preparedness managing unforeseen accidents or long-term environmental and health effects of new and existing chemicals.

The increased attention to sustainability and green agendas (EU 2022; EC 2024) also necessitates extended competencies in risk assessment. Currently, the European Union (EU) research funding is addressing hazards related to chemical risks in ambitious programs such as the Partnership for the Assessment of Risks from Chemicals (PARC 2024), the European Human Exposome Network (EHEN 2024), endocrine related projects (EURION 2024), new approach methodology projects (ASPIS 2024), and microplastic and nanoplastic projects (CUSP 2024). Researchers from all the five Nordic countries are participating in several of these existing projects/clusters.

It is important to point out that although regulation of chemicals in Europe occurs at the level of the European Union by the EU commission and its main agencies, it relies heavily on national expertise. This is also true for the intergovernmental Organisation for Economic Co-operation and Development (OECD), the International Agency for Research on Cancer (IARC), and the International Programme on Chemical Safety (IPCS). Risk assessments of chemical exposure at a national level are mainly performed by national agencies and experts. Hence, it is important to staff national agencies and knowledge supplying organisations with competent employees that are up to date with current research trends, as well as familiar with new methodologies.

Further, it is a matter of providing highly competent professionals with up-to-date knowledge on modern and advanced theories and techniques for application in risk analysis and safety assessment of chemicals for the decades to come. This concern also involves university education programs, limited funding for doctoral students, and post-doctoral positions, as well as limited number of well-trained teachers and sometimes students attending the education programs and courses.

The objective of the present survey was to map the current competence status and needs in the broad area of risk analysis in the Nordic countries. Risk analysis comprises risk assessment, risk management, and risk communication, and it requires multidisciplinary competences in a large number of chemical, environment, and health discipline areas (Supplementary Information (SI), Part A) for assessments and recommendations to and decisions by the stakeholders in politics, authorities, and private sector including NGOs. The survey specifically focussed on identifying the needs of non-academic stakeholders, and it is recommended to follow up with an additional survey to evaluate the academic educational framework with respect to toxicology-related disciplines in the Nordics.

## Methodology

The present study was initiated and designed by the “Competence provision needs for sustainable chemical risk analysis and safety in the Nordics” (CRIANN) steering group. It includes all the authors of this contribution (further details presented in the SI). The steering group represents different stakeholders in the Nordics (academia, authorities, research institutes, businesses), all with long experience from the field of risk assessment, management, and communication. The group also represent different fields of expertise. The steering group met frequently to discuss the outcome of the survey and agreed on the “Reflections” presented in this paper.

The potential respondents were approached with a letter (SI, PART A). The survey was conducted separately, by applying the same questionnaire, in each of the five Nordic countries. Since the organisation of agencies and organisation involved in chemical risk assessment is different between the countries, the members of the working group made for each country a list of agencies and organisations that were approached. The list was also shared between the five participating countries. A questionnaire was prepared (a copy is included in the SI, PART B) by a small core team and tested by all the members of the working group. The responses received were discussed, and an updated survey was prepared and directed to stakeholders outside the universities.

The total number of stakeholders approached in Denmark (DK), Finland (FI), Iceland (IS), Norway (NO), and Sweden (SE) were 19, 29, 7, 37, and 48, respectively. Confidentiality in processing the responses was assured through the ORU survey system Artologik Survey and Report. The responses obtained from DK, FI, NO, and SE (individually presented in SI, PARTs C, D, E, and F, respectively) were analysed to generate a system report for each country (SI). The free text interpretations were made by the working group and presented to the steering group for their comments.

The responses from Iceland (IS) were gathered through direct contacts with the main stakeholders identified, including the University of Iceland and its research institutes involved in environmental research, the Environment Agency, and the Food and Veterinary agency. As the research environment is very small in Iceland and the number of experts in environmental risk–related science is very limited, hence the communication routes are short and assisting in connecting with the main stakeholders. Therefore, it is estimated that even if the number of stakeholder interviews are few, the number of stakeholders is considered adequate for a representative overview of the field in Iceland. The working group prepared a merged report for the four Nordic countries, DK, FI, NO, and SE, while the Icelandic partner summarised the perceived competence needs based on personal network contacts. A summary of the results is presented in the SI (PART G).

This paper with the main findings will be distributed to relevant stakeholders (governmental groups, universities) in each of the five countries separately, with an invitation for a discussion on follow-up activities that can be initiated. The report will also be discussed in the national hubs from the EU program PARC, where research groups and stakeholders meet.

## Results and discussion

In total, 40 replies were received from DK, FI, NO, and SE, while no questionnaire responses were collected from IS. The responses primarily came from national authorities, research institutions (outside universities), industry/business, and consultants (87.5%), with fewer received from the hospital system, NGOs, and others. Across countries (excluding Iceland), the average response rate was 28%. However, the rate varied between the countries (FI: 45%, DK: 37%, SE: 24%, NO: 22%, while expert assessment was performed in IS). The actual numbers of questionnaire responses were dominated by national authorities and research institutes with 11 and 10 answers, respectively; industry/business with nine, consultants with five, while an additional five responses were distributed among other stakeholders (cf. SI). The responses were posted by one organisation with 99–109 employees (DK), two with 55–65 (FI), one with 33–43, and one with 55–65 (NO) personnel within risk analysis. The most abundant numbers of employees in the responding stakeholder organisations were 1–10 persons.

Country-specific generated analyses of the results are presented in the SI (PART C-F).

### Areas of competence

The answers received to question Q3 “What specialization, in chemical risk assessment /communication, do you have in your organization? Please estimate the number of personnel for each specialization.” included alternative (non-animal) in vitro methods, animal testing, bioinformatics, chemical analysis, chemistry/environmental chemistry, ecotoxicology, epidemiology, exposure assessment, QSAR and read-across, risk assessment, risk communication, risk management, statistics, systematic literature reviews, toxicology, and some others were mentioned. Based on the feedback received, it is clear that it is challenging to answer the question related to specialisation as personnel often possess expertise in multiple areas within risk analysis. Furthermore, it was mentioned that toxicity studies are outsourced by some of the respondents, to Contract Research Organisations (CROs). To increase understanding of the spectrum of competences required, we conclude that it would be beneficial to interview some actors in the field of risk analysis of chemicals. We propose this as a next step to improve understanding of the competence needs at different levels.

The survey shows that the responding organisations are built on academics, with DK, FI, and SE having a high proportion of personnel with doctoral and/or master’s degrees. These three countries also have significant number of personnel with bachelor’s degrees. NO shows lower numbers of personnel with any of the three academic degrees reported. In the latter case, this might reflect a national tradition of not commonly using academic degrees and titles in their daily interactions.

The European Register of Toxicologists (ERT) is a service of EUROTOX established in 1994. Individuals who want to be registered and are found to comply with the requirements defined by EUROTOX and National Societies of Toxicology. The Nordic countries all reported their numbers of European Registered Toxicologist (ERTs), i.e., 70 persons in NO, FI with 59, SE 45, and DK 39. None is registered in IS. How many of the reported ERTs that are active in risk assessments of environment and health hazards of anthropogenic chemicals is unclear. Because of General Data Protection Regulation (GDPR) in the European Union, it is not possible to identify in which sector these ERTs actually work or to get more background information.

### Personnel engaged in international organisations

**Fig. 1:**
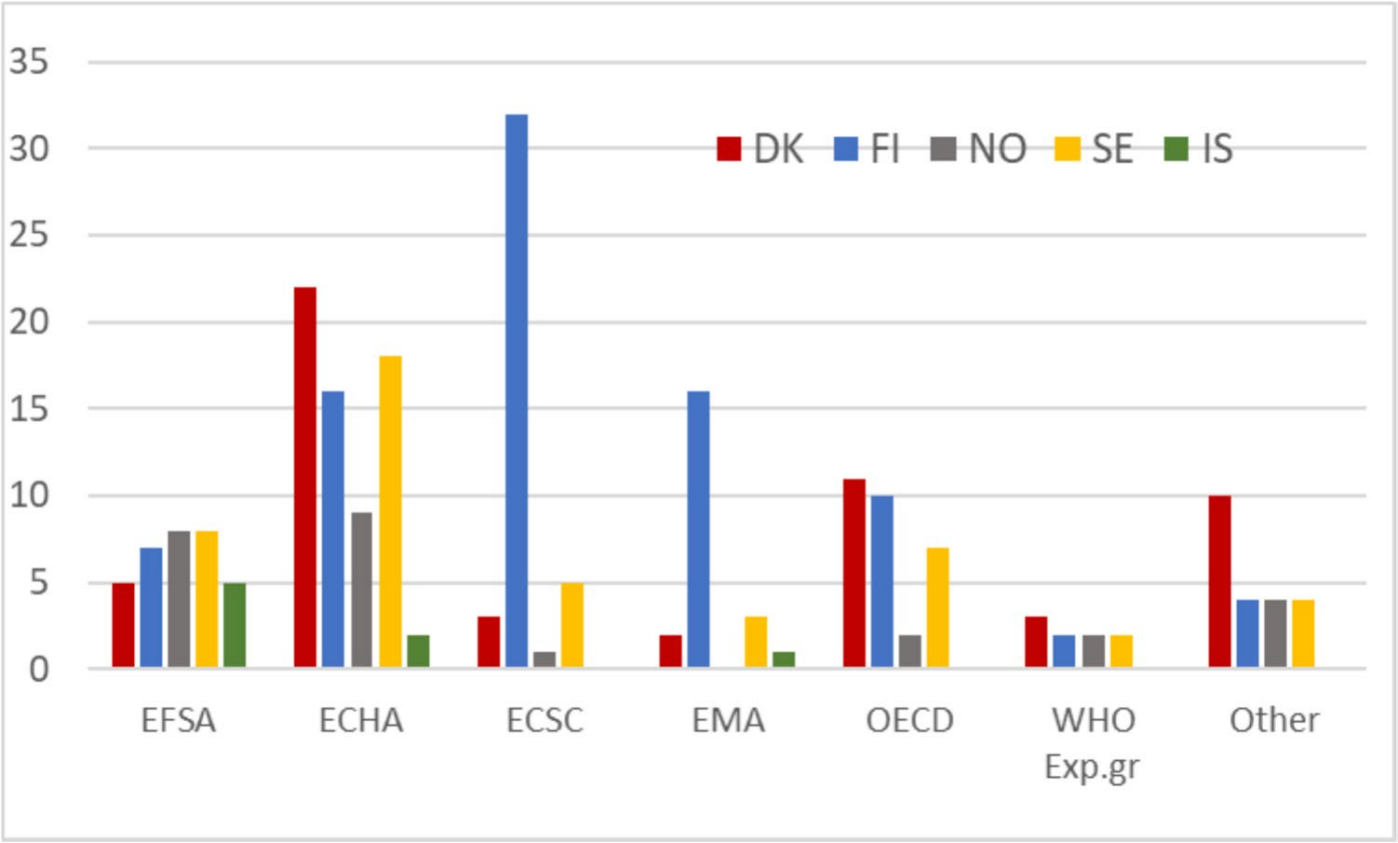
Number of personnel (*y*-axis) engaged in EU and/or Intergovernmental risk analysis organisations from the five Nordic countries. The organisations are EFSA (European Food Safety Authority); ECHA (European Chemicals Agency); ECSC (European Commission Scientific Committees); EMA (European Medicines Agency), OECD, and WHO expert groups

Figure 1 presents the number of personnel involved in European organisation like the European Food Safety Authority (EFSA), European Chemicals Agency (ECHA), the European Commission Scientific Committees (ECSC) or European Medicine Agencies (EMA), or in international organisations like OECD and WHO, as reported by the organisations that responded to the survey at the time that the survey was conducted. The group of “other” organisations comprises International Agency for Research on Cancer (IARC, a WHO organisation), The Strategic Approach to International Chemicals Management-SAICM working groups, UNEP-related working groups on sustainable chemicals, Eurometaux, CEFIC and ILSI (Working group on food allergens), and International Council for Harmonisation of Technical Requirements for Pharmaceuticals for Human Use (ICH). These assignments are not fulltime activities but require high level of competence. For some organisations, toxicologists working for national authorities are more likely to participate, but for other agencies (for example EFSA and WHO), this is less common. Moreover, participation in these assignments often involves direct competition with specialists from other countries. An underreporting on this topic is expected since individuals are most likely not representing their organisations but are invited to participate based on their individual expertise and track record. Sometimes the experts do this work in their free time. It is possible that a person is member of more than one organisation but not very likely since the workload in these expert groups is quite demanding.

### Age profile of personnel

**Fig. 2:**
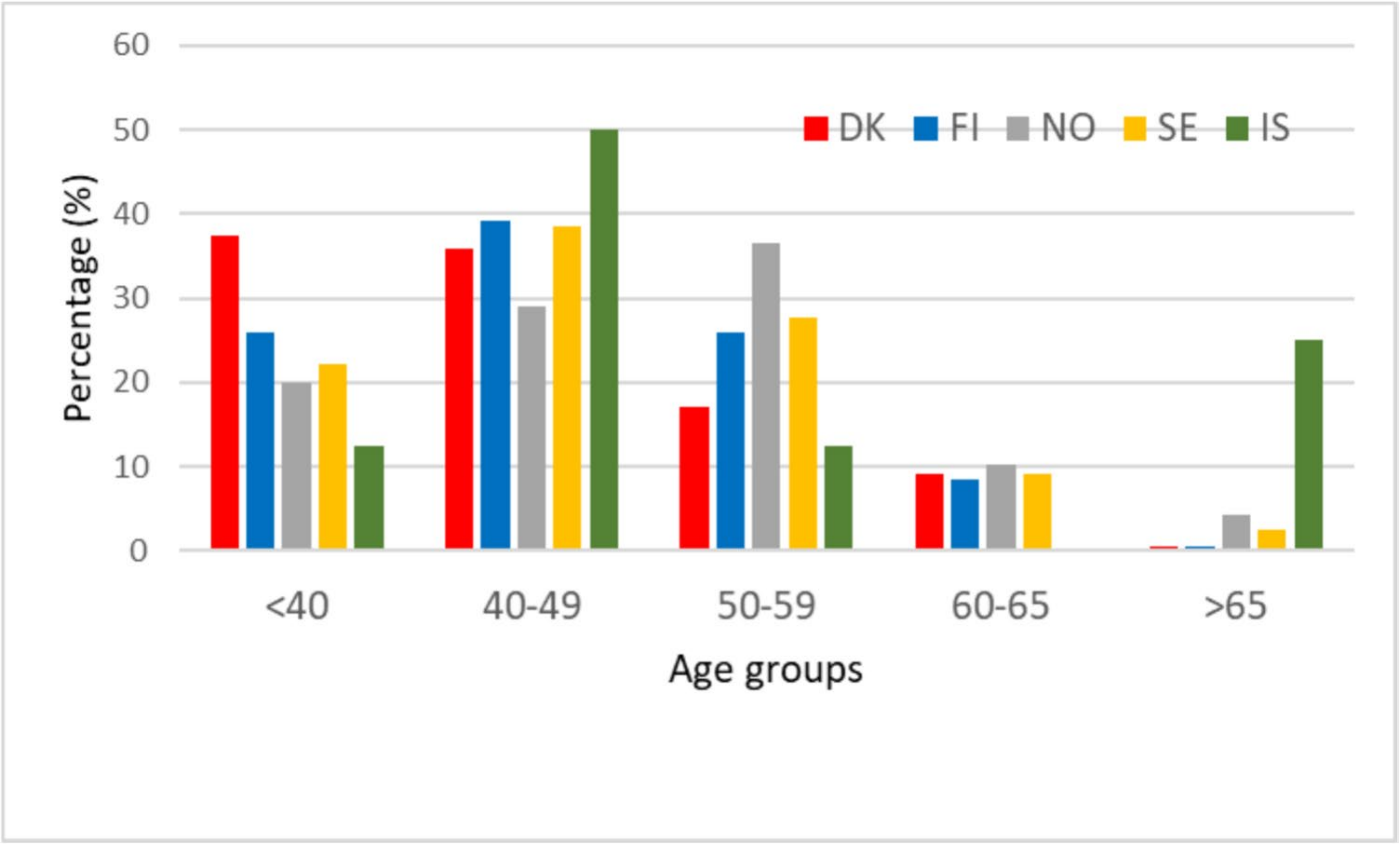
Relative personnel age profile (%) for DK, FI, NO, SE, and IS

The age profile of the personnel (Q6) is visualised in Fig. 2. The graph is constructed on the country-wise responses with the estimate of the mean values when ranges were reported from the individual countries (cf. SI, PART C-F) and expert assessments from IS. DK shows to have younger personnel than the other countries that all indicate a hump for the ages 40–59. The age profile at 60–65 years of age is similar between the countries except for IS with almost none in this age group. IS has 25% of their personnel in the age group > 65, which is far higher than NO and SE and in particular much higher than DK and FI.

### Need for future recruitment and training

In response to the question (Q7) “Is there a need for hiring of replacement/expanding the number of chemical risk assessment/communication personnel over the next 5–10 years,” needs were identified but not articulated as dramatic needs, rather as a matter of replacement for retirees. The needs related to changes in demands of the different organisations are based on their societal mandate. For an overview, please see responses in the SI (Part G, Q7).

When looking into the needs expressed for recruitments of relevant personnel for the coming 4–7 years (Q8), it is clear that there are extensive demands for personnel in both novel and contemporary fields of expertise. Needs for the future are mentioned in the areas, e.g., probabilistic risk assessment based on artificial intelligence, computational risk assessment, NAMs, exposure modelling, epidemiologists with register study skills, competence related to biocides, occupational health and toxicology, genotoxicology, endocrine toxicology, immune toxicology, and neurotoxicology. Further, needs for more/better knowledge in food toxicology, particularly in the area of natural toxins, radioactive contamination, and food allergy, are expressed. More competence in regulatory (eco)toxicology is repeatedly mentioned as a need. Further, it is also mentioned there is a need for training students with generic and basic knowledge in risk analysis related areas, a need competing with expert skills and knowledge. In conclusion, the responses from the Nordics, including views from IS, call for well-educated students at master and doctoral levels from the universities to act as generalists as well as experts.

**Fig. 3:**
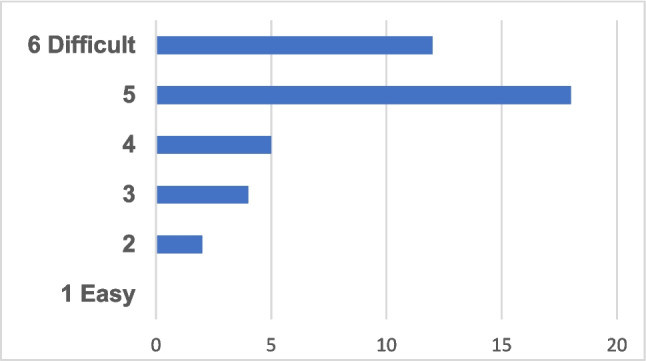
The actual numbers of responses to the question on how difficult it is to recruit personnel in risk analysis is presented in the graph. The majority of responses given are expressing the highest levels (5 and 6 on the scale) of difficulties to recruit personnel in risk assessment/communication in the Nordic countries

The survey also shows apparent difficulties for the organisations in all Nordic countries to recruit personnel in risk assessment/communication (Fig. 3).

It is noted that it is difficult for public administration to compete with the private sector for the most qualified personnel because of lower salary levels; the availability of permanent positions and career pathways are more limited. Concerns are expressed that there is a lack of education in risk assessment but simultaneously expressed that there are courses/programs in the traditional academic disciplines such as chemistry, biology, biochemistry, pharmacy, and toxicology. We see that in a number of European countries, including in the Nordic countries, departments of toxicology at universities are closing down. Based on the comments from the respondents, there is not enough qualified candidates available even though it varies between the countries. Still, the comments do vary. The responses indicate that academia does not deliver sufficient numbers of appropriately trained graduates (c.f. SI PART G, Q10). We conclude that the survey shows a competence provision need in education and training of risk assessment/communication students.

As many as 87% of the responding organisations offer internal training to their recruited personnel. The training includes all from basic courses, external courses, webinars to job training (cf. SI PART G, Q11).

In response to Q12 “Which areas of expertise are primarily lacking when you want to hire new personnel?,” it is evident that this relates to all the areas presented in the survey: alternative (non-animal) in vitro methods, animal testing, bioinformatics, chemical analysis, chemistry/environmental chemistry, ecotoxicology, epidemiology, exposure assessment, QSAR and read-across, risk assessment, risk communication, risk management, statistics, toxicology, and some others were mentioned, such as probabilistic risk assessment, artificial intelligence (AI), NAMs, and exposure modelling. Systematic literature reviews were not mentioned specifically in the responses. This may be explained by personnel with competence in systematic literature reviews benefit from expertise outside the toxicology/risk assessment field. The responses, and hence the expertise lacking, varied among the countries (cf. SI PART G, Q12).

**Fig. 4:**
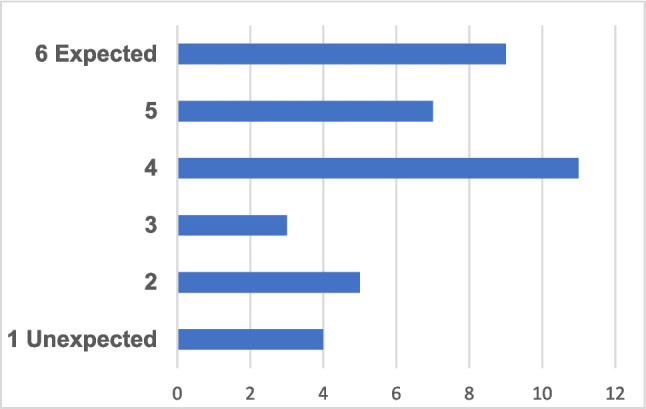
Number of responders and the expressed degree of expectation for future lack of expertise in the area of risk assessment/communication in the Nordic countries

The survey asked if the respondents expect a lack of expertise in the area of risk assessment/communication in the near future. This question was met with somewhat different views from the five countries. The interpretation of the results shown in Fig. 4 is an expected lack of expertise based on responses from 39 organisations. Also, IS report that there is today a severe lack of expertise in risk assessment/communication, and this situation is expected to continue.

## Managing the situation

The respondents commented on how they foresee to meet the needs of highly competent/qualified personnel in chemical risk assessment/communication in their organisation for the coming 5 years. The organisations call for external online or actual courses and conferences. Furthermore, they see participation in, e.g., EU-funded research projects as a possibility for competence improvements. Still, the major comments suggest internal courses and training to keep up with needs. The latter is difficult when competences are needed within areas, where the organisations lack previous expertise.

### Lack of educational programmes—suggested solutions

Responses and comments on how, on a national level, to optimise the numbers of competent professionals in risk analysis relate to proper toxicology education programs covering the main areas of risk assessment and communication (Q12, above or SI). The lack of academic programs in human toxicology is a main drawback for several countries and/or geographical regions within one country. The needs might therefore be met with good and competent teachers, marketing, innovative learning methods at the universities, and through workplace training. There is a proposition to establish a toxicological centre of expertise in Finland (Pyötsiä 2018), with a particular focus on developing toxicological expertise, especially in risk assessment. To approach this from a One Health perspective, broader recruitments are needed, especially in the field of medicine and chemistry. Moreover, enhancing collaboration between universities, research institutions, and industry is proposed. Striking a good balance between academia/research, risk assessment, regulators/administration, and industry in the educational program would be beneficial.

Ensuring sufficient funding is of critical importance and must be made available for education and training purposes. It is crucial to maintain a high international standard for the basic training of toxicologists and to educate a larger number of professionals in the long run. In the short term, forming a network of individuals working in this field is suggested to facilitate the sharing of competencies and job opportunities.

### Nordic collaboration

The respondents contributed with numerous suggestions for university courses (see SI PART G, Q13). These responses link to the question on how the Nordic countries could act jointly to optimise the numbers of competent persons for the stakeholders. Cooperation in the Nordics is recommended, e.g., in MSc and PhD in toxicology programs, which may be coordinated between key institutes, likewise on relevant (online) courses, workshops, webinars/seminars in risk analysis, Nordic postgraduate programs and student exchanges. It is considered important that candidates in this field get an option to develop a career with sufficient postdoctoral and/or other positions for young researchers.

A more active joint toxicology organisation is proposed, creating a strong network of the expertise in the Nordic countries, e.g., a Nordic Working Group for Chemicals, Environment and Health. A roster of experts and expertise could be established, similar to, e.g., Joint FAO/WHO Expert Committee on Food Additive (JECFA), Joint FAO/WHO Meeting on Pestcide Residues (JMPR) , or EFSA, referring to the Nordic Expert Group for Criteria Documentation of Health Risks from Chemicals as an example of well-functioning Nordic cooperation. More details in SI PART G, Q17. The comments focus on toxicology but is interpreted by the authors as suggestions for toxicology-related sciences and risk analysis as a whole.

As many as 83% of the respondents are positive to establish a closer, formalised cooperation between the Nordic countries in the area of chemical risk analysis, including training. Still a few responses questioned if the Nordics is big enough and instead favoured initiatives at the EU level. In principle, many of the research requirements are similar in the Nordic countries, but it is still a challenge due to organisational heterogeneity between the Nordic countries, i.e., role of universities and research institutes in education. More unformalised Nordic cooperation is needed in relation to risk assessment issues or ad hoc meetings when needed.

In conclusion, we note support for establishing a forum or an organisation for training and education that is a joint venture between the Nordic countries with appropriate funding.

Some additional comments picked up from the respondents relate to the needs for help requested by companies and authorities as risk assessment issues are becoming increasingly more complex, often based on EU driven actions in, e.g., the regulation of endocrine disruptors and carcinogenic compounds. This stresses the needs for highly qualified people with high risk analysis competences.

## Reflections

The present survey was directed to stakeholders in the area of risk assessment and communication (i.e., risk analysis) to enable identification of their present and future needs for competent personnel for different discipline areas included in risk analysis. The responses covered very well the professional areas of risk analysis. Our perspective presented here is based on individual and/or group observations and experience of professionals in the Nordic countries, including authorities, companies, and research institutions. The reflections from the authors are based on the survey results done by the expert group behind the survey, representing many different disciplines.

We observed a contradiction in the responses between, on the one hand, answers that did not express dramatic needs for new competent personnel, and on the other side, responses expressing significant difficulties in hiring qualified personnel in the risk assessment/communication areas. The responses are over all expressing difficulties in finding and hiring competent staff members independently of the area of competence needs.

We envision substantial needs for competent specialists in chemical risk analysis to replace the retiring generation of experts, who presently serve as teachers in academia, experts at governmental and regional authorities, industry and business professionals, and as NGO members. It is evident that academia is currently unable to provide the society with sufficient numbers of trained “toxicologists,” i.e., experienced people in risk assessment and communication, to meet future expert demands in the Nordic countries. Chemical risk analysis competence in the Nordics is at stake.

A joint letter from Finnish authorities on this topic resulted in a Finnish governmental inquiry (Pyötsiä 2018). Lack of relevant expertise has also expressed at meetings between risk analysis experts in the Nordics. Further, education programmes are seen in some European countries, e.g., Germany and the Netherlands (GT 2025; DNP 2024.

The areas of toxicology and related science disciplines are undergoing a continuous transition with increased use of, e.g., exposome methodologies, use of mechanistic data organised in adverse outcome pathways (AOPs), NAMs, and computer-based laboratory/artificial intelligence. It seems that most respondent of the survey do not have a strategy on how to address these changes in their organisations and what additional competencies are needed in order to optimally explore and support these new methods. At the same time, the respondents presented pronounced needs of novel areas of knowledge to pursue and develop risk analysis. The expected future lack of expertise in risk analysis was also shown in the answers. This is forming an important base for further discussion with universities on how to meet these demands.

The survey clearly shows a request for improved up-to-date academic education in the individual countries but increased cooperation within the Nordics is also suggested. A huge number of suggestions of courses, activities, and study programs were presented, as means to improve the current situation of lack of competences in risk analysis. To increase understanding of the spectrum of competences required, we recommend interviewing some actors in the field of risk analysis of chemicals and a continuous dialogue between universities and users. Managing the competence provision needs exposed in this survey will require deep interviews of representatives of relevant universities to address the issues of academic educations and future plans in risk analysis disciplines.

The vast majority (83%) of the responses promoted the establishment of a joint Nordic organisation/institute in risk analysis. The experience from, e.g., the Dutch post graduate education in toxicology risk assessment training network (DNP 2024) as well as from Germany (GT 2025) is initiatives that can be further explored in the Nordics, preferably as a Nordic coordinated initiative. However, the input from the stakeholders must be taken into account as part of this process.

The EU research agenda for the environment, climate, and health 2021–2030 is in fact requesting research as phrased under RG6.6. “Transformational change in education, training and research” (HERA 2022). Competence provision needs are requested, as expressed in the partnership program PARC (2024), in EHEN (2024), and in the exposome infrastructure program Eirene (2024), since highly educated and trained individuals are prerequisites for present and future advanced research and management in risk analysis of chemicals to protect human health and the environment.

Finally, the competence provision needs shown for the Nordic countries is regarded relevant also for the EU and beyond. The needs are likely potentiated by the present green transformation of industrial technologies and production of infrastructure, materials, goods, and products.

## Supplementary Information

Below is the link to the electronic supplementary material.

Supplementary file1 (PDF 5160 KB)

## Data Availability

All data collected in the present study are presented in the Supplemental Information, which is included in the submission.

## Declarations

### Clinical trial number

Not applicable: The present study is not a clinical study.

### Additional information

Potential publication of the contribution to ESPR should be done Open Access.

## References

[CR1] ASPIS (2024) EC cluster of projects advancing the safety assessment of chemicals without the use of animal testing. https://aspis-cluster.eu/cluster/. Accessed 28 Jan 2025

[CR2] CUSP (2024) cluster of projects collaborating in research on the complex relationship between micro- and nanoplastics and human health, from early life to adulthood. https://cusp-research.eu/about/. Accessed 28 Jan 2025

[CR3] DNP (2024) Postgraduate education in toxicology in the Netherlands. https://toxcourses.nl/. Accessed 28 Jan 2025

[CR4] EC (2024) Environmental strategies and actions plans. https://environment.ec.europa.eu/strategy_en. Accessed 28 Jan 2025

[CR5] EHEN (2024) The European Human Exposome Network (EHEN). https://www.humanexposome.eu/. Accessed 28 Jan 2025

[CR6] Eirene RI (2024) Environmental exposure assessment research infrastructure. https://eirene.eu/. Accessed 28 Jan 2025

[CR7] EU (2022) Strategic research and innovation plan for safe and sustainable chemicals and materials, Publications Office of the European Union. https://op.europa.eu/en/publication-detail/-/publication/9f04603f-534b-11ed-92ed-01aa75ed71a1/language-en2023. Accessed 28 Jan 2025

[CR8] EURION (2024) European cluster to improve identification of endocrine disruptors. New testing and screening methods to identify endocrine disrupting chemicals. https://eurion-cluster.eu/. Accessed 28 Jan 2025

[CR9] GT (2025) Das Weiterbildungsprogramm der Gesellschaft für Toxikologie (GT) https://toxikologie.de/weiterbildung/weiterbildungsprogramm-fachtoxikologe-gt/. Accessed 28 Jan 2025

[CR10] HERA (2022) EU research agenda for the environment, climate & health 2021–20230 (final report). https://spheraresearch.org/hera-eu-research-agenda/. Accessed 28 Jan 2025

[CR11] PARC (2024), European Partnership for the Assessment of Risks from Chemicals (PARC). |https://www.anses.fr/en/content/european-partnership-assessment-risks-chemicals-parc. Accessed 28 Jan 2025 10.3389/ftox.2024.1461967 PMC11339028 39176170

[CR12] Pyötsiä J (2018) Securing competence in toxicology. Reports and Memorandums of the Ministry of Social Affairs and Health. STM/4411/2017 (in Finnish with Swedish and English abstracts). 65 p. ISBN PDF: 978–952–00–3935–6. https://julkaisut.valtioneuvosto.fi/bitstream/handle/10024/160912/STM_22_Toksikologisen%20osaamisen%20turvaaminen_WEB.pdf. Accessed 28 Jan 2024

[CR13] UN (2015) The 17 Goals. https://sdgs.un.org/goals. Accessed 3 Jul 2024

[CR14] WHO (2023a) Declaration of the Seventh Ministerial Conference on Environment and Health, Budapest, Hungary 5–7 2023. https://www.who.int/europe/publications/i/item/EURO-Budapest2023-6 Accessed 3 Jul 2024

[CR15] WHO (2023b) Advancing the global agenda on prevention and control of noncommunicable diseases 2000 to 2020 https://www.who.int/publications/i/item/9789240072695. Accessed 3 Jul 2024

