# Supplementary material for: Chemical risk analysis competence in the Nordics is at stake
Source: Environ Sci Pollut Res Int. 2025 Apr 2;32(17):11299–307. doi: 10.1007/s11356-025-36318-2 (PMC12014805; doi:10.1007/s11356-025-36318-2)
Supplement: Supplementary file 1 — Supplementary file1 (PDF 5160 KB) [file 11356_2025_36318_MOESM1_ESM.pdf]

Chemical Risk Analysis Competence in the Nordics is at stake

Åke Bergman[1*], Hrönn Jörundsdóttir[3], Lisbeth E. Knudsen[4], Matti Viluksela[5,6], Jan Alexander[2], Åse Krøkje[7], Kimmo Peltonen[8], Jaana Rysä[5], Anette Schnipper[9], Per Sporrong[1], Ingrid Ericson Jogsten[1], Halldór P Halldórsson[10], Annika Hanberg[11], Karin Sørig Hougaard[4,12], Gunilla Sandström[13,14], Johan Øvrevik[2,15] and Hubert Dirven[2]

[1] Örebro University, School of Science and Technology, SE-701 82 Örebro, Sweden

[2] Norwegian Institute of Public Health, P.O. Box 222 Skøyen, N-0213 Oslo, Norway

[3] Icelandic Food and Veterinary Authority, Austurvegur 64, 800 Selfoss, Iceland

[4] University of Copenhagen, Department of Public Health, DK-1315 Copenhagen, Denmark

[5] University of Eastern Finland, School of Pharmacy, P.O. Box 1627, FI-70211 Kuopio, Finland

[6] The Finnish Institute for Health and Welfare, Environmental Health Unit, FI-70701, Kuopio, Finland

[7] Norwegian University of Science and Technology, Department of Biology, Høgskoleringen 5, NO-7491 Trondheim, Norway

[8] The Finnish Safety and Chemicals Agency, PL 66 (Opastinsilta 12 B), 00521 Helsinki, FINLAND

[9] Kornvej 19, DK-4040 Jyllinge, Denmark

[10] University of Iceland´s Research Centre in Sudurnes, Gardvegi 1, 245 Sandgerdi, Iceland

[11] Karolinska Institutet, Institute of Environmental Medicine, PO Box 210, SE-17177 Stockholm, Sweden

[12] The National Research Center for the Working Environment, Lersø Parkallé 105, DK 2100 Copenhagen Ø, Denmark

[13] Ahlsell AB, 117 98 Stockholm

[14] Ahlsell AB, Adolfsbergsvägen 5, 702 27 Örebro

[15] University of Oslo, Department of Biosciences (human toxicology), Postboks 1066, Blindern, 0316 Oslo, Norway

Content

SI Part A: Invitation Letter

SI Part B: Preview of the CRIANN Mapping Questionnaire

SI Part C: System generated analyses of the result from the Danish respondents

SI Part D: System generated analyses of the result from the Finnish respondents

SI Part E: System generated analyses of the result from the Norwegian respondents

SI Part F: System generated analyses of the result from the Swedish respondents

SI Part G: Merged analyses of the result of respondents from Denmark, Finland, Norway and Sweden

SI Part A Invitation Letter

Email to all invited to respond to the survey.

«Dear Recipient:

As active in the area of Chemical Risk Analysis (Risk assessment and Risk communication) of chemicals, we are concerned that our five Nordic countries within some years will not be able to meet societal demands for highly educated and well-trained professionals in the chemical risk analysis area to contribute with high quality knowledge in the governmental, private and nongovernmental sectors. Please read more about the background for this survey in the attached document "Background to the Nordic Competence Provision Survey 2022".

Accordingly, we kindly ask you to please consider to participate in this mapping effort. Your input will be instrumental for the outcome of the effort we are undertaking, the more info we can get the better. The mapping is anonymous. You will be able to save the answers you give and get back later to the questionnaire. You then have to click on the link shown below once more to get back to you saved questionnaire. *In order to help in your preparation to respond please see the attached pdf file "Preview CRIANN Mapping Questionnaire"*. Please note that it is not possible to respond within that pdf.

The goals of our initiative are to map the current situation in the five Nordic countries; to identify the needs for competence provision and to propose measures for how the Nordic countries should act to meet these needs. To meet the goals, we kindly ask you to fill in the electronic questionnaire "Risk analysis competence provision questionnaire for Sweden" (one survey per each Nordic country is sent out).

You are identified as stakeholders from the Nordics representing/active in government and regional authorities, industry/business and non-governmental organizations. Based on results of the mapping, a compilation is made and a final report including policy recommendations is prepared and communicated to decision makers.

Please note that ***Risk analysis*** comprise Risk assessment and Risk communication which includes many areas of expertise, such as: Alternative (non-animal) in vitro methods, Animal testing, Chemical analysis, Chemistry/ Environmental chemistry, Ecotoxicology, Epidemiology, Exposure assessment, Food safety, QSAR and read-across, Risk assessment, Risk communication, Risk management, Statistics, Toxicology and many more.

It is not possible to forward the questionnaire but if you please contact me, I will then be able to send the questionnaire to additional persons.

The questionnaire may take approx. 20 min. to respond to but please take advantage of the Preview pdf file attached to this email to prepare your answers.

***Please respond by Friday March 4,* 2022**

Please do not hesitate to let me know that you want to be contacted for further input/discussion related to our mapping effort. That has to be done by you contacting me via email since the mapping is entirely anonymous.

Thank you for participating

On behalf of the Project Group»

SI Part B

Preview of the CRIANN Mapping Questionnaire

Preview - CRIANN Mapping Questionnaire

1. Please indicate your area of affiliation? (mandatory)

- ◯ Research institute
- ◯ National authority
- ◯ Regional authority
- ◯ Hospital practice
- ◯ Industry/Business
- ◯ NGO
- ◯ Consultant
- ◯ Other

2. What is the number of personnel in chemical risk assessment/risk communication in your organization?

Preview

[illegible]. What specialization, in chemical risk assessment/communication, do you have in your organization? Please estimate the number of personnel for each specialization.

Alternative (non-animal) in vitro methods

Animal testing

Bioinformatics

Chemical analysis

Chemistry/Environmental chemistry

Ecotoxicology

Epidemiology

Exposure assessment

QSAR and read-across

Risk assessment

Risk communication

Risk management

Statistics

Systematic literature reviews

Toxicology

Other (please comment below)

Comment

Preview

[illegible]any of the personnel in your organization, with the chemical risk assessment/communication tasks you presented in the previous question, holds a:

Bachelor degree:

Master degree:

Doctoral degree (PhDs, MDs, Dtech (TkT, D.I.T), etc):

5. How many of your personnel are ERTs (European Registered Toxicologist)?

6. What is the present age profile of the risk analysis personnel in your organization?

< 40 years of age:

40-50 years of age:

50-60 years of age:

60-65 years of age:

> 65 years of age:

7. Is there a need for hiring of replacement/expanding the number of chemical risk assessment/communication personnel over the next 5-10 years in your organization, e.g. due to retirement or change in duties/deliverables within your organisation?

- YES
- NO

Please comment

Preview

[illegible] needs do you forsee you will have recruiting relevant personnel for the coming 4 - 7 years? Please address your needs for personnel with general toxicology and related science educations, versus highly specified education.

9. Please indicate your experience related to how easy it is to recruit competencies in chemical risk assessment/ communication you want to have/hire. Please use a number between 1 (easy to recruit) and 6 (difficult to recruit).

- ◯ 1. Easy to recruit
- ◯ 2.
- ◯ 3.
- ◯ 4.
- ◯ 5.
- ◯ 6. Difficult to recruit

10. Indicate if academia delivers sufficient number of candidates to fulfill your needs (master and/or PhD level)?

11. Do you find it necessary to train your new personnel in the areas of chemical risk assessment/communication due to limited/poor knowledge from academia or previous affiliation(s)/work experience?

- ◯ YES
- ◯ NO

If YES, please comment on how you train the personnel.

Preview

[illegible]h areas of expertise areas are primarily lacking when you want to hire new personnel?

- [ ] Alternative (non-animal) in vitro methods
- [ ] Animal testing
- [ ] Bioinformatics
- [ ] Chemical analysis
- [ ] Chemistry/Environmental chemistry
- [ ] Ecotoxicology
- [ ] Epidemiology
- [ ] Exposure assessment
- [ ] QSAR and read-across
- [ ] Risk assessment
- [ ] Risk communication
- [ ] Risk management
- [ ] Statistics
- [ ] Systematic literature reviews
- [ ] Toxicology
- [ ] Other (please comment below)

Comment

13. Please suggest university courses related to chemical risk assessment/communication you would like to see offered by academia in a near future.

Preview

[illegible]se indicate, if you expect a lack of expertise in the area of risk assessment/ communication in your organisation, in the near future.

- ◯ 1. Not expected
- ◯ 2.
- ◯ 3.
- ◯ 4.
- ◯ 5.
- ◯ 6. Definitely expected

15. Please explain how you foresee to meet the needs of highly competent/qualified personnel in chemical risk assessment/communication in your organization five years from now (e.g. external courses, workshops or internal courses).

16. Please give suggestions what can be done nationally to optimize the numbers of competent persons for your organization.

17. Please suggest how the Nordic countries could act jointly to optimize the numbers of competent persons for your organization.

Preview

18. Do you see positive possibilities for a closer, formalized cooperation between the Nordic countries in the area of chemical risk analysis, including training?

- YES
- NO

If YES - How could this cooperation be accomplished? / If NO - Why would that be hard to accomplish?

19. How many of your experts within chemical risk assessment/communication are involved in international assignments related to the organizations indicated below?

EFSA panels:

ECHA expert groups and member state committee:

EC scientific committees:

EMA committees or working groups:

OECD working groups:

WHO expert groups:

Other(s) (please comment below)

Comment

20. Do you expect that the number of personnel involved in international assignments (c.f. previous question) will increase or decrease in the coming 5 years?

- Increase
- Decrease
- No change is expected

Please comment.

Preview

[illegible]. Any additional reflections or comments regarding competence provision needs in the area of risk assessment/communication?

Save Submit

SI Part C

System generated analyses of the result from the Danish respondents

Risk analysis competence provision questionnaire for Denmark

Respondents: 24
Answer Count: 7
Answer Frequency: 29,17 %

Please indicate your area of affiliation? (mandatory)

Please indicate your area of affiliation? (mandatory)	Number of responses
Research institute	2 (28,6%)
National authority	2 (28,6%)
Regional authority	0 (0,0%)
Hospital practice	0 (0,0%)
Industry/Business	2 (28,6%)
NGO	0 (0,0%)
Consultant	1 (14,3%)
Other	0 (0,0%)
Total	7 (100,0%)

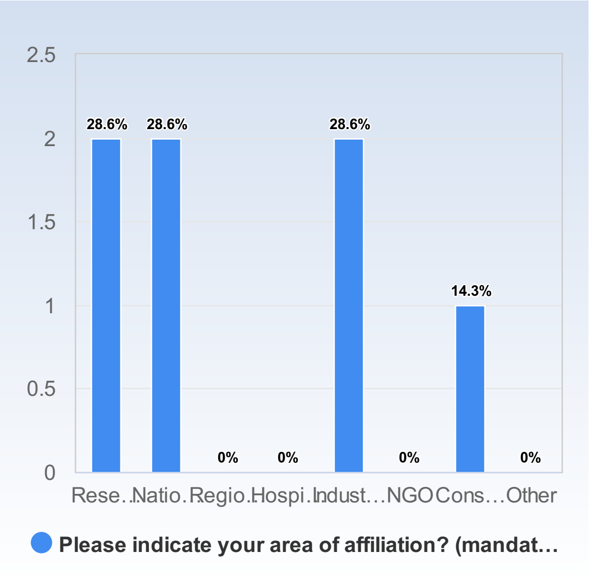

	Mean	Standard Deviation	Coefficient of Variation	Min	Lower Quartile	Median	Upper Quartile	Max
Please indicate your area of affiliation? (mandatory)	4,4	2,6	59,5 %	2,0	2,5	3,0	6,0	9,0

What is the number of personnel in chemical risk assessment/risk communication in your organization?

What is the number of personnel in chemical risk assessment/risk communication in your organization?	Number of responses
0 - 10	3 (42,9%)
11 - 21	2 (28,6%)
22 - 32	1 (14,3%)
33 - 43	0 (0,0%)
44 - 54	0 (0,0%)
55 - 65	0 (0,0%)
66 - 76	0 (0,0%)
77 - 87	0 (0,0%)
88 - 98	0 (0,0%)
99 - 109	1 (14,3%)
Total	7 (100,0%)

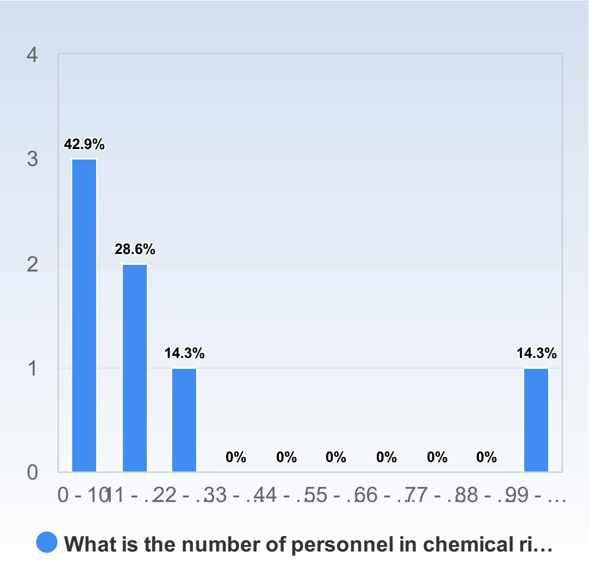

	Mean	Standard Deviation	Coefficient of Variation	Min	Lower Quartile	Median	Upper Quartile	Max
What is the number of personnel in chemical risk assessment/risk communication in your organization?	24,7	34,8	141,0 %	0,0	4,0	15,0	25,0	100,0

What specialization, in chemical risk assessment /communication, do you have in your organization?
Please estimate the number of personnel for each specialization.

Alternative (non-animal) in vitro methods

Alternative (non-animal) in vitro methods	Number of responses
0	0 (0,0%)
1	0 (0,0%)
2	3 (75,0%)
3	0 (0,0%)
4	1 (25,0%)
5	0 (0,0%)
6	0 (0,0%)
7	0 (0,0%)
8	0 (0,0%)
9	0 (0,0%)
Total	4 (100,0%)

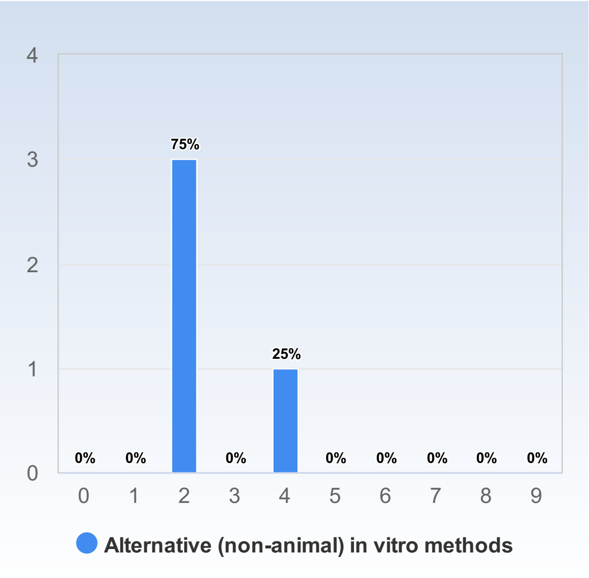

	Mean	Standard Deviation	Coefficient of Variation	Min	Lower Quartile	Median	Upper Quartile	Max
Alternative (non-animal) in vitro methods	2,5	1,0	40,0 %	2,0	2,0	2,0	3,0	4,0

Animal testing

Animal testing	Number of responses
2 - 5	1 (33,3%)
6 - 9	1 (33,3%)
10 - 13	0 (0,0%)
14 - 17	0 (0,0%)
18 - 21	0 (0,0%)
22 - 25	0 (0,0%)
26 - 29	0 (0,0%)
30 - 33	0 (0,0%)
34 - 37	0 (0,0%)
38 - 41	1 (33,3%)
Total	3 (100,0%)

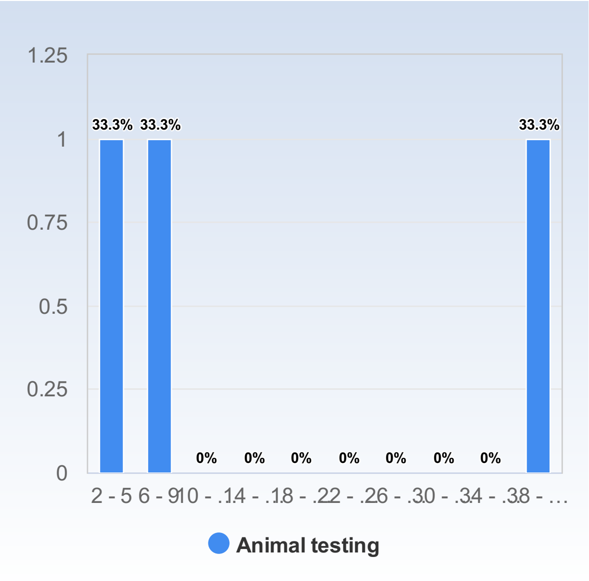

	Mean	Standard Deviation	Coefficient of Variation	Min	Lower Quartile	Median	Upper Quartile	Max
Animal testing	16,7	20,4	122,6 %	2,0	5,0	8,0	24,0	40,0

Bioinformatics

Bioinformatics	Number of responses
0	1 (33,3%)
1	1 (33,3%)
2	1 (33,3%)
3	0 (0,0%)
4	0 (0,0%)
5	0 (0,0%)
6	0 (0,0%)
7	0 (0,0%)
8	0 (0,0%)
9	0 (0,0%)
Total	3 (100,0%)

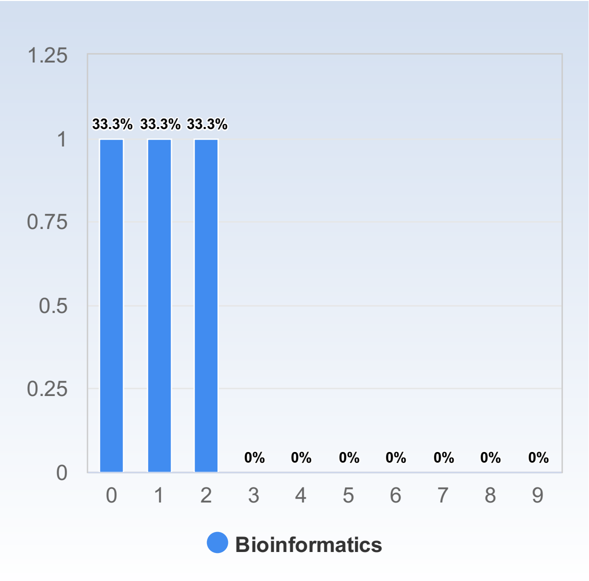

	Mean	Standard Deviation	Coefficient of Variation	Min	Lower Quartile	Median	Upper Quartile	Max
Bioinformatics	1,0	1,0	100,0 %	0,0	0,5	1,0	1,5	2,0

Chemical analysis

Chemical analysis	Number of responses
2	0 (0,0%)
3	1 (33,3%)
4	1 (33,3%)
5	0 (0,0%)
6	0 (0,0%)
7	0 (0,0%)
8	0 (0,0%)
9	0 (0,0%)
10	1 (33,3%)
11	0 (0,0%)
Total	3 (100,0%)

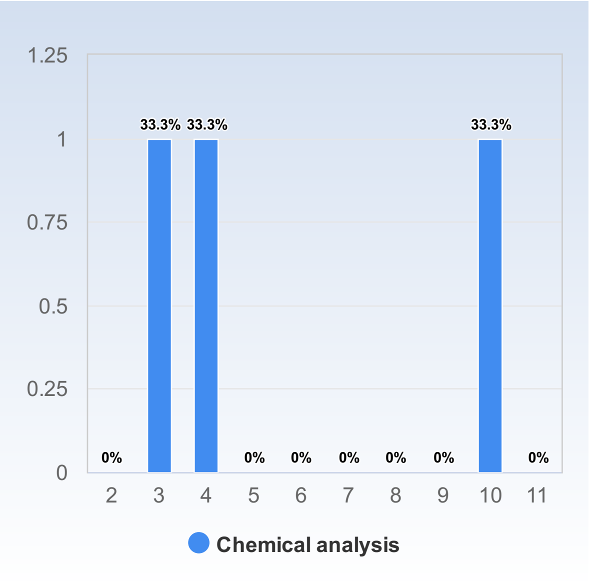

	Mean	Standard Deviation	Coefficient of Variation	Min	Lower Quartile	Median	Upper Quartile	Max
Chemical analysis	5,7	3,8	66,8 %	3,0	3,5	4,0	7,0	10,0

Chemistry/Environmental chemistry

Chemistry/Environmental chemistry	Number of responses
0 - 2	1 (50,0%)
3 - 5	0 (0,0%)
6 - 8	0 (0,0%)
9 - 11	0 (0,0%)
12 - 14	0 (0,0%)
15 - 17	0 (0,0%)
18 - 20	0 (0,0%)
21 - 23	0 (0,0%)
24 - 26	1 (50,0%)
27 - 29	0 (0,0%)
Total	2 (100,0%)

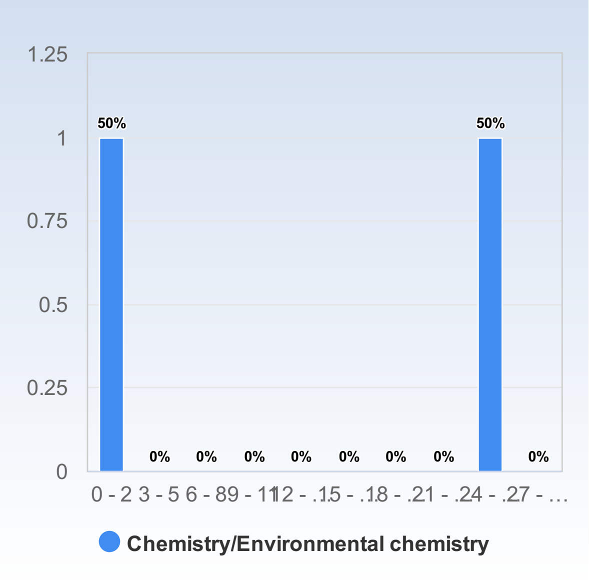

	Mean	Standard Deviation	Coefficient of Variation	Min	Lower Quartile	Median	Upper Quartile	Max
Chemistry/Environmental chemistry	13,5	16,3	120,5 %	2,0	13,5	13,5	13,5	25,0

Ecotoxicology

Ecotoxicology	Number of responses
0 - 2	2 (50,0%)
3 - 5	1 (25,0%)
6 - 8	0 (0,0%)
9 - 11	0 (0,0%)
12 - 14	0 (0,0%)
15 - 17	0 (0,0%)
18 - 20	0 (0,0%)
21 - 23	0 (0,0%)
24 - 26	1 (25,0%)
27 - 29	0 (0,0%)
Total	4 (100,0%)

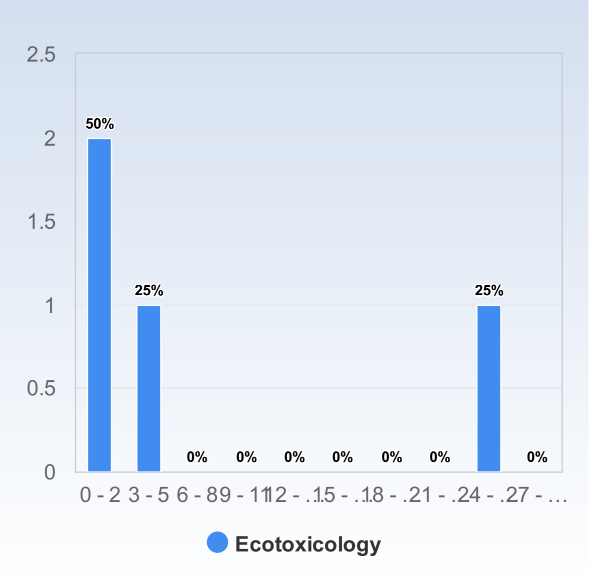

	Mean	Standard Deviation	Coefficient of Variation	Min	Lower Quartile	Median	Upper Quartile	Max
Ecotoxicology	7,5	11,7	156,4 %	0,0	1,0	2,5	14,0	25,0

Epidemiology

Epidemiology	Number of responses
0	0 (0,0%)
1	2 (50,0%)
2	2 (50,0%)
3	0 (0,0%)
4	0 (0,0%)
5	0 (0,0%)
6	0 (0,0%)
7	0 (0,0%)
8	0 (0,0%)
9	0 (0,0%)
Total	4 (100,0%)

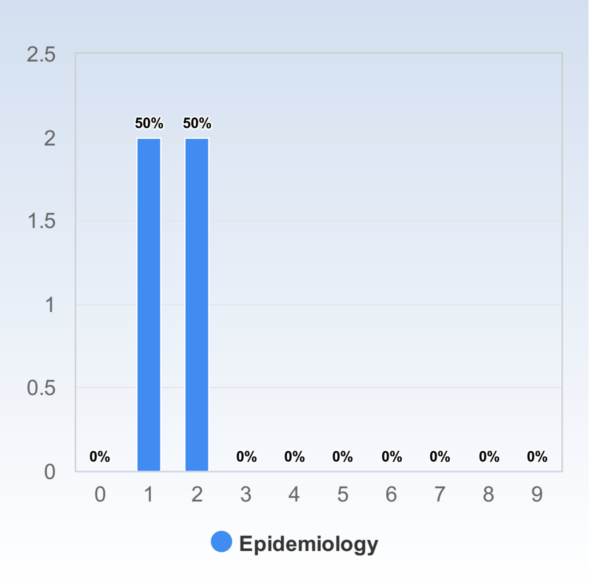

	Mean	Standard Deviation	Coefficient of Variation	Min	Lower Quartile	Median	Upper Quartile	Max
Epidemiology	1,5	0,6	38,5 %	1,0	1,0	1,5	2,0	2,0

Exposure assessment

Exposure assessment	Number of responses
2 - 7	3 (75,0%)
8 - 13	0 (0,0%)
14 - 19	0 (0,0%)
20 - 25	0 (0,0%)
26 - 31	0 (0,0%)
32 - 37	0 (0,0%)
38 - 43	0 (0,0%)
44 - 49	0 (0,0%)
50 - 55	0 (0,0%)
56 - 61	1 (25,0%)
Total	4 (100,0%)

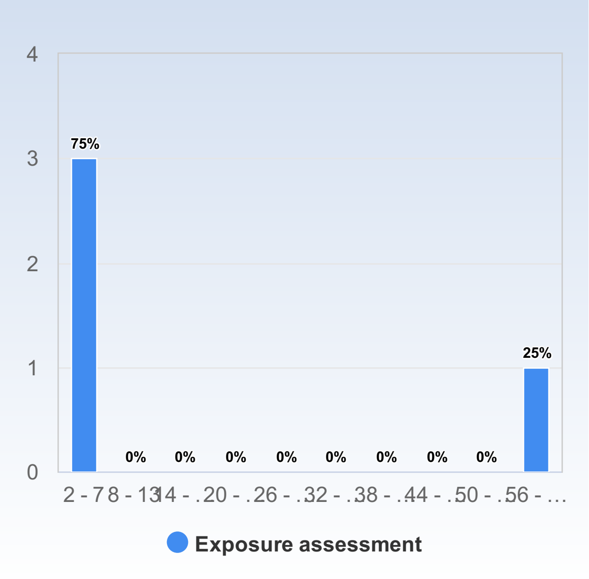

	Mean	Standard Deviation	Coefficient of Variation	Min	Lower Quartile	Median	Upper Quartile	Max
Exposure assessment	17,5	28,4	162,3 %	2,0	2,0	4,0	33,0	60,0

QSAR and read-across

QSAR and read-across	Number of responses
0	0 (0,0%)
1	3 (60,0%)
2	1 (20,0%)
3	0 (0,0%)
4	0 (0,0%)
5	1 (20,0%)
6	0 (0,0%)
7	0 (0,0%)
8	0 (0,0%)
9	0 (0,0%)
Total	5 (100,0%)

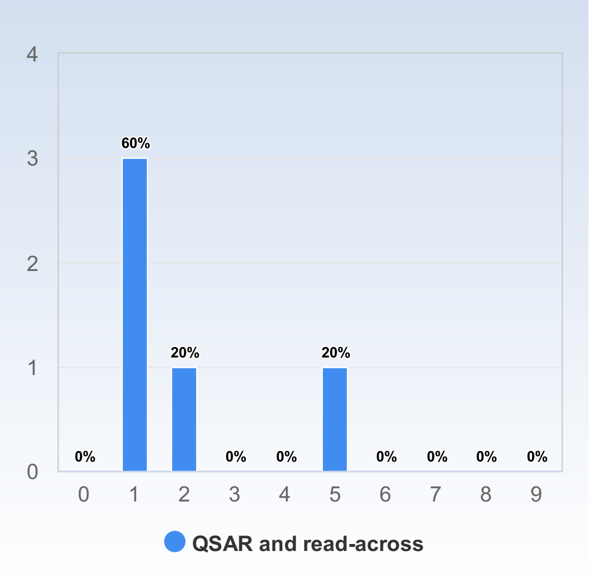

	Mean	Standard Deviation	Coefficient of Variation	Min	Lower Quartile	Median	Upper Quartile	Max
QSAR and read-across	2,0	1,7	86,6 %	1,0	1,0	1,0	2,0	5,0

Risk assessment

Risk assessment	Number of responses
1 - 6	3 (60,0%)
7 - 12	1 (20,0%)
13 - 18	0 (0,0%)
19 - 24	0 (0,0%)
25 - 30	0 (0,0%)
31 - 36	0 (0,0%)
37 - 42	0 (0,0%)
43 - 48	0 (0,0%)
49 - 54	0 (0,0%)
55 - 60	1 (20,0%)
Total	5 (100,0%)

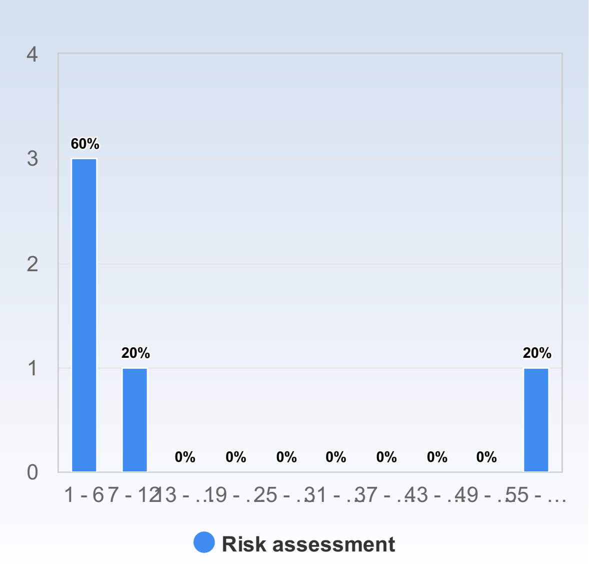

	Mean	Standard Deviation	Coefficient of Variation	Min	Lower Quartile	Median	Upper Quartile	Max
Risk assessment	15,2	25,2	165,7 %	1,0	2,0	5,0	8,0	60,0

Risk communication

Risk communication	Number of responses
2	1 (20,0%)
3	1 (20,0%)
4	0 (0,0%)
5	1 (20,0%)
6	0 (0,0%)
7	0 (0,0%)
8	1 (20,0%)
9	0 (0,0%)
10	1 (20,0%)
11	0 (0,0%)
Total	5 (100,0%)

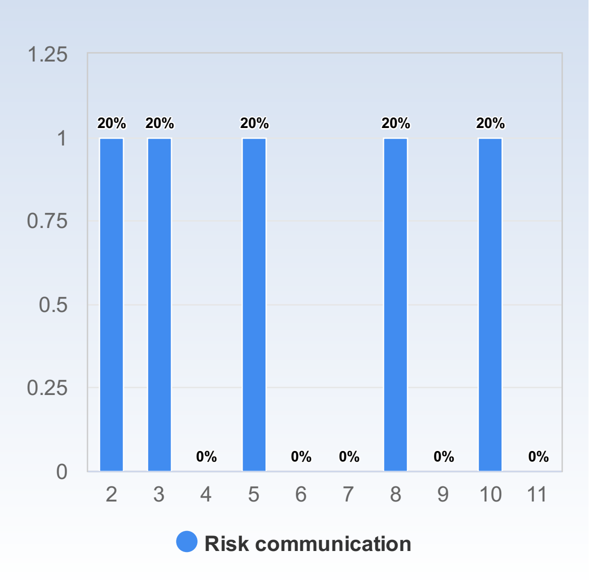

	Mean	Standard Deviation	Coefficient of Variation	Min	Lower Quartile	Median	Upper Quartile	Max
Risk communication	5,6	3,4	60,0 %	2,0	3,0	5,0	8,0	10,0

Risk management

Risk management	Number of responses
1 - 6	2 (50,0%)
7 - 12	1 (25,0%)
13 - 18	0 (0,0%)
19 - 24	0 (0,0%)
25 - 30	0 (0,0%)
31 - 36	0 (0,0%)
37 - 42	0 (0,0%)
43 - 48	0 (0,0%)
49 - 54	0 (0,0%)
55 - 60	1 (25,0%)
Total	4 (100,0%)

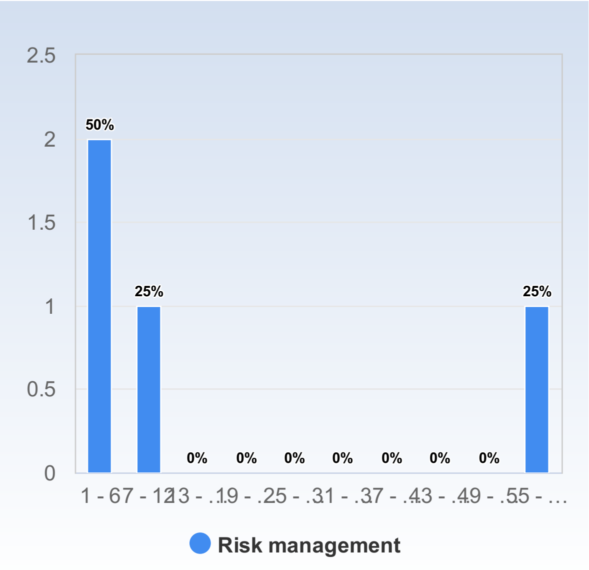

	Mean	Standard Deviation	Coefficient of Variation	Min	Lower Quartile	Median	Upper Quartile	Max
Risk management	18,5	27,8	150,4 %	1,0	3,0	6,5	34,0	60,0

Statistics

Statistics	Number of responses
0	2 (100,0%)
1	0 (0,0%)
2	0 (0,0%)
3	0 (0,0%)
4	0 (0,0%)
5	0 (0,0%)
6	0 (0,0%)
7	0 (0,0%)
8	0 (0,0%)
9	0 (0,0%)
Total	2 (100,0%)

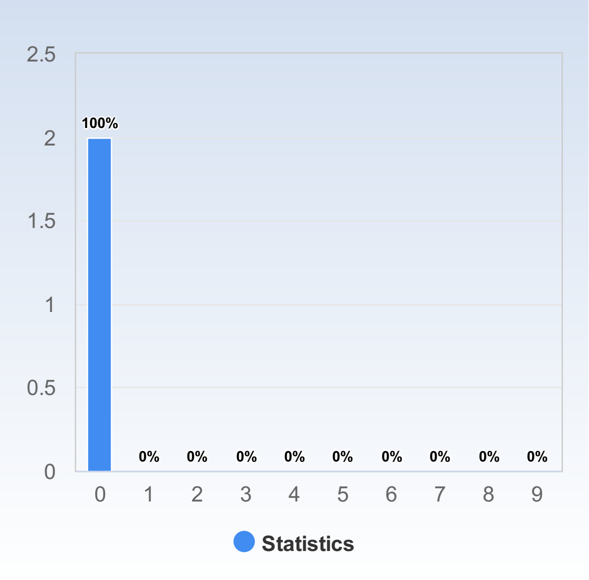

	Mean	Standard Deviation	Coefficient of Variation	Min	Lower Quartile	Median	Upper Quartile	Max
Statistics	0,0	0,0	NaN %	0,0	0,0	0,0	0,0	0,0

Systematic literature reviews

Systematic literature reviews	Number of responses
0	1 (25,0%)
1	0 (0,0%)
2	0 (0,0%)
3	2 (50,0%)
4	0 (0,0%)
5	1 (25,0%)
6	0 (0,0%)
7	0 (0,0%)
8	0 (0,0%)
9	0 (0,0%)
Total	4 (100,0%)

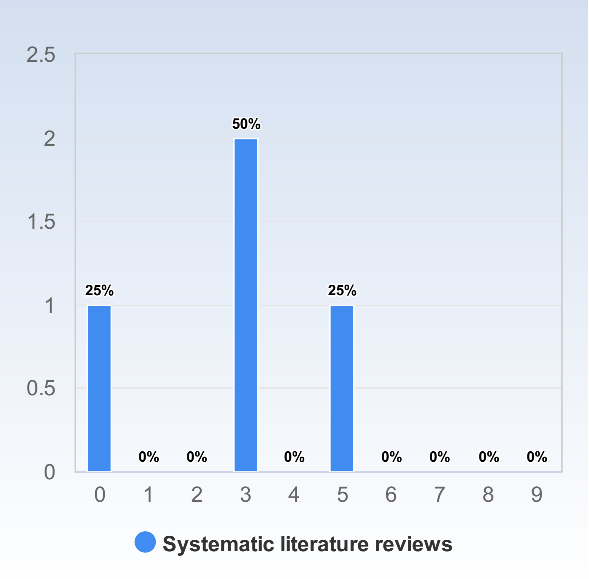

	Mean	Standard Deviation	Coefficient of Variation	Min	Lower Quartile	Median	Upper Quartile	Max
Systematic literature reviews	2,8	2,1	75,0 %	0,0	1,5	3,0	4,0	5,0

Toxicology

Toxicology	Number of responses
1 - 4	2 (40,0%)
5 - 8	0 (0,0%)
9 - 12	1 (20,0%)
13 - 16	0 (0,0%)
17 - 20	0 (0,0%)
21 - 24	0 (0,0%)
25 - 28	0 (0,0%)
29 - 32	1 (20,0%)
33 - 36	0 (0,0%)
37 - 40	1 (20,0%)
Total	5 (100,0%)

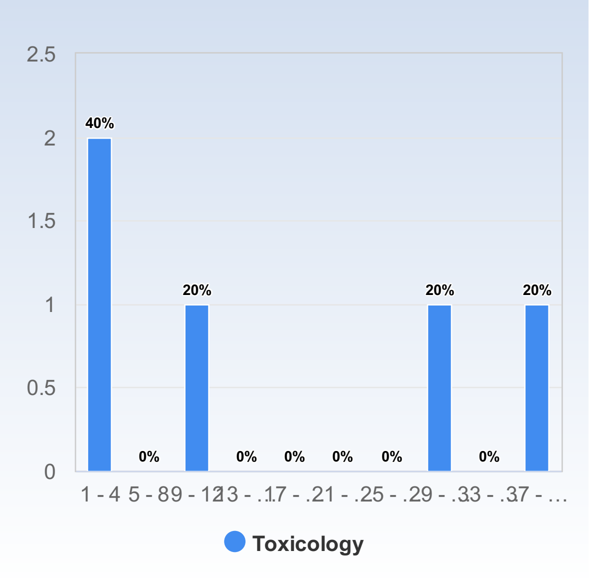

	Mean	Standard Deviation	Coefficient of Variation	Min	Lower Quartile	Median	Upper Quartile	Max
Toxicology	17,0	17,1	100,7 %	1,0	4,0	10,0	30,0	40,0

Other (please comment below)

Other (please comment below)	Number of responses
0	1 (50,0%)
1	1 (50,0%)
2	0 (0,0%)
3	0 (0,0%)
4	0 (0,0%)
5	0 (0,0%)
6	0 (0,0%)
7	0 (0,0%)
8	0 (0,0%)
9	0 (0,0%)
Total	2 (100,0%)

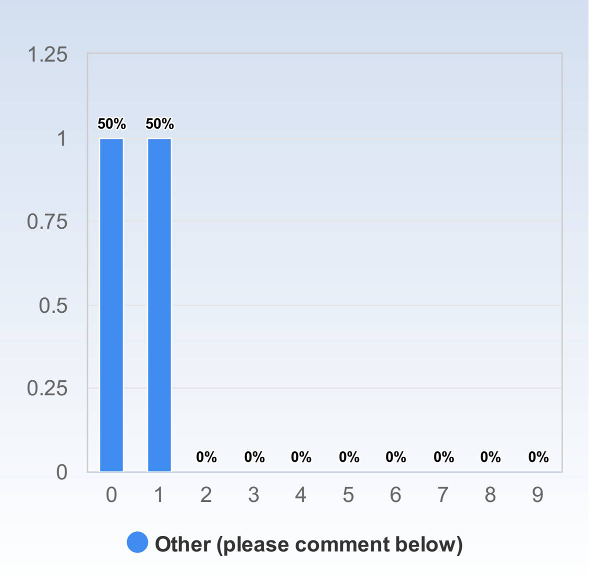

	Mean	Standard Deviation	Coefficient of Variation	Min	Lower Quartile	Median	Upper Quartile	Max
Other (please comment below)	0,5	0,7	141,4 %	0,0	0,5	0,5	0,5	1,0

Comment
Toxicogenomics
The figure 30 is specifically those considered as "toxicologist". They are all involved in in vitro/in vivo methods, exposure and risk assessment. The total number of personell working in all these fields are large, but not all focus on toxicology as a discipline. Numbers of those working with testing are higher than given here, as toxicology studies are outsourced. In addition the number of chemical analysis, ADME and assay scientists are many (not counted here).
On the previous page only employees with research functions are indicated, not technicians<br>I have tried to indicate the person years allocated to each task.

How many of the personnel in your organization, with the chemical risk assessment /communication tasks you presented in the previous question, holds a:

Bachelor degree:

Bachelor degree:	Number of responses
0 - 2	2 (50,0%)
3 - 5	0 (0,0%)
6 - 8	0 (0,0%)
9 - 11	0 (0,0%)
12 - 14	0 (0,0%)
15 - 17	1 (25,0%)
18 - 20	1 (25,0%)
21 - 23	0 (0,0%)
24 - 26	0 (0,0%)
27 - 29	0 (0,0%)
Total	4 (100,0%)

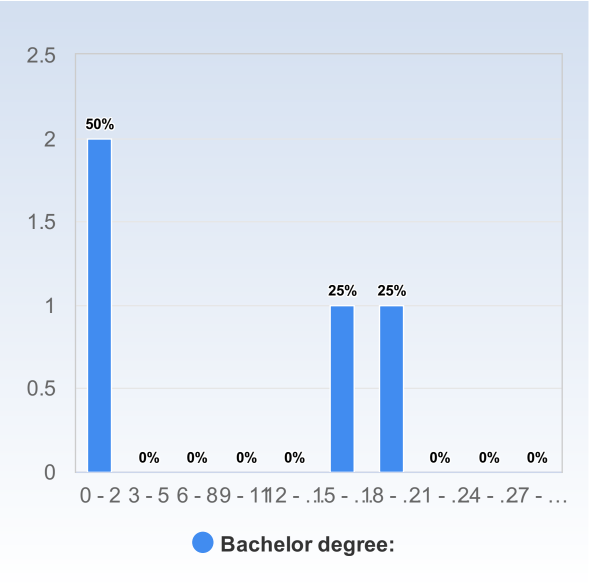

	Mean	Standard Deviation	Coefficient of Variation	Min	Lower Quartile	Median	Upper Quartile	Max
Bachelor degree:	9,3	9,8	105,7 %	0,0	1,0	8,5	17,5	20,0

Master degree:

Master degree:	Number of responses
2 - 8	2 (40,0%)
9 - 15	1 (20,0%)
16 - 22	1 (20,0%)
23 - 29	0 (0,0%)
30 - 36	0 (0,0%)
37 - 43	0 (0,0%)
44 - 50	0 (0,0%)
51 - 57	0 (0,0%)
58 - 64	0 (0,0%)
65 - 71	1 (20,0%)
Total	5 (100,0%)

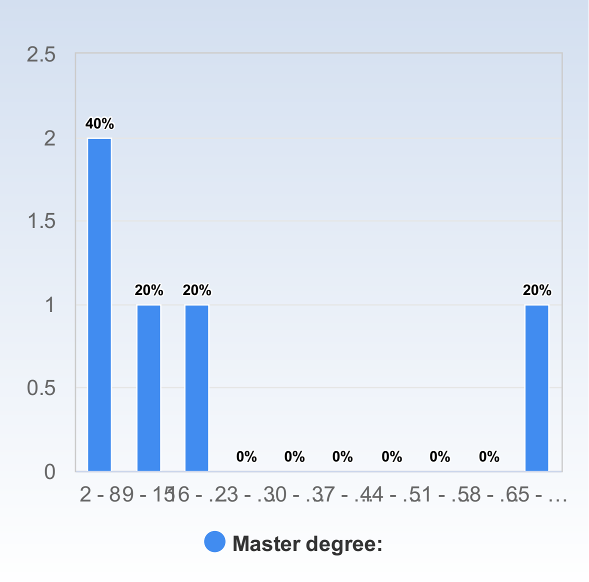

	Mean	Standard Deviation	Coefficient of Variation	Min	Lower Quartile	Median	Upper Quartile	Max
Master degree:	22,4	27,6	123,1 %	3,0	4,0	15,0	20,0	70,0

Doctoral degree (PhDs, MDs, Dtech (TkT, D.I.T), etc):

Doctoral degree (PhDs, MDs, Dtech (TkT, D.I.T), etc):	Number of responses
2 - 4	1 (20,0%)
5 - 7	0 (0,0%)
8 - 10	0 (0,0%)
11 - 13	0 (0,0%)
14 - 16	1 (20,0%)
17 - 19	1 (20,0%)
20 - 22	0 (0,0%)
23 - 25	0 (0,0%)
26 - 28	1 (20,0%)
29 - 31	1 (20,0%)
Total	5 (100,0%)

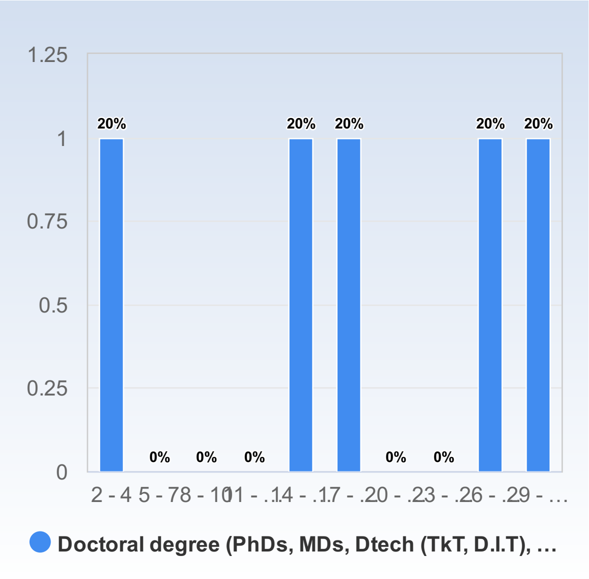

	Mean	Standard Deviation	Coefficient of Variation	Min	Lower Quartile	Median	Upper Quartile	Max
Doctoral degree (PhDs, MDs, Dtech (TkT, D.I.T), etc):	18,2	10,9	59,7 %	2,0	15,0	18,0	26,0	30,0

How many of your personnel are ERTs (European Registered Toxicologist)?

How many of your personnel are ERTs (European Registered Toxicologist)?	Number of responses
0 - 10	6 (100,0%)
11 - 21	0 (0,0%)
22 - 32	0 (0,0%)
33 - 43	0 (0,0%)
44 - 54	0 (0,0%)
55 - 65	0 (0,0%)
66 - 76	0 (0,0%)
77 - 87	0 (0,0%)
88 - 98	0 (0,0%)
99 - 109	0 (0,0%)
Total	6 (100,0%)

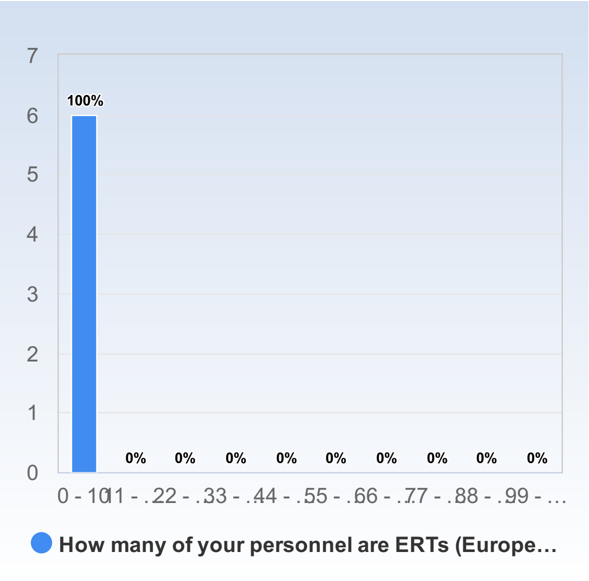

	Mean	Standard Deviation	Coefficient of Variation	Min	Lower Quartile	Median	Upper Quartile	Max
How many of your personnel are ERTs (European Registered Toxicologist)?	2,8	3,9	138,4 %	0,0	0,0	1,5	3,5	10,0

What is the present age profile of the risk analysis personnel in your organization?

< 40 years of age:

< 40 years of age:	Number of responses
2 - 6	2 (50,0%)
7 - 11	1 (25,0%)
12 - 16	0 (0,0%)
17 - 21	0 (0,0%)
22 - 26	0 (0,0%)
27 - 31	0 (0,0%)
32 - 36	0 (0,0%)
37 - 41	0 (0,0%)
42 - 46	0 (0,0%)
47 - 51	1 (25,0%)
Total	4 (100,0%)

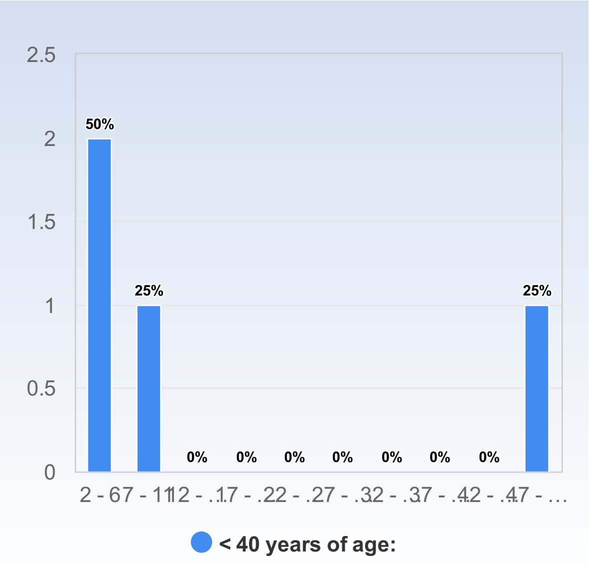

	Mean	Standard Deviation	Coefficient of Variation	Min	Lower Quartile	Median	Upper Quartile	Max
< 40 years of age:	16,3	22,6	138,8 %	3,0	4,0	6,0	28,5	50,0

40-50 years of age:

40-50 years of age:	Number of responses
1 - 3	1 (16,7%)
4 - 6	1 (16,7%)
7 - 9	2 (33,3%)
10 - 12	1 (16,7%)
13 - 15	0 (0,0%)
16 - 18	0 (0,0%)
19 - 21	0 (0,0%)
22 - 24	0 (0,0%)
25 - 27	0 (0,0%)
28 - 30	1 (16,7%)
Total	6 (100,0%)

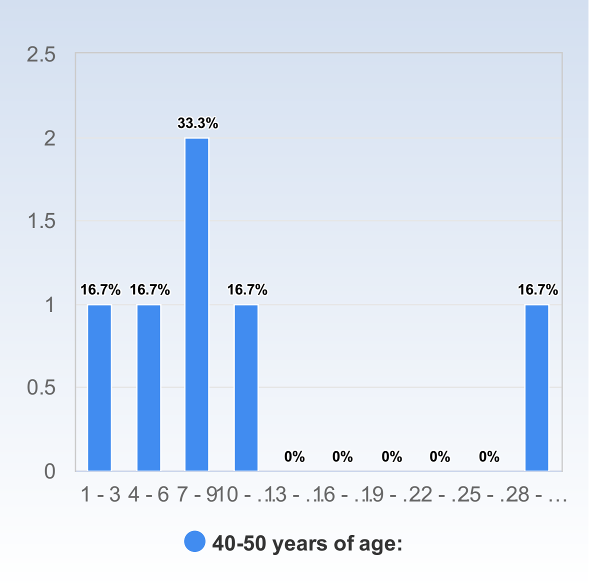

	Mean	Standard Deviation	Coefficient of Variation	Min	Lower Quartile	Median	Upper Quartile	Max
40-50 years of age:	10,2	10,3	101,0 %	1,0	5,5	8,0	9,5	30,0

50-60 years of age:

50-60 years of age:	Number of responses
1	2 (33,3%)
2	0 (0,0%)
3	1 (16,7%)
4	0 (0,0%)
5	1 (16,7%)
6	0 (0,0%)
7	0 (0,0%)
8	0 (0,0%)
9	0 (0,0%)
10	2 (33,3%)
Total	6 (100,0%)

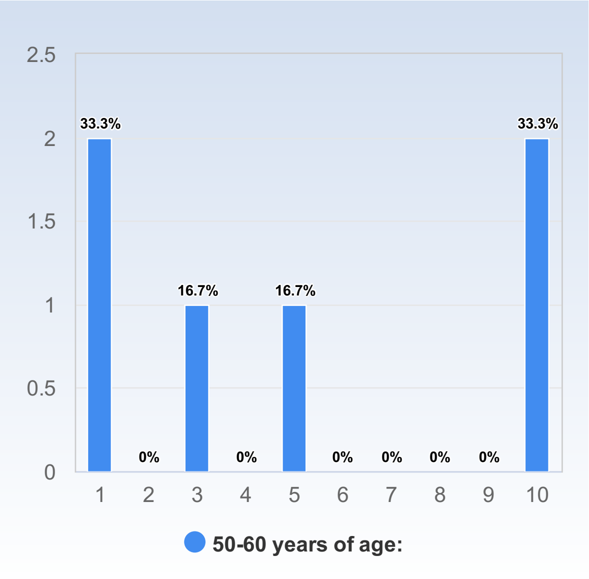

	Mean	Standard Deviation	Coefficient of Variation	Min	Lower Quartile	Median	Upper Quartile	Max
50-60 years of age:	5,0	4,1	82,9 %	1,0	2,0	4,0	7,5	10,0

60-65 years of age:

60-65 years of age:	Number of responses
1	1 (33,3%)
2	0 (0,0%)
3	0 (0,0%)
4	0 (0,0%)
5	1 (33,3%)
6	0 (0,0%)
7	0 (0,0%)
8	0 (0,0%)
9	0 (0,0%)
10	1 (33,3%)
Total	3 (100,0%)

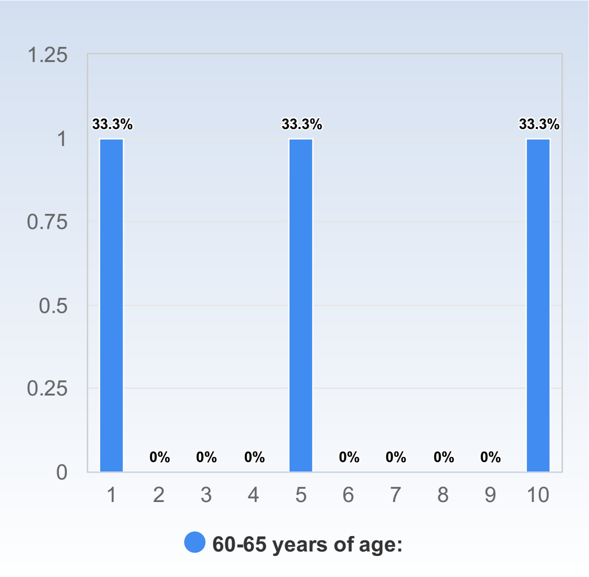

	Mean	Standard Deviation	Coefficient of Variation	Min	Lower Quartile	Median	Upper Quartile	Max
60-65 years of age:	5,3	4,5	84,5 %	1,0	3,0	5,0	7,5	10,0

> 65 years of age:

> 65 years of age:	Number of responses
0	0 (0,0%)
1	1 (100,0%)
2	0 (0,0%)
3	0 (0,0%)
4	0 (0,0%)
5	0 (0,0%)
6	0 (0,0%)
7	0 (0,0%)
8	0 (0,0%)
9	0 (0,0%)
Total	1 (100,0%)

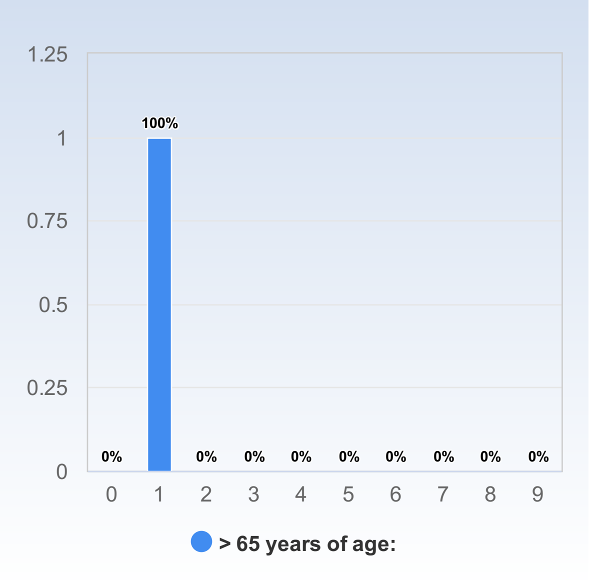

	Mean	Standard Deviation	Coefficient of Variation	Min	Lower Quartile	Median	Upper Quartile	Max
> 65 years of age:	1,0	0,0	NaN %	1,0	1,0	1,0	1,0	1,0

Is there a need for hiring of replacement /expanding the number of chemical risk assessment/communication personnel over the next 5-10 years in your organization, e.g. due to retirement or change in duties/deliverables within your organisation?

Is there a need for hiring of replacement /expanding the number of chemical risk assessment/communication personnel over the next 5-10 years in your organization, e.g. due to retirement or change in duties/deliverables within your organisation?	Number of responses
YES	6 (100,0%)
NO	0 (0,0%)
Total	6 (100,0%)

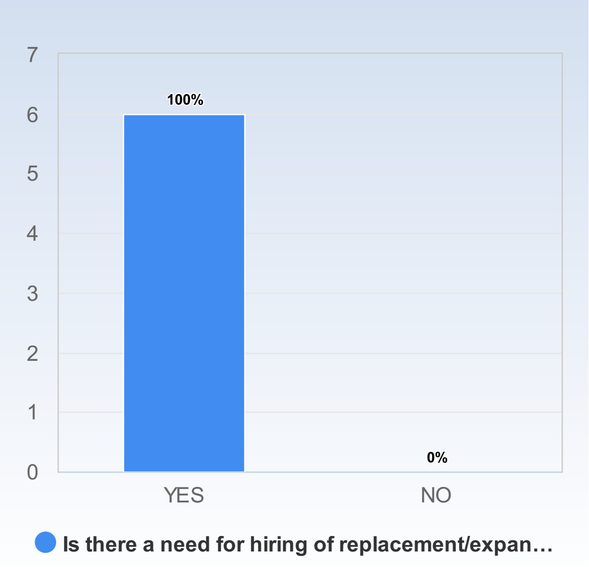

	Mean	Standard Deviation	Coefficient of Variation	Min	Lower Quartile	Median	Upper Quartile	Max
Is there a need for hiring of replacement/expanding the number of chemical risk assessment/communication personnel over the next 5-10 years in your organization, e.g. due to retirement or change in duties/deliverables within your organisation?	1,0	0,0	0,0 %	1,0	1,0	1,0	1,0	1,0

Please comment
probably, but entirely depending on funding
To a small degree based on natural changeover.
No expected change in number of personel. Continuous replacement.
We have vacant positions in especially exposure assessment, and additional recruitment might be relevant depending on potential increase in funding.<br>Recruitment due to retirement will not be relevant the next 10 years.

Which needs do you forsee you will have recruiting relevant personnel for the coming 4 - 7 years? Please address your needs for personnel with general toxicology and related science educations, versus highly specified education.

Which needs do you forsee you will have recruiting relevant personnel for the coming 4 - 7 years? Please address your needs for personnel with general toxicology and related science educations, versus highly specified education.
hopefully we will need additional expertise in exposure assessment and risk assessment. We will need expertise at the higest levels
Expertise in probabilistic risk assessment based on artificial intelligence and novel approach methodologies (NAMs) and exposure modelling.
The primary need is for personel with experince in regulatory (eco)toxicology. Both highly specialized and more general competences are needed for different tasks.
We expect to be able to recruit general scientists and provide education in toxicology.<br>Challenge will be to include scientists strong in computational skills with insight in our field
Personnel in field exposure assessment/monitoring (science education)<br>Epidemiologists with skills in register studies (public health education)<br>Maybe toxicologists

Please indicate your experience related to how easy it is to recruit competencies in chemical risk assessment/ communication you want to have /hire. Please use a number between 1 (easy to recruit) and 6 (difficult to recruit).

Please indicate your experience related to how easy it is to recruit competencies in chemical risk assessment/ communication you want to have /hire. Please use a number between 1 (easy to recruit) and 6 (difficult to recruit).	Number of responses
1. Easy to recruit	0 (0,0%)
2.	0 (0,0%)
3.	2 (33,3%)
4.	2 (33,3%)
5.	2 (33,3%)
6. Difficult to recruit	0 (0,0%)
Total	6 (100,0%)

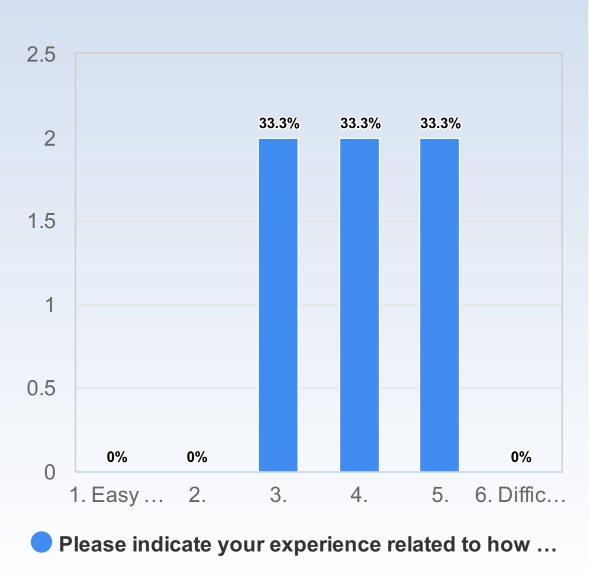

	Mean	Standard Deviation	Coefficient of Variation	Min	Lower Quartile	Median	Upper Quartile	Max
Please indicate your experience related to how easy it is to recruit competencies in chemical risk assessment/ communication you want to have /hire. Please use a number between 1 (easy to recruit) and 6 (difficult to recruit) .	4,0	0,9	22,4 %	3,0	3,5	4,0	4,5	5,0

# Indicate if academia delivers sufficient number of candidates to fulfill your needs (master and/or PhD level)?

Indicate if academia delivers sufficient number of candidates to fulfill your needs (master and/or PhD level)?
PhD level
Not (yet) with the expertise mentioned earlier (probabilistic risk assessment, AI, NAMs, exposure modelling)
Not sufficient with regard to toxicologists. Difficult for public administration to compete with private companies.
Yes, as we can provide further competence development.<br>However, a general concern exists as there is strong competition in getting the best candidates.
For exposure assessment, there are not sufficient candidates.<br>It is also difficult in other fields, maybe not so much in toxicology

Do you find it necessary to train your new personnel in the areas of chemical risk assessment/communication due to limited/poor knowledge from academia or previous affiliation (s)/work experience?

Do you find it necessary to train your new personnel in the areas of chemical risk assessment/communication due to limited/poor knowledge from academia or previous affiliation(s) /work experience?	Number of responses
YES	6 (100,0%)
NO	0 (0,0%)
Total	6 (100,0%)

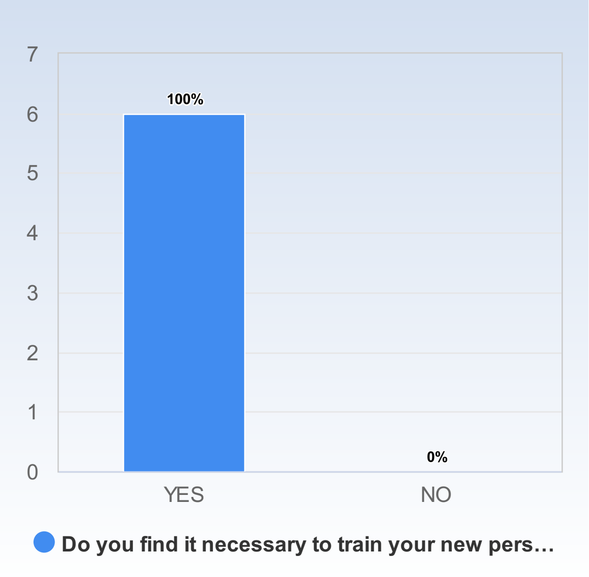

	Mean	Standard Deviation	Coefficient of Variation	Min	Lower Quartile	Median	Upper Quartile	Max
Do you find it necessary to train your new personnel in the areas of chemical risk assessment/communication due to limited/poor knowledge from academia or previous affiliation(s)/work experience?	1,0	0,0	0,0 %	1,0	1,0	1,0	1,0	1,0

If YES, please comment on how you train the personnel.
on job training, NIVA courses,
Courses, webinars
Regulatory knowledge is often insufficient. Very different levels of scientific competences from newly recruited staff. Depends amongst others on the level of specialisation during the studies.
Task specific training needed.<br>Internal and external courses, "sidemandsoplæring"

Which areas of expertise areas are primarily lacking when you want to hire new personnel?

Which areas of expertise areas are primarily lacking when you want to hire new personnel?	Number of responses
Alternative (non-animal) in vitro methods	0 (0,0%)
Animal testing	0 (0,0%)
Bioinformatics	1 (16,7%)
Chemical analysis	1 (16,7%)
Chemistry/Environmental chemistry	1 (16,7%)
Ecotoxicology	1 (16,7%)
Epidemiology	1 (16,7%)
Exposure assessment	2 (33,3%)
QSAR and read-across	3 (50,0%)
Risk assessment	0 (0,0%)
Risk communication	0 (0,0%)
Risk management	0 (0,0%)
Statistics	0 (0,0%)
Systematic literature reviews	0 (0,0%)
Toxicology	0 (0,0%)
Other (please comment below)	2 (33,3%)
Total	12 (200,0%)

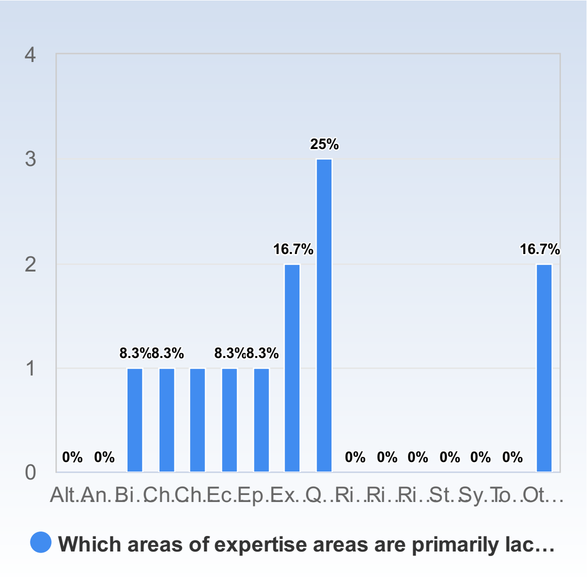

	Mean	Standard Deviation	Coefficient of Variation	Min	Lower Quartile	Median	Upper Quartile	Max
Which areas of expertise areas are primarily lacking when you want to hire new personnel?	19,3	4,1	21,2 %	14,0	16,5	19,0	20,0	27,0

Comment
Probabilistic risk assessment, AI, NAMS and exposure modelling
Not possible to answer since it depends on the specific position.
Management is not always specifically looking for toxicology skills. General scientist/biological skills are not lacking currently.

Please suggest university courses related to chemical risk assessment/communication you would like to see offered by academia in a near future.

Please suggest university courses related to chemical risk assessment/communication you would like to see offered by academia in a near future.
Bench mark dose modelling for quantitative risk assessment
Probabilistic risk assessment based on AI, NAMs (including e.g., RASAR) and exposure modelling
Regulatory toxicology
Use of digital tools, computational toxicology (as we have difficulties in internal education).<br>Risk assessment of materials/impurities (requires multiple skills and experience in materials understanding and chemistry as well as biology)
Course in applied toxicology. There used to be a post graudate course at University of Copenhagen, but it does not seem to be available any more.

Please indicate, if you expect a lack of expertise in the area of risk assessment/ communication in your organisation, in the near future.

Please indicate, if you expect a lack of expertise in the area of risk assessment/ communication in your organisation, in the near future.	Number of responses
1. Not expected	0 (0,0%)
2.	4 (66,7%)
3.	0 (0,0%)
4.	1 (16,7%)
5.	0 (0,0%)
6. Definitely expected	1 (16,7%)
Total	6 (100,0%)

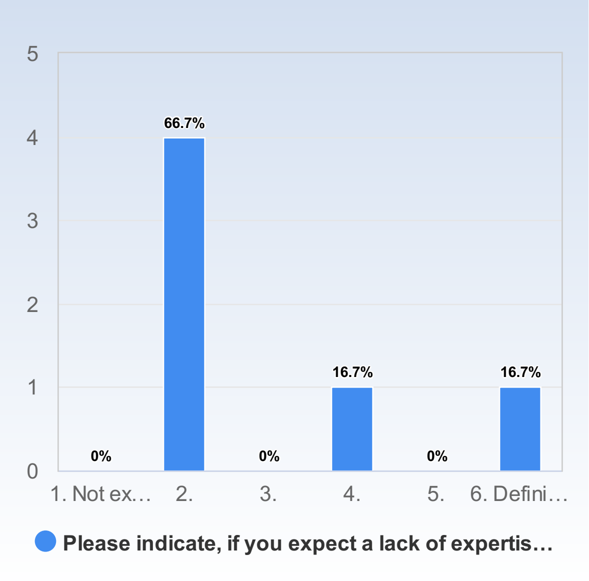

	Mean	Standard Deviation	Coefficient of Variation	Min	Lower Quartile	Median	Upper Quartile	Max
Please indicate, if you expect a lack of expertise in the area of risk assessment/ communication in your organisation, in the near future.	3,0	1,7	55,8 %	2,0	2,0	2,0	3,0	6,0

Please explain how you foresee to meet the needs of highly competent/qualified personnel in chemical risk assessment/communication in your organization five years from now (e.g. external courses, workshops or internal courses).

Please explain how you foresee to meet the needs of highly competent/qualified personnel in chemical risk assessment/communication in your organization five years from now (e.g. external courses, workshops or internal courses).
we educate phd students and post docs, but we also recruit from other institutions, ie universities.
Internal and external courses.
Internal training as mentioned earlier (courses, webinars, ...)
Internal training is most important. Availability of extarnal courses is not meeting the demand currently.
Same expectations as now. Expect to do internal training mainly
Participate in courses, e.g. online and physical courses

Please give suggestions what can be done nationally to optimize the numbers of competent persons for your organization.

Please give suggestions what can be done nationally to optimize the numbers of competent persons for your organization.
It would be great to have a proper toxicology eductation in DK fx a master of toxicology at one of the universities, and to have a PhD school that focusses on different aspects of toxicology
Include more risk analysis courses in relevant educations (such as chemistry, biology etc.).
Organize courses and training that fit into New Generation Risk Assessment (i.e., without the use of animals).
Establishment of a toxicology education.
Course in Applied toxicology in Denmark

Please suggest how the Nordic countries could act <u>jointly</u> to optimize the numbers of competent persons for your organization.

Please suggest how the Nordic countries could act **jointly** to optimize the numbers of competent persons for your organization.
The master of toxicology and the phd school could be a Nordic activity or be coordinated betweeen key institutes in Nordic countries
Co-operate to establish a joint education (cf. ERT).
Start with talking and identifying gaps.
Relevant courses with selected topics.
Joint courses including industry focus
Exchange between countries<br>Common courses

Do you see positive possibilities for a closer, formalized cooperation between the Nordic countries in the area of chemical risk analysis, including training?

Do you see positive possibilities for a closer, formalized cooperation between the Nordic countries in the area of chemical risk analysis, including training?	Number of responses
YES	5 (83,3%)
NO	1 (16,7%)
Total	6 (100,0%)

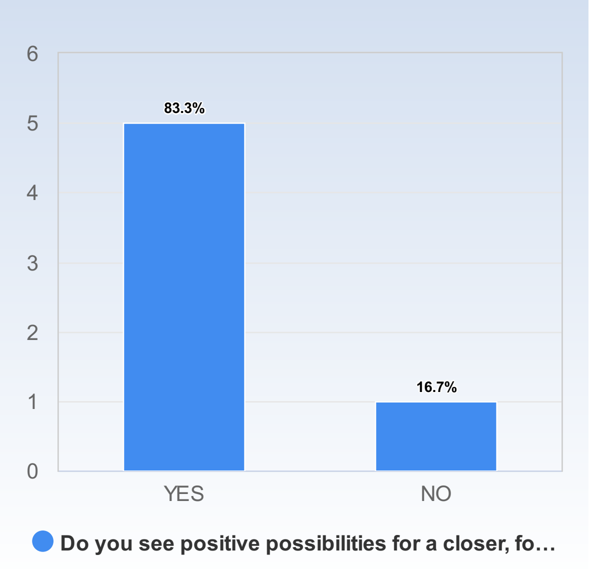

	Mean	Standard Deviation	Coefficient of Variation	Min	Lower Quartile	Median	Upper Quartile	Max
Do you see positive possibilities for a closer, formalized cooperation between the Nordic countries in the area of chemical risk analysis, including training?	1,2	0,4	35,0 %	1,0	1,0	1,0	1,0	2,0

If YES - How could this cooperation be accomplished? / If NO - Why would that be hard to accomplish?
yes using NIVA courses and other Nordic inititatives. it would also be good if the Nordic countries could collaborate closer in risk assessment and regulation fx in occupational health
Co-operate to establish a joint education (cf. ERT).
Agree to implement New generation Risk Assessment, create an understanding of what it implies and identify gaps in the current training and education.
Formalised cooperation allready exists beween authorties (Nordic Chemical Group).
Via the Nordic council of ministers; NIVA

How many of your experts within chemical risk assessment/communication are involved in international assignments related to the organizations indicated below?

EFSA panels:

EFSA panels:	Number of responses
0	1 (20,0%)
1	3 (60,0%)
2	1 (20,0%)
3	0 (0,0%)
4	0 (0,0%)
5	0 (0,0%)
6	0 (0,0%)
7	0 (0,0%)
8	0 (0,0%)
9	0 (0,0%)
Total	5 (100,0%)

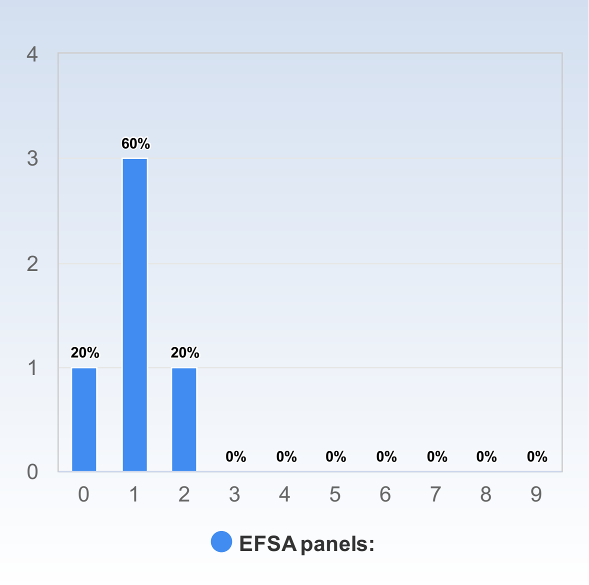

	Mean	Standard Deviation	Coefficient of Variation	Min	Lower Quartile	Median	Upper Quartile	Max
EFSA panels:	1,0	0,7	70,7 %	0,0	1,0	1,0	1,0	2,0

ECHA expert groups and member state committee:

ECHA expert groups and member state committee:	Number of responses
0 - 2	2 (66,7%)
3 - 5	0 (0,0%)
6 - 8	0 (0,0%)
9 - 11	0 (0,0%)
12 - 14	0 (0,0%)
15 - 17	0 (0,0%)
18 - 20	1 (33,3%)
21 - 23	0 (0,0%)
24 - 26	0 (0,0%)
27 - 29	0 (0,0%)
Total	3 (100,0%)

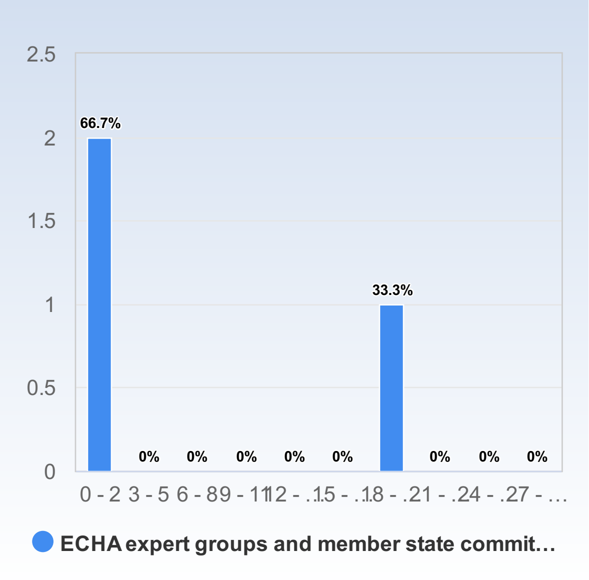

	Mean	Standard Deviation	Coefficient of Variation	Min	Lower Quartile	Median	Upper Quartile	Max
ECHA expert groups and member state committee:	7,0	11,3	161,0 %	0,0	0,5	1,0	10,5	20,0

EC scientific committees:

EC scientific committees:	Number of responses
0	2 (66,7%)
1	1 (33,3%)
2	0 (0,0%)
3	0 (0,0%)
4	0 (0,0%)
5	0 (0,0%)
6	0 (0,0%)
7	0 (0,0%)
8	0 (0,0%)
9	0 (0,0%)
Total	3 (100,0%)

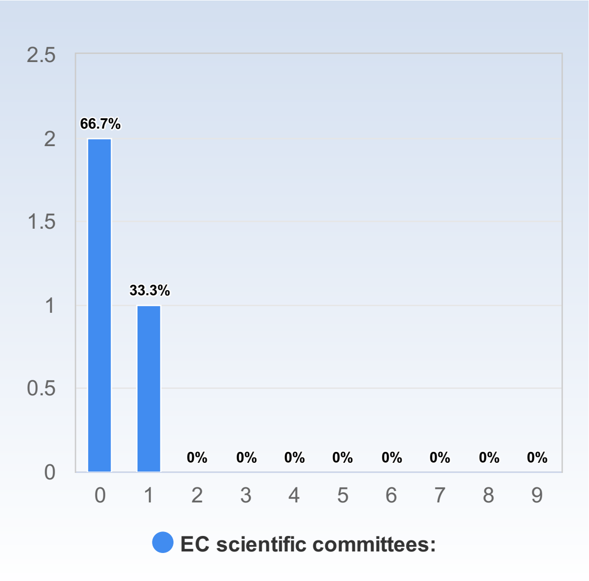

	Mean	Standard Deviation	Coefficient of Variation	Min	Lower Quartile	Median	Upper Quartile	Max
EC scientific committees:	0,3	0,6	173,2 %	0,0	0,0	0,0	0,5	1,0

EMA committees or working groups:

EMA committees or working groups:	Number of responses
0	2 (100,0%)
1	0 (0,0%)
2	0 (0,0%)
3	0 (0,0%)
4	0 (0,0%)
5	0 (0,0%)
6	0 (0,0%)
7	0 (0,0%)
8	0 (0,0%)
9	0 (0,0%)
Total	2 (100,0%)

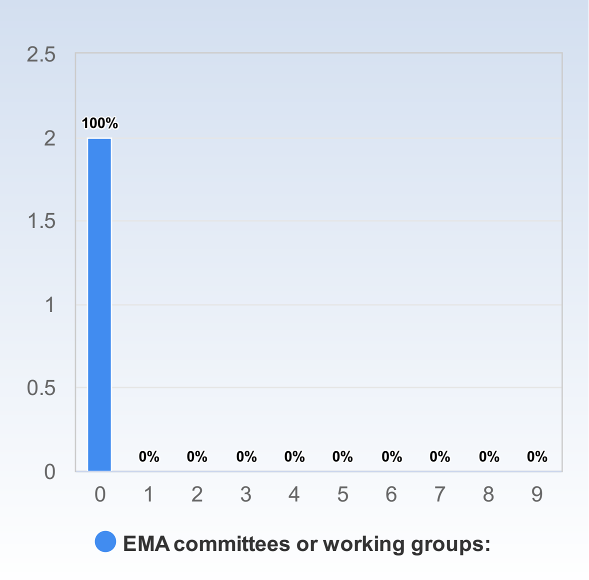

	Mean	Standard Deviation	Coefficient of Variation	Min	Lower Quartile	Median	Upper Quartile	Max
EMA committees or working groups:	0,0	0,0	NaN %	0,0	0,0	0,0	0,0	0,0

OECD working groups:

OECD working groups:	Number of responses
0	1 (20,0%)
1	2 (40,0%)
2	0 (0,0%)
3	1 (20,0%)
4	0 (0,0%)
5	1 (20,0%)
6	0 (0,0%)
7	0 (0,0%)
8	0 (0,0%)
9	0 (0,0%)
Total	5 (100,0%)

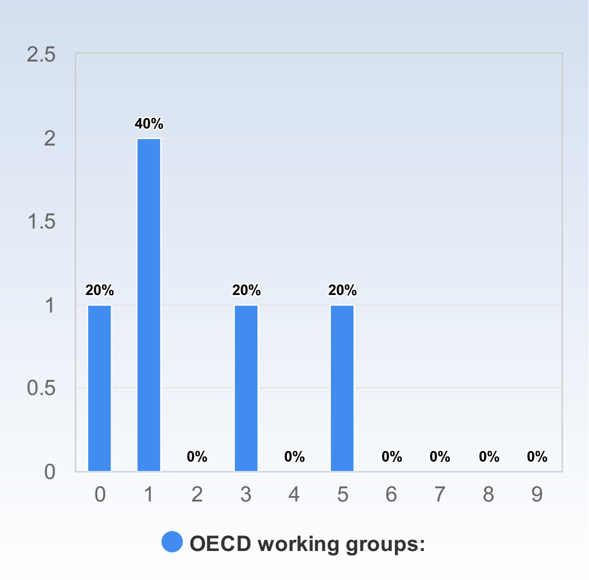

	Mean	Standard Deviation	Coefficient of Variation	Min	Lower Quartile	Median	Upper Quartile	Max
OECD working groups:	2,0	2,0	100,0 %	0,0	1,0	1,0	3,0	5,0

WHO expert groups:

WHO expert groups:	Number of responses
0	2 (66,7%)
1	1 (33,3%)
2	0 (0,0%)
3	0 (0,0%)
4	0 (0,0%)
5	0 (0,0%)
6	0 (0,0%)
7	0 (0,0%)
8	0 (0,0%)
9	0 (0,0%)
Total	3 (100,0%)

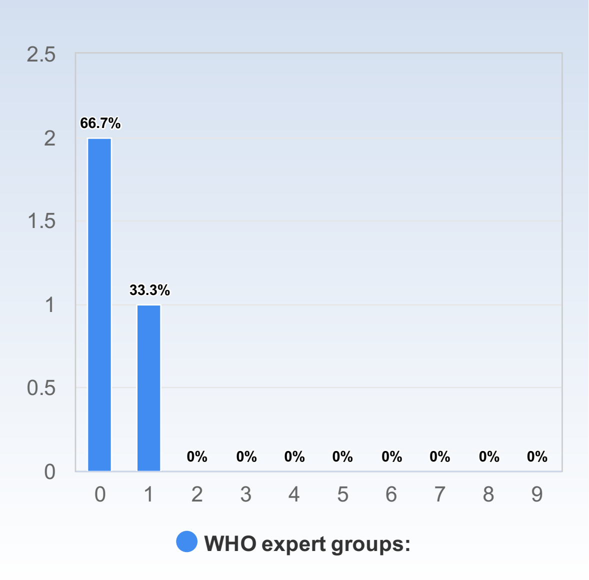

	Mean	Standard Deviation	Coefficient of Variation	Min	Lower Quartile	Median	Upper Quartile	Max
WHO expert groups:	0,3	0,6	173,2 %	0,0	0,0	0,0	0,5	1,0

Other(s) (please comment below)

Other(s) (please comment below)	Number of responses
0	1 (20,0%)
1	2 (40,0%)
2	1 (20,0%)
3	0 (0,0%)
4	0 (0,0%)
5	1 (20,0%)
6	0 (0,0%)
7	0 (0,0%)
8	0 (0,0%)
9	0 (0,0%)
Total	5 (100,0%)

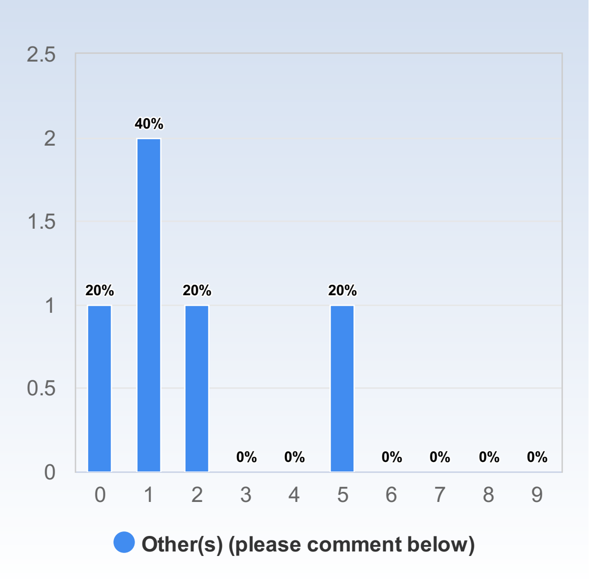

	Mean	Standard Deviation	Coefficient of Variation	Min	Lower Quartile	Median	Upper Quartile	Max
Other(s) (please comment below)	1,8	1,9	106,9 %	0,0	1,0	1,0	2,0	5,0

Comment
Nordic Expert Group
Participation is supported/driven by the participation in the Horizon2020 ONTOX project and includes also the ASPIS cluster and (from May 2022) also PARC.
Others: Commission working groups
Toxicologists not involved in above committees. Some give input to ICH guidelines etc. Several collaborate with other industries on giving input to regulatory improvements
ISO working groups for standardization of methods

Do you expect that the number of personnel involved in international assignments (c.f. previous question) will increase or decrease in the coming 5 years?

Do you expect that the number of personnel involved in international assignments (c.f. previous question) will increase or decrease in the coming 5 years?	Number of responses
Increase	2 (33,3%)
Decrease	2 (33,3%)
No change is expected	2 (33,3%)
Total	6 (100,0%)

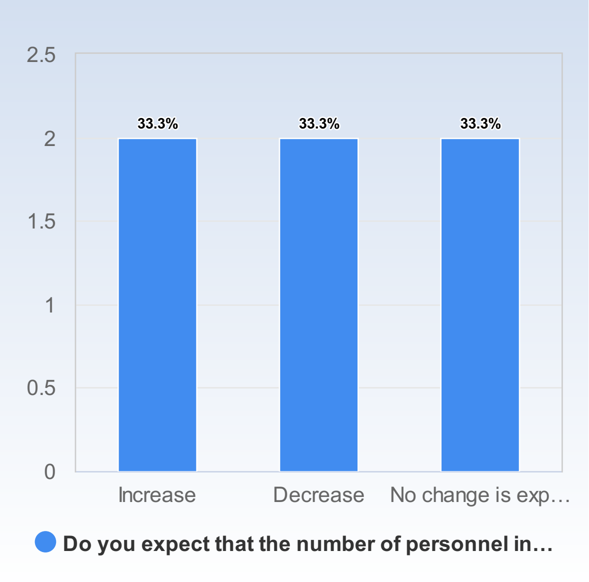

	Mean	Standard Deviation	Coefficient of Variation	Min	Lower Quartile	Median	Upper Quartile	Max
Do you expect that the number of personnel involved in international assignments (c.f. previous question) will increase or decrease in the coming 5 years?	2,0	0,9	44,7 %	1,0	1,5	2,0	2,5	3,0

Please comment.
these activities are diffcult to cover by external funding, and we have to cover our research 120% by external funding.
Small increase.
One will retire, and the cost of participating in ISO working groups are high. Our institute is cutting down costs and focusing on our core tasks, so less and less time is available for participation in such tasks.

Any additional reflections or comments regarding competence provision needs in the area of risk assessment/communication?

Any additional reflections or comments regarding competence provision needs in the area of risk assessment/communication?
There are a lot of EU initiatives aiming at chemical risk assessment without the use of animals. Analyse the needs and expectations of these initiatives and build relevant courses, workshops, webinars, ... to train new risk assessors, managers, communicators for these new tasks and challenges.
No
Use of toxicological discipline could be better integrated part of several lines of education. Could benefit from industrial perspective also.

SI Part D

System generated analyses of the result from the Finnish respondents

Risk analysis competence provision questionnaire for Finland

Respondents: 50
Answer Count: 13
Answer Frequency: 26,00 %

Please indicate your area of affiliation? (mandatory)

Please indicate your area of affiliation? (mandatory)	Number of responses
Research institute	4 (30,8%)
National authority	5 (38,5%)
Regional authority	0 (0,0%)
Hospital practice	0 (0,0%)
Industry/Business	2 (15,4%)
NGO	0 (0,0%)
Consultant	2 (15,4%)
Other	0 (0,0%)
Total	13 (100,0%)

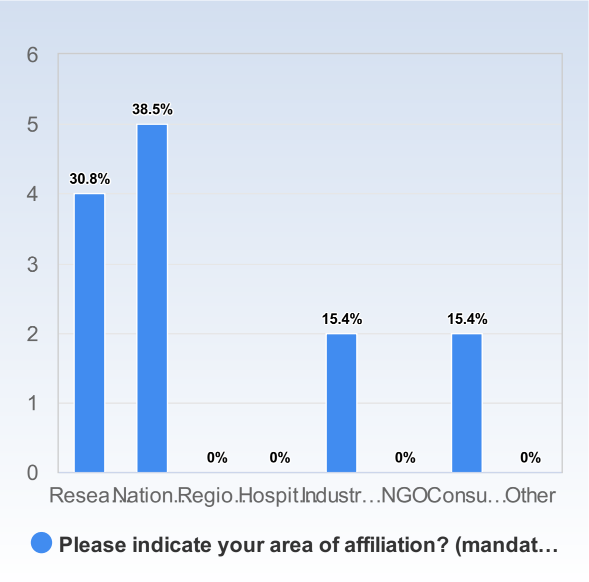

	Mean	Standard Deviation	Coefficient of Variation	Min	Lower Quartile	Median	Upper Quartile	Max
Please indicate your area of affiliation? (mandatory)	4,1	2,6	62,9 %	2,0	2,0	3,0	6,0	9,0

What is the number of personnel in chemical risk assessment/risk communication in your organization?

What is the number of personnel in chemical risk assessment/risk communication in your organization?	Number of responses
0 - 10	8 (66,7%)
11 - 21	2 (16,7%)
22 - 32	0 (0,0%)
33 - 43	0 (0,0%)
44 - 54	0 (0,0%)
55 - 65	2 (16,7%)
66 - 76	0 (0,0%)
77 - 87	0 (0,0%)
88 - 98	0 (0,0%)
99 - 109	0 (0,0%)
Total	12 (100,0%)

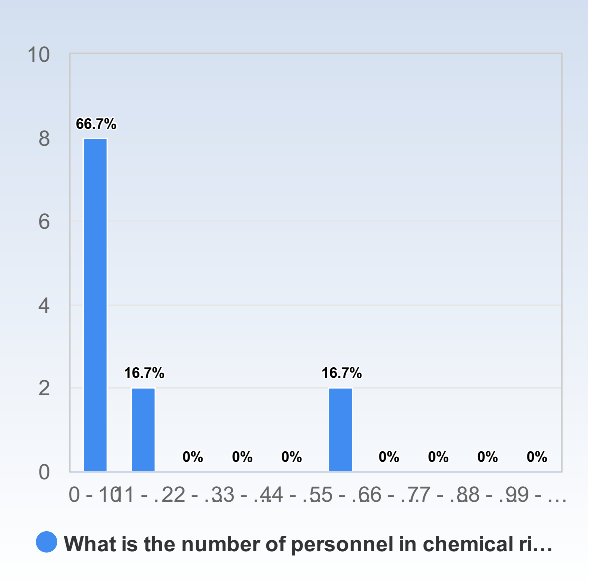

	Mean	Standard Deviation	Coefficient of Variation	Min	Lower Quartile	Median	Upper Quartile	Max
What is the number of personnel in chemical risk assessment/risk communication in your organization?	60,5	163,2	269,9 %	1,0	4,0	10,0	15,0	600,0

What specialization, in chemical risk assessment /communication, do you have in your organization?
Please estimate the number of personnel for each specialization.

Alternative (non-animal) in vitro methods

Alternative (non-animal) in vitro methods	Number of responses
0 - 10	2 (50,0%)
11 - 21	1 (25,0%)
22 - 32	0 (0,0%)
33 - 43	0 (0,0%)
44 - 54	0 (0,0%)
55 - 65	0 (0,0%)
66 - 76	0 (0,0%)
77 - 87	0 (0,0%)
88 - 98	0 (0,0%)
99 - 109	1 (25,0%)
Total	4 (100,0%)

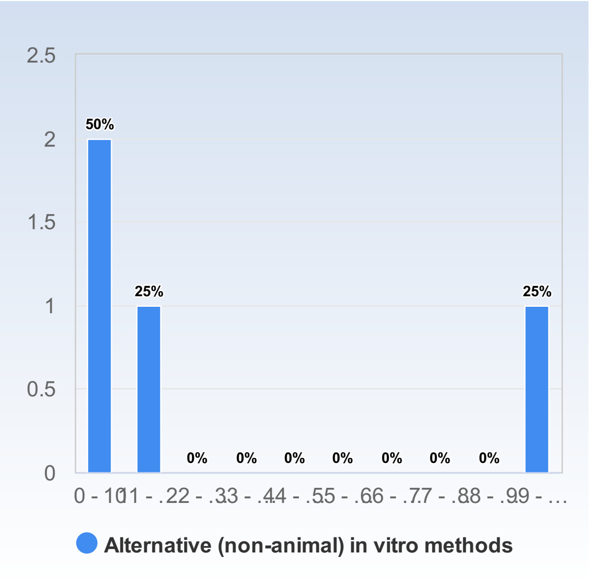

	Mean	Standard Deviation	Coefficient of Variation	Min	Lower Quartile	Median	Upper Quartile	Max
Alternative (non-animal) in vitro methods	29,8	47,1	158,2 %	0,0	4,0	9,5	55,5	100,0

Animal testing

Animal testing	Number of responses
0 - 1	3 (75,0%)
2 - 3	0 (0,0%)
4 - 5	0 (0,0%)
6 - 7	0 (0,0%)
8 - 9	0 (0,0%)
10 - 11	0 (0,0%)
12 - 13	1 (25,0%)
14 - 15	0 (0,0%)
16 - 17	0 (0,0%)
18 - 19	0 (0,0%)
Total	4 (100,0%)

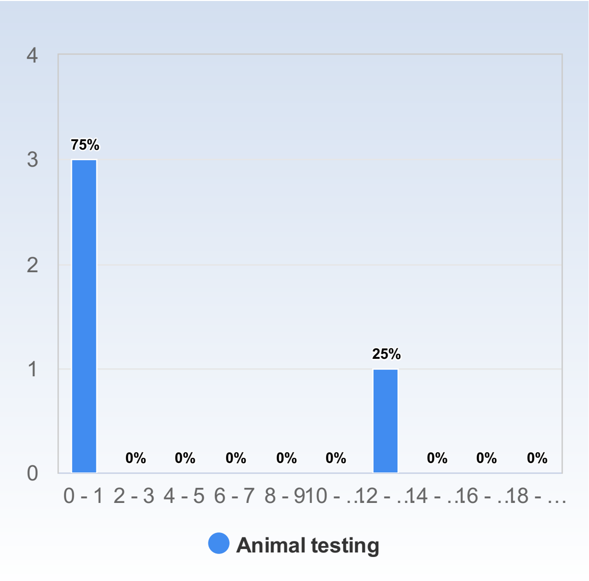

	Mean	Standard Deviation	Coefficient of Variation	Min	Lower Quartile	Median	Upper Quartile	Max
Animal testing	3,3	5,9	180,1 %	0,0	0,0	0,5	6,5	12,0

Bioinformatics

Bioinformatics	Number of responses
0 - 3	3 (75,0%)
4 - 7	0 (0,0%)
8 - 11	0 (0,0%)
12 - 15	0 (0,0%)
16 - 19	0 (0,0%)
20 - 23	0 (0,0%)
24 - 27	0 (0,0%)
28 - 31	1 (25,0%)
32 - 35	0 (0,0%)
36 - 39	0 (0,0%)
Total	4 (100,0%)

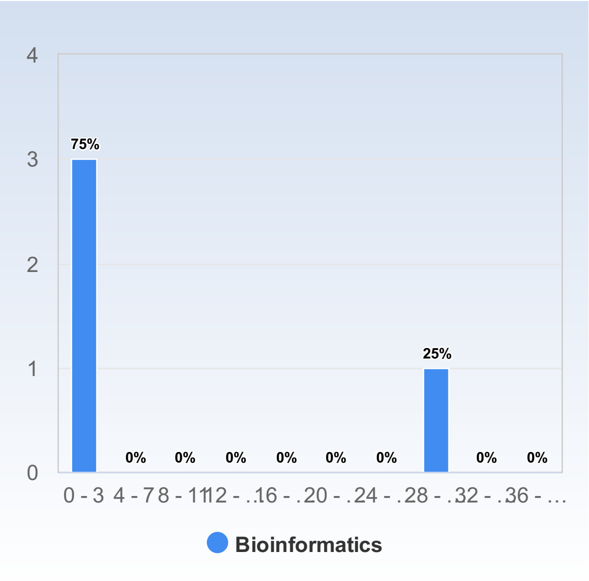

	Mean	Standard Deviation	Coefficient of Variation	Min	Lower Quartile	Median	Upper Quartile	Max
Bioinformatics	8,0	14,7	183,4 %	0,0	0,5	1,0	15,5	30,0

Chemical analysis

Chemical analysis	Number of responses
1 - 6	6 (66,7%)
7 - 12	0 (0,0%)
13 - 18	0 (0,0%)
19 - 24	0 (0,0%)
25 - 30	1 (11,1%)
31 - 36	0 (0,0%)
37 - 42	1 (11,1%)
43 - 48	0 (0,0%)
49 - 54	0 (0,0%)
55 - 60	1 (11,1%)
Total	9 (100,0%)

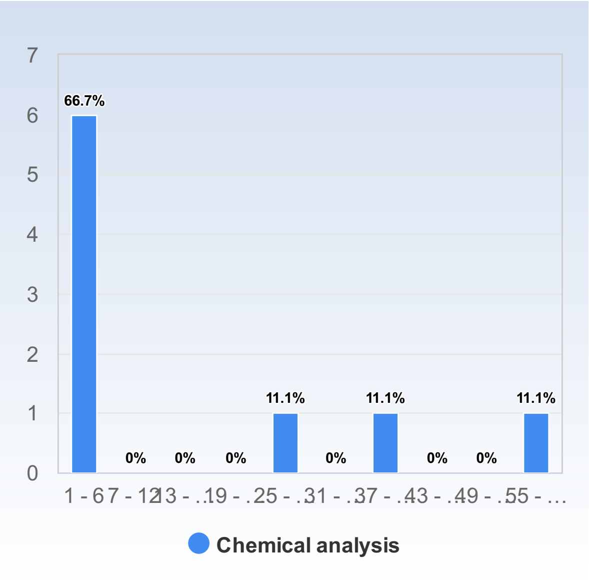

	Mean	Standard Deviation	Coefficient of Variation	Min	Lower Quartile	Median	Upper Quartile	Max
Chemical analysis	16,2	21,2	130,8 %	1,0	2,0	5,0	26,0	60,0

Chemistry/Environmental chemistry

Chemistry/Environmental chemistry	Number of responses
2 - 11	5 (71,4%)
12 - 21	1 (14,3%)
22 - 31	0 (0,0%)
32 - 41	0 (0,0%)
42 - 51	0 (0,0%)
52 - 61	0 (0,0%)
62 - 71	0 (0,0%)
72 - 81	0 (0,0%)
82 - 91	0 (0,0%)
92 - 101	1 (14,3%)
Total	7 (100,0%)

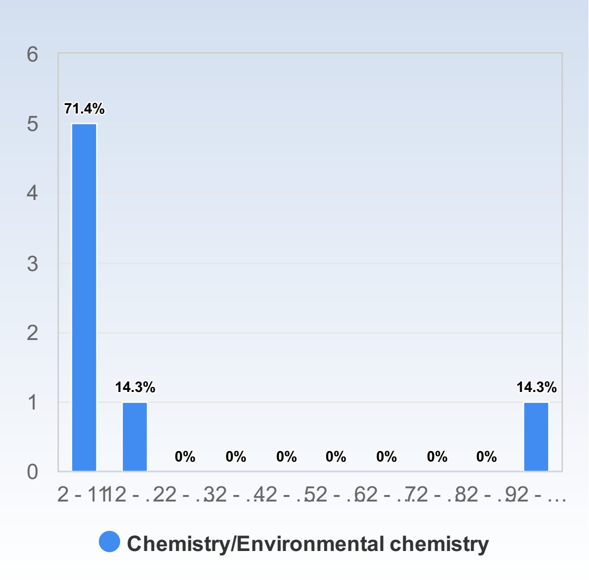

	Mean	Standard Deviation	Coefficient of Variation	Min	Lower Quartile	Median	Upper Quartile	Max
Chemistry/Environmental chemistry	19,7	35,7	181,2 %	2,0	3,0	5,0	12,5	100,0

Ecotoxicology

Ecotoxicology	Number of responses
0 - 1	2 (28,6%)
2 - 3	2 (28,6%)
4 - 5	1 (14,3%)
6 - 7	0 (0,0%)
8 - 9	0 (0,0%)
10 - 11	1 (14,3%)
12 - 13	0 (0,0%)
14 - 15	1 (14,3%)
16 - 17	0 (0,0%)
18 - 19	0 (0,0%)
Total	7 (100,0%)

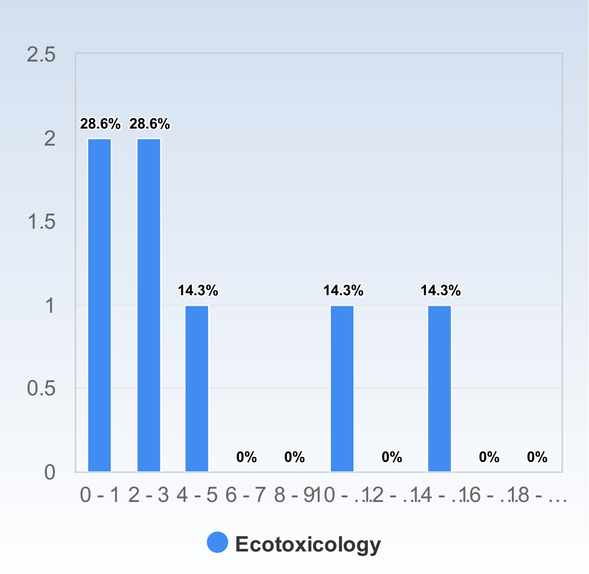

	Mean	Standard Deviation	Coefficient of Variation	Min	Lower Quartile	Median	Upper Quartile	Max
Ecotoxicology	4,7	5,7	120,4 %	0,0	1,0	2,0	7,0	15,0

Epidemiology

Epidemiology	Number of responses
0	2 (33,3%)
1	4 (66,7%)
2	0 (0,0%)
3	0 (0,0%)
4	0 (0,0%)
5	0 (0,0%)
6	0 (0,0%)
7	0 (0,0%)
8	0 (0,0%)
9	0 (0,0%)
Total	6 (100,0%)

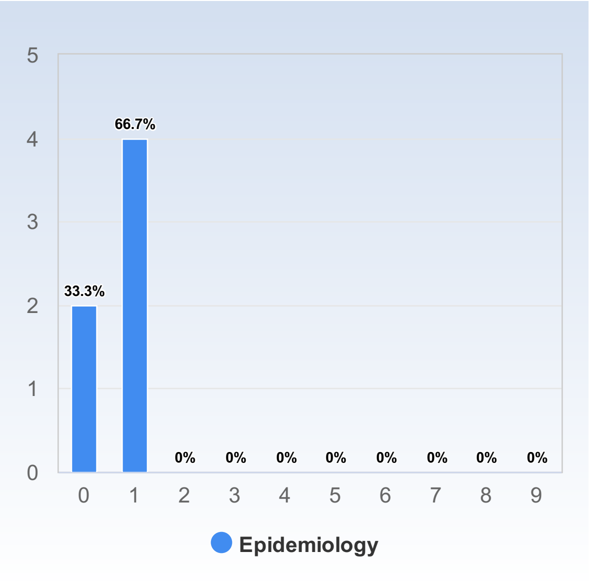

	Mean	Standard Deviation	Coefficient of Variation	Min	Lower Quartile	Median	Upper Quartile	Max
Epidemiology	0,7	0,5	77,5 %	0,0	0,5	1,0	1,0	1,0

Exposure assessment

Exposure assessment	Number of responses
3 - 4	2 (25,0%)
5 - 6	2 (25,0%)
7 - 8	0 (0,0%)
9 - 10	2 (25,0%)
11 - 12	0 (0,0%)
13 - 14	0 (0,0%)
15 - 16	1 (12,5%)
17 - 18	0 (0,0%)
19 - 20	0 (0,0%)
21 - 22	1 (12,5%)
Total	8 (100,0%)

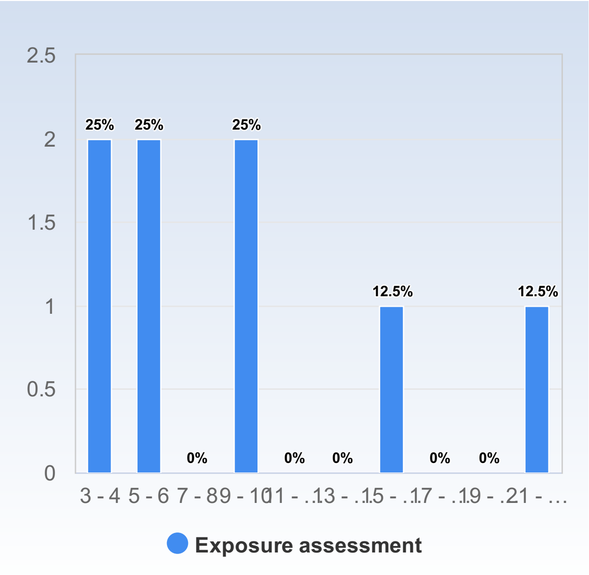

	Mean	Standard Deviation	Coefficient of Variation	Min	Lower Quartile	Median	Upper Quartile	Max
Exposure assessment	9,1	6,2	67,7 %	3,0	4,5	7,5	12,5	21,0

QSAR and read-across

QSAR and read-across	Number of responses
0 - 1	4 (66,7%)
2 - 3	1 (16,7%)
4 - 5	0 (0,0%)
6 - 7	0 (0,0%)
8 - 9	0 (0,0%)
10 - 11	1 (16,7%)
12 - 13	0 (0,0%)
14 - 15	0 (0,0%)
16 - 17	0 (0,0%)
18 - 19	0 (0,0%)
Total	6 (100,0%)

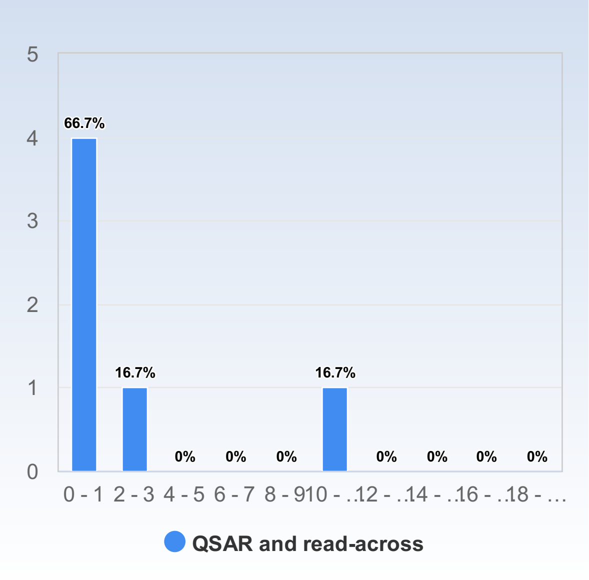

	Mean	Standard Deviation	Coefficient of Variation	Min	Lower Quartile	Median	Upper Quartile	Max
QSAR and read-across	2,3	4,3	185,2 %	0,0	0,0	0,5	1,5	11,0

Risk assessment

Risk assessment	Number of responses
0 - 5	7 (63,6%)
6 - 11	1 (9,1%)
12 - 17	1 (9,1%)
18 - 23	0 (0,0%)
24 - 29	0 (0,0%)
30 - 35	0 (0,0%)
36 - 41	0 (0,0%)
42 - 47	1 (9,1%)
48 - 53	1 (9,1%)
54 - 59	0 (0,0%)
Total	11 (100,0%)

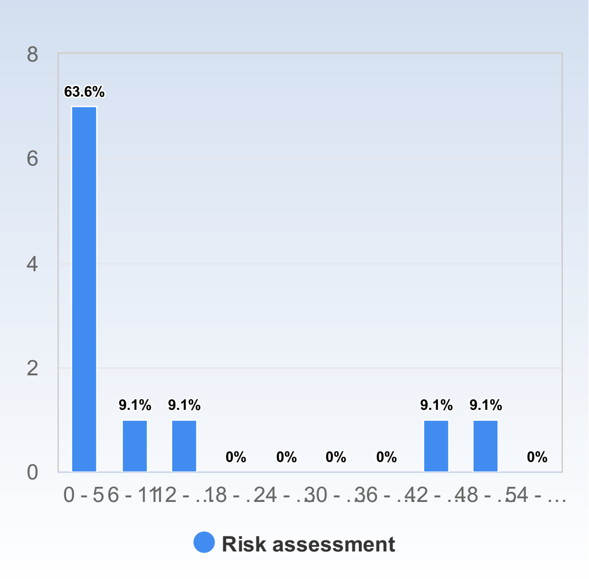

	Mean	Standard Deviation	Coefficient of Variation	Min	Lower Quartile	Median	Upper Quartile	Max
Risk assessment	11,4	18,4	161,8 %	0,0	1,0	2,0	10,5	50,0

Risk communication

Risk communication	Number of responses
0 - 3	5 (55,6%)
4 - 7	1 (11,1%)
8 - 11	0 (0,0%)
12 - 15	1 (11,1%)
16 - 19	0 (0,0%)
20 - 23	1 (11,1%)
24 - 27	0 (0,0%)
28 - 31	0 (0,0%)
32 - 35	0 (0,0%)
36 - 39	1 (11,1%)
Total	9 (100,0%)

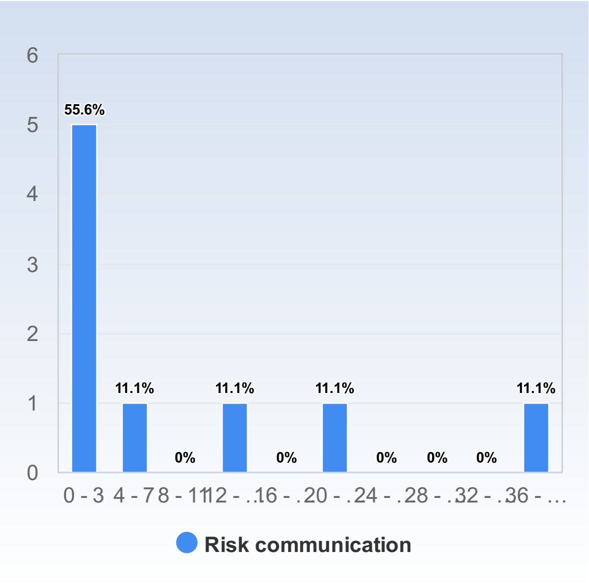

	Mean	Standard Deviation	Coefficient of Variation	Min	Lower Quartile	Median	Upper Quartile	Max
Risk communication	9,6	12,1	126,1 %	1,0	2,0	2,0	15,0	36,0

Risk management

Risk management	Number of responses
0 - 5	3 (42,9%)
6 - 11	1 (14,3%)
12 - 17	1 (14,3%)
18 - 23	0 (0,0%)
24 - 29	0 (0,0%)
30 - 35	0 (0,0%)
36 - 41	1 (14,3%)
42 - 47	0 (0,0%)
48 - 53	1 (14,3%)
54 - 59	0 (0,0%)
Total	7 (100,0%)

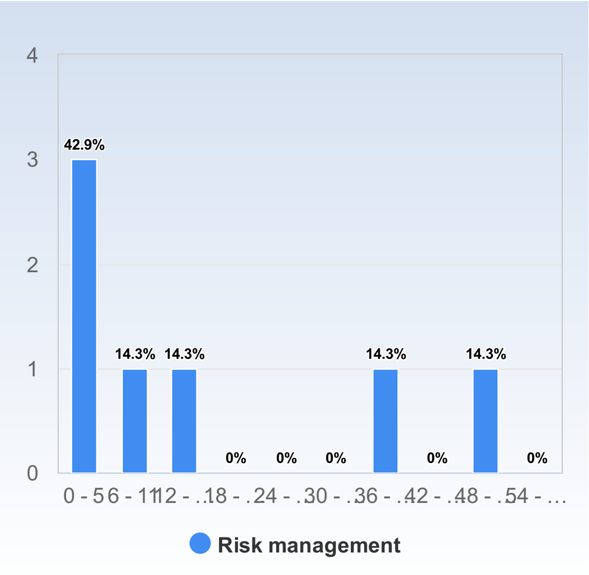

	Mean	Standard Deviation	Coefficient of Variation	Min	Lower Quartile	Median	Upper Quartile	Max
Risk management	16,9	19,4	115,1 %	0,0	4,5	6,0	26,5	50,0

Statistics

Statistics	Number of responses
0 - 1	2 (40,0%)
2 - 3	0 (0,0%)
4 - 5	2 (40,0%)
6 - 7	0 (0,0%)
8 - 9	0 (0,0%)
10 - 11	0 (0,0%)
12 - 13	0 (0,0%)
14 - 15	1 (20,0%)
16 - 17	0 (0,0%)
18 - 19	0 (0,0%)
Total	5 (100,0%)

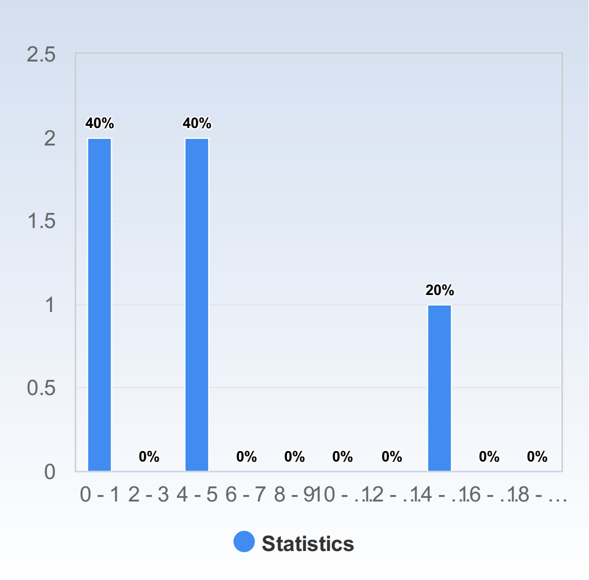

	Mean	Standard Deviation	Coefficient of Variation	Min	Lower Quartile	Median	Upper Quartile	Max
Statistics	5,0	6,0	119,2 %	0,0	1,0	4,0	5,0	15,0

Systematic literature reviews

Systematic literature reviews	Number of responses
0 - 1	2 (33,3%)
2 - 3	1 (16,7%)
4 - 5	0 (0,0%)
6 - 7	1 (16,7%)
8 - 9	0 (0,0%)
10 - 11	2 (33,3%)
12 - 13	0 (0,0%)
14 - 15	0 (0,0%)
16 - 17	0 (0,0%)
18 - 19	0 (0,0%)
Total	6 (100,0%)

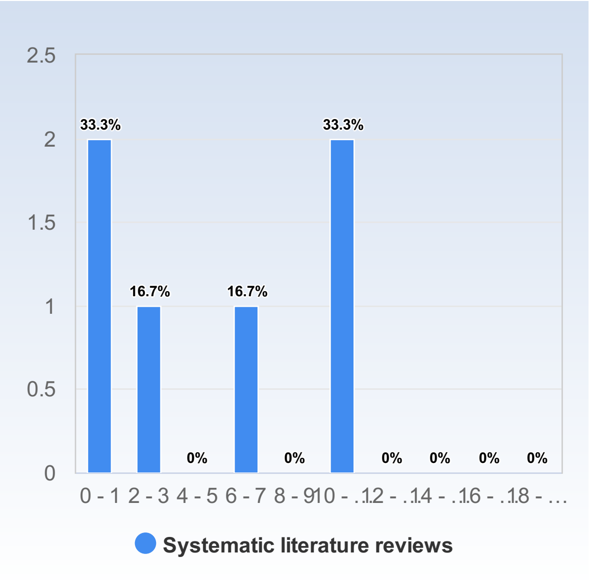

	Mean	Standard Deviation	Coefficient of Variation	Min	Lower Quartile	Median	Upper Quartile	Max
Systematic literature reviews	5,0	4,7	94,7 %	0,0	1,5	4,0	8,0	11,0

Toxicology

Toxicology	Number of responses
0 - 1	5 (41,7%)
2 - 3	3 (25,0%)
4 - 5	1 (8,3%)
6 - 7	0 (0,0%)
8 - 9	0 (0,0%)
10 - 11	1 (8,3%)
12 - 13	0 (0,0%)
14 - 15	2 (16,7%)
16 - 17	0 (0,0%)
18 - 19	0 (0,0%)
Total	12 (100,0%)

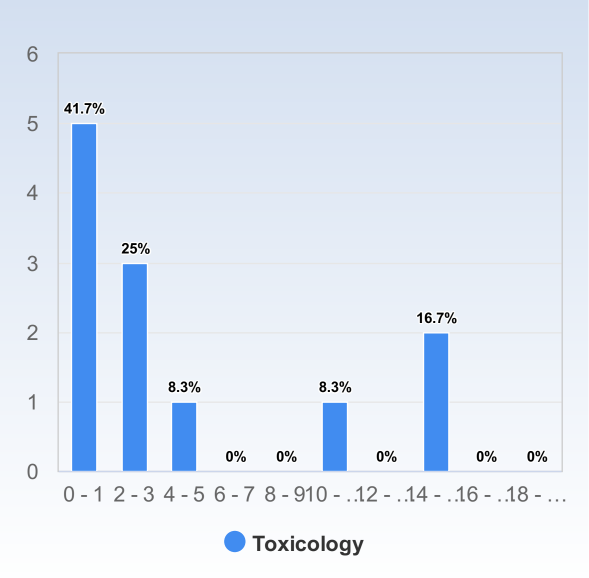

	Mean	Standard Deviation	Coefficient of Variation	Min	Lower Quartile	Median	Upper Quartile	Max
Toxicology	4,6	5,4	116,7 %	0,0	1,0	2,0	7,5	15,0

Other (please comment below)

Other (please comment below)	Number of responses
0	0 (0,0%)
1	0 (0,0%)
2	0 (0,0%)
3	0 (0,0%)
4	1 (100,0%)
5	0 (0,0%)
6	0 (0,0%)
7	0 (0,0%)
8	0 (0,0%)
9	0 (0,0%)
Total	1 (100,0%)

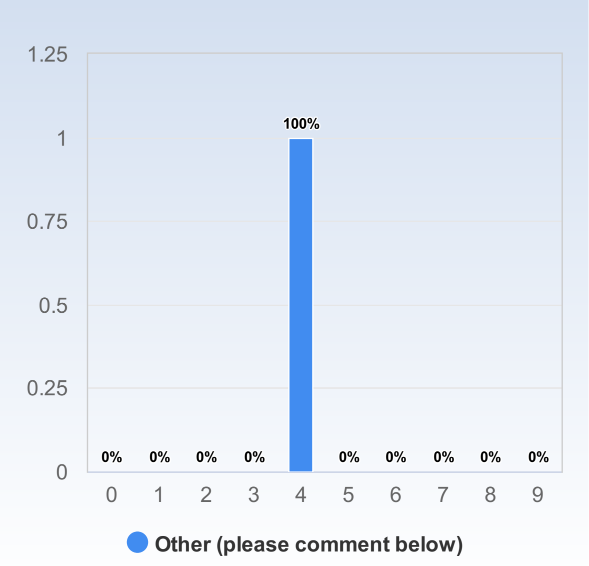

	Mean	Standard Deviation	Coefficient of Variation	Min	Lower Quartile	Median	Upper Quartile	Max
Other (please comment below)	4,0	0,0	NaN %	4,0	4,0	4,0	4,0	4,0

Comment
One person part-time
Very difficult the address the exact numbers of the people working in the specific areas in a large research organisation. Please take these numbers as indicative.
Risk assessment includes exposure assessment.<br>Number of researchers involved in the field is 4, but chemical risk assessment mainly by 2 persons. One out of 2 conducts epidemiological assessments, 2 other persons are statisticians. Chemical risk assessment needs knowledge of toxicology. All the personnel is involved with communication.
We would like to point out that same persons work in different specializations. Also, most of our staff carry out work related to risk assessment only part-time. However, the risk assessment is linked to everybody's work more or less, and that is why we named all our researchers in question 2.<br>Our main task is chemical analysis, which takes about 3 person years in total (involved 5 persons). Exposure and risk assessment + communication and toxicology takes nowadays only 1 – 1.5 person year in total.
Four persons have some knowledge on various disciplines of toxicology but are not toxicologists by education.
Actual work in some areas asked in the Authority is mainy other, but skills to do is available. Risk assessmet in based on comparison to TDI or similar definitons made by EFSA or other specific institutes
Finnish Poison Information Centre, we have a team of 15 people with pharmaceutical and medical background. We focus on clinical toxicology with expertise in risk assessment, management and medical toxicology.
26 persons working in our chemistry lab (all marked to "chemical analyses" are involved in the analysing either air, surface or biomonitoring samples from the workplaces. These include also laboratory technicians. Exposure assessors (26) include industrial hygienists providing industrial hygienic services to Finnish workplaces and 4 industrial hygienist who are mainly involved in research projects (instead of expert services provided by our institute). Toxicologist performing toxicology research in lab have been divided between in vitro/in vivo according to PM distribution/year (however, there are 4 toxicologist able to run animal studies). Additionally, there are toxicologists/chemists (7) who are more involved in risk assessment activities; these include regulatory risk assessment, limit value setting, risk assessment activities in research projects, toxicological support to our occupational health clinics, biomonitoring activities. These have been divided between "toxicology" and "Risk Assessment" but this is rather arbitrary division.
Same person deals with several areas of specialization. We have excluded chemistry because most of our personnel are chemists, as we are a chemical company.

How many of the personnel in your organization, with the chemical risk assessment /communication tasks you presented in the previous question, holds a:

Bachelor degree:

Bachelor degree:	Number of responses
0 - 4	1 (25,0%)
5 - 9	1 (25,0%)
10 - 14	0 (0,0%)
15 - 19	0 (0,0%)
20 - 24	1 (25,0%)
25 - 29	0 (0,0%)
30 - 34	0 (0,0%)
35 - 39	0 (0,0%)
40 - 44	1 (25,0%)
45 - 49	0 (0,0%)
Total	4 (100,0%)

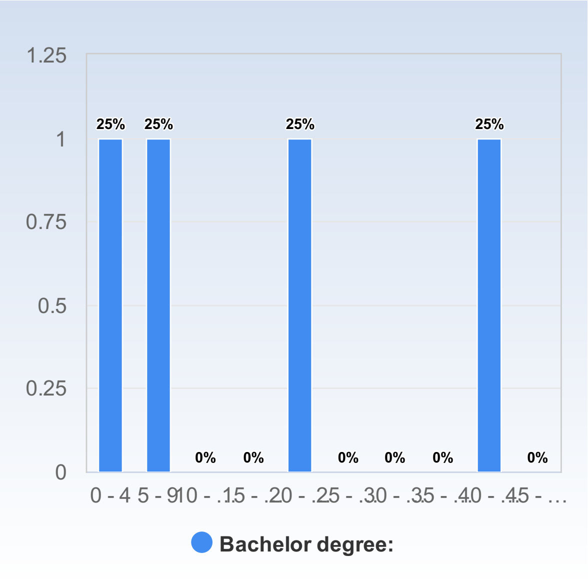

	Mean	Standard Deviation	Coefficient of Variation	Min	Lower Quartile	Median	Upper Quartile	Max
Bachelor degree:	17,3	17,7	102,8 %	0,0	3,5	14,5	31,0	40,0

Master degree:

Master degree:	Number of responses
0 - 4	7 (58,3%)
5 - 9	2 (16,7%)
10 - 14	0 (0,0%)
15 - 19	0 (0,0%)
20 - 24	2 (16,7%)
25 - 29	0 (0,0%)
30 - 34	0 (0,0%)
35 - 39	0 (0,0%)
40 - 44	1 (8,3%)
45 - 49	0 (0,0%)
Total	12 (100,0%)

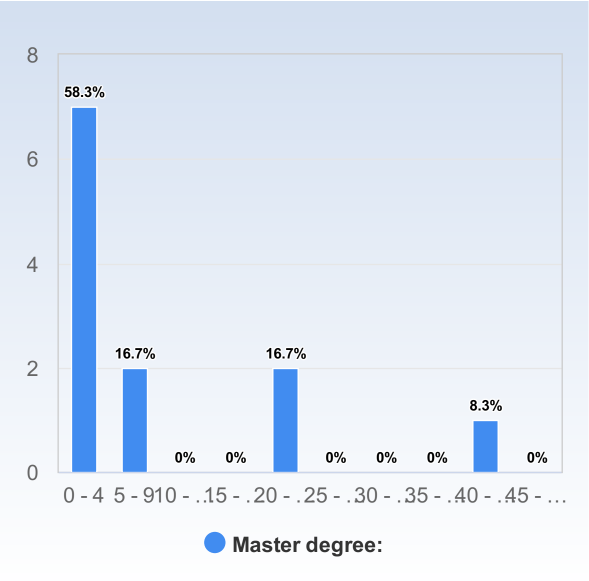

	Mean	Standard Deviation	Coefficient of Variation	Min	Lower Quartile	Median	Upper Quartile	Max
Master degree:	9,2	12,6	137,9 %	1,0	1,5	3,0	14,0	42,0

Doctoral degree (PhDs, MDs, Dtech (TkT, D.I.T), etc):

Doctoral degree (PhDs, MDs, Dtech (TkT, D.I.T), etc):	Number of responses
1 - 2	2 (18,2%)
3 - 4	3 (27,3%)
5 - 6	2 (18,2%)
7 - 8	0 (0,0%)
9 - 10	1 (9,1%)
11 - 12	1 (9,1%)
13 - 14	1 (9,1%)
15 - 16	0 (0,0%)
17 - 18	0 (0,0%)
19 - 20	1 (9,1%)
Total	11 (100,0%)

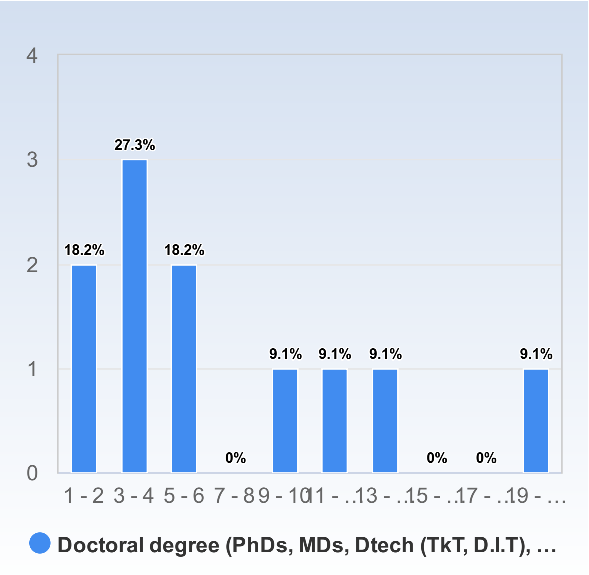

	Mean	Standard Deviation	Coefficient of Variation	Min	Lower Quartile	Median	Upper Quartile	Max
Doctoral degree (PhDs, MDs, Dtech (TkT, D.I.T), etc):	7,1	5,7	79,9 %	1,0	3,0	5,0	11,0	19,0

How many of your personnel are ERTs (European Registered Toxicologist)?

How many of your personnel are ERTs (European Registered Toxicologist)?	Number of responses
0 - 10	13 (100,0%)
11 - 21	0 (0,0%)
22 - 32	0 (0,0%)
33 - 43	0 (0,0%)
44 - 54	0 (0,0%)
55 - 65	0 (0,0%)
66 - 76	0 (0,0%)
77 - 87	0 (0,0%)
88 - 98	0 (0,0%)
99 - 109	0 (0,0%)
Total	13 (100,0%)

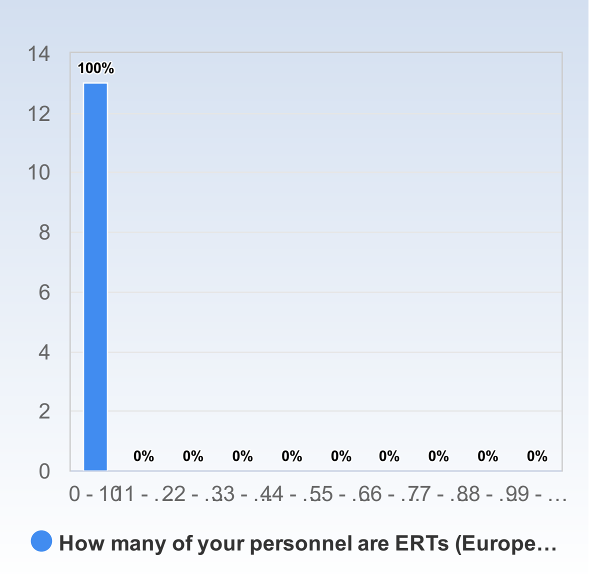

	Mean	Standard Deviation	Coefficient of Variation	Min	Lower Quartile	Median	Upper Quartile	Max
How many of your personnel are ERTs (European Registered Toxicologist)?	1,0	1,8	178,0 %	0,0	0,0	0,0	1,0	6,0

What is the present age profile of the risk analysis personnel in your organization?

< 40 years of age:

< 40 years of age:	Number of responses
0 - 2	5 (50,0%)
3 - 5	1 (10,0%)
6 - 8	1 (10,0%)
9 - 11	1 (10,0%)
12 - 14	1 (10,0%)
15 - 17	0 (0,0%)
18 - 20	0 (0,0%)
21 - 23	1 (10,0%)
24 - 26	0 (0,0%)
27 - 29	0 (0,0%)
Total	10 (100,0%)

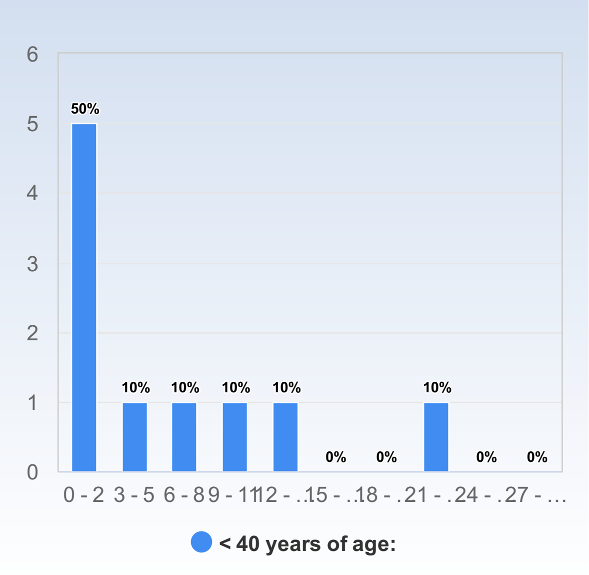

	Mean	Standard Deviation	Coefficient of Variation	Min	Lower Quartile	Median	Upper Quartile	Max
< 40 years of age:	6,2	7,1	115,0 %	1,0	1,5	2,5	8,5	23,0

40-50 years of age:

40-50 years of age:	Number of responses
0 - 2	3 (27,3%)
3 - 5	4 (36,4%)
6 - 8	1 (9,1%)
9 - 11	0 (0,0%)
12 - 14	0 (0,0%)
15 - 17	0 (0,0%)
18 - 20	1 (9,1%)
21 - 23	1 (9,1%)
24 - 26	1 (9,1%)
27 - 29	0 (0,0%)
Total	11 (100,0%)

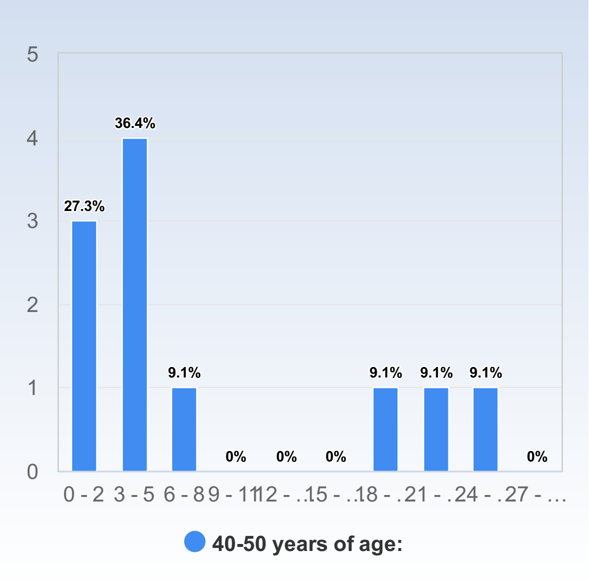

	Mean	Standard Deviation	Coefficient of Variation	Min	Lower Quartile	Median	Upper Quartile	Max
40-50 years of age:	8,5	8,9	105,8 %	1,0	2,5	4,0	13,5	25,0

50-60 years of age:

50-60 years of age:	Number of responses
2 - 3	6 (60,0%)
4 - 5	1 (10,0%)
6 - 7	0 (0,0%)
8 - 9	1 (10,0%)
10 - 11	0 (0,0%)
12 - 13	1 (10,0%)
14 - 15	0 (0,0%)
16 - 17	0 (0,0%)
18 - 19	0 (0,0%)
20 - 21	1 (10,0%)
Total	10 (100,0%)

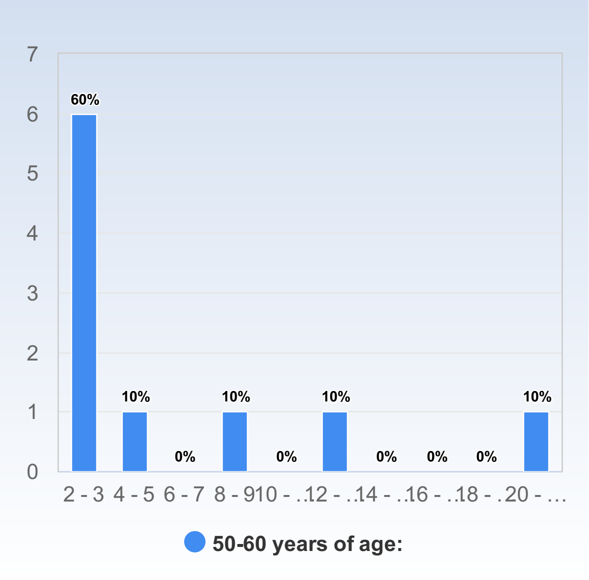

	Mean	Standard Deviation	Coefficient of Variation	Min	Lower Quartile	Median	Upper Quartile	Max
50-60 years of age:	5,9	6,1	103,4 %	2,0	2,0	3,0	6,0	20,0

60-65 years of age:

60-65 years of age:	Number of responses
0	1 (16,7%)
1	1 (16,7%)
2	2 (33,3%)
3	0 (0,0%)
4	0 (0,0%)
5	0 (0,0%)
6	1 (16,7%)
7	0 (0,0%)
8	0 (0,0%)
9	1 (16,7%)
Total	6 (100,0%)

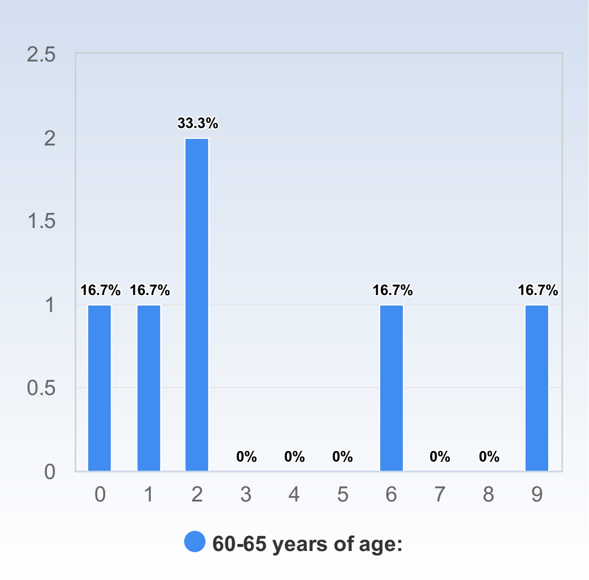

	Mean	Standard Deviation	Coefficient of Variation	Min	Lower Quartile	Median	Upper Quartile	Max
60-65 years of age:	3,3	3,4	103,3 %	0,0	1,5	2,0	4,0	9,0

> 65 years of age:

> 65 years of age:	Number of responses
0	1 (50,0%)
1	1 (50,0%)
2	0 (0,0%)
3	0 (0,0%)
4	0 (0,0%)
5	0 (0,0%)
6	0 (0,0%)
7	0 (0,0%)
8	0 (0,0%)
9	0 (0,0%)
Total	2 (100,0%)

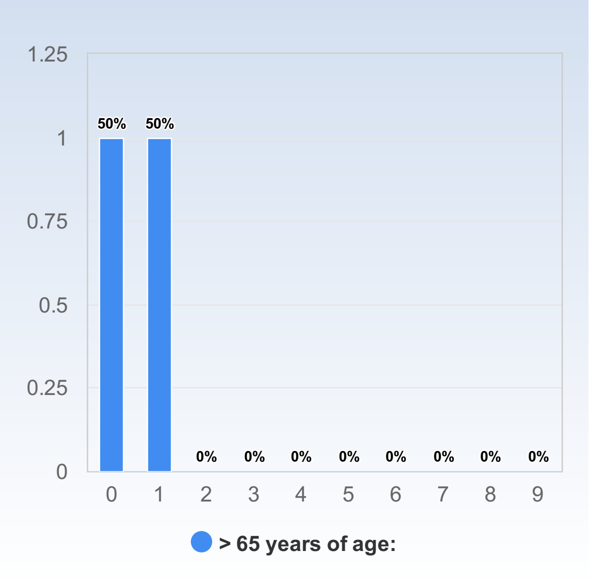

	Mean	Standard Deviation	Coefficient of Variation	Min	Lower Quartile	Median	Upper Quartile	Max
> 65 years of age:	0,5	0,7	141,4 %	0,0	0,5	0,5	0,5	1,0

Is there a need for hiring of replacement /expanding the number of chemical risk assessment/communication personnel over the next 5-10 years in your organization, e.g. due to retirement or change in duties/deliverables within your organisation?

Is there a need for hiring of replacement /expanding the number of chemical risk assessment/communication personnel over the next 5-10 years in your organization, e.g. due to retirement or change in duties/deliverables within your organisation?	Number of responses
YES	11 (84,6%)
NO	2 (15,4%)
Total	13 (100,0%)

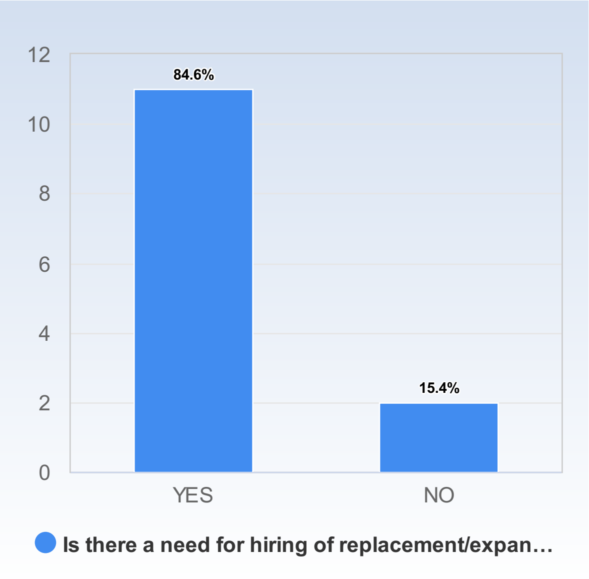

	Mean	Standard Deviation	Coefficient of Variation	Min	Lower Quartile	Median	Upper Quartile	Max
Is there a need for hiring of replacement/expanding the number of chemical risk assessment/communication personnel over the next 5-10 years in your organization, e.g. due to retirement or change in duties/deliverables within your organisation?	1,2	0,4	32,5 %	1,0	1,0	1,0	1,0	2,0

Please comment
regulative experience
In principle, we would need continuation in analytic epidemiology on cancer risks caused by occupational and environmental hazards, but our institute has decided that it will not be within a key function for its activities
There is need due the new directives from EU and retirement.
No retirements in the nearest future, and the turn over of the permanent personnel is low. Project researchers will be recruited when needed.
A few persons (toxicologists) have already retired, and no one has been hired to replace them
Certain number expertise needed, due to retirement new experts needed.
Currently not foreseen.
realistic need is, but it may me left out in authoritys strategic planning
ERT will retire within next 5 years. Some other retirements within next few years.
Need to hire industrial hygienists able to measure and assess exposure at workplaces; we have currently two open positions. In addition, we have recently hired one new toxicologists and we might also need one additional (temporary contract) researcher in our genotoxicology laboratory this year or next year.
due to retirement 3, 2-5 additional due to increasing workload ->if financially possible

Which needs do you forsee you will have recruiting relevant personnel for the coming 4 - 7 years? Please address your needs for personnel with general toxicology and related science educations, versus highly specified education.

Which needs do you forsee you will have recruiting relevant personnel for the coming 4 - 7 years? Please address your needs for personnel with general toxicology and related science educations, versus highly specified education.
general toxicology, regulative toxicology, biocidal substances
Lack of funding and other resources will be the main problem. Another problem is that there will be lack of well capable specialists in the collaborating institutes due to the same problem in their resource allocation.
1. New regulations from EU i.e. Do Not Significant Harm principle and Single Use Plastic Directive<br>2. Retirement<br>3. New Biomaterials development<br>4. Recirculation and utilization of industrial side streams
We have a need for toxicology experts, but it is unlikely we will have the possibility to recruit permanent personnel.
We would need to hire person/persons with environmental toxicology and risk assessment educations
Need to assure to have in minimum one general toxicologist in the organisation.
There is likely a need to hire toxicologists, in particular general toxicologists. Risk assessors or exposure assessors are more challenging to hire, as rarely a general toxicologist has also experience in risk assessment or exposure assessment or e.g. a (food) chemist has experience in exposure assessment.
Basic training on understanding toxicity is urgently needed. This provides route to specific education
Personnel with general toxicology educations, with chemistry and pharmacy educations.
Finnish Poison Information Center has a continuous need for BScs in Pharmacy and MDs with special expertise in acute medicine, pharmacology and toxicology.
See the previous question. We need industrial hygienists and since it requires special expertise and education possibilities for it are limited in Finland we have had challenges in finding suitable candidates. In addition, in latest toxicology recruitment we had challenges to find suitable candidates; in two last recruitments the other one who was hired came outside Nordic countries (we were specifically searching some existing experience in genotoxicology and laboratory work). For risk assessment positions we have had open lately, it has been also really difficult to find toxicologists who would have had earlier experience/or any real knowledge of toxicological risk assessment or occupational toxicology. Since occupational toxicology is rather important field if we consider e.g. regulatory toxicology (e.g. under REACH) there is a real need to increase knowledge of this field. This should start already from the basic toxicology education.
General toxicology and risk assessment, environmental toxicology.
MSc in ecotoxicology/environmental chemistry, MSc in chemistry; MSc in toxicology; general toxicology

Please indicate your experience related to how easy it is to recruit competencies in chemical risk assessment/ communication you want to have /hire. Please use a number between 1 (easy to recruit) and 6 (difficult to recruit).

Please indicate your experience related to how easy it is to recruit competencies in chemical risk assessment/ communication you want to have /hire. Please use a number between 1 (easy to recruit) and 6 (difficult to recruit).	Number of responses
1. Easy to recruit	0 (0,0%)
2.	0 (0,0%)
3.	1 (7,7%)
4.	1 (7,7%)
5.	10 (76,9%)
6. Difficult to recruit	1 (7,7%)
Total	13 (100,0%)

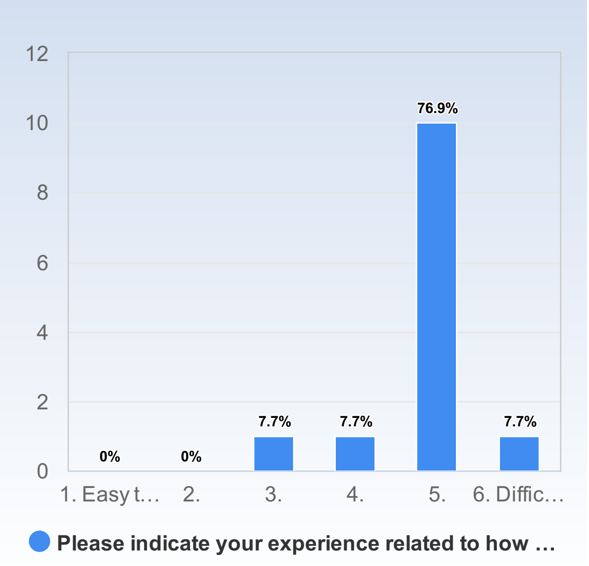

	Mean	Standard Deviation	Coefficient of Variation	Min	Lower Quartile	Median	Upper Quartile	Max
Please indicate your experience related to how easy it is to recruit competencies in chemical risk assessment/ communication you want to have /hire. Please use a number between 1 (easy to recruit) and 6 (difficult to recruit) .	4,8	0,7	14,2 %	3,0	5,0	5,0	5,0	6,0

# Indicate if academia delivers sufficient number of candidates to fulfill your needs (master and/or PhD level)?

Indicate if academia delivers sufficient number of candidates to fulfill your needs (master and/or PhD level)?
Number of candidates may be sufficient, but the quality varies too much
At least within the PhD level, there is not succifient number of trainees on analytic epidemiology on chemical hazards
There is no relevant education for new biomaterials risk evaluation thus the job requires additional training
Risk assessment science is not taught in any Finnish university, neither are toxicologists easily available. Therefore the candidates are hired according to their other competences.
Academia delivers sufficient amount of toxicologists with master degrees every year, but usually they don’t have the required experience for risk assessment.
Yes and no. It is not only getting newly graduated experts, but also have work experience. But in general: more academic MS / PhDs are needed to provide sufficient pool of candates.
At the moment not. MSc-level would suffice in order for these people to be available on the work markets faster.
In Helsinki area there enough training in chemistry, biology, biochemistry or pharmacy.
As regards chemistry/pharmacy educations, yes.
We have identified problems in recruiting medical doctors with interest in clinical toxicology. While there are several subspecialty training programs available for medical doctors, a subspecialty training in clinical toxicology does not exist. In Finland, there is neither a professorship in clinical toxicology.
There might be a need for more experts especially in the field of industrial hygiene but possibly also in toxicology. In the case of toxicology, in toxicology education even more emphasis could be given for risk assessment skills and the students should be made more familiar also with the special field of occupational toxicology.
No, especially regulatory toxicology/ecotoxicology.
To our knowledge, toxicology can only be studied after a BSc degree in chemistry / pharmacy / medicine / biochemistry or equivalent; a BSc degree in toxicology is not available.<br>The MSc in Toxicology is only offered as an international study program, in which there may not be enough experts who also speak Finnish fluently, which is necessary in order to work for the Finnish national authority.<br>Recruitment challenges describe skills needeed and job assignments in a way that reaches all potential applicants.<br>Tukes may not be a sufficiently well-known employer<br>ECHA competes for staff.

Do you find it necessary to train your new personnel in the areas of chemical risk assessment/communication due to limited/poor knowledge from academia or previous affiliation (s)/work experience?

Do you find it necessary to train your new personnel in the areas of chemical risk assessment/communication due to limited/poor knowledge from academia or previous affiliation(s) /work experience?	Number of responses
YES	12 (92,3%)
NO	1 (7,7%)
Total	13 (100,0%)

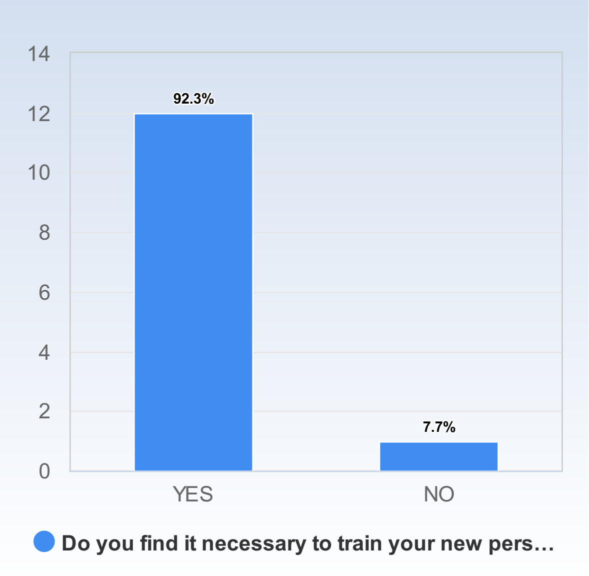

	Mean	Standard Deviation	Coefficient of Variation	Min	Lower Quartile	Median	Upper Quartile	Max
Do you find it necessary to train your new personnel in the areas of chemical risk assessment/communication due to limited/poor knowledge from academia or previous affiliation(s)/work experience?	1,1	0,3	25,8 %	1,0	1,0	1,0	1,0	2,0

If YES, please comment on how you train the personnel.
internal training and external courses
Not possible for the moment, because of lack of research resources and prioritization
Hands on work i.e. research proposal preparation, occupational health regulations, monthly QMS announcements
Mostly on the job learning.<br>Conferences, BTSF and other courses, and previous materials are utilized.
We don’t have specific training resources, our way is to participate in relevant courses and learn from older, more experienced colleagues
By sending them to external training programmes and cources - even basic level training sometimes needed.
Through internal and external training courses, and external webinars.
internal and external seminars/trainings or presentations are useful.
Regulatory guidelines, internal SOPs, supervisor training, training courses.
A long self-tailored introduction/familiarization period of ~6 months
In the fields of industrial hygiene and occupational toxicology (and regulatory risk assessment related to e.g. limit value setting) it is not possible to find personnel who would have those skills when entering the institute. Therefore, we are currently considering if it would be possible to set up "trainee" positions in our institute to fulfill our own future needs for skilled personnel but also to increase the expertise in this fields in Finland in general. This is however only very early phases of the planning.
Experienced colleagues are training new personnel. BTSF trainings & risk assessment trainings organized by Wageningen University etc...<br>Work shop organized by Karolinska institutet, ECHA and EU member states.<br>Persons in enforcement have to carry our risk assessment and risk management tasks, but all of them do not have toxicology degree

Which areas of expertise areas are primarily lacking when you want to hire new personnel?

Which areas of expertise areas are primarily lacking when you want to hire new personnel?	Number of responses
Alternative (non-animal) in vitro methods	2 (16,7%)
Animal testing	1 (8,3%)
Bioinformatics	2 (16,7%)
Chemical analysis	0 (0,0%)
Chemistry/Environmental chemistry	2 (16,7%)
Ecotoxicology	3 (25,0%)
Epidemiology	4 (33,3%)
Exposure assessment	7 (58,3%)
QSAR and read-across	5 (41,7%)
Risk assessment	8 (66,7%)
Risk communication	4 (33,3%)
Risk management	3 (25,0%)
Statistics	3 (25,0%)
Systematic literature reviews	0 (0,0%)
Toxicology	7 (58,3%)
Other (please comment below)	2 (16,7%)
Total	53 (441,7%)

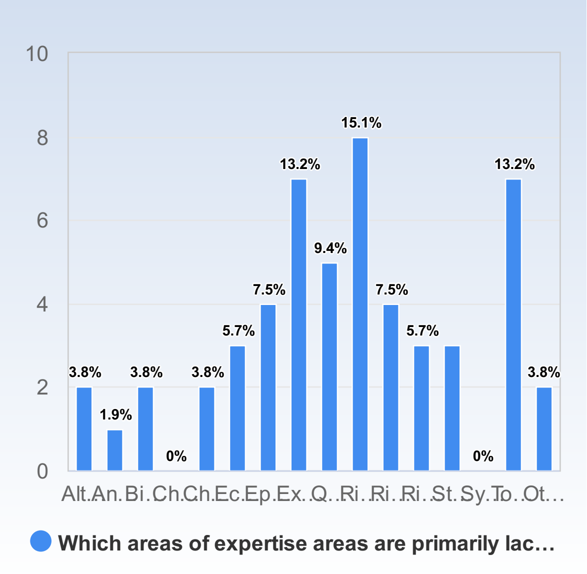

	Mean	Standard Deviation	Coefficient of Variation	Min	Lower Quartile	Median	Upper Quartile	Max
Which areas of expertise areas are primarily lacking when you want to hire new personnel?	20,5	3,8	18,7 %	12,0	18,0	21,0	23,0	27,0

Comment
regulatory toxicology and ecotoxicology
Recirculation, industrial side streams, new biomaterials, new processes for waste treatment health hazards
As I mentioned in my previous answers, industrial hygiene and occupational toxicology/toxicological risk assessment expertise are the main challenges. However, we have had some problems in finding also experts experienced in specific in vitro methods.
Regulatory expertise.

Please suggest university courses related to chemical risk assessment/communication you would like to see offered by academia in a near future.

Please suggest university courses related to chemical risk assessment/communication you would like to see offered by academia in a near future.
Regulatory toxicology
Waste management chemical and microbiological risks, thermochemical processing volatile gaseous components
Chemical risk assessment on food safety (Codex Alimentarius Commission)<br>Risk communication on food safety<br>In Finland there is only one university (far from capital area) that offers master's degree in toxicology. At least University of Helsinki should give such a possibility, too.
e.g. Evaluation of in vivo and in vitro studies for risk assessment, Mixture toxicity and cumulative risk assessment, Computational toxicology
Assessment on food contact materials. Hazard evaluation. Modern toxicology techniques (omics) applied to risk assments.
Any courses on risk assessment and risk communication would be welcomed. Also courses on toxicology (general) for non-toxicologists i.e. courses which would not aim at ERT but would help e.g. food chemists, nutritionists etc in their work within the area of risk assessment.
Consumer issues are challenging, because some chemical are in several authorities area - needs communication platform. Nanotehchnology is exellent example for this issue with Titanium dioxide and additve (E171).<br>In Denmark there is a kind of food safety issue discussion group, that also authorities follow and occasionally comment as well.<br>Fraunhofer ITEM provides good trainings in toxicolgy at least in food and feed area (provided by EFSA for EFSA connected people).
Regulatory toxicology-Risk assessment-medicinal products.<br>Ecotoxicology.
Medical / clinical acute toxicology
We have had discussions with UEF on the collaboration between our institute and UEF on the organisation of the occupational toxicology course. Whether this collaboration will happen is, however, still open. What comes to industrial hygiene, although there are courses related to it at UEF, it seems that number of students specializing for it is currently not sufficient for our needs.
Global regulatory (eco)toxicology.
In silico methods in practice for Plant Protection Products, alternative in vitro methods in regulatory toxicology,<br>Toxicokinetics. Genotoxicology, Dermal absorption, Formulation chemistry

Please indicate, if you expect a lack of expertise in the area of risk assessment/ communication in your organisation, in the near future.

Please indicate, if you expect a lack of expertise in the area of risk assessment/ communication in your organisation, in the near future.	Number of responses
1. Not expected	2 (15,4%)
2.	0 (0,0%)
3.	1 (7,7%)
4.	5 (38,5%)
5.	3 (23,1%)
6. Definitely expected	2 (15,4%)
Total	13 (100,0%)

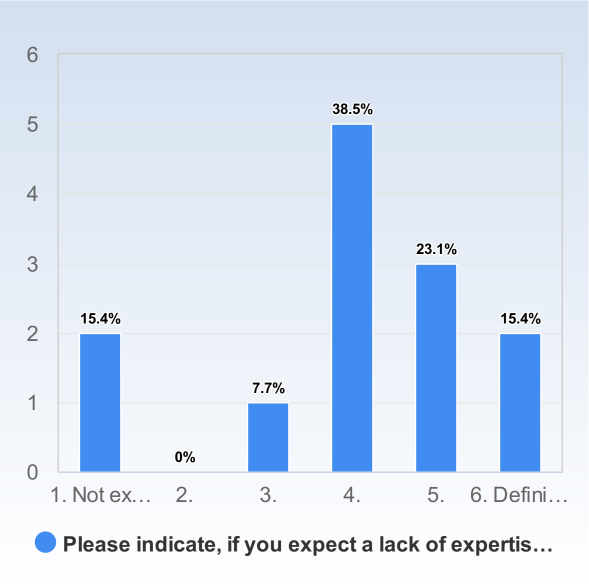

	Mean	Standard Deviation	Coefficient of Variation	Min	Lower Quartile	Median	Upper Quartile	Max
Please indicate, if you expect a lack of expertise in the area of risk assessment/ communication in your organisation, in the near future.	4,0	1,6	39,5 %	1,0	4,0	4,0	5,0	6,0

Please explain how you foresee to meet the needs of highly competent/qualified personnel in chemical risk assessment/communication in your organization five years from now (e.g. external courses, workshops or internal courses).

Please explain how you foresee to meet the needs of highly competent/qualified personnel in chemical risk assessment/communication in your organization five years from now (e.g. external courses, workshops or internal courses).
Good working circumstances, larger team supporting the individual expert in his/her job
First of all, prioritization and funding should be made available for the key factors still needing research (e.g. identifying carcinogenic risks) in the national research funding. If this will not be in the priority on the national funding it will not be well in the budgets and resource allocation of the collaborative institutes and it will be very difficult to organize and perform appropriate research and training.
1. Hands on experience<br>2. Legislation development by the policy makers<br>3. QMS reporting at work when new processes are developed
Internal training, BTSF and other external courses, conferences,
Participation in relevant research and external courses
External courses and external training programmes and workshops.
Through internal and external training. Through keeping abreast of the new developments at EFSA, ECHA, EMA, EC, FDA, EPA, JECFA, IPCS, FAO/WHO, ILSI etc. Through training courses, workshops, guidance documents etc organised by the aforementioned bodies.
There is need for understanding and communicating of safety issues and explanations of withdrawals or bans to consumers.<br>Currently EU and for example Horizon Europe programs asks in partnerships a collaboration and chemical safety issues are also included.
External courses, workshops.
Finnish Poison Information Center is a 60-year old center located within the largest hospital district of Finland (HUS). We rely on strong collaboration with the clinicians and good contacts with other Finnish authorities. We have our own training program for new employees as well as a biweekly own scientific meeting program. We also collaborate with Nordic Poison Centres and participate in external conferences and courses.
I already mentioned that trainee -program we have initially planned and discussions with UEF related to occupational toxicology course. We have also discussed with UEF on the possibility of our personnel to participate for some toxicology courses in UEF (as part of continued education). We are also following closely external courses available in Europe and for example some of our experts have participated in toxicology/risk assessment courses organised by Karoliska Institute.
Internal/external training and utilizing external consultants.
Internal or external courses on specific topics, eg. QSAR, in vitro methods; risk assessment in general.<br>With continuous learning and updating education using external courses.<br>Co-operation with European colleagues. ECHA workshops.

Please give suggestions what can be done nationally to optimize the numbers of competent persons for your organization.

Please give suggestions what can be done nationally to optimize the numbers of competent persons for your organization.
Good teachers, marketing, innovative learning methods, learning by doing
National policy makers should update legislation taking into consideration biodiversity and chemical burden for each new chemical compound (i.e. Do Not Signifcant Harm policy from EU).
Increase of toxicology education, especially in geographical areas where it is needed. ALSO risk assessment education!<br>Resources to recruit needs to be increased.
There are plans to establish a toxicological centre of expertise in Finland that would develop toxicological expertise, in particular regarding risk assessment. This plan now waits decisions and resources from the government in order to realize.
By providing enough basic training options for potential students (on txicology, ecotoxicology) is essential starting point. Alsdo providing options for Master of Science graduated (on chemistry, ecology, medicine, biology...) to further specialize to toxicology as a second graduations (up to PhD level).
Perhaps on-line training courses for self-learning could be developed.
Enough delivery of academia level educated candidates.
Need to attract more medical doctors to the field.
See also my previous replies. Increase collaboration between universities and research institutes and e.g. industry who also needs this expertise. Ensure funding; e.g. funding related to organising such kind of trainee positions in this field would be useful.
More co-operation with industry during studies.
To establish national toxicology centre or otherwise create a functioning and flexible network of experts

Please suggest how the Nordic countries could act jointly to optimize the numbers of competent persons for your organization.

Please suggest how the Nordic countries could act **jointly** to optimize the numbers of competent persons for your organization.
Co-operation like workshops, webinars/seminars, subgroups of special items,<br><br>Regulation is same in EU-level. Is Nordic countries right forum?
Please see the previous reply.
Food safety risk assessment courses in English given by the Nordic countries together.<br>Something like Nordiska Hälsovårdshögskolan should be founded again?
Nordic countries could organize joint training courses
Maybe a joint Nordic programme in one of nordic countri, or even splitting the expertise area between different Nordic counties (if resources cannot be put on one country only).
Perhaps creating a strong network of the expertise in the Nordic countries. A roster of experts and expertise could be established similar to JECFA or EFSA.
Common trainings and projects organized by HSSD or NIVA for example or other nordic organisations
Join training of academy students.<br>Student exchange between universities.<br>Nordic workshops.
Increase collaboration to provide training and education options.
We should consider organising post-graduate training course jointly.
Co-operation with different stakeholders, e.g. industry, organizations, authorities, universities.
Continue co-operation in trainings / courses on specific topics, ensure funding eg. via NKE (the Nordic Working Group for Chemicals, Enviroment an Health).<br>Nordic level co-operation in university level education DTU, KI, Göteborg?? better co-operation network between Nordic universities offering ecotox and tox education. A list of Nordic persons with their expertise could be useful.

Do you see positive possibilities for a closer, formalized cooperation between the Nordic countries in the area of chemical risk analysis, including training?

Do you see positive possibilities for a closer, formalized cooperation between the Nordic countries in the area of chemical risk analysis, including training?	Number of responses
YES	10 (76,9%)
NO	3 (23,1%)
Total	13 (100,0%)

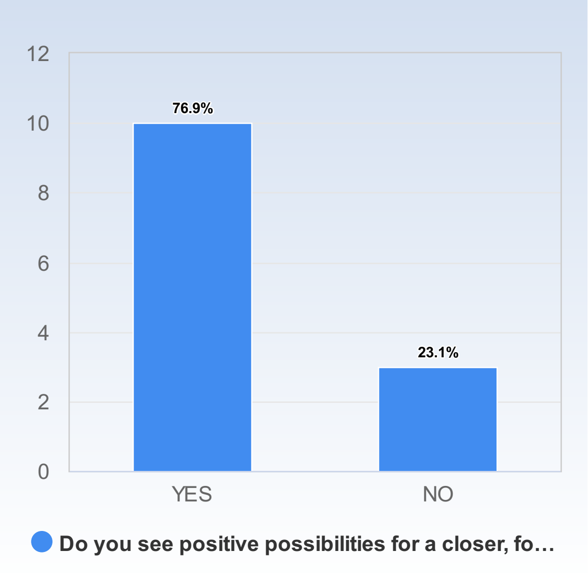

	Mean	Standard Deviation	Coefficient of Variation	Min	Lower Quartile	Median	Upper Quartile	Max
Do you see positive possibilities for a closer, formalized cooperation between the Nordic countries in the area of chemical risk analysis, including training?	1,2	0,4	35,6 %	1,0	1,0	1,0	1,0	2,0

If YES - How could this cooperation be accomplished? / If NO - Why would that be hard to accomplish?
Eu-level is better.
In principle, many of the research requirements are quite the same in the Nordic countries. Still, it is not easy to organize such projects that would satisfy some standard definitions and harmonisation, because of lack of organization and resources for research collaboration, and heterogeneity between the research groups in their goals and projects
Maybe in the field pharma, but in biotech (recycling, utilization of sidestreams) very difficult for conflicting interests
Co-organized training to found the basis for cooperation (e.g. mutual language on concepts is needed)<br>We already have some research cooperation, although its sthrengthening would benefit the quality of risk assessment.
NO, we suggest that European level would be better
I se ths positive option. But I cannot recognize the roadblocks. I guess the key is: how to finance this? Are there enough capacity and number of teachers / professors?
see above
look pervious page
See Q17.
We already collaborate with Nordic Poison Centres in terms of antidote use, for example.
For example organising post-graduate training program in this field.
PPP Northern Zone cooperation is already strong but needs to be continued.<br>More unformalized Nordic cooperation would be needed. That cooperation could be regular meetings in risk assessment issues or Ad Hoc meetings when needed.

How many of your experts within chemical risk assessment/communication are involved in international assignments related to the organizations indicated below?

EFSA panels:

EFSA panels:	Number of responses
0	5 (83,3%)
1	0 (0,0%)
2	1 (16,7%)
3	0 (0,0%)
4	0 (0,0%)
5	0 (0,0%)
6	0 (0,0%)
7	0 (0,0%)
8	0 (0,0%)
9	0 (0,0%)
Total	6 (100,0%)

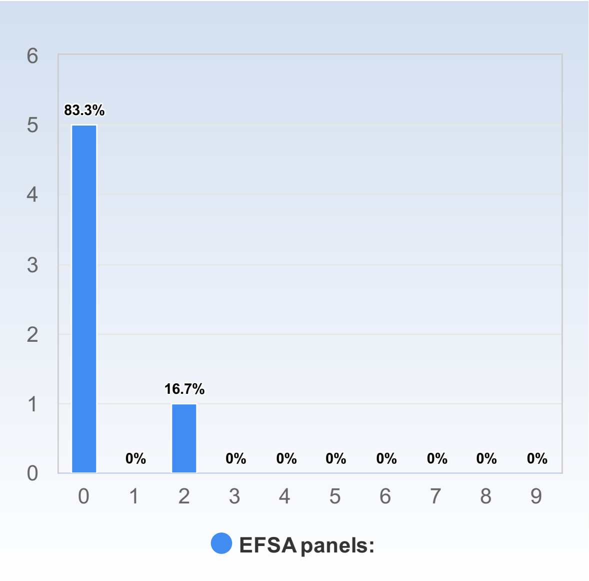

	Mean	Standard Deviation	Coefficient of Variation	Min	Lower Quartile	Median	Upper Quartile	Max
EFSA panels:	0,3	0,8	244,9 %	0,0	0,0	0,0	0,0	2,0

ECHA expert groups and member state committee:

ECHA expert groups and member state committee:	Number of responses
0	5 (62,5%)
1	1 (12,5%)
2	1 (12,5%)
3	0 (0,0%)
4	0 (0,0%)
5	0 (0,0%)
6	0 (0,0%)
7	0 (0,0%)
8	1 (12,5%)
9	0 (0,0%)
Total	8 (100,0%)

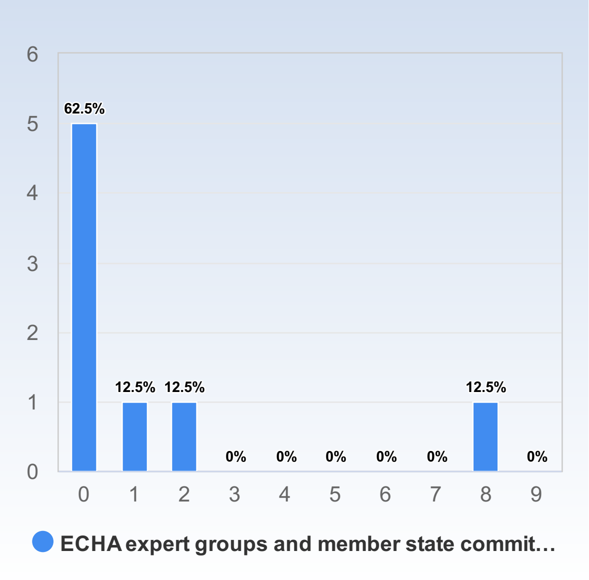

	Mean	Standard Deviation	Coefficient of Variation	Min	Lower Quartile	Median	Upper Quartile	Max
ECHA expert groups and member state committee:	1,4	2,8	201,8 %	0,0	0,0	0,0	1,5	8,0

EC scientific committees:

EC scientific committees:	Number of responses
0 - 2	6 (85,7%)
3 - 5	0 (0,0%)
6 - 8	0 (0,0%)
9 - 11	0 (0,0%)
12 - 14	0 (0,0%)
15 - 17	0 (0,0%)
18 - 20	1 (14,3%)
21 - 23	0 (0,0%)
24 - 26	0 (0,0%)
27 - 29	0 (0,0%)
Total	7 (100,0%)

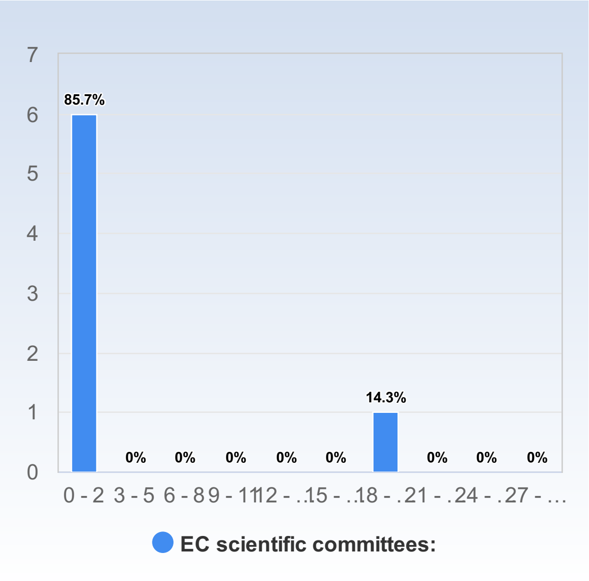

	Mean	Standard Deviation	Coefficient of Variation	Min	Lower Quartile	Median	Upper Quartile	Max
EC scientific committees:	3,1	7,5	237,7 %	0,0	0,0	0,0	1,0	20,0

EMA committees or working groups:

EMA committees or working groups:	Number of responses
0 - 1	5 (83,3%)
2 - 3	0 (0,0%)
4 - 5	0 (0,0%)
6 - 7	0 (0,0%)
8 - 9	0 (0,0%)
10 - 11	1 (16,7%)
12 - 13	0 (0,0%)
14 - 15	0 (0,0%)
16 - 17	0 (0,0%)
18 - 19	0 (0,0%)
Total	6 (100,0%)

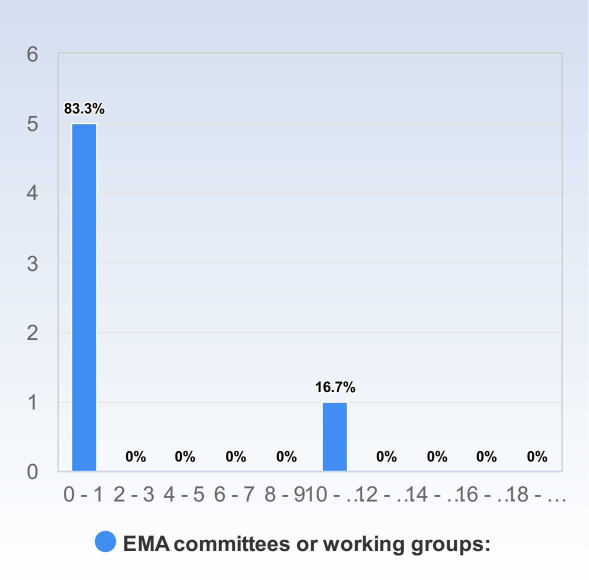

	Mean	Standard Deviation	Coefficient of Variation	Min	Lower Quartile	Median	Upper Quartile	Max
EMA committees or working groups:	1,7	4,1	244,9 %	0,0	0,0	0,0	0,0	10,0

OECD working groups:

OECD working groups:	Number of responses
0	5 (71,4%)
1	1 (14,3%)
2	0 (0,0%)
3	0 (0,0%)
4	1 (14,3%)
5	0 (0,0%)
6	0 (0,0%)
7	0 (0,0%)
8	0 (0,0%)
9	0 (0,0%)
Total	7 (100,0%)

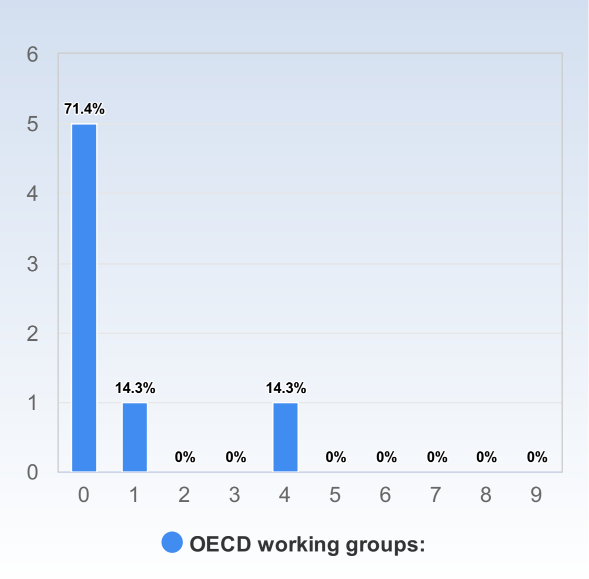

	Mean	Standard Deviation	Coefficient of Variation	Min	Lower Quartile	Median	Upper Quartile	Max
OECD working groups:	0,7	1,5	209,4 %	0,0	0,0	0,0	0,5	4,0

WHO expert groups:

WHO expert groups:	Number of responses
0	5 (55,6%)
1	2 (22,2%)
2	1 (11,1%)
3	1 (11,1%)
4	0 (0,0%)
5	0 (0,0%)
6	0 (0,0%)
7	0 (0,0%)
8	0 (0,0%)
9	0 (0,0%)
Total	9 (100,0%)

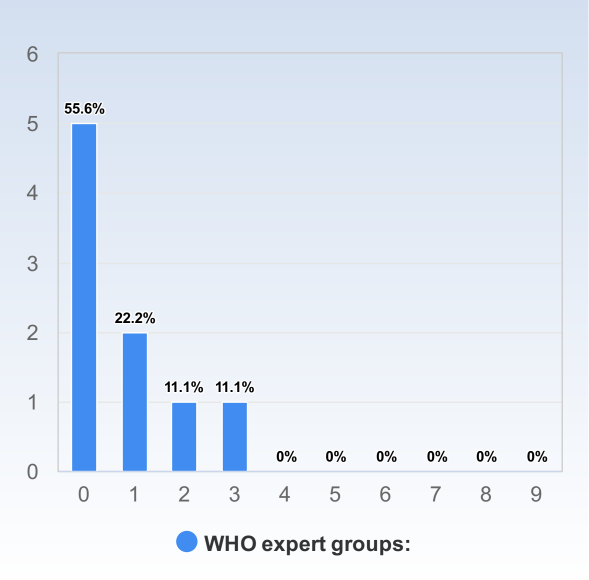

	Mean	Standard Deviation	Coefficient of Variation	Min	Lower Quartile	Median	Upper Quartile	Max
WHO expert groups:	0,8	1,1	140,5 %	0,0	0,0	0,0	1,0	3,0

Other(s) (please comment below)

Other(s) (please comment below)	Number of responses
0	2 (50,0%)
1	2 (50,0%)
2	0 (0,0%)
3	0 (0,0%)
4	0 (0,0%)
5	0 (0,0%)
6	0 (0,0%)
7	0 (0,0%)
8	0 (0,0%)
9	0 (0,0%)
Total	4 (100,0%)

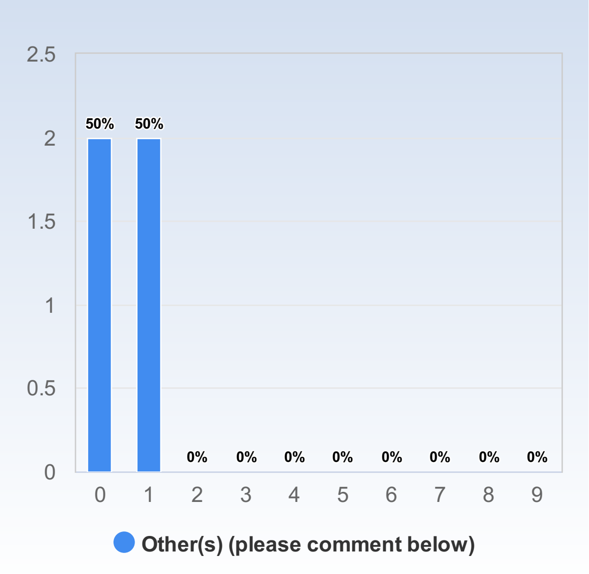

	Mean	Standard Deviation	Coefficient of Variation	Min	Lower Quartile	Median	Upper Quartile	Max
Other(s) (please comment below)	0,5	0,6	115,5 %	0,0	0,0	0,5	1,0	1,0

Comment
One person part-time
No personnel available for such assignments.
It would be desirable to participate, but our minimal resources are not enough to do so. At the moment only national risk assessment assignments could be carried out.
No one in that high level forums.
One ex-EFSA staff member who coordinated EFSA CONTAM WGs and the PANEL and developed CONTAM risk assessments for 10 years works in our company. She has also some experience in risk communication and/or management from EC, EFSA and ECHA, as well as from national FSAs.
Nordic Expert Group for Criteria Documentation of Health Risks from Chemicals (NEG). Related to ECHA expert groups, one of our experts is a member of RAC but there are two others who have been assigned as member's advisors.
Industry experts from individual companies usually not participating these panels.

Do you expect that the number of personnel involved in international assignments (c.f. previous question) will increase or decrease in the coming 5 years?

Do you expect that the number of personnel involved in international assignments (c.f. previous question) will increase or decrease in the coming 5 years?	Number of responses
Increase	4 (36,4%)
Decrease	1 (9,1%)
No change is expected	6 (54,5%)
Total	11 (100,0%)

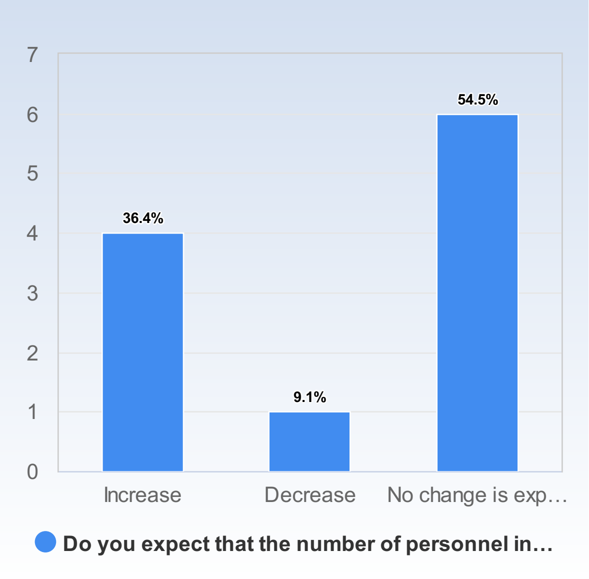

	Mean	Standard Deviation	Coefficient of Variation	Min	Lower Quartile	Median	Upper Quartile	Max
Do you expect that the number of personnel involved in international assignments (c.f. previous question) will increase or decrease in the coming 5 years?	2,2	1,0	45,0 %	1,0	1,0	3,0	3,0	3,0

Please comment.
may be some
After the retirement of the part-time expert, there will be no substitutes available at our institute (other than possible participation by the retired experts)
Increasing research funding is coming from EU and national funding sources are aligning their requirements for funding with the EU
We really hope that the answer ‘Increase’ would happen, but if thinking realistically, probably no change is expected
Actually no view to this question.
We are a CRO/consultant company and have therefore conflict of interest as regards to participation to the work of the above public bodies.
REPRESENTATION IN INTERNATIONAL ORGANISATIONS ALREADY AT HIGH LEVEL.

Any additional reflections or comments regarding competence provision needs in the area of risk assessment/communication?

Any additional reflections or comments regarding competence provision needs in the area of risk assessment/communication?
Please, take the number of people given in this questionnaire only indicative since it very difficult to assess the actual number of people involved in risk assessment / communication.
These answers reflect the situation in Finnish Food Authority's Risk Assessment Unit. There will be additional information in a separate form(s) from other units, where our answers are excluded.
No additional views.
Currently we do not have much solutions but we are open to discuss and we see a need for further co-operation.

SI Part E

System generated analyses of the result from the Norwegian respondents

Risk analysis competence provision questionnaire for Norway

Respondents: 59
Answer Count: 8
Answer Frequency: 13,56 %

Please indicate your area of affiliation? (mandatory)

Please indicate your area of affiliation? (mandatory)	Number of responses
Research institute	3 (37,5%)
National authority	1 (12,5%)
Regional authority	0 (0,0%)
Hospital practice	2 (25,0%)
Industry/Business	1 (12,5%)
NGO	0 (0,0%)
Consultant	0 (0,0%)
Other	1 (12,5%)
Total	8 (100,0%)

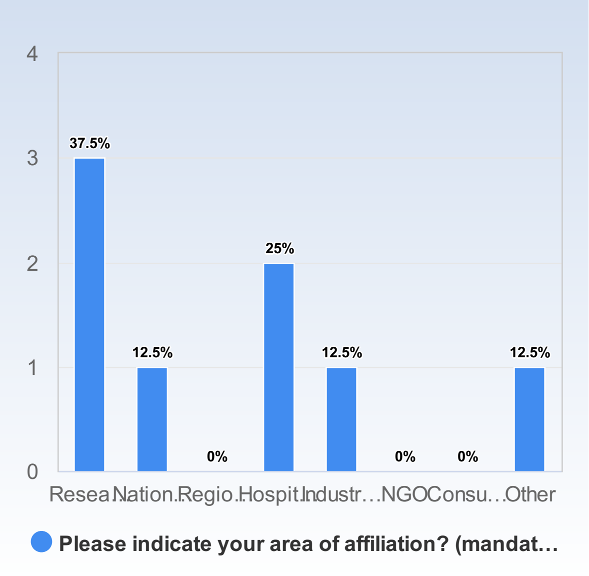

	Mean	Standard Deviation	Coefficient of Variation	Min	Lower Quartile	Median	Upper Quartile	Max
Please indicate your area of affiliation? (mandatory)	4,4	2,8	63,4 %	2,0	2,0	4,0	5,5	10,0

What is the number of personnel in chemical risk assessment/risk communication in your organization?

What is the number of personnel in chemical risk assessment/risk communication in your organization?	Number of responses
0 - 10	4 (50,0%)
11 - 21	2 (25,0%)
22 - 32	0 (0,0%)
33 - 43	1 (12,5%)
44 - 54	0 (0,0%)
55 - 65	1 (12,5%)
66 - 76	0 (0,0%)
77 - 87	0 (0,0%)
88 - 98	0 (0,0%)
99 - 109	0 (0,0%)
Total	8 (100,0%)

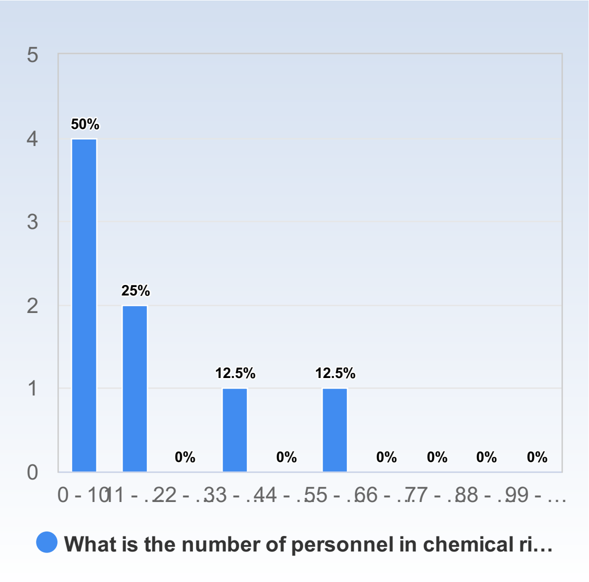

	Mean	Standard Deviation	Coefficient of Variation	Min	Lower Quartile	Median	Upper Quartile	Max
What is the number of personnel in chemical risk assessment/risk communication in your organization?	18,6	21,3	114,1 %	1,0	3,5	11,0	28,0	63,0

What specialization, in chemical risk assessment /communication, do you have in your organization?
Please estimate the number of personnel for each specialization.

Alternative (non-animal) in vitro methods

Alternative (non-animal) in vitro methods	Number of responses
0	1 (16,7%)
1	1 (16,7%)
2	0 (0,0%)
3	2 (33,3%)
4	2 (33,3%)
5	0 (0,0%)
6	0 (0,0%)
7	0 (0,0%)
8	0 (0,0%)
9	0 (0,0%)
Total	6 (100,0%)

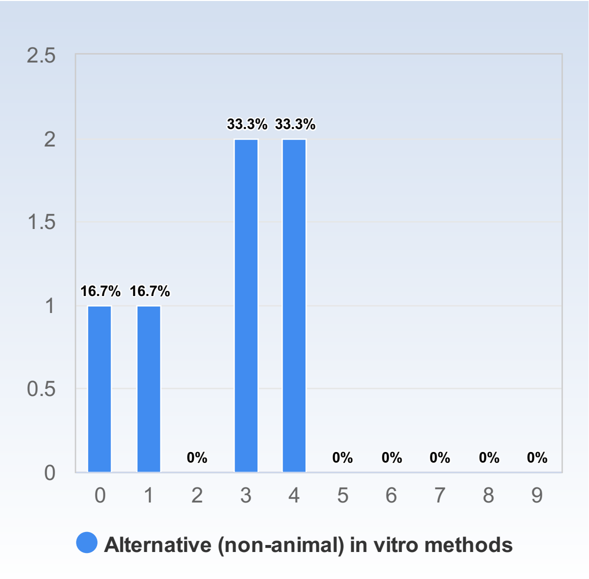

	Mean	Standard Deviation	Coefficient of Variation	Min	Lower Quartile	Median	Upper Quartile	Max
Alternative (non-animal) in vitro methods	2,5	1,6	65,7 %	0,0	2,0	3,0	3,5	4,0

Animal testing

Animal testing	Number of responses
0 - 1	1 (16,7%)
2 - 3	2 (33,3%)
4 - 5	2 (33,3%)
6 - 7	0 (0,0%)
8 - 9	0 (0,0%)
10 - 11	0 (0,0%)
12 - 13	0 (0,0%)
14 - 15	1 (16,7%)
16 - 17	0 (0,0%)
18 - 19	0 (0,0%)
Total	6 (100,0%)

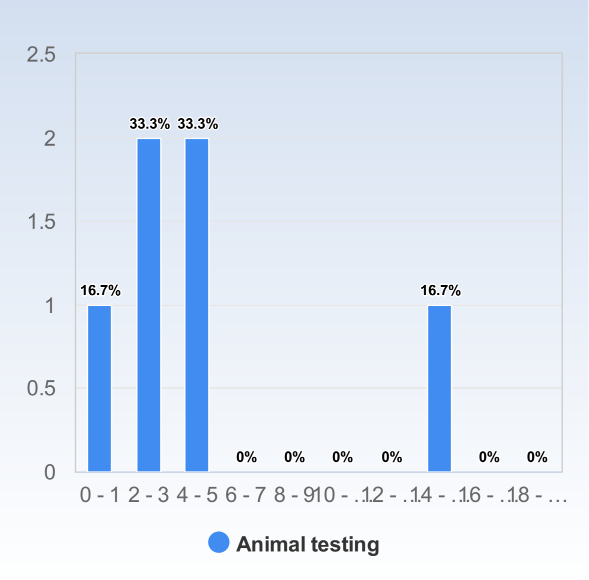

	Mean	Standard Deviation	Coefficient of Variation	Min	Lower Quartile	Median	Upper Quartile	Max
Animal testing	4,8	5,2	107,4 %	1,0	2,0	3,0	4,5	15,0

Bioinformatics

Bioinformatics	Number of responses
0	1 (25,0%)
1	1 (25,0%)
2	0 (0,0%)
3	2 (50,0%)
4	0 (0,0%)
5	0 (0,0%)
6	0 (0,0%)
7	0 (0,0%)
8	0 (0,0%)
9	0 (0,0%)
Total	4 (100,0%)

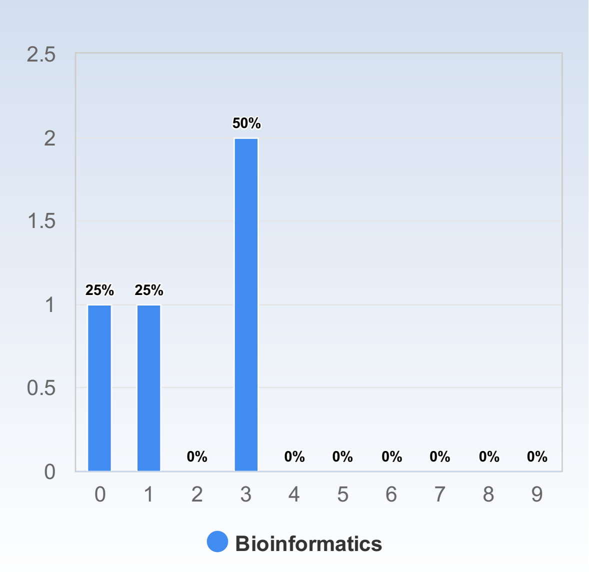

	Mean	Standard Deviation	Coefficient of Variation	Min	Lower Quartile	Median	Upper Quartile	Max
Bioinformatics	1,8	1,5	85,7 %	0,0	0,5	2,0	3,0	3,0

Chemical analysis

Chemical analysis	Number of responses
0	0 (0,0%)
1	1 (25,0%)
2	1 (25,0%)
3	1 (25,0%)
4	1 (25,0%)
5	0 (0,0%)
6	0 (0,0%)
7	0 (0,0%)
8	0 (0,0%)
9	0 (0,0%)
Total	4 (100,0%)

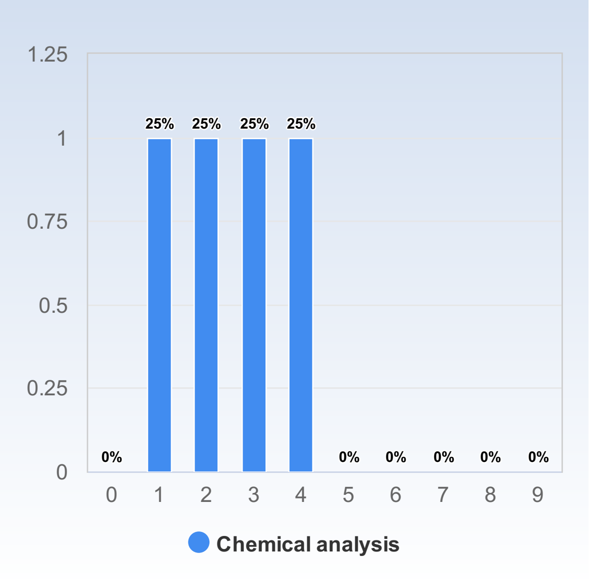

	Mean	Standard Deviation	Coefficient of Variation	Min	Lower Quartile	Median	Upper Quartile	Max
Chemical analysis	2,5	1,3	51,6 %	1,0	1,5	2,5	3,5	4,0

Chemistry/Environmental chemistry

Chemistry/Environmental chemistry	Number of responses
1	1 (16,7%)
2	2 (33,3%)
3	1 (16,7%)
4	0 (0,0%)
5	1 (16,7%)
6	0 (0,0%)
7	0 (0,0%)
8	0 (0,0%)
9	0 (0,0%)
10	1 (16,7%)
Total	6 (100,0%)

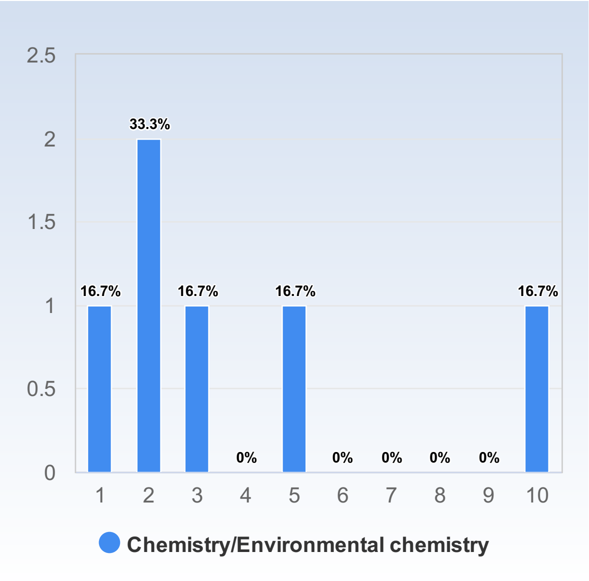

	Mean	Standard Deviation	Coefficient of Variation	Min	Lower Quartile	Median	Upper Quartile	Max
Chemistry/Environmental chemistry	3,8	3,3	86,4 %	1,0	2,0	2,5	4,0	10,0

Ecotoxicology

Ecotoxicology	Number of responses
0	0 (0,0%)
1	1 (50,0%)
2	1 (50,0%)
3	0 (0,0%)
4	0 (0,0%)
5	0 (0,0%)
6	0 (0,0%)
7	0 (0,0%)
8	0 (0,0%)
9	0 (0,0%)
Total	2 (100,0%)

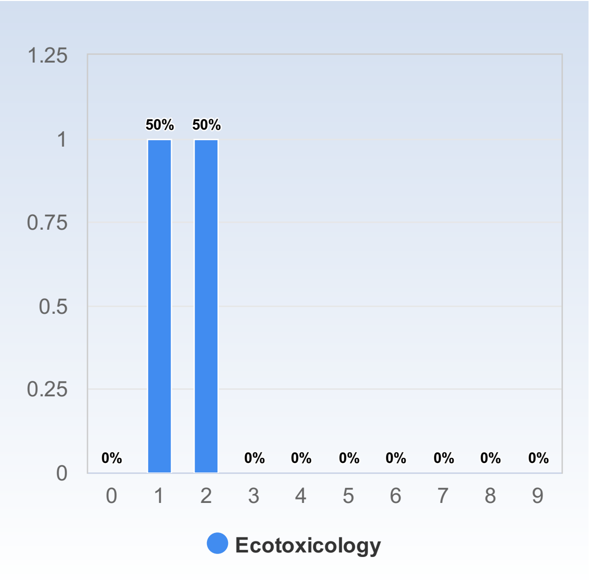

	Mean	Standard Deviation	Coefficient of Variation	Min	Lower Quartile	Median	Upper Quartile	Max
Ecotoxicology	1,5	0,7	47,1 %	1,0	1,5	1,5	1,5	2,0

Epidemiology

Epidemiology	Number of responses
0 - 2	2 (40,0%)
3 - 5	1 (20,0%)
6 - 8	0 (0,0%)
9 - 11	1 (20,0%)
12 - 14	0 (0,0%)
15 - 17	0 (0,0%)
18 - 20	1 (20,0%)
21 - 23	0 (0,0%)
24 - 26	0 (0,0%)
27 - 29	0 (0,0%)
Total	5 (100,0%)

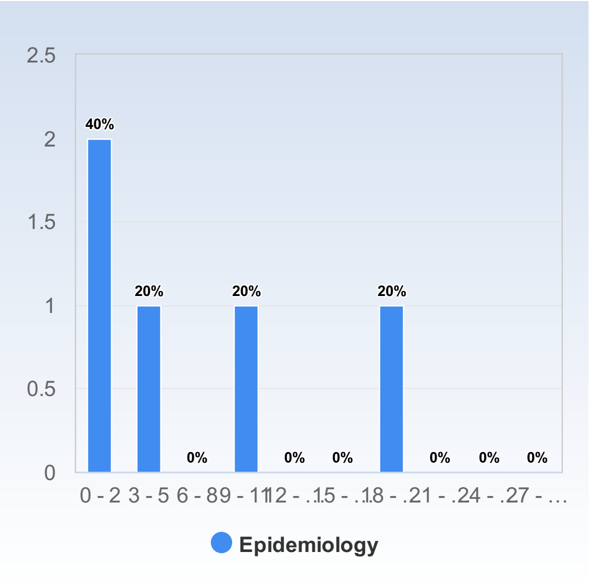

	Mean	Standard Deviation	Coefficient of Variation	Min	Lower Quartile	Median	Upper Quartile	Max
Epidemiology	7,4	8,0	107,9 %	0,0	2,0	5,0	10,0	20,0

Exposure assessment

Exposure assessment	Number of responses
0	0 (0,0%)
1	2 (25,0%)
2	2 (25,0%)
3	0 (0,0%)
4	2 (25,0%)
5	2 (25,0%)
6	0 (0,0%)
7	0 (0,0%)
8	0 (0,0%)
9	0 (0,0%)
Total	8 (100,0%)

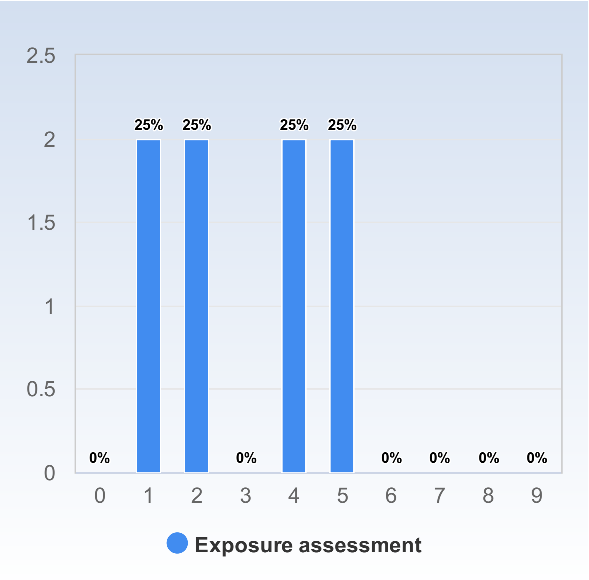

	Mean	Standard Deviation	Coefficient of Variation	Min	Lower Quartile	Median	Upper Quartile	Max
Exposure assessment	3,0	1,7	56,3 %	1,0	1,5	3,0	4,5	5,0

QSAR and read-across

QSAR and read-across	Number of responses
0	1 (33,3%)
1	0 (0,0%)
2	2 (66,7%)
3	0 (0,0%)
4	0 (0,0%)
5	0 (0,0%)
6	0 (0,0%)
7	0 (0,0%)
8	0 (0,0%)
9	0 (0,0%)
Total	3 (100,0%)

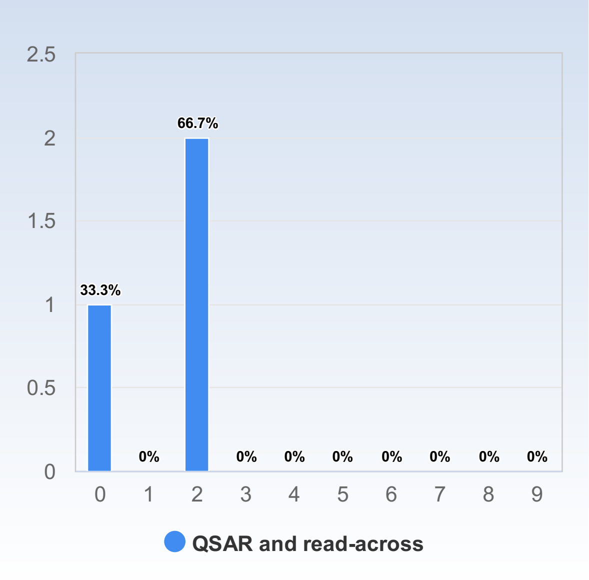

	Mean	Standard Deviation	Coefficient of Variation	Min	Lower Quartile	Median	Upper Quartile	Max
QSAR and read-across	1,3	1,2	86,6 %	0,0	1,0	2,0	2,0	2,0

Risk assessment

Risk assessment	Number of responses
0 - 2	2 (25,0%)
3 - 5	4 (50,0%)
6 - 8	0 (0,0%)
9 - 11	0 (0,0%)
12 - 14	0 (0,0%)
15 - 17	0 (0,0%)
18 - 20	1 (12,5%)
21 - 23	0 (0,0%)
24 - 26	1 (12,5%)
27 - 29	0 (0,0%)
Total	8 (100,0%)

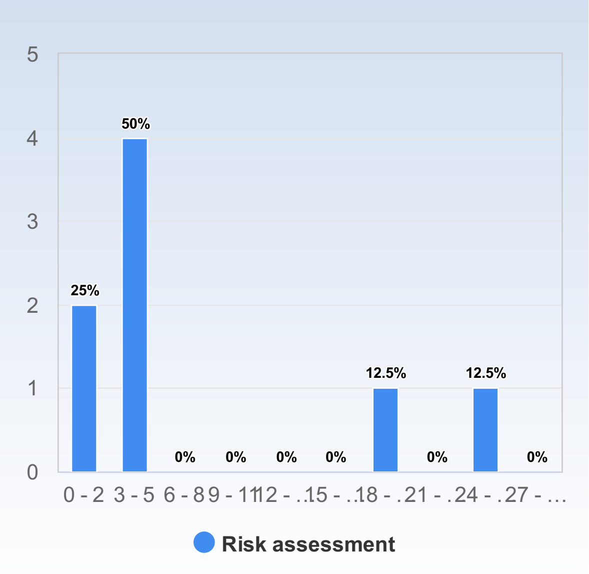

	Mean	Standard Deviation	Coefficient of Variation	Min	Lower Quartile	Median	Upper Quartile	Max
Risk assessment	7,9	8,7	110,8 %	1,0	2,5	4,5	11,5	25,0

Risk communication

Risk communication	Number of responses
0	1 (20,0%)
1	0 (0,0%)
2	1 (20,0%)
3	1 (20,0%)
4	1 (20,0%)
5	1 (20,0%)
6	0 (0,0%)
7	0 (0,0%)
8	0 (0,0%)
9	0 (0,0%)
Total	5 (100,0%)

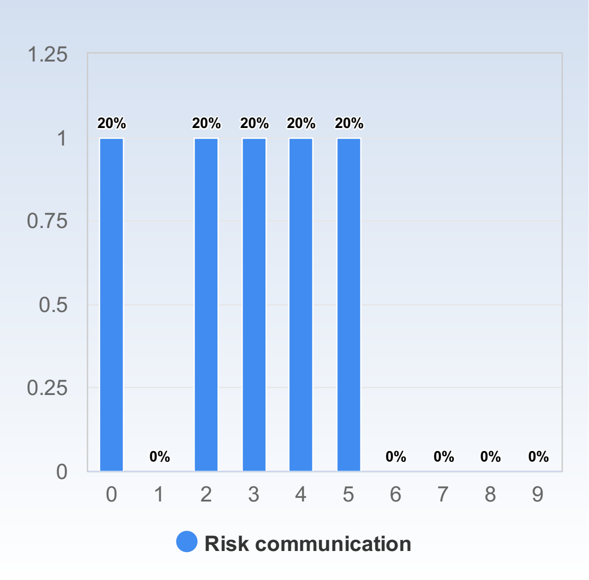

	Mean	Standard Deviation	Coefficient of Variation	Min	Lower Quartile	Median	Upper Quartile	Max
Risk communication	2,8	1,9	68,7 %	0,0	2,0	3,0	4,0	5,0

Risk management

Risk management	Number of responses
0	0 (0,0%)
1	1 (33,3%)
2	1 (33,3%)
3	0 (0,0%)
4	1 (33,3%)
5	0 (0,0%)
6	0 (0,0%)
7	0 (0,0%)
8	0 (0,0%)
9	0 (0,0%)
Total	3 (100,0%)

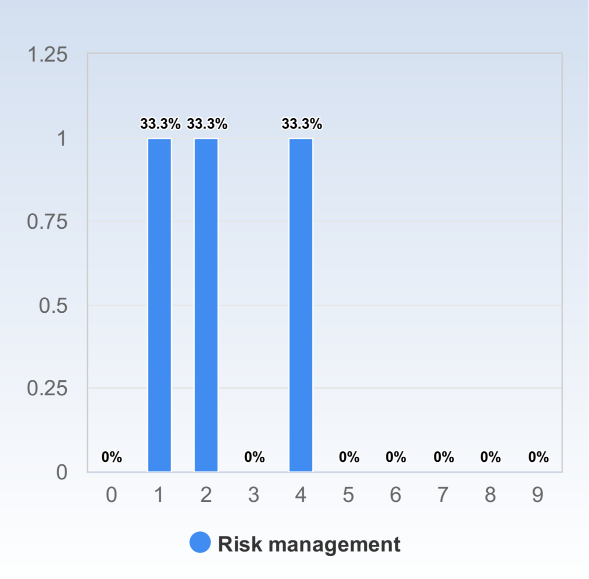

	Mean	Standard Deviation	Coefficient of Variation	Min	Lower Quartile	Median	Upper Quartile	Max
Risk management	2,3	1,5	65,5 %	1,0	1,5	2,0	3,0	4,0

Statistics

Statistics	Number of responses
0	0 (0,0%)
1	3 (50,0%)
2	0 (0,0%)
3	1 (16,7%)
4	1 (16,7%)
5	0 (0,0%)
6	0 (0,0%)
7	0 (0,0%)
8	1 (16,7%)
9	0 (0,0%)
Total	6 (100,0%)

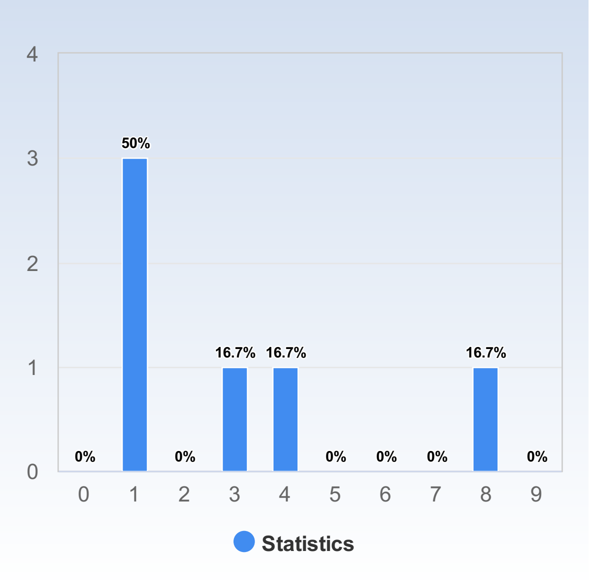

	Mean	Standard Deviation	Coefficient of Variation	Min	Lower Quartile	Median	Upper Quartile	Max
Statistics	3,0	2,8	91,9 %	1,0	1,0	2,0	3,5	8,0

Systematic literature reviews

Systematic literature reviews	Number of responses
0	0 (0,0%)
1	2 (33,3%)
2	1 (16,7%)
3	1 (16,7%)
4	1 (16,7%)
5	0 (0,0%)
6	0 (0,0%)
7	0 (0,0%)
8	1 (16,7%)
9	0 (0,0%)
Total	6 (100,0%)

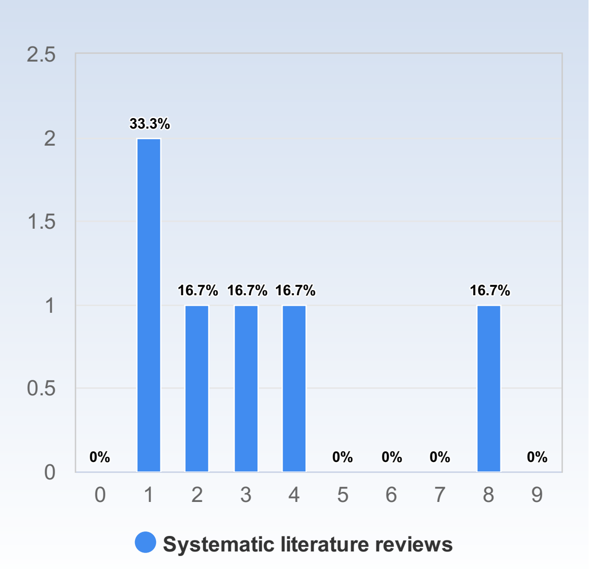

	Mean	Standard Deviation	Coefficient of Variation	Min	Lower Quartile	Median	Upper Quartile	Max
Systematic literature reviews	3,2	2,6	83,4 %	1,0	1,5	2,5	3,5	8,0

Toxicology

Toxicology	Number of responses
0 - 2	2 (25,0%)
3 - 5	2 (25,0%)
6 - 8	2 (25,0%)
9 - 11	0 (0,0%)
12 - 14	0 (0,0%)
15 - 17	1 (12,5%)
18 - 20	0 (0,0%)
21 - 23	0 (0,0%)
24 - 26	1 (12,5%)
27 - 29	0 (0,0%)
Total	8 (100,0%)

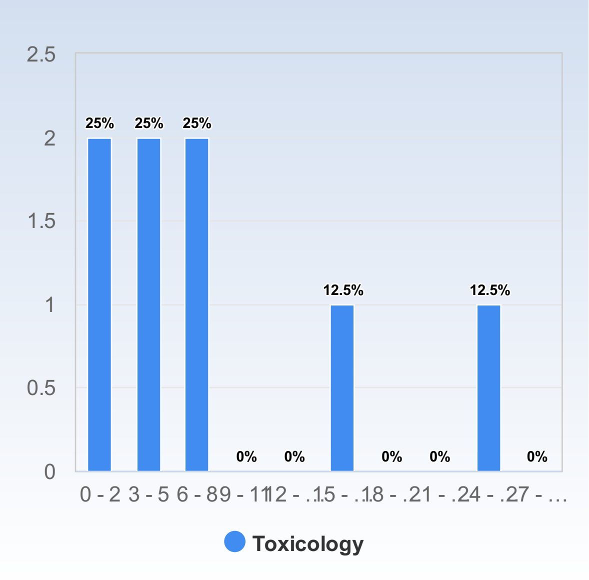

	Mean	Standard Deviation	Coefficient of Variation	Min	Lower Quartile	Median	Upper Quartile	Max
Toxicology	8,0	8,1	101,6 %	1,0	2,5	5,5	11,0	25,0

Other (please comment below)

Other (please comment below)	Number of responses
0	0 (0,0%)
1	0 (0,0%)
2	0 (0,0%)
3	1 (100,0%)
4	0 (0,0%)
5	0 (0,0%)
6	0 (0,0%)
7	0 (0,0%)
8	0 (0,0%)
9	0 (0,0%)
Total	1 (100,0%)

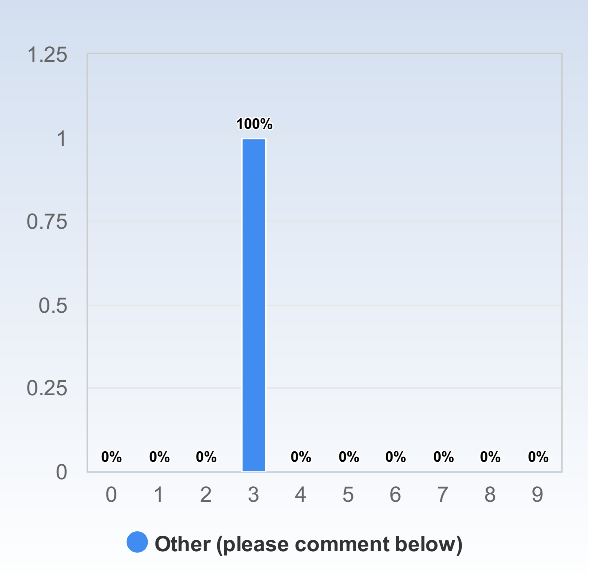

	Mean	Standard Deviation	Coefficient of Variation	Min	Lower Quartile	Median	Upper Quartile	Max
Other (please comment below)	3,0	0,0	NaN %	3,0	3,0	3,0	3,0	3,0

Comment
Specialisation is a relative term. I have some experience within these topics. In addition to myself I have colleagues who are specialised in risk assessment methodology, and SEVESO risk. This is of course also chemical risk assessment competency, but I have not included them in the number here since the questionnaire seems to be focused on chemical hazard as such, not so much how it is used.
Veterinary toxicology, wild and farm animal poisonings
it is very challenging to provide numbers since our institution is very big with many different specialities and not each speciality knows each other personally. the numbers might not reflect the real situation and must sees as an rough estimate.

How many of the personnel in your organization, with the chemical risk assessment /communication tasks you presented in the previous question, holds a:

Bachelor degree:

Bachelor degree:	Number of responses
-2147483648	0 (0,0%)
-2147483647	0 (0,0%)
-2147483646	0 (0,0%)
-2147483645	0 (0,0%)
-2147483644	0 (0,0%)
-2147483643	0 (0,0%)
-2147483642	0 (0,0%)
-2147483641	0 (0,0%)
-2147483640	0 (0,0%)
-2147483639	0 (0,0%)
Total	0 (0,0%)

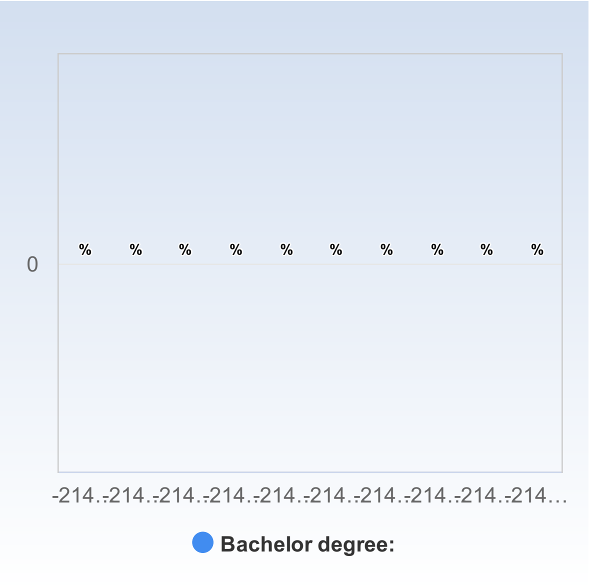

	Mean	Standard Deviation	Coefficient of Variation	Min	Lower Quartile	Median	Upper Quartile	Max
Bachelor degree:	0,0	0,0	NaN %	∞	0,0	0,0	0,0	-∞

Master degree:

Master degree:	Number of responses
1 - 3	3 (75,0%)
4 - 6	0 (0,0%)
7 - 9	0 (0,0%)
10 - 12	0 (0,0%)
13 - 15	0 (0,0%)
16 - 18	0 (0,0%)
19 - 21	0 (0,0%)
22 - 24	0 (0,0%)
25 - 27	0 (0,0%)
28 - 30	1 (25,0%)
Total	4 (100,0%)

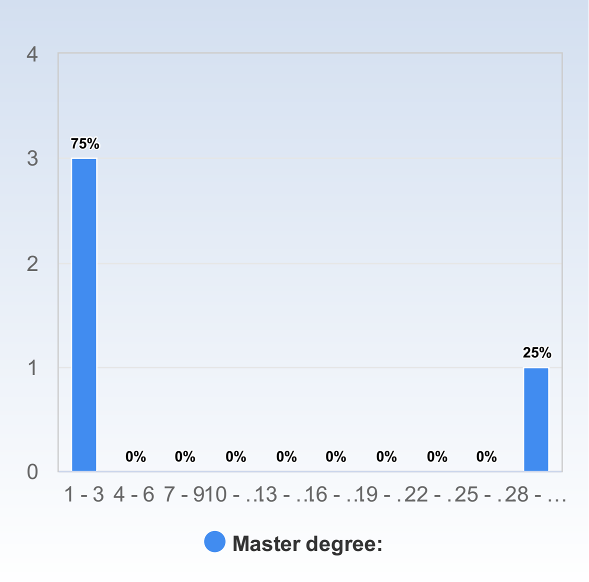

	Mean	Standard Deviation	Coefficient of Variation	Min	Lower Quartile	Median	Upper Quartile	Max
Master degree:	8,5	14,3	168,7 %	1,0	1,0	1,5	16,0	30,0

Doctoral degree (PhDs, MDs, Dtech (TkT, D.I.T), etc):

Doctoral degree (PhDs, MDs, Dtech (TkT, D.I.T), etc):	Number of responses
0 - 3	1 (14,3%)
4 - 7	1 (14,3%)
8 - 11	2 (28,6%)
12 - 15	0 (0,0%)
16 - 19	0 (0,0%)
20 - 23	1 (14,3%)
24 - 27	0 (0,0%)
28 - 31	1 (14,3%)
32 - 35	0 (0,0%)
36 - 39	1 (14,3%)
Total	7 (100,0%)

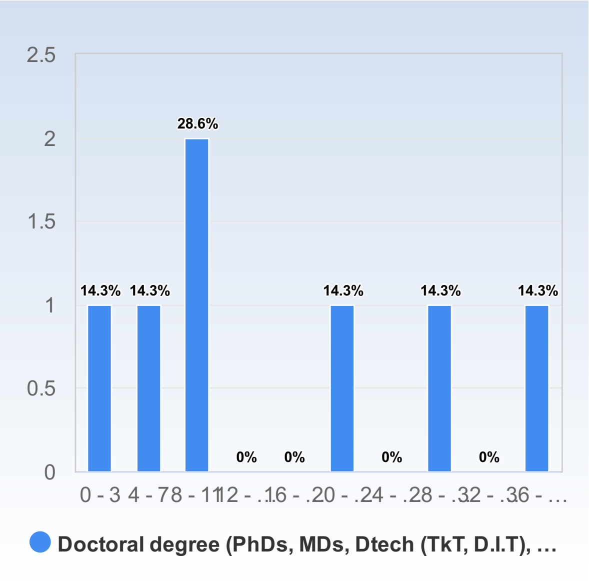

	Mean	Standard Deviation	Coefficient of Variation	Min	Lower Quartile	Median	Upper Quartile	Max
Doctoral degree (PhDs, MDs, Dtech (TkT, D.I.T), etc):	15,9	13,2	83,1 %	1,0	7,0	10,0	25,0	36,0

How many of your personnel are ERTs (European Registered Toxicologist)?

How many of your personnel are ERTs (European Registered Toxicologist)?	Number of responses
0 - 10	7 (100,0%)
11 - 21	0 (0,0%)
22 - 32	0 (0,0%)
33 - 43	0 (0,0%)
44 - 54	0 (0,0%)
55 - 65	0 (0,0%)
66 - 76	0 (0,0%)
77 - 87	0 (0,0%)
88 - 98	0 (0,0%)
99 - 109	0 (0,0%)
Total	7 (100,0%)

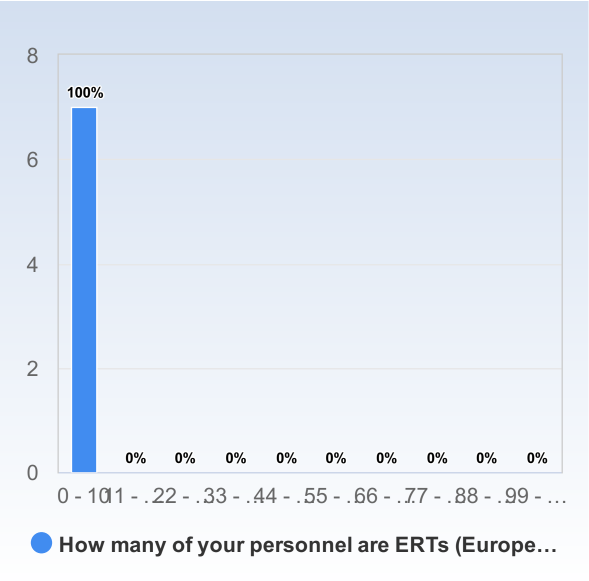

	Mean	Standard Deviation	Coefficient of Variation	Min	Lower Quartile	Median	Upper Quartile	Max
How many of your personnel are ERTs (European Registered Toxicologist)?	2,4	2,3	94,7 %	0,0	1,0	1,0	4,0	6,0

What is the present age profile of the risk analysis personnel in your organization?

< 40 years of age:

< 40 years of age:	Number of responses
0 - 3	1 (25,0%)
4 - 7	0 (0,0%)
8 - 11	1 (25,0%)
12 - 15	0 (0,0%)
16 - 19	1 (25,0%)
20 - 23	0 (0,0%)
24 - 27	0 (0,0%)
28 - 31	0 (0,0%)
32 - 35	1 (25,0%)
36 - 39	0 (0,0%)
Total	4 (100,0%)

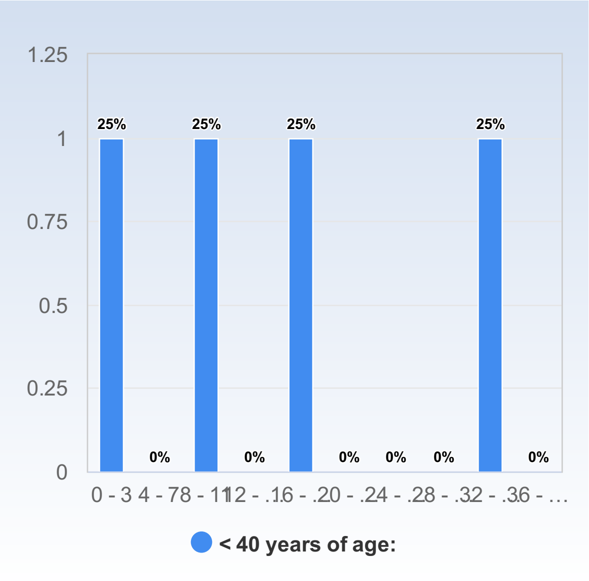

	Mean	Standard Deviation	Coefficient of Variation	Min	Lower Quartile	Median	Upper Quartile	Max
< 40 years of age:	15,3	13,1	86,2 %	2,0	6,0	13,0	24,5	33,0

40-50 years of age:

40-50 years of age:	Number of responses
0 - 5	5 (71,4%)
6 - 11	0 (0,0%)
12 - 17	0 (0,0%)
18 - 23	0 (0,0%)
24 - 29	1 (14,3%)
30 - 35	0 (0,0%)
36 - 41	0 (0,0%)
42 - 47	0 (0,0%)
48 - 53	1 (14,3%)
54 - 59	0 (0,0%)
Total	7 (100,0%)

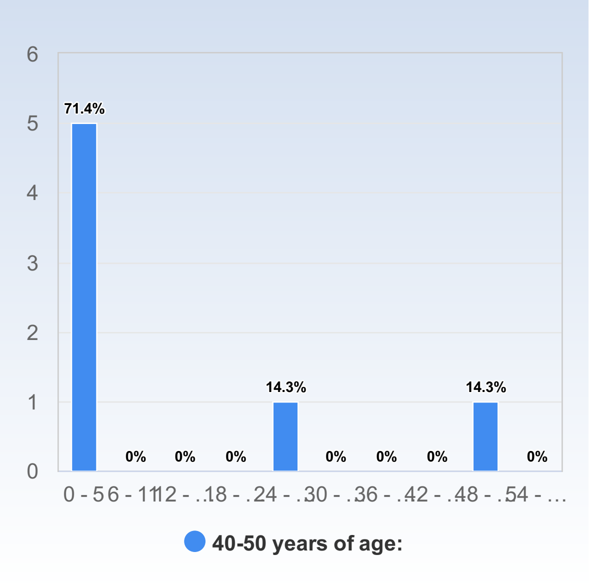

	Mean	Standard Deviation	Coefficient of Variation	Min	Lower Quartile	Median	Upper Quartile	Max
40-50 years of age:	12,6	19,4	154,2 %	1,0	1,5	3,0	14,5	52,0

50-60 years of age:

50-60 years of age:	Number of responses
2 - 5	1 (16,7%)
6 - 9	1 (16,7%)
10 - 13	0 (0,0%)
14 - 17	2 (33,3%)
18 - 21	0 (0,0%)
22 - 25	0 (0,0%)
26 - 29	0 (0,0%)
30 - 33	1 (16,7%)
34 - 37	0 (0,0%)
38 - 41	1 (16,7%)
Total	6 (100,0%)

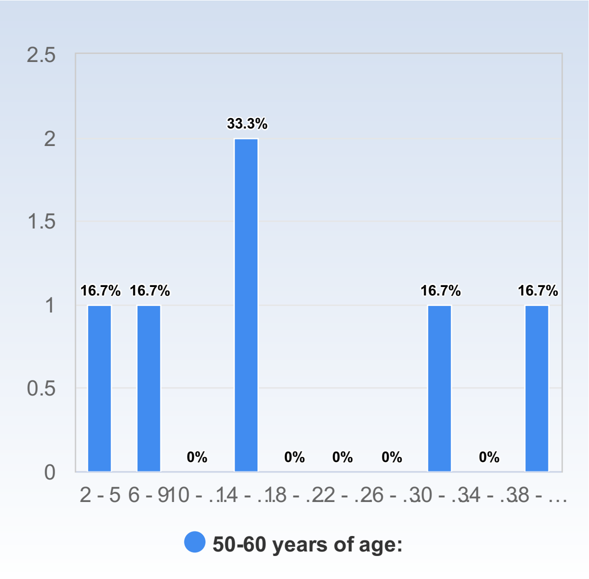

	Mean	Standard Deviation	Coefficient of Variation	Min	Lower Quartile	Median	Upper Quartile	Max
50-60 years of age:	18,2	14,4	79,4 %	2,0	10,5	15,5	23,0	40,0

60-65 years of age:

60-65 years of age:	Number of responses
1 - 2	1 (25,0%)
3 - 4	1 (25,0%)
5 - 6	0 (0,0%)
7 - 8	1 (25,0%)
9 - 10	0 (0,0%)
11 - 12	0 (0,0%)
13 - 14	0 (0,0%)
15 - 16	0 (0,0%)
17 - 18	0 (0,0%)
19 - 20	1 (25,0%)
Total	4 (100,0%)

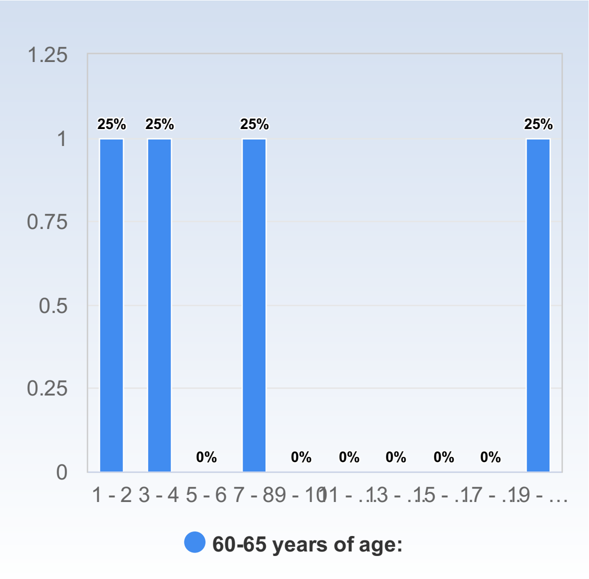

	Mean	Standard Deviation	Coefficient of Variation	Min	Lower Quartile	Median	Upper Quartile	Max
60-65 years of age:	8,0	8,5	106,6 %	1,0	2,0	5,5	14,0	20,0

> 65 years of age:

> 65 years of age:	Number of responses
0	1 (33,3%)
1	0 (0,0%)
2	0 (0,0%)
3	0 (0,0%)
4	0 (0,0%)
5	1 (33,3%)
6	0 (0,0%)
7	0 (0,0%)
8	1 (33,3%)
9	0 (0,0%)
Total	3 (100,0%)

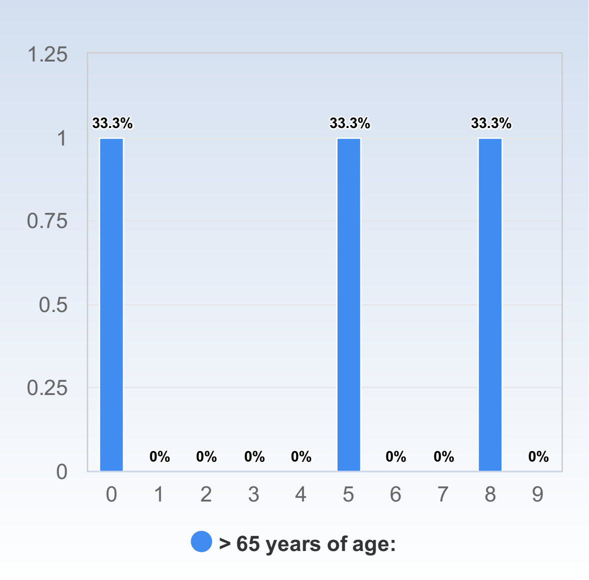

	Mean	Standard Deviation	Coefficient of Variation	Min	Lower Quartile	Median	Upper Quartile	Max
> 65 years of age:	4,3	4,0	93,3 %	0,0	2,5	5,0	6,5	8,0

Is there a need for hiring of replacement /expanding the number of chemical risk assessment/communication personnel over the next 5-10 years in your organization, e.g. due to retirement or change in duties/deliverables within your organisation?

Is there a need for hiring of replacement /expanding the number of chemical risk assessment/communication personnel over the next 5-10 years in your organization, e.g. due to retirement or change in duties/deliverables within your organisation?	Number of responses
YES	7 (87,5%)
NO	1 (12,5%)
Total	8 (100,0%)

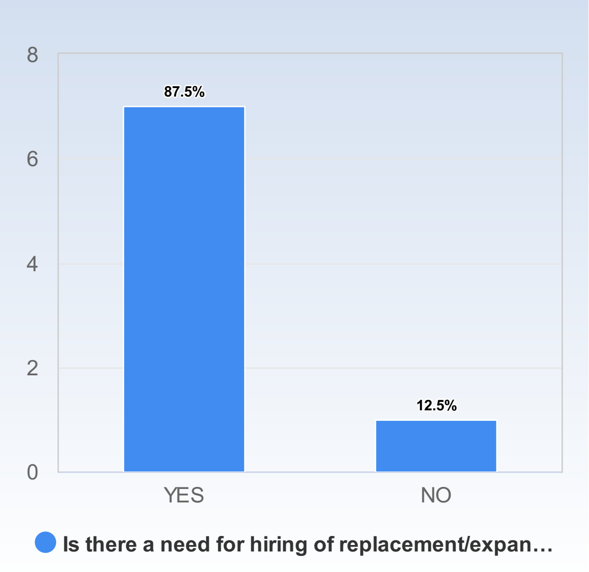

	Mean	Standard Deviation	Coefficient of Variation	Min	Lower Quartile	Median	Upper Quartile	Max
Is there a need for hiring of replacement/expanding the number of chemical risk assessment/communication personnel over the next 5-10 years in your organization, e.g. due to retirement or change in duties/deliverables within your organisation?	1,1	0,4	31,4 %	1,0	1,0	1,0	1,0	2,0

Please comment
If possible we would have had two persons with this type of skills in our organisation, but that is not possible to get - there are none in this area.
retirements. However, future need is hard to decide, depending on tasks of the organization
We wil have some people that will retire. Possible that other people find positions outside the department, including managerial roles
We need new expertice and replace / strengthen existing areas of risk assessment within the department.
Retirement and change in deliverables

Which needs do you forsee you will have recruiting relevant personnel for the coming 4 - 7 years? Please address your needs for personnel with general toxicology and related science educations, versus highly specified education.

Which needs do you forsee you will have recruiting relevant personnel for the coming 4 - 7 years? Please address your needs for personnel with general toxicology and related science educations, versus highly specified education.
We will hire one more risk assessor preferably with a Master in risk assessment. we will also try to hire a person with skills within ecotoxicology/environmental management system skills. Due to retirement a general toxicologist will also be needed in about 7 years.
chemistry, risk assessments, veterinary toxicology, feed toxicology, poisonings
We expect that we have 6-8 vacancies. We expect general Toxicology training and thereafter spesialisation in a specific area of toxicology like inhalation, genetic, immunotoxicology and exposure sciences
General toxicology, computer science, exposure assessment, statistics, and NAMs
Regulatory toxicologist with competence in bioinformatics, new generation risk assessment methodologies
The department will need both personal with general toxicology with PhD degree, and more highly specialized personnel, especially within new methods (in vivo/in silico), bioinformatics, exposure assessments and environmental epidemiology. Areas as endocrine, immune and neurologic effects will also be areas of interest for the future.
Both, general toxicology/related science education preferably young recruits which are interessted to become specialised in our field and also persons with highly specified education which can step in, continue and further develop our already started work.
No needs

Please indicate your experience related to how easy it is to recruit competencies in chemical risk assessment/ communication you want to have /hire. Please use a number between 1 (easy to recruit) and 6 (difficult to recruit).

Please indicate your experience related to how easy it is to recruit competencies in chemical risk assessment/ communication you want to have /hire. Please use a number between 1 (easy to recruit) and 6 (difficult to recruit).	Number of responses
1. Easy to recruit	0 (0,0%)
2.	0 (0,0%)
3.	0 (0,0%)
4.	2 (25,0%)
5.	1 (12,5%)
6. Difficult to recruit	5 (62,5%)
Total	8 (100,0%)

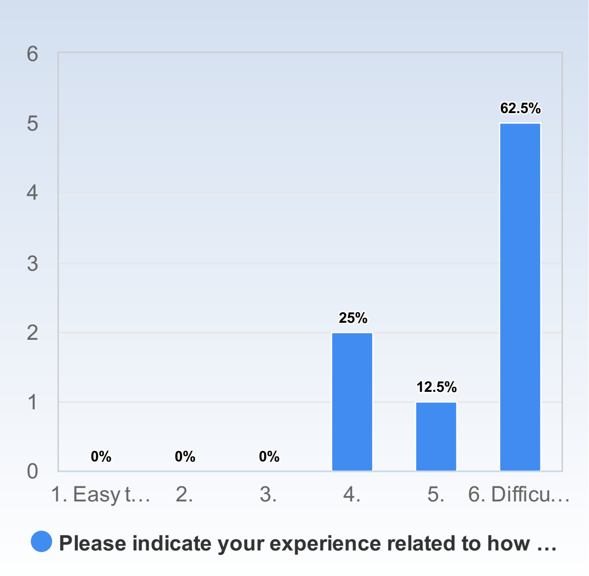

	Mean	Standard Deviation	Coefficient of Variation	Min	Lower Quartile	Median	Upper Quartile	Max
Please indicate your experience related to how easy it is to recruit competencies in chemical risk assessment/ communication you want to have /hire. Please use a number between 1 (easy to recruit) and 6 (difficult to recruit).	5,4	0,9	17,0 %	4,0	4,5	6,0	6,0	6,0

# Indicate if academia delivers sufficient number of candidates to fulfill your needs (master and/or PhD level)?

Indicate if academia delivers sufficient number of candidates to fulfill your needs (master and/or PhD level)?
We are not located in a central area around Oslo or Trondhjem, meaning that there are very few people with relevant background (master or higher) within human- and eco-toxicology. Local process industry would have hired more toxicologists had it been possible to find them...
very limited in feed sceience, veterinary toxicology, etc. limited in pathology,
On a master level there are a number of candidates but very much ecotoxicology focussed and not a lot with a human toxicology focus. PhD are much more difficult to recruit since there are not enough funded PhD positions available. Universities focus more on ecotoxicology than human toxicology
No
Not for human toxicologists
There is a growing need for personal within risk assessment with PhD degree. We are very concerned for future possibilities for employing toxicologists with required expertise/skills.
There is not a sufficient number of candidates available, both at Master and Phd level.
Yes

Do you find it necessary to train your new personnel in the areas of chemical risk assessment/communication due to limited/poor knowledge from academia or previous affiliation (s)/work experience?

Do you find it necessary to train your new personnel in the areas of chemical risk assessment/communication due to limited/poor knowledge from academia or previous affiliation(s) /work experience?	Number of responses
YES	7 (87,5%)
NO	1 (12,5%)
Total	8 (100,0%)

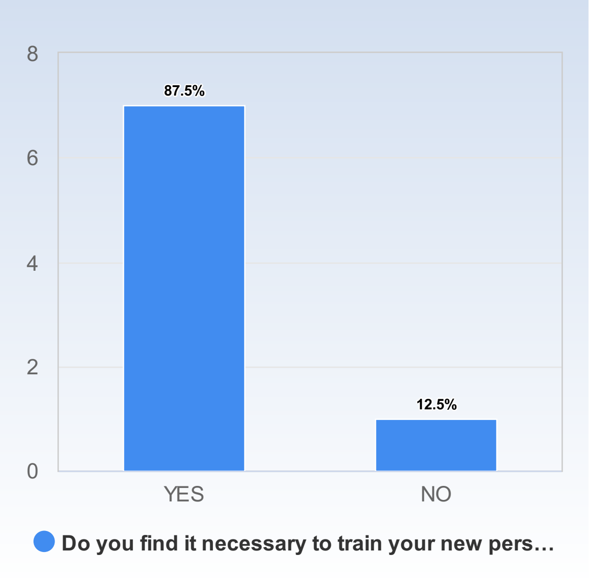

	Mean	Standard Deviation	Coefficient of Variation	Min	Lower Quartile	Median	Upper Quartile	Max
Do you find it necessary to train your new personnel in the areas of chemical risk assessment/communication due to limited/poor knowledge from academia or previous affiliation(s)/work experience?	1,1	0,4	31,4 %	1,0	1,0	1,0	1,0	2,0

If YES, please comment on how you train the personnel.
Yes, but it is only possible to a certain degree. The need for solid academic background within toxicology, ecotoxicology or biochemistry is almost a requirement to succeed.
On the job training being activite in the consultancy work and in research projects
Relevant workshops, courses
We put them under trainiship of senior personnel
Yes, new employs will be included in risk assessment teams and trained by experienced personal in the department. In addition, it is important that all personal participate in relevant courses and conferences.
Internal seminars, literature studies, participation on conferences/workshops

Which areas of expertise areas are primarily lacking when you want to hire new personnel?

Which areas of expertise areas are primarily lacking when you want to hire new personnel?	Number of responses
Alternative (non-animal) in vitro methods	4 (57,1%)
Animal testing	3 (42,9%)
Bioinformatics	4 (57,1%)
Chemical analysis	3 (42,9%)
Chemistry/Environmental chemistry	2 (28,6%)
Ecotoxicology	1 (14,3%)
Epidemiology	1 (14,3%)
Exposure assessment	4 (57,1%)
QSAR and read-across	5 (71,4%)
Risk assessment	2 (28,6%)
Risk communication	0 (0,0%)
Risk management	0 (0,0%)
Statistics	3 (42,9%)
Systematic literature reviews	0 (0,0%)
Toxicology	6 (85,7%)
Other (please comment below)	0 (0,0%)
Total	38 (542,9%)

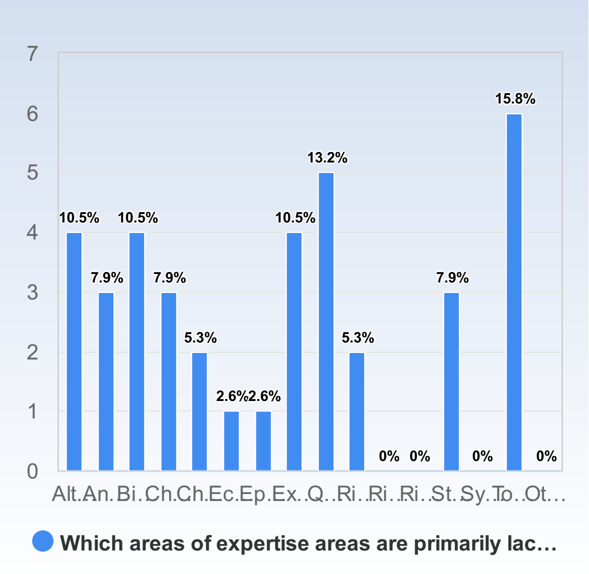

	Mean	Standard Deviation	Coefficient of Variation	Min	Lower Quartile	Median	Upper Quartile	Max
Which areas of expertise areas are primarily lacking when you want to hire new personnel?	18,4	4,8	25,9 %	12,0	14,0	19,0	21,0	26,0

Comment
We are going to need personell with toxicology background, that also have specialised in the areas noted above
None

Please suggest university courses related to chemical risk assessment/communication you would like to see offered by academia in a near future.

Please suggest university courses related to chemical risk assessment/communication you would like to see offered by academia in a near future.
Basic toxicology both within human and eco. Regulatory toxicology.
More focus on regulatory framework and toxicologists with bioinformatics and biostatistics skills. More hands-on experience with advanced laboratory models including in vitro, transcriptomics and proteomic data
MSc in risk assessment
Human toxicology, OMICS
- New methods in risk assessment (in vitro/in silico)<br>- exposure assessment
case related courses in order to train the students for future real life scenarios

Please indicate, if you expect a lack of expertise in the area of risk assessment/ communication in your organisation, in the near future.

Please indicate, if you expect a lack of expertise in the area of risk assessment/ communication in your organisation, in the near future.	Number of responses
1. Not expected	1 (12,5%)
2.	0 (0,0%)
3.	1 (12,5%)
4.	2 (25,0%)
5.	1 (12,5%)
6. Definitely expected	3 (37,5%)
Total	8 (100,0%)

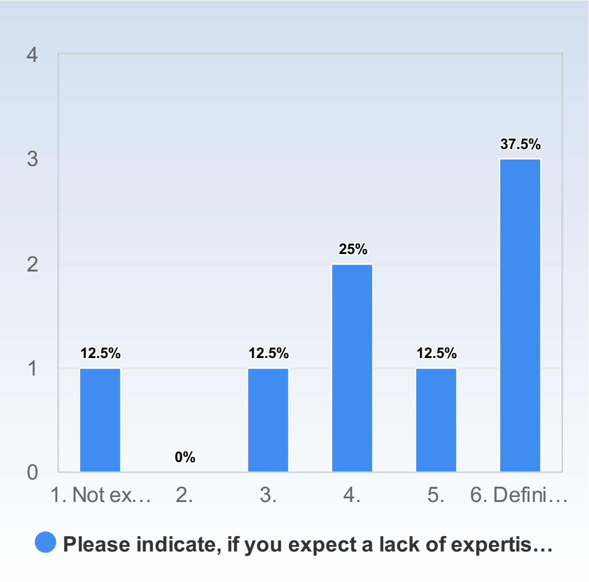

	Mean	Standard Deviation	Coefficient of Variation	Min	Lower Quartile	Median	Upper Quartile	Max
Please indicate, if you expect a lack of expertise in the area of risk assessment/ communication in your organisation, in the near future.	4,4	1,8	40,4 %	1,0	3,5	4,5	6,0	6,0

Please explain how you foresee to meet the needs of highly competent/qualified personnel in chemical risk assessment/communication in your organization five years from now (e.g. external courses, workshops or internal courses).

Please explain how you foresee to meet the needs of highly competent/qualified personnel in chemical risk assessment/communication in your organization five years from now (e.g. external courses, workshops or internal courses).
Use of international consultancies.
recruitment, external courses, internal training in suspected animal poisonings,
Combination of external courses and internal workshops. Active participation in a number of EU projects might offer training opportunities
Participating in workshops, courses, internal training. Maybe participation in the EU-FORA fellowship
External course, internal courses and internal courses as well as practical training and internship
Yes, new employs will be trained by experienced personal and participate in relevant courses and conferences.
external courses and workshops and also internal seminars/discussions
External courses, sertification

Please give suggestions what can be done nationally to optimize the numbers of competent persons for your organization.

Please give suggestions what can be done nationally to optimize the numbers of competent persons for your organization.
Basic and specialised toxicology should be strengthened at Universities. Also in terms of finacial support to PhDs or post docs. Belgium, Germany, France, Uk all have consultancy companies with highly qualified toxicologists - often with different people specialising in different areas like litterature review, follow up on animal testing, exposure assessments, QSAR, ecotox, epidemiology etc. We don't have any of this in Norway. It is some scattered knowledge within a few organisations like DNV and so on, but they aren't big enough to provide broad enough support. We are always dependent on international support. It is sad that we can't get that type of support in Norway. And for advanced chemical process industry outside Oslo, it is very difficult to find people with sufficient academic background within this field.
Toxicology training in a One Health perspective, collected compendium related to animal poisoning, causes and symptoms for poisonings,
Specific training program to recruit young toxicologists into the area of human toxicology. The research council should award some research projects within human toxicology so that we can hire PhD students and postdocs
Increas the focus on human toxicology and methods for risk assessment. This should also be combined with methods for systematic review and evidence-based toxicology thinking
Education of human toxicologists at the university level, provision of university courses in human toxicology
We need more national funded research within human health and toxicology to ensure PhD candidates for future toxicology science and advisory work. More programs with focus on human health and toxicology issues in The Research Council of Norway is highly essential.
Recruitment and academic education of young people to guarantee a high enough number of competent persons in the future. Starting recruitment already at school level by demonstrating how interessing, useful, exiting and necessary this specific work is. Publishing more topic related articles in social media as e.g. news papers, to educate the general population and to catch attention and interesst of especially younger people who are at a stage where deciding about their porfessional future pathway.
No suggestions, have no needs

Please suggest how the Nordic countries could act jointly to optimize the numbers of competent persons for your organization.

Please suggest how the Nordic countries could act **jointly** to optimize the numbers of competent persons for your organization.
Nordic training and network, related to veterinary toxicology as the total need is limited,
Organise training courses (3-4 days) that focus on specfic areas. On-line courses that people can take in their own time.<br>A Nordic PhD program with research projects
Nordic training in relevant topics
Setting up common courses with common sylabus
Arranging joint training programs for the countries will be a valuable initiative.
See also 16)<br>Education of compentent persons on a Nordic level by arranging Nordic courses/workshops for people who already have a relevant basic education - further development - and academic courses/schools for people beeing in an earlier career stage (as students or PhD-candidates).
No suggestions, have no needs

Do you see positive possibilities for a closer, formalized cooperation between the Nordic countries in the area of chemical risk analysis, including training?

Do you see positive possibilities for a closer, formalized cooperation between the Nordic countries in the area of chemical risk analysis, including training?	Number of responses
YES	7 (100,0%)
NO	0 (0,0%)
Total	7 (100,0%)

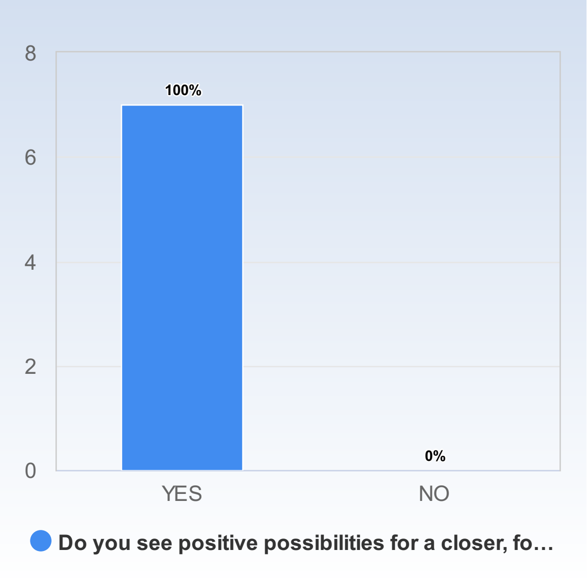

	Mean	Standard Deviation	Coefficient of Variation	Min	Lower Quartile	Median	Upper Quartile	Max
Do you see positive possibilities for a closer, formalized cooperation between the Nordic countries in the area of chemical risk analysis, including training?	1,0	0,0	0,0 %	1,0	1,0	1,0	1,0	1,0

If YES - How could this cooperation be accomplished? / If NO - Why would that be hard to accomplish?
Don't know
Nordic council should put some money on the table to develop training courses of sponsor PhD projects in the area of human toxicology
For subjects that are of interest for risk managers in more nordic countries, it is better to collaborate than to perform separate assessment
Collabortaion of toxicology societies, closer contact between regulatory authorities
Nordic workshops, courses, meetings organised by national/nordic authorities and together with e.g. national toxicological/chemical societies
Courses/workshops with different hosts in the Nordic countries.

How many of your experts within chemical risk assessment/communication are involved in international assignments related to the organizations indicated below?

EFSA panels:

EFSA panels:	Number of responses
0	1 (33,3%)
1	0 (0,0%)
2	0 (0,0%)
3	1 (33,3%)
4	1 (33,3%)
5	0 (0,0%)
6	0 (0,0%)
7	0 (0,0%)
8	0 (0,0%)
9	0 (0,0%)
Total	3 (100,0%)

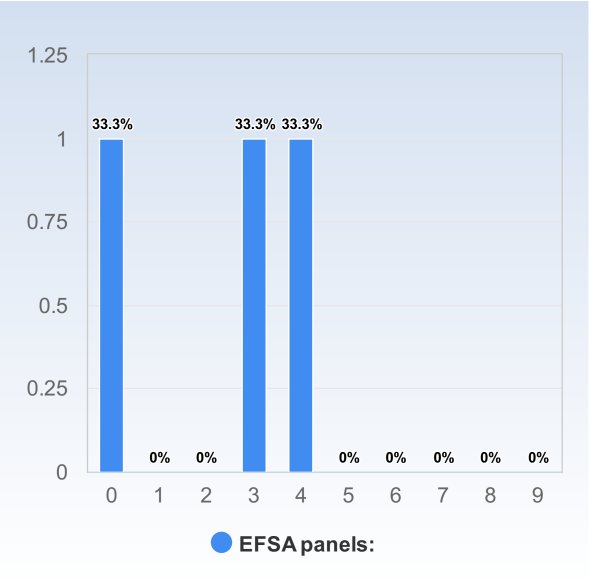

	Mean	Standard Deviation	Coefficient of Variation	Min	Lower Quartile	Median	Upper Quartile	Max
EFSA panels:	2,3	2,1	89,2 %	0,0	1,5	3,0	3,5	4,0

ECHA expert groups and member state committee:

ECHA expert groups and member state committee:	Number of responses
0	1 (33,3%)
1	0 (0,0%)
2	0 (0,0%)
3	1 (33,3%)
4	0 (0,0%)
5	0 (0,0%)
6	1 (33,3%)
7	0 (0,0%)
8	0 (0,0%)
9	0 (0,0%)
Total	3 (100,0%)

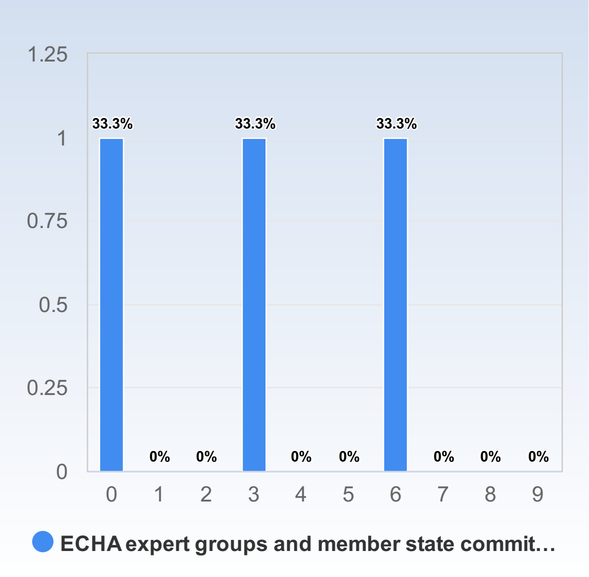

	Mean	Standard Deviation	Coefficient of Variation	Min	Lower Quartile	Median	Upper Quartile	Max
ECHA expert groups and member state committee:	3,0	3,0	100,0 %	0,0	1,5	3,0	4,5	6,0

EC scientific committees:

EC scientific committees:	Number of responses
0	1 (100,0%)
1	0 (0,0%)
2	0 (0,0%)
3	0 (0,0%)
4	0 (0,0%)
5	0 (0,0%)
6	0 (0,0%)
7	0 (0,0%)
8	0 (0,0%)
9	0 (0,0%)
Total	1 (100,0%)

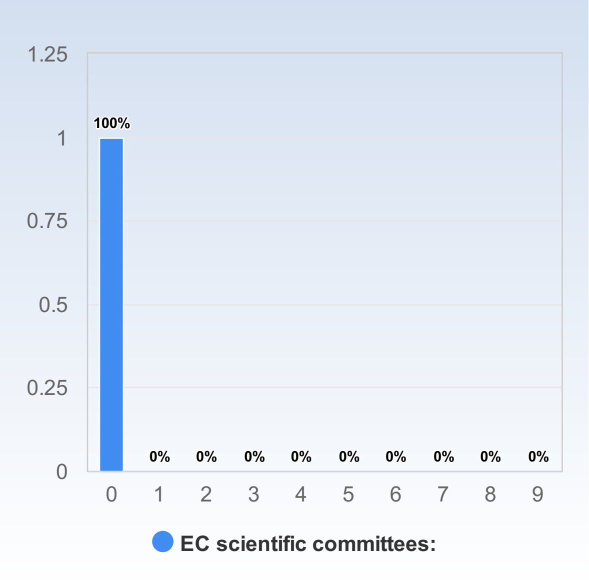

	Mean	Standard Deviation	Coefficient of Variation	Min	Lower Quartile	Median	Upper Quartile	Max
EC scientific committees:	0,0	0,0	NaN %	0,0	0,0	0,0	0,0	0,0

EMA committees or working groups:

EMA committees or working groups:	Number of responses
-2147483648	0 (0,0%)
-2147483647	0 (0,0%)
-2147483646	0 (0,0%)
-2147483645	0 (0,0%)
-2147483644	0 (0,0%)
-2147483643	0 (0,0%)
-2147483642	0 (0,0%)
-2147483641	0 (0,0%)
-2147483640	0 (0,0%)
-2147483639	0 (0,0%)
Total	0 (0,0%)

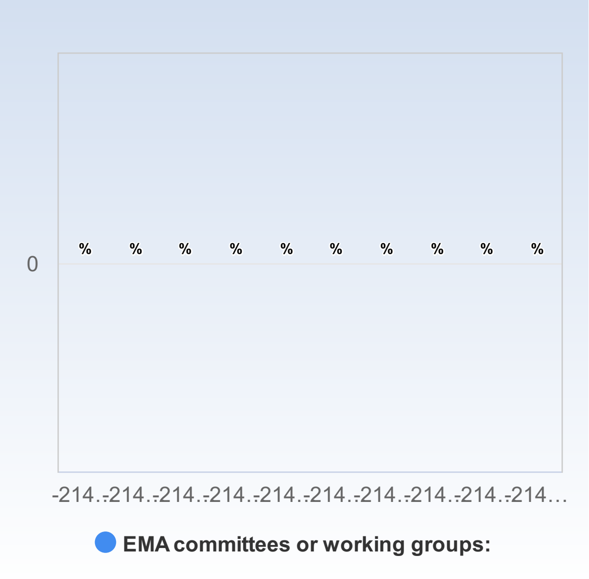

	Mean	Standard Deviation	Coefficient of Variation	Min	Lower Quartile	Median	Upper Quartile	Max
EMA committees or working groups:	0,0	0,0	NaN %	∞	0,0	0,0	0,0	-∞

OECD working groups:

OECD working groups:	Number of responses
0	0 (0,0%)
1	2 (100,0%)
2	0 (0,0%)
3	0 (0,0%)
4	0 (0,0%)
5	0 (0,0%)
6	0 (0,0%)
7	0 (0,0%)
8	0 (0,0%)
9	0 (0,0%)
Total	2 (100,0%)

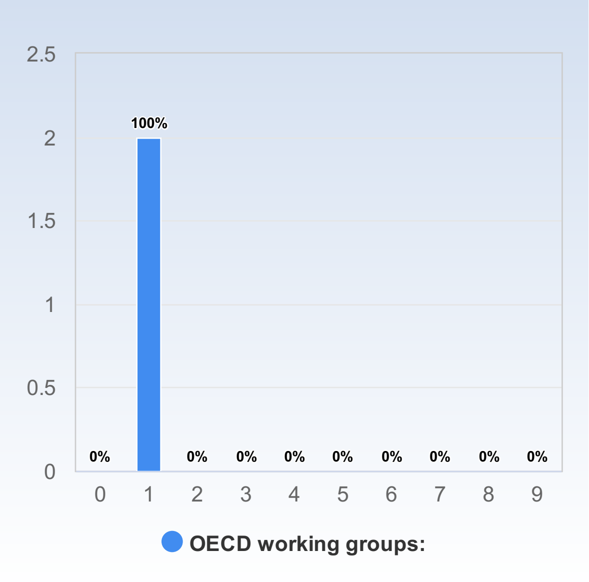

	Mean	Standard Deviation	Coefficient of Variation	Min	Lower Quartile	Median	Upper Quartile	Max
OECD working groups:	1,0	0,0	0,0 %	1,0	1,0	1,0	1,0	1,0

WHO expert groups:

WHO expert groups:	Number of responses
0	1 (50,0%)
1	1 (50,0%)
2	0 (0,0%)
3	0 (0,0%)
4	0 (0,0%)
5	0 (0,0%)
6	0 (0,0%)
7	0 (0,0%)
8	0 (0,0%)
9	0 (0,0%)
Total	2 (100,0%)

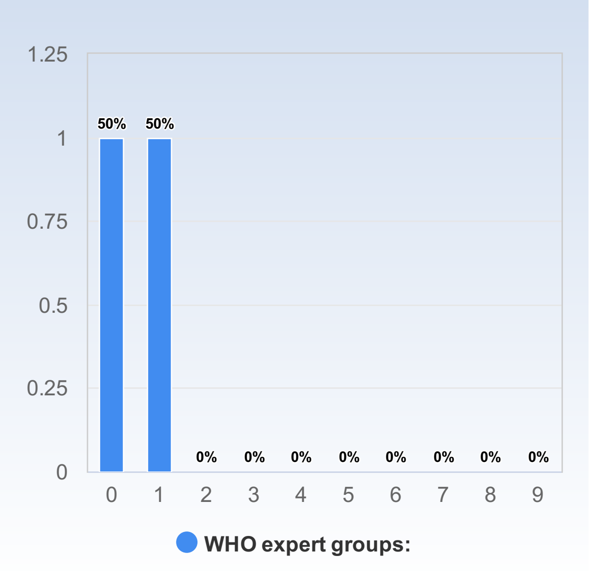

	Mean	Standard Deviation	Coefficient of Variation	Min	Lower Quartile	Median	Upper Quartile	Max
WHO expert groups:	0,5	0,7	141,4 %	0,0	0,5	0,5	0,5	1,0

Other(s) (please comment below)

Other(s) (please comment below)	Number of responses
0	0 (0,0%)
1	2 (66,7%)
2	1 (33,3%)
3	0 (0,0%)
4	0 (0,0%)
5	0 (0,0%)
6	0 (0,0%)
7	0 (0,0%)
8	0 (0,0%)
9	0 (0,0%)
Total	3 (100,0%)

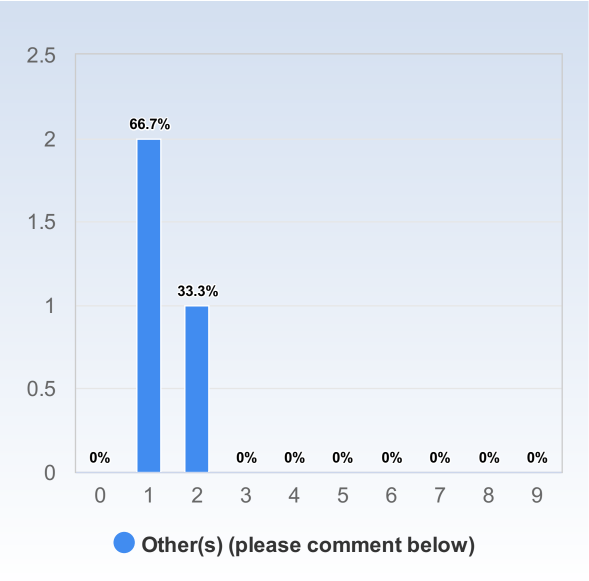

	Mean	Standard Deviation	Coefficient of Variation	Min	Lower Quartile	Median	Upper Quartile	Max
Other(s) (please comment below)	1,3	0,6	43,3 %	1,0	1,0	1,0	1,5	2,0

Comment
I am involved in technical working groups for several Reach consortia. I have also been chair for several Reach consortia work groups internationally. I have also represented industry in relevant industry organisations like Eurometaux and CEFIC.
previously activly participated in relevant EFSA Working groups, EC scientific committees, Participates in reference laboratory studies, and method developments and and ILSI Worg group on food allergens
IARC
NEG
None of the above

Do you expect that the number of personnel involved in international assignments (c.f. previous question) will increase or decrease in the coming 5 years?

Do you expect that the number of personnel involved in international assignments (c.f. previous question) will increase or decrease in the coming 5 years?	Number of responses
Increase	5 (62,5%)
Decrease	0 (0,0%)
No change is expected	3 (37,5%)
Total	8 (100,0%)

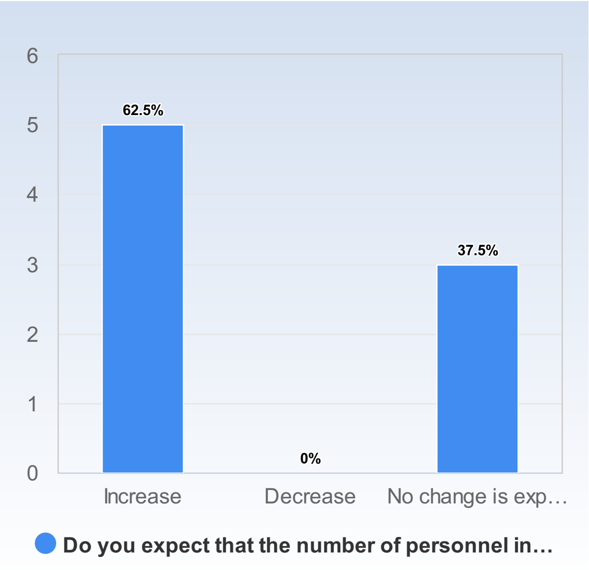

	Mean	Standard Deviation	Coefficient of Variation	Min	Lower Quartile	Median	Upper Quartile	Max
Do you expect that the number of personnel involved in international assignments (c.f. previous question) will increase or decrease in the coming 5 years?	1,8	1,0	59,1 %	1,0	1,0	1,0	3,0	3,0

Please comment.
Increase because there will a high demand of these specialised experts. But not sure if we can prioritise participation in these international groups above national tasks
Togeter with climate changes, biodiversity and pollution/chemical exposure is a global challenge, and international cooperation is higly nessesary.

Any additional reflections or comments regarding competence provision needs in the area of risk assessment/communication?

Any additional reflections or comments regarding competence provision needs in the area of risk assessment/communication?
It is timely that this is put on the Nordic agenda!

SI Part F

System generated analyses of the result from the Swedish respondents

Risk analysis competence provision questionnaire for Sweden

Respondents: 74
Answer Count: 12
Answer Frequency: 16,22 %

Please indicate your area of affiliation? (mandatory)

Please indicate your area of affiliation? (mandatory)	Number of responses
Research institute	1 (8,3%)
National authority	3 (25,0%)
Regional authority	0 (0,0%)
Hospital practice	0 (0,0%)
Industry/Business	4 (33,3%)
NGO	1 (8,3%)
Consultant	2 (16,7%)
Other	1 (8,3%)
Total	12 (100,0%)

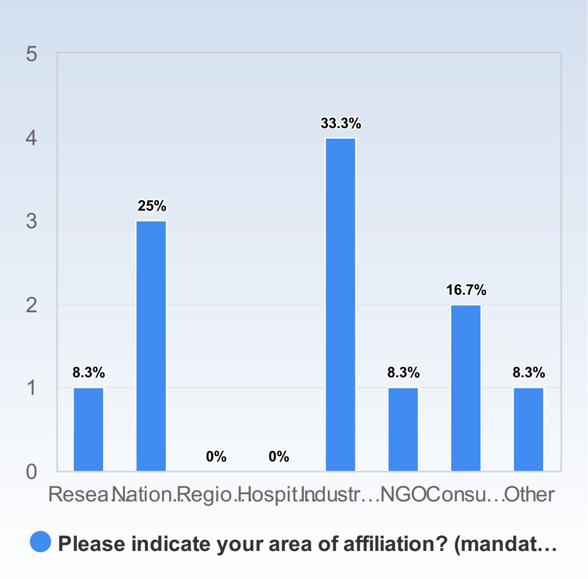

	Mean	Standard Deviation	Coefficient of Variation	Min	Lower Quartile	Median	Upper Quartile	Max
Please indicate your area of affiliation? (mandatory)	5,9	2,7	45,8 %	2,0	3,0	6,0	8,5	10,0

What is the number of personnel in chemical risk assessment/risk communication in your organization?

What is the number of personnel in chemical risk assessment/risk communication in your organization?	Number of responses
0 - 10	7 (63,6%)
11 - 21	3 (27,3%)
22 - 32	1 (9,1%)
33 - 43	0 (0,0%)
44 - 54	0 (0,0%)
55 - 65	0 (0,0%)
66 - 76	0 (0,0%)
77 - 87	0 (0,0%)
88 - 98	0 (0,0%)
99 - 109	0 (0,0%)
Total	11 (100,0%)

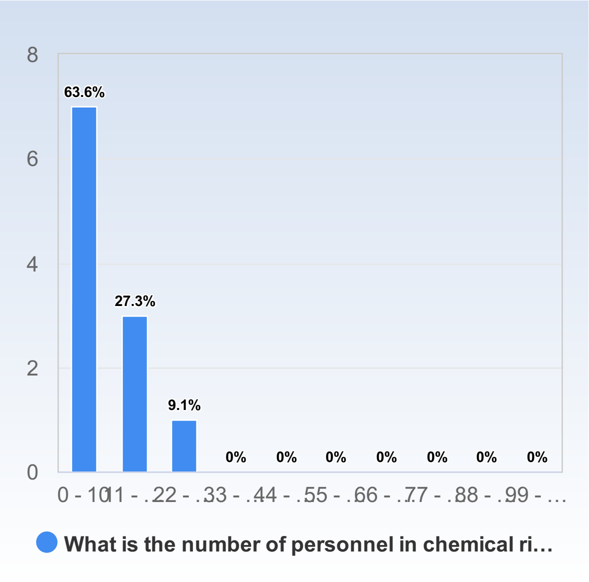

	Mean	Standard Deviation	Coefficient of Variation	Min	Lower Quartile	Median	Upper Quartile	Max
What is the number of personnel in chemical risk assessment/risk communication in your organization?	24,3	49,5	203,5 %	2,0	6,5	7,5	18,5	180,0

What specialization, in chemical risk assessment /communication, do you have in your organization?
Please estimate the number of personnel for each specialization.

Alternative (non-animal) in vitro methods

Alternative (non-animal) in vitro methods	Number of responses
0	0 (0,0%)
1	3 (60,0%)
2	0 (0,0%)
3	1 (20,0%)
4	0 (0,0%)
5	1 (20,0%)
6	0 (0,0%)
7	0 (0,0%)
8	0 (0,0%)
9	0 (0,0%)
Total	5 (100,0%)

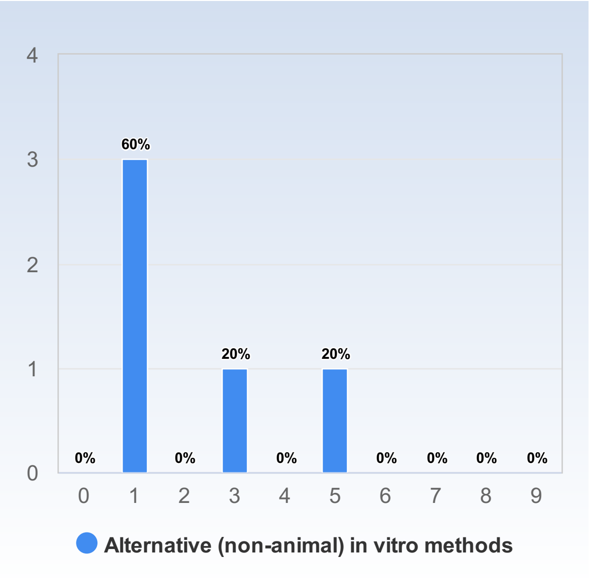

	Mean	Standard Deviation	Coefficient of Variation	Min	Lower Quartile	Median	Upper Quartile	Max
Alternative (non-animal) in vitro methods	2,2	1,8	81,3 %	1,0	1,0	1,0	3,0	5,0

Animal testing

Animal testing	Number of responses
0 - 1	3 (42,9%)
2 - 3	2 (28,6%)
4 - 5	0 (0,0%)
6 - 7	0 (0,0%)
8 - 9	1 (14,3%)
10 - 11	0 (0,0%)
12 - 13	0 (0,0%)
14 - 15	1 (14,3%)
16 - 17	0 (0,0%)
18 - 19	0 (0,0%)
Total	7 (100,0%)

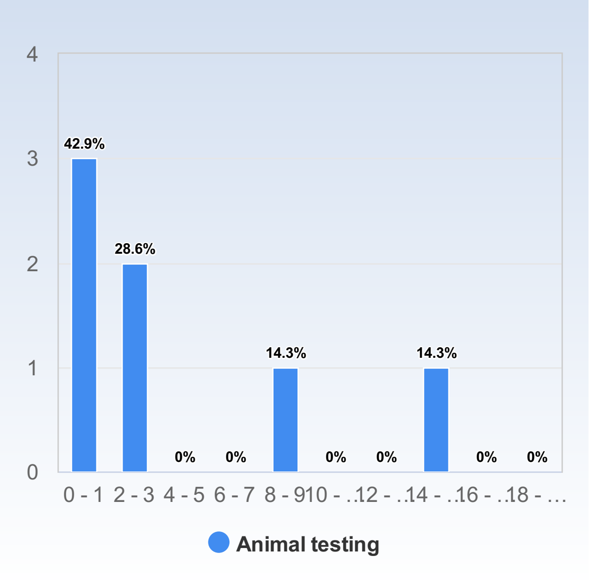

	Mean	Standard Deviation	Coefficient of Variation	Min	Lower Quartile	Median	Upper Quartile	Max
Animal testing	4,4	5,3	119,4 %	1,0	1,0	2,0	5,5	15,0

Bioinformatics

Bioinformatics	Number of responses
0	1 (33,3%)
1	2 (66,7%)
2	0 (0,0%)
3	0 (0,0%)
4	0 (0,0%)
5	0 (0,0%)
6	0 (0,0%)
7	0 (0,0%)
8	0 (0,0%)
9	0 (0,0%)
Total	3 (100,0%)

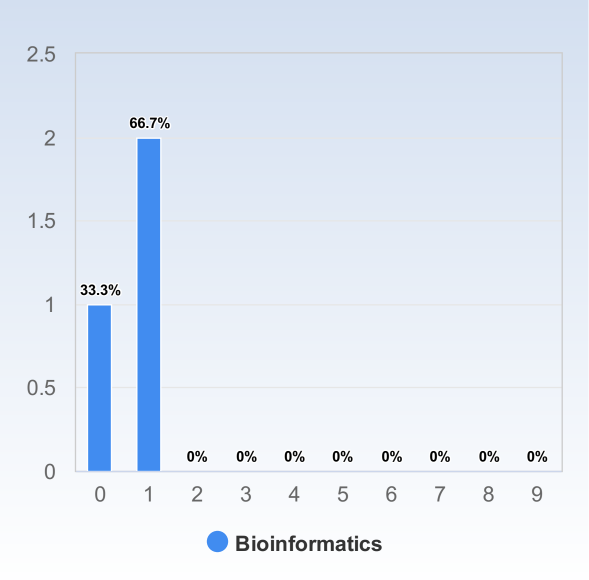

	Mean	Standard Deviation	Coefficient of Variation	Min	Lower Quartile	Median	Upper Quartile	Max
Bioinformatics	0,7	0,6	86,6 %	0,0	0,5	1,0	1,0	1,0

Chemical analysis

Chemical analysis	Number of responses
1	1 (14,3%)
2	2 (28,6%)
3	2 (28,6%)
4	1 (14,3%)
5	0 (0,0%)
6	0 (0,0%)
7	0 (0,0%)
8	0 (0,0%)
9	0 (0,0%)
10	1 (14,3%)
Total	7 (100,0%)

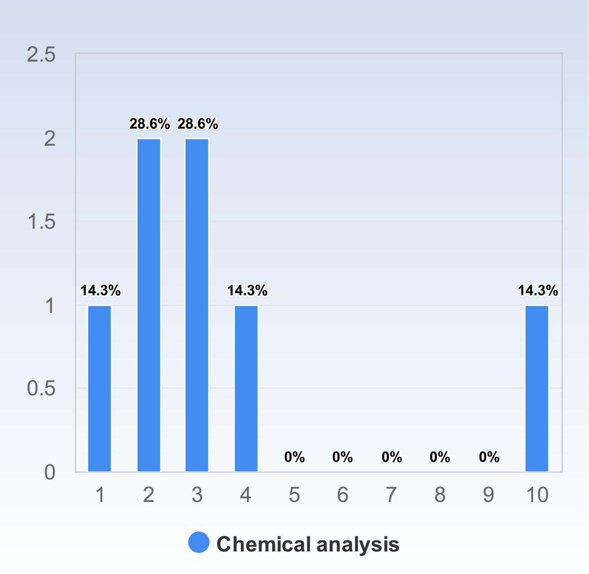

	Mean	Standard Deviation	Coefficient of Variation	Min	Lower Quartile	Median	Upper Quartile	Max
Chemical analysis	3,6	3,0	83,8 %	1,0	2,0	3,0	3,5	10,0

Chemistry/Environmental chemistry

Chemistry/Environmental chemistry	Number of responses
0 - 2	5 (50,0%)
3 - 5	3 (30,0%)
6 - 8	1 (10,0%)
9 - 11	0 (0,0%)
12 - 14	0 (0,0%)
15 - 17	0 (0,0%)
18 - 20	0 (0,0%)
21 - 23	0 (0,0%)
24 - 26	1 (10,0%)
27 - 29	0 (0,0%)
Total	10 (100,0%)

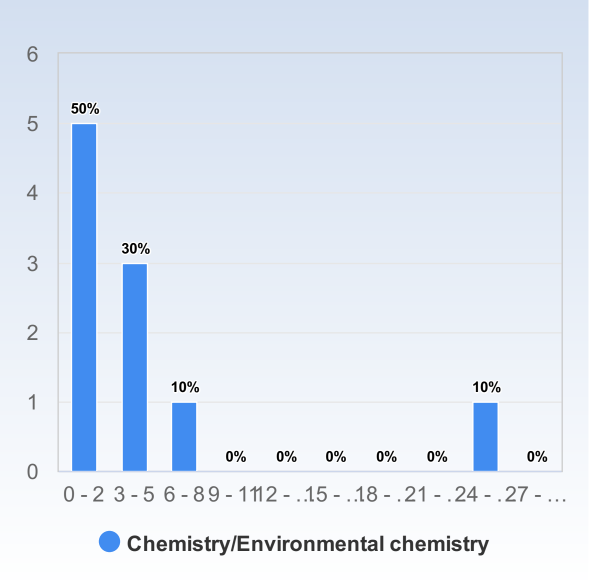

	Mean	Standard Deviation	Coefficient of Variation	Min	Lower Quartile	Median	Upper Quartile	Max
Chemistry/Environmental chemistry	5,2	7,2	138,3 %	1,0	2,0	3,0	4,0	25,0

Ecotoxicology

Ecotoxicology	Number of responses
0 - 2	7 (77,8%)
3 - 5	0 (0,0%)
6 - 8	1 (11,1%)
9 - 11	0 (0,0%)
12 - 14	0 (0,0%)
15 - 17	0 (0,0%)
18 - 20	0 (0,0%)
21 - 23	0 (0,0%)
24 - 26	1 (11,1%)
27 - 29	0 (0,0%)
Total	9 (100,0%)

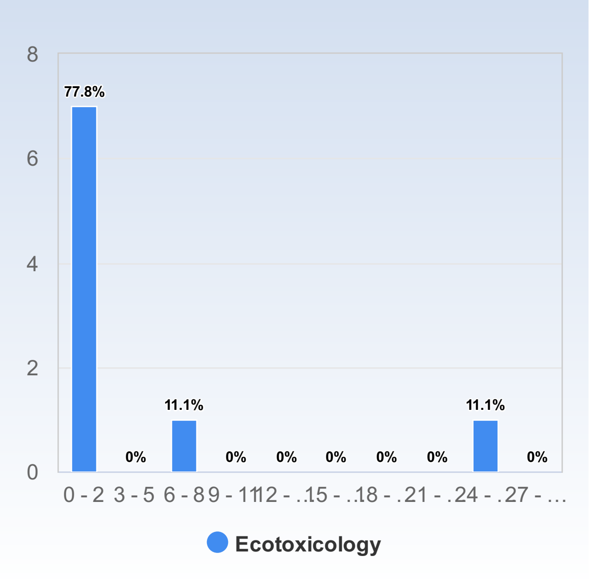

	Mean	Standard Deviation	Coefficient of Variation	Min	Lower Quartile	Median	Upper Quartile	Max
Ecotoxicology	4,8	7,7	161,7 %	1,0	2,0	2,0	2,0	25,0

Epidemiology

Epidemiology	Number of responses
0	0 (0,0%)
1	1 (33,3%)
2	2 (66,7%)
3	0 (0,0%)
4	0 (0,0%)
5	0 (0,0%)
6	0 (0,0%)
7	0 (0,0%)
8	0 (0,0%)
9	0 (0,0%)
Total	3 (100,0%)

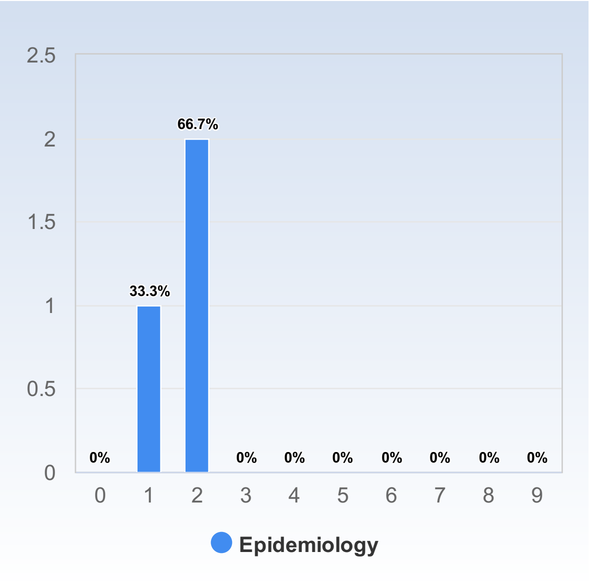

	Mean	Standard Deviation	Coefficient of Variation	Min	Lower Quartile	Median	Upper Quartile	Max
Epidemiology	1,7	0,6	34,6 %	1,0	1,5	2,0	2,0	2,0

Exposure assessment

Exposure assessment	Number of responses
0 - 1	1 (14,3%)
2 - 3	4 (57,1%)
4 - 5	1 (14,3%)
6 - 7	0 (0,0%)
8 - 9	0 (0,0%)
10 - 11	0 (0,0%)
12 - 13	0 (0,0%)
14 - 15	1 (14,3%)
16 - 17	0 (0,0%)
18 - 19	0 (0,0%)
Total	7 (100,0%)

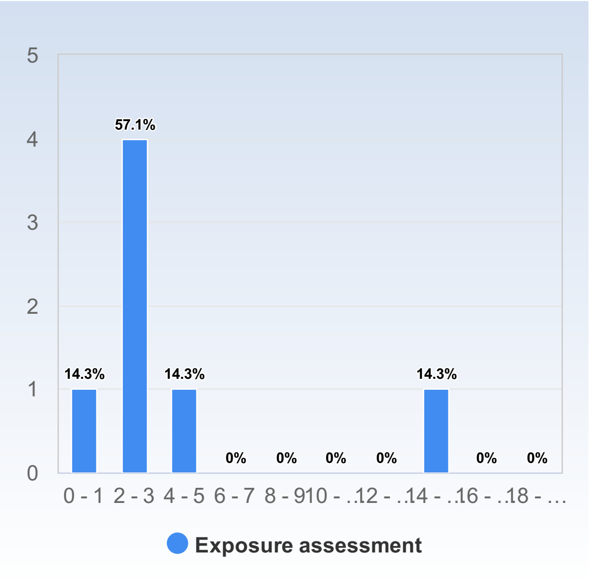

	Mean	Standard Deviation	Coefficient of Variation	Min	Lower Quartile	Median	Upper Quartile	Max
Exposure assessment	4,6	4,8	104,0 %	1,0	2,5	3,0	4,0	15,0

QSAR and read-across

QSAR and read-across	Number of responses
0	1 (14,3%)
1	1 (14,3%)
2	3 (42,9%)
3	0 (0,0%)
4	2 (28,6%)
5	0 (0,0%)
6	0 (0,0%)
7	0 (0,0%)
8	0 (0,0%)
9	0 (0,0%)
Total	7 (100,0%)

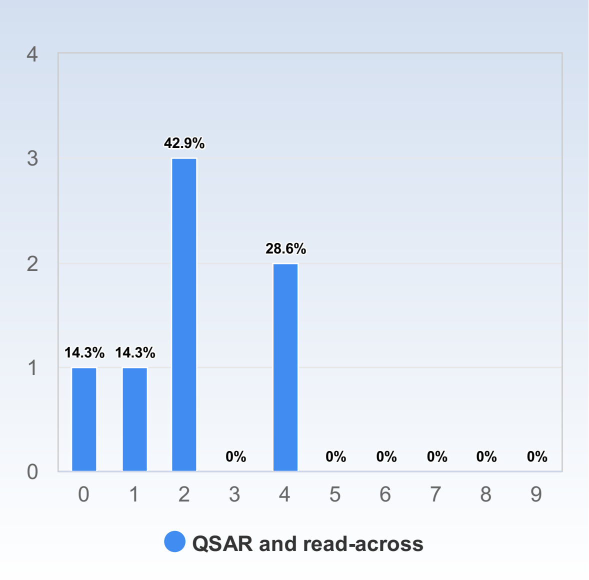

	Mean	Standard Deviation	Coefficient of Variation	Min	Lower Quartile	Median	Upper Quartile	Max
QSAR and read-across	2,1	1,5	68,3 %	0,0	1,5	2,0	3,0	4,0

Risk assessment

Risk assessment	Number of responses
0 - 3	2 (22,2%)
4 - 7	5 (55,6%)
8 - 11	0 (0,0%)
12 - 15	1 (11,1%)
16 - 19	0 (0,0%)
20 - 23	0 (0,0%)
24 - 27	0 (0,0%)
28 - 31	0 (0,0%)
32 - 35	1 (11,1%)
36 - 39	0 (0,0%)
Total	9 (100,0%)

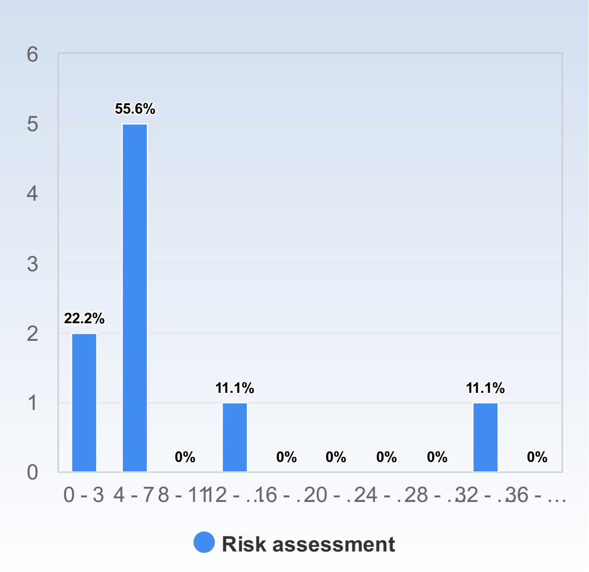

	Mean	Standard Deviation	Coefficient of Variation	Min	Lower Quartile	Median	Upper Quartile	Max
Risk assessment	8,8	9,8	111,2 %	2,0	4,0	6,0	7,0	33,0

Risk communication

Risk communication	Number of responses
2 - 3	1 (16,7%)
4 - 5	1 (16,7%)
6 - 7	2 (33,3%)
8 - 9	0 (0,0%)
10 - 11	0 (0,0%)
12 - 13	0 (0,0%)
14 - 15	0 (0,0%)
16 - 17	1 (16,7%)
18 - 19	0 (0,0%)
20 - 21	1 (16,7%)
Total	6 (100,0%)

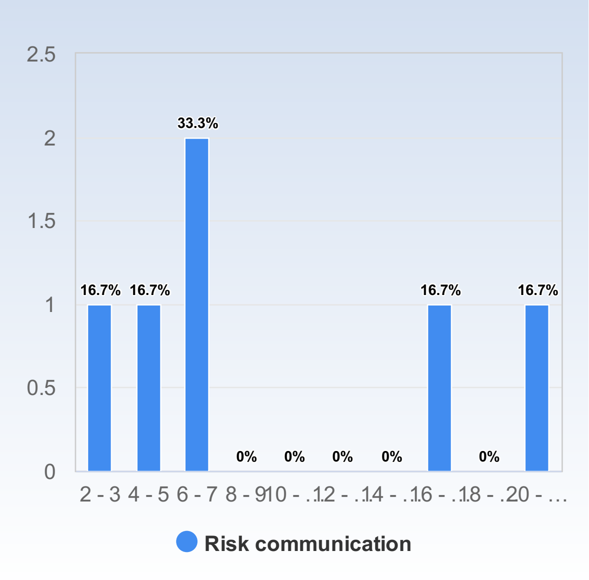

	Mean	Standard Deviation	Coefficient of Variation	Min	Lower Quartile	Median	Upper Quartile	Max
Risk communication	9,7	7,1	73,3 %	3,0	5,5	7,0	12,0	20,0

Risk management

Risk management	Number of responses
2 - 3	2 (28,6%)
4 - 5	1 (14,3%)
6 - 7	2 (28,6%)
8 - 9	0 (0,0%)
10 - 11	0 (0,0%)
12 - 13	0 (0,0%)
14 - 15	1 (14,3%)
16 - 17	0 (0,0%)
18 - 19	0 (0,0%)
20 - 21	1 (14,3%)
Total	7 (100,0%)

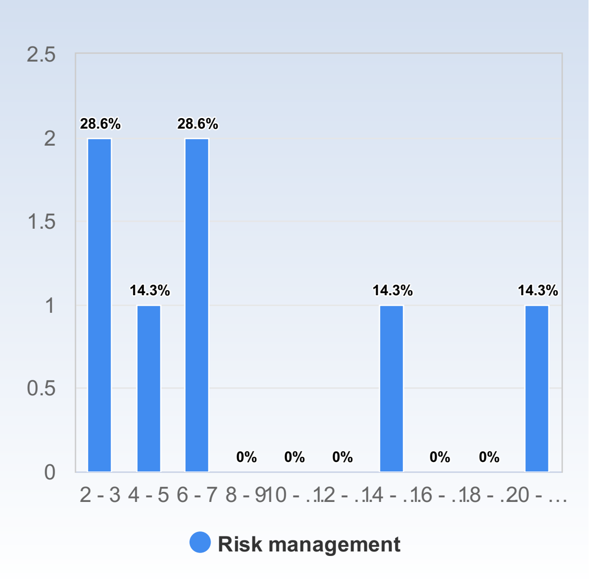

	Mean	Standard Deviation	Coefficient of Variation	Min	Lower Quartile	Median	Upper Quartile	Max
Risk management	8,1	6,9	84,3 %	2,0	3,0	7,0	11,0	20,0

Statistics

Statistics	Number of responses
0	0 (0,0%)
1	0 (0,0%)
2	0 (0,0%)
3	1 (33,3%)
4	1 (33,3%)
5	1 (33,3%)
6	0 (0,0%)
7	0 (0,0%)
8	0 (0,0%)
9	0 (0,0%)
Total	3 (100,0%)

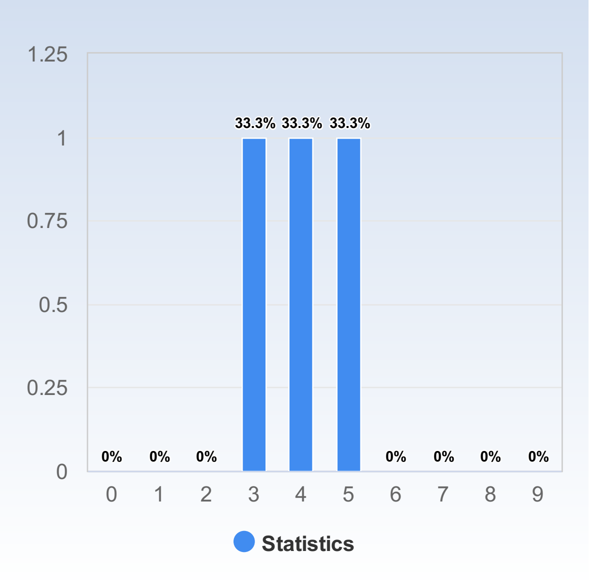

	Mean	Standard Deviation	Coefficient of Variation	Min	Lower Quartile	Median	Upper Quartile	Max
Statistics	4,0	1,0	25,0 %	3,0	3,5	4,0	4,5	5,0

Systematic literature reviews

Systematic literature reviews	Number of responses
0 - 3	2 (33,3%)
4 - 7	2 (33,3%)
8 - 11	1 (16,7%)
12 - 15	0 (0,0%)
16 - 19	0 (0,0%)
20 - 23	0 (0,0%)
24 - 27	0 (0,0%)
28 - 31	0 (0,0%)
32 - 35	0 (0,0%)
36 - 39	1 (16,7%)
Total	6 (100,0%)

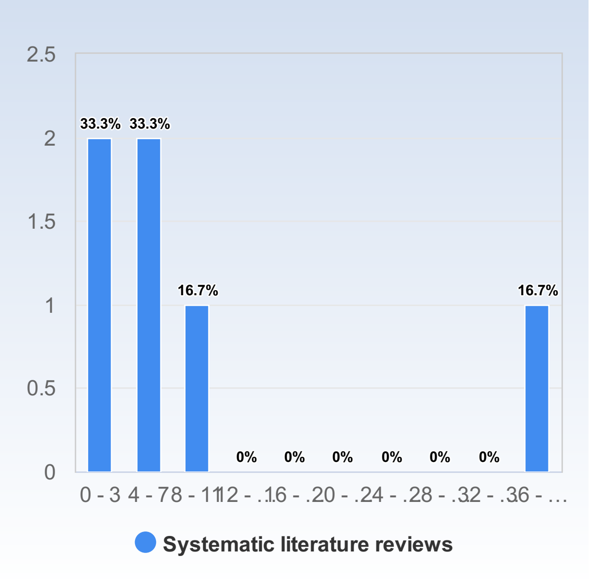

	Mean	Standard Deviation	Coefficient of Variation	Min	Lower Quartile	Median	Upper Quartile	Max
Systematic literature reviews	10,2	13,5	132,6 %	1,0	4,0	5,0	7,5	37,0

Toxicology

Toxicology	Number of responses
1 - 4	9 (81,8%)
5 - 8	0 (0,0%)
9 - 12	0 (0,0%)
13 - 16	0 (0,0%)
17 - 20	1 (9,1%)
21 - 24	0 (0,0%)
25 - 28	0 (0,0%)
29 - 32	0 (0,0%)
33 - 36	0 (0,0%)
37 - 40	1 (9,1%)
Total	11 (100,0%)

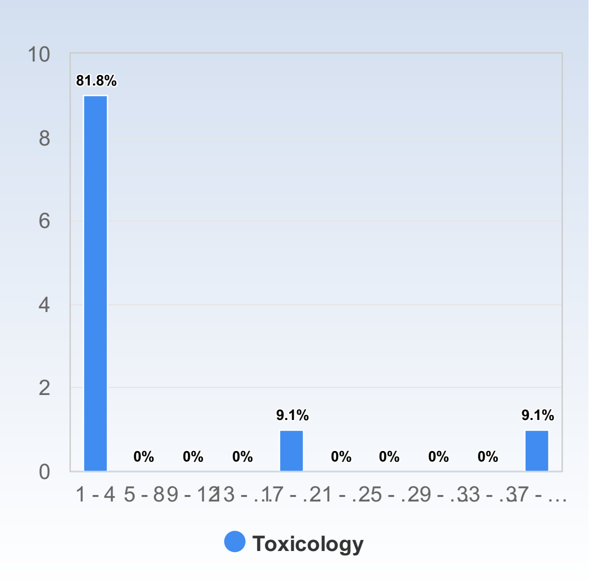

	Mean	Standard Deviation	Coefficient of Variation	Min	Lower Quartile	Median	Upper Quartile	Max
Toxicology	6,8	11,9	174,8 %	1,0	2,0	2,0	3,0	40,0

Other (please comment below)

Other (please comment below)	Number of responses
0	0 (0,0%)
1	0 (0,0%)
2	0 (0,0%)
3	1 (100,0%)
4	0 (0,0%)
5	0 (0,0%)
6	0 (0,0%)
7	0 (0,0%)
8	0 (0,0%)
9	0 (0,0%)
Total	1 (100,0%)

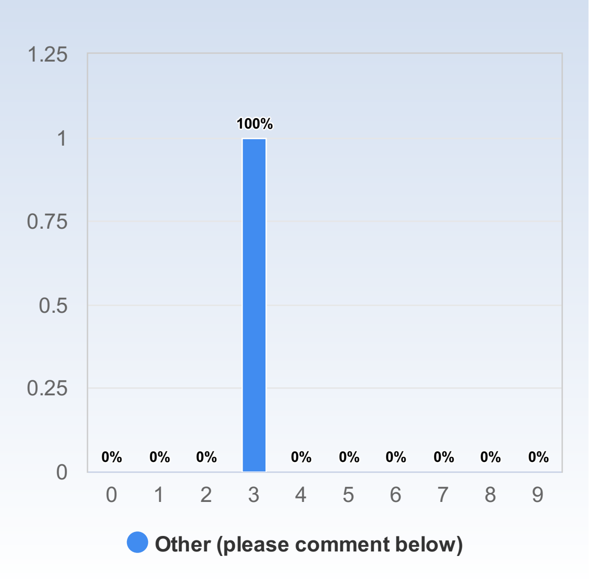

	Mean	Standard Deviation	Coefficient of Variation	Min	Lower Quartile	Median	Upper Quartile	Max
Other (please comment below)	3,0	0,0	NaN %	3,0	3,0	3,0	3,0	3,0

Comment
This is a new field in our industry and we are trying to make the best out of it.
Social-ek

How many of the personnel in your organization, with the chemical risk assessment /communication tasks you presented in the previous question, holds a:

Bachelor degree:

Bachelor degree:	Number of responses
0	1 (20,0%)
1	1 (20,0%)
2	2 (40,0%)
3	0 (0,0%)
4	0 (0,0%)
5	1 (20,0%)
6	0 (0,0%)
7	0 (0,0%)
8	0 (0,0%)
9	0 (0,0%)
Total	5 (100,0%)

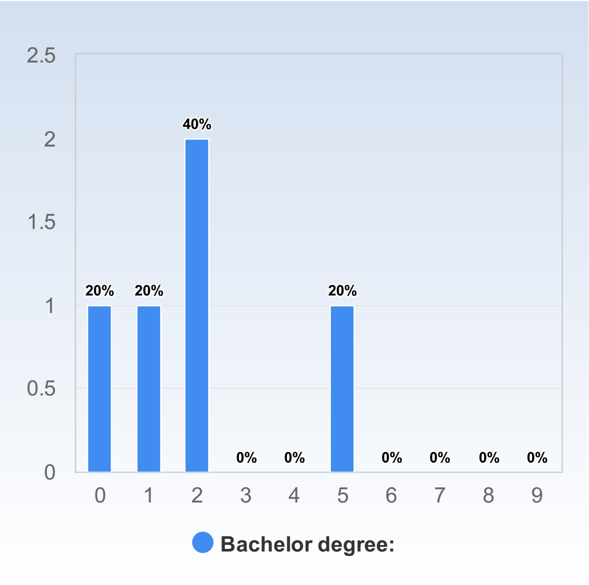

	Mean	Standard Deviation	Coefficient of Variation	Min	Lower Quartile	Median	Upper Quartile	Max
Bachelor degree:	2,0	1,9	93,5 %	0,0	1,0	2,0	2,0	5,0

Master degree:

Master degree:	Number of responses
1 - 6	8 (72,7%)
7 - 12	2 (18,2%)
13 - 18	0 (0,0%)
19 - 24	0 (0,0%)
25 - 30	0 (0,0%)
31 - 36	0 (0,0%)
37 - 42	0 (0,0%)
43 - 48	0 (0,0%)
49 - 54	0 (0,0%)
55 - 60	1 (9,1%)
Total	11 (100,0%)

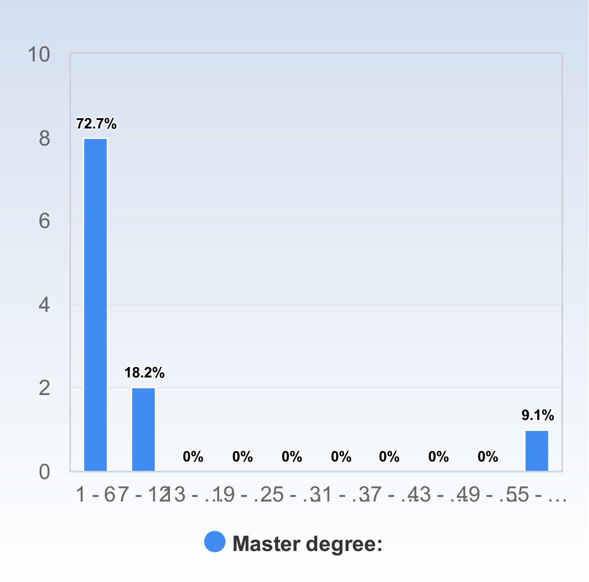

	Mean	Standard Deviation	Coefficient of Variation	Min	Lower Quartile	Median	Upper Quartile	Max
Master degree:	9,2	17,1	186,5 %	1,0	2,0	4,0	7,0	60,0

Doctoral degree (PhDs, MDs, Dtech (TkT, D.I.T), etc):

Doctoral degree (PhDs, MDs, Dtech (TkT, D.I.T), etc):	Number of responses
1 - 4	7 (58,3%)
5 - 8	3 (25,0%)
9 - 12	0 (0,0%)
13 - 16	1 (8,3%)
17 - 20	0 (0,0%)
21 - 24	0 (0,0%)
25 - 28	0 (0,0%)
29 - 32	0 (0,0%)
33 - 36	0 (0,0%)
37 - 40	1 (8,3%)
Total	12 (100,0%)

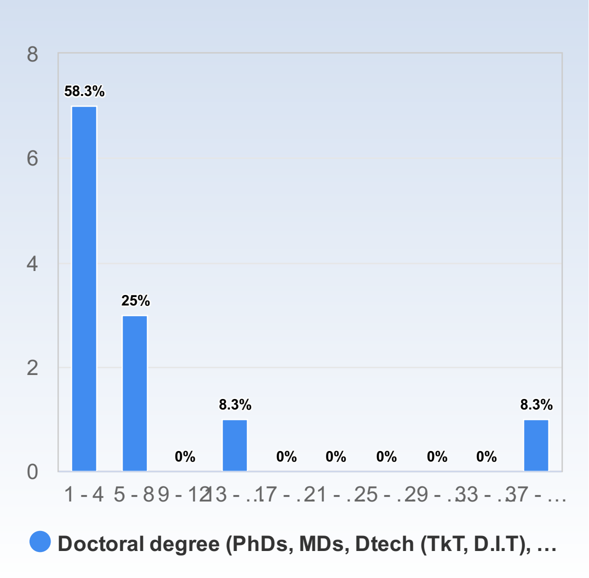

	Mean	Standard Deviation	Coefficient of Variation	Min	Lower Quartile	Median	Upper Quartile	Max
Doctoral degree (PhDs, MDs, Dtech (TkT, D.I.T), etc):	7,1	10,9	153,9 %	1,0	1,5	4,0	6,0	40,0

How many of your personnel are ERTs (European Registered Toxicologist)?

How many of your personnel are ERTs (European Registered Toxicologist)?	Number of responses
0 - 10	11 (100,0%)
11 - 21	0 (0,0%)
22 - 32	0 (0,0%)
33 - 43	0 (0,0%)
44 - 54	0 (0,0%)
55 - 65	0 (0,0%)
66 - 76	0 (0,0%)
77 - 87	0 (0,0%)
88 - 98	0 (0,0%)
99 - 109	0 (0,0%)
Total	11 (100,0%)

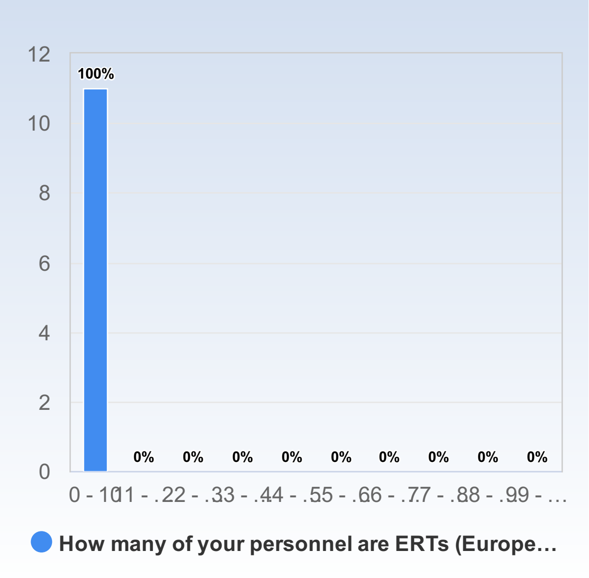

	Mean	Standard Deviation	Coefficient of Variation	Min	Lower Quartile	Median	Upper Quartile	Max
How many of your personnel are ERTs (European Registered Toxicologist)?	0,9	0,9	103,8 %	0,0	0,0	1,0	2,0	2,0

What is the present age profile of the risk analysis personnel in your organization?

< 40 years of age:

< 40 years of age:	Number of responses
0 - 4	7 (77,8%)
5 - 9	1 (11,1%)
10 - 14	0 (0,0%)
15 - 19	0 (0,0%)
20 - 24	0 (0,0%)
25 - 29	0 (0,0%)
30 - 34	0 (0,0%)
35 - 39	0 (0,0%)
40 - 44	1 (11,1%)
45 - 49	0 (0,0%)
Total	9 (100,0%)

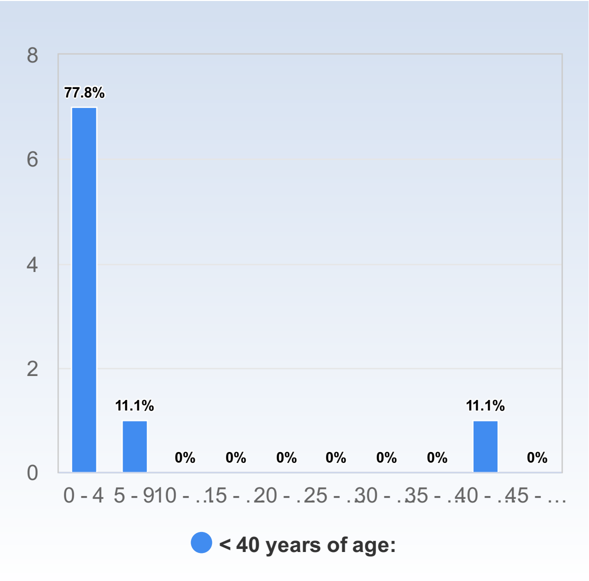

	Mean	Standard Deviation	Coefficient of Variation	Min	Lower Quartile	Median	Upper Quartile	Max
< 40 years of age:	6,6	13,4	204,0 %	1,0	1,0	2,0	4,0	42,0

40-50 years of age:

40-50 years of age:	Number of responses
0 - 6	8 (72,7%)
7 - 13	2 (18,2%)
14 - 20	0 (0,0%)
21 - 27	0 (0,0%)
28 - 34	0 (0,0%)
35 - 41	0 (0,0%)
42 - 48	0 (0,0%)
49 - 55	0 (0,0%)
56 - 62	0 (0,0%)
63 - 69	1 (9,1%)
Total	11 (100,0%)

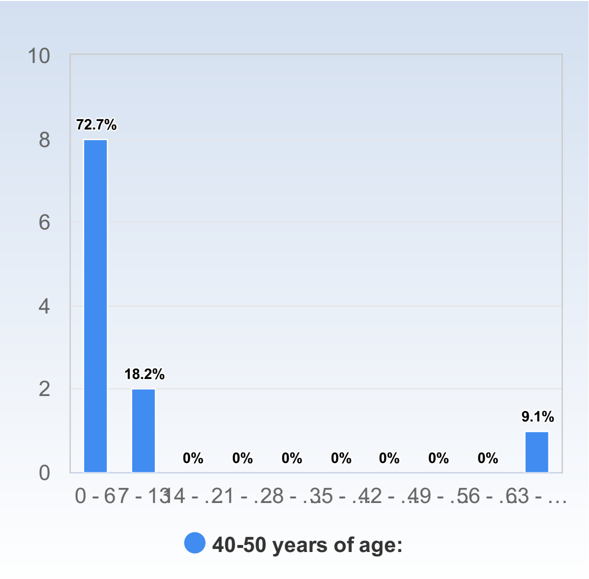

	Mean	Standard Deviation	Coefficient of Variation	Min	Lower Quartile	Median	Upper Quartile	Max
40-50 years of age:	9,7	18,0	184,7 %	1,0	2,5	3,0	7,5	63,0

50-60 years of age:

50-60 years of age:	Number of responses
0 - 5	9 (90,0%)
6 - 11	0 (0,0%)
12 - 17	0 (0,0%)
18 - 23	0 (0,0%)
24 - 29	0 (0,0%)
30 - 35	0 (0,0%)
36 - 41	0 (0,0%)
42 - 47	0 (0,0%)
48 - 53	0 (0,0%)
54 - 59	1 (10,0%)
Total	10 (100,0%)

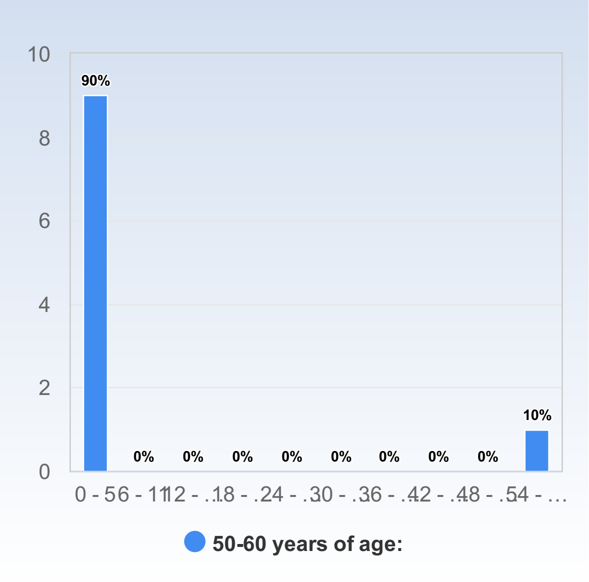

	Mean	Standard Deviation	Coefficient of Variation	Min	Lower Quartile	Median	Upper Quartile	Max
50-60 years of age:	7,8	16,6	213,4 %	1,0	1,5	2,5	4,0	55,0

60-65 years of age:

60-65 years of age:	Number of responses
0 - 1	2 (40,0%)
2 - 3	2 (40,0%)
4 - 5	0 (0,0%)
6 - 7	0 (0,0%)
8 - 9	0 (0,0%)
10 - 11	0 (0,0%)
12 - 13	0 (0,0%)
14 - 15	0 (0,0%)
16 - 17	0 (0,0%)
18 - 19	1 (20,0%)
Total	5 (100,0%)

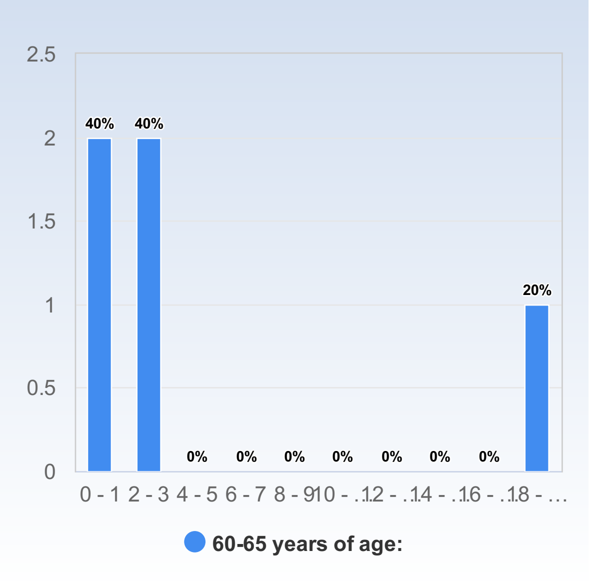

	Mean	Standard Deviation	Coefficient of Variation	Min	Lower Quartile	Median	Upper Quartile	Max
60-65 years of age:	5,2	7,2	138,9 %	1,0	1,0	3,0	3,0	18,0

> 65 years of age:

> 65 years of age:	Number of responses
0	0 (0,0%)
1	0 (0,0%)
2	1 (50,0%)
3	0 (0,0%)
4	0 (0,0%)
5	1 (50,0%)
6	0 (0,0%)
7	0 (0,0%)
8	0 (0,0%)
9	0 (0,0%)
Total	2 (100,0%)

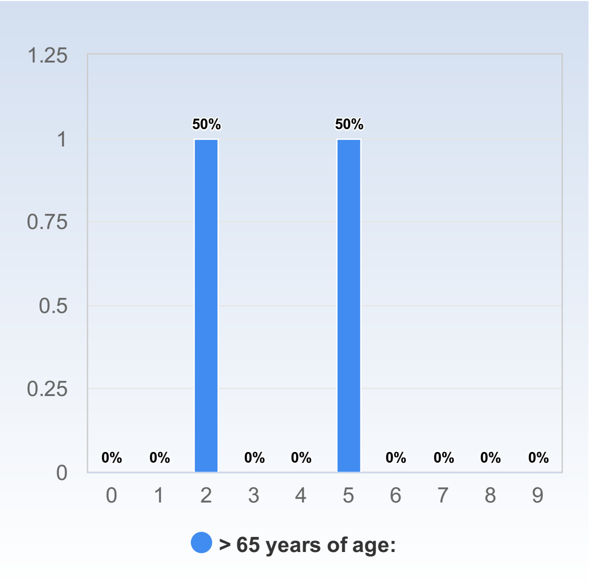

	Mean	Standard Deviation	Coefficient of Variation	Min	Lower Quartile	Median	Upper Quartile	Max
> 65 years of age:	3,5	2,1	60,6 %	2,0	3,5	3,5	3,5	5,0

Is there a need for hiring of replacement /expanding the number of chemical risk assessment/communication personnel over the next 5-10 years in your organization, e.g. due to retirement or change in duties/deliverables within your organisation?

Is there a need for hiring of replacement /expanding the number of chemical risk assessment/communication personnel over the next 5-10 years in your organization, e.g. due to retirement or change in duties/deliverables within your organisation?	Number of responses
YES	11 (91,7%)
NO	1 (8,3%)
Total	12 (100,0%)

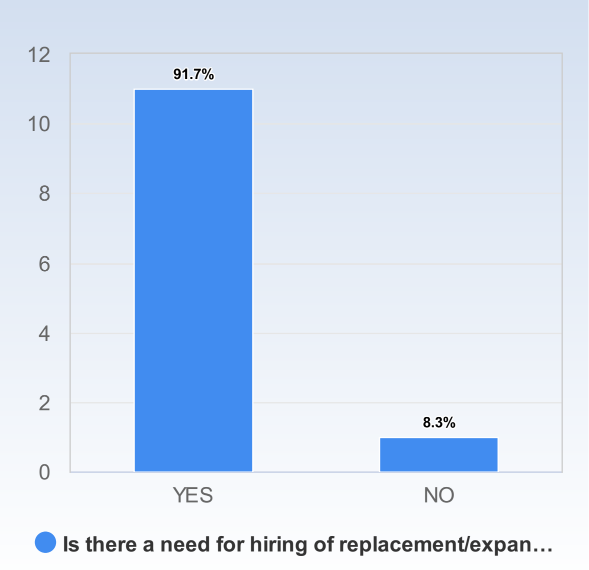

	Mean	Standard Deviation	Coefficient of Variation	Min	Lower Quartile	Median	Upper Quartile	Max
Is there a need for hiring of replacement/expanding the number of chemical risk assessment/communication personnel over the next 5-10 years in your organization, e.g. due to retirement or change in duties/deliverables within your organisation?	1,1	0,3	26,6 %	1,0	1,0	1,0	1,0	2,0

Please comment
Competence in radionuclear risk assessment is needed
We already have a need today, but there is no staff available in the field, especially chaleching for a small, member organization in a niche industry area.
Due to retirement but also due to increasing demands from customers

Which needs do you forsee you will have recruiting relevant personnel for the coming 4 - 7 years? Please address your needs for personnel with general toxicology and related science educations, versus highly specified education.

Which needs do you forsee you will have recruiting relevant personnel for the coming 4 - 7 years? Please address your needs for personnel with general toxicology and related science educations, versus highly specified education.
Recruitment of 5-10 general toxicologists over the next 7 years.
We need to recrute PhD competence with relevant scientific education in toxicology and chemistry.
Our needs will be general toxicology to be able to assess chemicals for safe substitution and risk assessment for air and dermal contact.
We foresee recruiting both tox and ecotox competence in general. Regarding highly specified education we see that both competence within environmental fate and ED is needed. When recruiting we see that few has regulatory knowledge and this is a competence we foresee is needed in combination with above.
The organisation has and will have sufficient staffing in these roles. Yet, if people decide to leave they are very difficult to replace because of lack of experience and training required for the chemical industry.
There will be needs.
We have a lack of experts with competence in food toxicology especially in area of natural toxins, radioactive contamination and food allergy.
General competence of more importance than specified.
We are currently recruiting a person specialized in monitoring of human exposure to hazardous substances in the everyday environment.
In general, students today seem to choose broad, non-specialized educations. This applies to the highest degree to education in the environmental field and means that there are not many with a good knowledge of chemistry and toxicology.
General tox, in combination with an understanding of legislation
Generellt behöver vi mest rekrytera toxikologer, ekotoxikologer, kemister. Gärna disputerade och med olika specialinriktningar/kompetenser. Vi är inne i en större förändring med både en ny förordning (Marknadskontrollförordningen) och Sveriges ordförandeskap i EU som kommer att påverka rekryteringsbehovet och kompetensutvecklingsbehovet av befintlig personal under många år.

Please indicate your experience related to how easy it is to recruit competencies in chemical risk assessment/ communication you want to have /hire. Please use a number between 1 (easy to recruit) and 6 (difficult to recruit).

Please indicate your experience related to how easy it is to recruit competencies in chemical risk assessment/ communication you want to have /hire. Please use a number between 1 (easy to recruit) and 6 (difficult to recruit).	Number of responses
1. Easy to recruit	0 (0,0%)
2.	2 (16,7%)
3.	1 (8,3%)
4.	0 (0,0%)
5.	4 (33,3%)
6. Difficult to recruit	5 (41,7%)
Total	12 (100,0%)

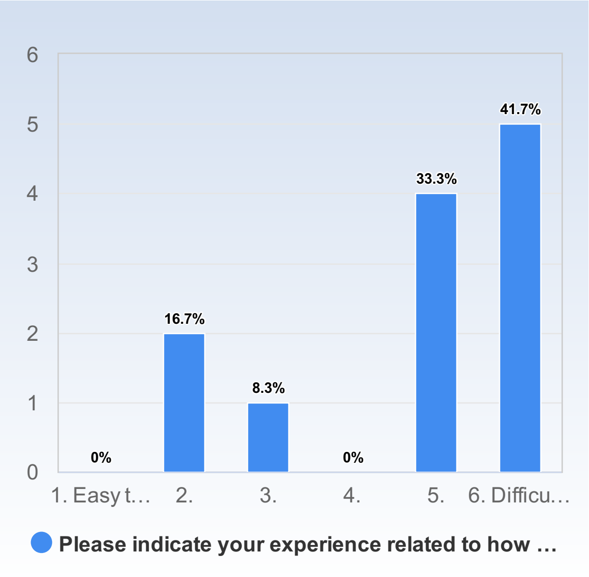

	Mean	Standard Deviation	Coefficient of Variation	Min	Lower Quartile	Median	Upper Quartile	Max
Please indicate your experience related to how easy it is to recruit competencies in chemical risk assessment/ communication you want to have /hire. Please use a number between 1 (easy to recruit) and 6 (difficult to recruit) .	4,8	1,5	32,5 %	2,0	4,0	5,0	6,0	6,0

# Indicate if academia delivers sufficient number of candidates to fulfill your needs (master and/or PhD level)?

Indicate if academia delivers sufficient number of candidates to fulfill your needs (master and/or PhD level)?
Do not know, but there is an obvious lack of Toxicologists in Sweden with any work experience within the area of Risk Assessment. Perhaps the area is too small.....
-
I believe they do, however we are new in the field and it is hard to attract people.
Yes the number of candidates fulfills our need however we would like them to have a more regulatory knowledge within their education.
For chemical industry you need people with interdisciplinary knowledge which is underestimated in academia. Not just in order to better grasp the problems you are faced with but also in order to communicate with other specialists (process engineers, chemists, legal, communication team, business managers etc). The toxicology training used to be better in this but I feel that during the last 20 years the training has become more proned to develop very specialised students only suitable for further academic research.
Lack of experience is the problem
Academia does not deliver candidates fully trained i food toxicology in Sweden.
Not enough to fulfill our needs. We have very few good jobb candidates. We often need PhD level, with broad knowlegde.
My experience is that fresh PhDs are not aware of the possibilities within local authorities. So it's not so much about sufficient number altogether, more that there is a lack of interested candidates.
Se answer before. So, no they don't.
We need experienced competenses, PhDs are great for instilling confidence and demonstrating the importance of knowledge
Ja det gör man ännu.

Do you find it necessary to train your new personnel in the areas of chemical risk assessment/communication due to limited/poor knowledge from academia or previous affiliation (s)/work experience?

Do you find it necessary to train your new personnel in the areas of chemical risk assessment/communication due to limited/poor knowledge from academia or previous affiliation(s) /work experience?	Number of responses
YES	8 (72,7%)
NO	3 (27,3%)
Total	11 (100,0%)

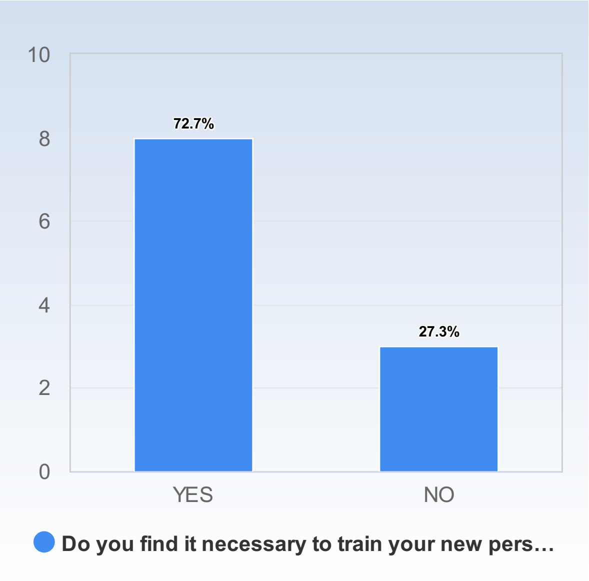

	Mean	Standard Deviation	Coefficient of Variation	Min	Lower Quartile	Median	Upper Quartile	Max
Do you find it necessary to train your new personnel in the areas of chemical risk assessment/communication due to limited/poor knowledge from academia or previous affiliation(s)/work experience?	1,3	0,5	36,7 %	1,0	1,0	1,0	1,5	2,0

If YES, please comment on how you train the personnel.
Formal internal education. Informal education during work.
How to interpret regulation and guidance. We always need to educate for IUCLID and Chesar.
Yes if a newly examined person is hired, that would be necessary. But previous experience is preferred.
Academia does not deliver candidates fully trained i food toxicology in Sweden. Since the food toxicolgy area is a specialized area with demands on knowledge of food habits, consumption etc, this is something that needs to be covered "on the job" by courses by efsa, or by learning from colleagues.
We try to train staff in basic knowledge of different groups of chemicals and their applications and the most common hazards associated with these chemicals. We encourage everyone to attend seminars, webinars, etc. to gain as much knowledge as possible. When possible and we find a suitable course, employees can take these, but the supply is poor.
We need people with competence and experience. We find it possible to teach in legal and other requirements (communication), but impossible for us to teach in deep knowledge in risk assessment.

Which areas of expertise areas are primarily lacking when you want to hire new personnel?

Which areas of expertise areas are primarily lacking when you want to hire new personnel?	Number of responses
Alternative (non-animal) in vitro methods	0 (0,0%)
Animal testing	1 (9,1%)
Bioinformatics	0 (0,0%)
Chemical analysis	0 (0,0%)
Chemistry/Environmental chemistry	3 (27,3%)
Ecotoxicology	1 (9,1%)
Epidemiology	1 (9,1%)
Exposure assessment	1 (9,1%)
QSAR and read-across	1 (9,1%)
Risk assessment	3 (27,3%)
Risk communication	3 (27,3%)
Risk management	3 (27,3%)
Statistics	0 (0,0%)
Systematic literature reviews	0 (0,0%)
Toxicology	3 (27,3%)
Other (please comment below)	6 (54,5%)
Total	26 (236,4%)

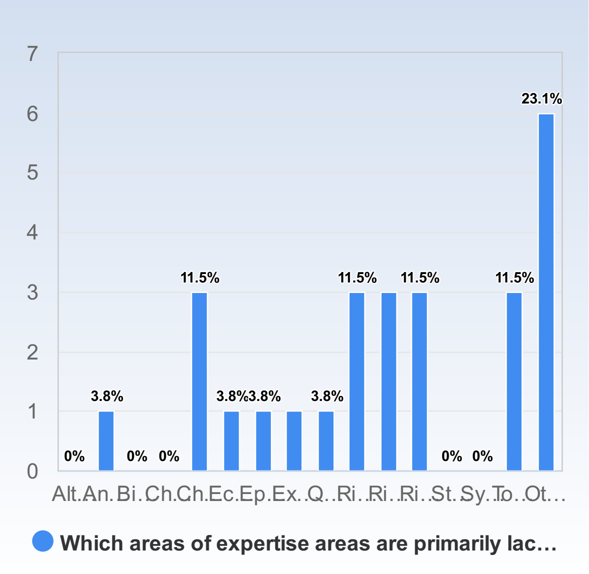

	Mean	Standard Deviation	Coefficient of Variation	Min	Lower Quartile	Median	Upper Quartile	Max
Which areas of expertise areas are primarily lacking when you want to hire new personnel?	22,0	4,2	19,2 %	13,0	19,5	22,0	26,0	27,0

Comment
Connection to industry related topics further from the "normal" areas.
Regulatory<br>IUCLID<br>Chesar<br>How to read guidance
If a hire know one of the topic above, all the others will be lacking. As a regulatory industry toxicologist you need to understand (not only grasp) all boxes above and more!
The combination of the above checked expertise is almost impossible to find in young people.<br>The mindset regarding finding and learning new information is the most important.
Food allergy<br>Radionuclear contamination<br>Immunology
Experience about how to give advice to end-users of chemicals/articles for avoiding/reducing chemical risks.
A combination of more or less everything above, several individuals can complete each other.
Vi hittar i allmänhet dessa kompetenser när vi söker dem.

Please suggest university courses related to chemical risk assessment/communication you would like to see offered by academia in a near future.

Please suggest university courses related to chemical risk assessment/communication you would like to see offered by academia in a near future.
More courses focusing on guidlines and test methods for chemical risk assesment, both in silico, in vitro and in vivo testing.
-
Risk communication in connection to safe substitution and in the communication with suppliers to increase knowledge in the area.
IUCLID<br>Chesar<br>Regulatory globally<br>Needs to be included in the university education
Practical case studies in risk assessment and communication.
There should be more courses on chemical legislation and application of the legislation.<br>CLP, REACH, Chemical riskassessment
Food toxicology. Risk- benefit assessments. Immunology. Epidemiology. Radionuclear contamination
Risk assessment and risk management of the catchment areas for abstraction points of water intended for human consumption - to fulfill the new drinking water directive.
Chemical risk reduction in non-chemical contexts (i.e. outside the traditional laboratory/industrial context. Focussing on the use of materials, articles, products and communicating with suppliers and customers. How to get information from suppliers etc)
Courses that meet the need for staff with knowledge of chemicals, toxicology and legislation at a level that allows them to work in different industries. It is not always necessary to have knowledge at a master's level in, for example, toxicology. However, the broad educations that have become popular sometimes include only one semester in total of toxicology and chemistry. It is not enough to understand chemicals and how they work in different materials, different fractions, toxicological aspects and legislation.
Understanding legal requirements and legal processes. Reach, CLP, ...
Test methods (non-animal), Basic compare animal testing vs non-animal testing. How to integrate research info, useful regulatory actions and understanding how science and policy interact. Developments in bioinformatics.

Please indicate, if you expect a lack of expertise in the area of risk assessment/ communication in your organisation, in the near future.

Please indicate, if you expect a lack of expertise in the area of risk assessment/ communication in your organisation, in the near future.	Number of responses
1. Not expected	1 (8,3%)
2.	1 (8,3%)
3.	1 (8,3%)
4.	3 (25,0%)
5.	3 (25,0%)
6. Definitely expected	3 (25,0%)
Total	12 (100,0%)

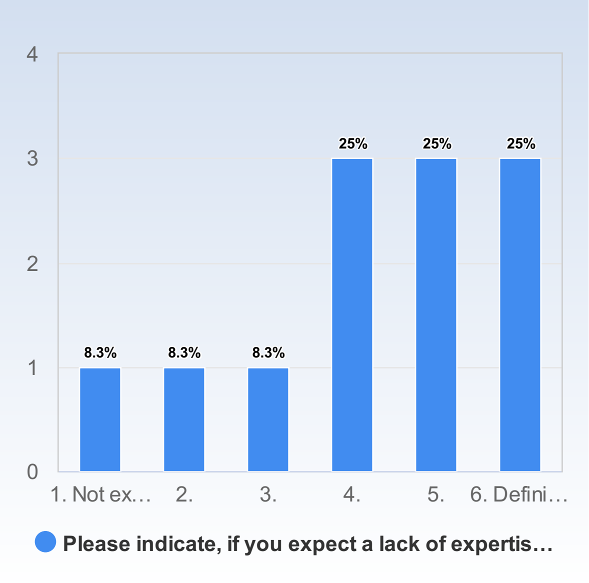

	Mean	Standard Deviation	Coefficient of Variation	Min	Lower Quartile	Median	Upper Quartile	Max
Please indicate, if you expect a lack of expertise in the area of risk assessment/ communication in your organisation, in the near future.	4,3	1,6	37,7 %	1,0	3,5	4,5	5,5	6,0

Please explain how you foresee to meet the needs of highly competent/qualified personnel in chemical risk assessment/communication in your organization five years from now (e.g. external courses, workshops or internal courses).

Please explain how you foresee to meet the needs of highly competent/qualified personnel in chemical risk assessment/communication in your organization five years from now (e.g. external courses, workshops or internal courses).
A mix of recruitment, external and internal courses.
External and internal courses, workshops etc.
Mostly with research projects and courses for personnel.
Internal education, collaboration with universities/consultants, workshop by authorities and international conferences.
If no staff quite we have the right level om competence. If we need to hire and replace a lot of internal training is required.
Hoping to find good people with the right mindset. Then internal education.
Via external courses, workshops or internal courses
If we can not find a highly qualified person to hire, I guess we have to educate a less qualified person. Most internal education (person to person), but also external 1-2 day courses.
We hope to further develop our cooperation with academia in terms of oint projects, master students, discussions about research findings and our questions etc.
We try to hire new personnel through search recrutments that unfortunatelly are time consuming and expensive.
hire experienced and supplement with internal supervision

Please give suggestions what can be done nationally to optimize the numbers of competent persons for your organization.

Please give suggestions what can be done nationally to optimize the numbers of competent persons for your organization.
Adjust the current national courses in Toxicology to meet the current needs of Swedish organisations.
-
I think the Substitution centre is doing a great job, however the need is for all industries.
See earlier replies. We recruit globally and do not see a specific national need.
N/A
Networking
We find it efficient to train toxicologists "on the job" after recruitment. The most important prerequicite is that the basic training of toxicologists is kept at a high international level.
Long term: To educate more people.<br>Short term: To form a network with people working in the area that can share competens and job opportunities.
Se answer on question 14
networking, cooperations between konsultants and other organisations. Support networking with like-minded abroad
A good balance between academia/research, regulators/administration and industry.

Please suggest how the Nordic countries could act jointly to optimize the numbers of competent persons for your organization.

Please suggest how the Nordic countries could act **jointly** to optimize the numbers of competent persons for your organization.
A more active joint toxicology organization.
The Nordic Expert Group for Criteria Documentation of Health Risks from Chemicals is an example of well-functioning Nordic joint work. Other types of background documentation as well as impact assessments for specific chemical agents are fields that may be suitable for Nordic collaboration.
Make sure there is a possibility to help all industries.
Nordic trainings and networks are always useful to increase the critical mass.
Colaboration and networking
The option to cooperate with other Nordic authorities, for example through the EFSA EU-fora mechanism with expert exchange.
Have no suggestion.
Can universities merge, use their common resource pool and develop different types of education? Some parts may be common to all students. Others could be aimed at those who want to continue in academia and others at those who want to enter, for example, industry.
Networking and common trainings and educations. Invite to seminars and presentations.
Inget svar här.

Do you see positive possibilities for a closer, formalized cooperation between the Nordic countries in the area of chemical risk analysis, including training?

Do you see positive possibilities for a closer, formalized cooperation between the Nordic countries in the area of chemical risk analysis, including training?	Number of responses
YES	8 (80,0%)
NO	2 (20,0%)
Total	10 (100,0%)

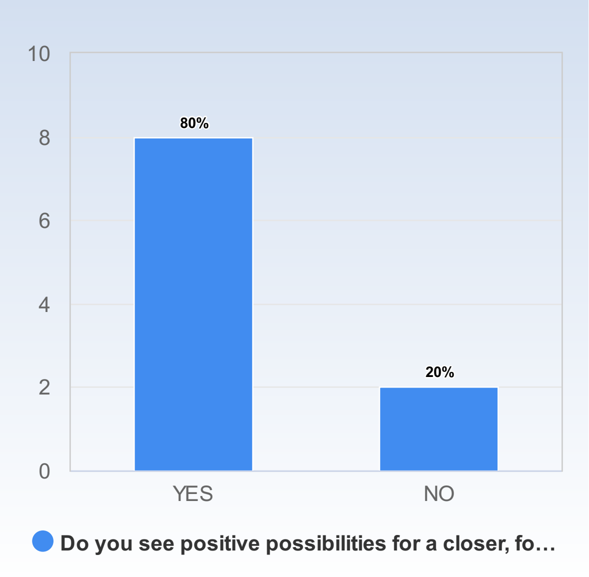

	Mean	Standard Deviation	Coefficient of Variation	Min	Lower Quartile	Median	Upper Quartile	Max
Do you see positive possibilities for a closer, formalized cooperation between the Nordic countries in the area of chemical risk analysis, including training?	1,2	0,4	35,1 %	1,0	1,0	1,0	1,0	2,0

If YES - How could this cooperation be accomplished? / If NO - Why would that be hard to accomplish?
-
Our area is to small to address this.
Workshops where knowledge can be shared.
Networking, collaboration between national authorities
see for example question 17
Do not know
From my own experience I know that one nordic country are not big enough to be speciliced in all diciplinces
See no 17
Inget svar på denna fråga.

How many of your experts within chemical risk assessment/communication are involved in international assignments related to the organizations indicated below?

EFSA panels:

EFSA panels:	Number of responses
0	2 (40,0%)
1	1 (20,0%)
2	1 (20,0%)
3	1 (20,0%)
4	0 (0,0%)
5	0 (0,0%)
6	0 (0,0%)
7	0 (0,0%)
8	0 (0,0%)
9	0 (0,0%)
Total	5 (100,0%)

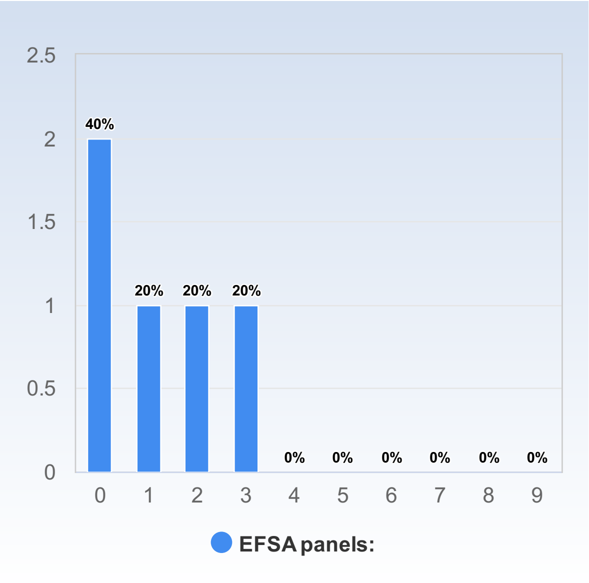

	Mean	Standard Deviation	Coefficient of Variation	Min	Lower Quartile	Median	Upper Quartile	Max
EFSA panels:	1,2	1,3	108,7 %	0,0	0,0	1,0	2,0	3,0

ECHA expert groups and member state committee:

ECHA expert groups and member state committee:	Number of responses
0 - 1	3 (75,0%)
2 - 3	0 (0,0%)
4 - 5	0 (0,0%)
6 - 7	0 (0,0%)
8 - 9	0 (0,0%)
10 - 11	0 (0,0%)
12 - 13	0 (0,0%)
14 - 15	1 (25,0%)
16 - 17	0 (0,0%)
18 - 19	0 (0,0%)
Total	4 (100,0%)

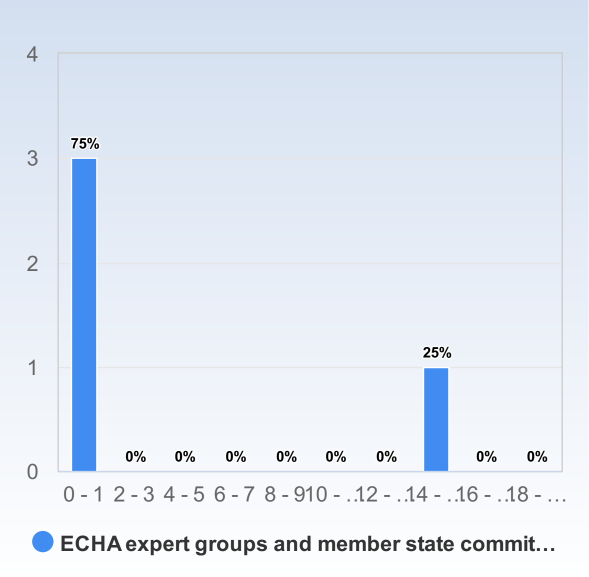

	Mean	Standard Deviation	Coefficient of Variation	Min	Lower Quartile	Median	Upper Quartile	Max
ECHA expert groups and member state committee:	4,3	7,2	169,0 %	0,0	0,5	1,0	8,0	15,0

EC scientific committees:

EC scientific committees:	Number of responses
0	2 (66,7%)
1	0 (0,0%)
2	0 (0,0%)
3	1 (33,3%)
4	0 (0,0%)
5	0 (0,0%)
6	0 (0,0%)
7	0 (0,0%)
8	0 (0,0%)
9	0 (0,0%)
Total	3 (100,0%)

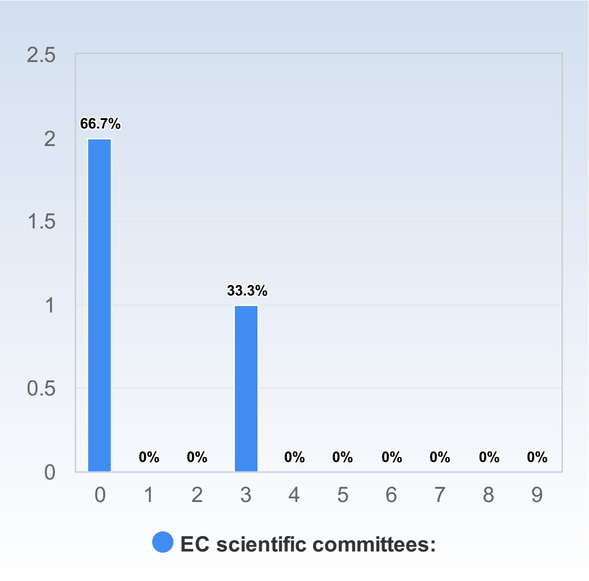

	Mean	Standard Deviation	Coefficient of Variation	Min	Lower Quartile	Median	Upper Quartile	Max
EC scientific committees:	1,0	1,7	173,2 %	0,0	0,0	0,0	1,5	3,0

EMA committees or working groups:

EMA committees or working groups:	Number of responses
0	2 (66,7%)
1	1 (33,3%)
2	0 (0,0%)
3	0 (0,0%)
4	0 (0,0%)
5	0 (0,0%)
6	0 (0,0%)
7	0 (0,0%)
8	0 (0,0%)
9	0 (0,0%)
Total	3 (100,0%)

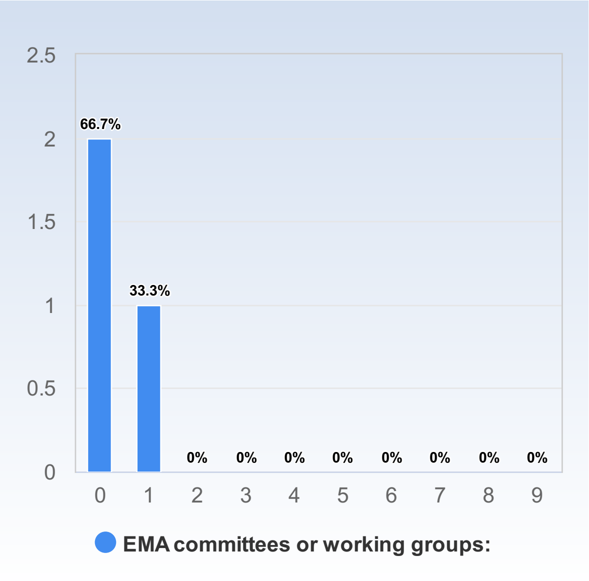

	Mean	Standard Deviation	Coefficient of Variation	Min	Lower Quartile	Median	Upper Quartile	Max
EMA committees or working groups:	0,3	0,6	173,2 %	0,0	0,0	0,0	0,5	1,0

OECD working groups:

OECD working groups:	Number of responses
0	2 (50,0%)
1	1 (25,0%)
2	0 (0,0%)
3	0 (0,0%)
4	1 (25,0%)
5	0 (0,0%)
6	0 (0,0%)
7	0 (0,0%)
8	0 (0,0%)
9	0 (0,0%)
Total	4 (100,0%)

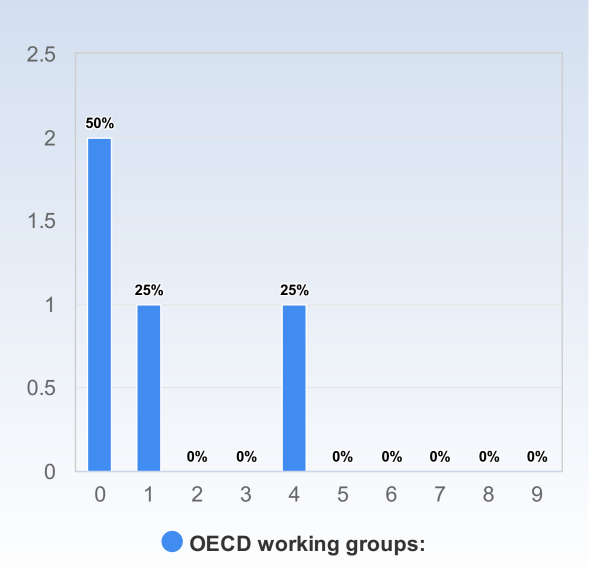

	Mean	Standard Deviation	Coefficient of Variation	Min	Lower Quartile	Median	Upper Quartile	Max
OECD working groups:	1,3	1,9	151,4 %	0,0	0,0	0,5	2,5	4,0

WHO expert groups:

WHO expert groups:	Number of responses
0	2 (100,0%)
1	0 (0,0%)
2	0 (0,0%)
3	0 (0,0%)
4	0 (0,0%)
5	0 (0,0%)
6	0 (0,0%)
7	0 (0,0%)
8	0 (0,0%)
9	0 (0,0%)
Total	2 (100,0%)

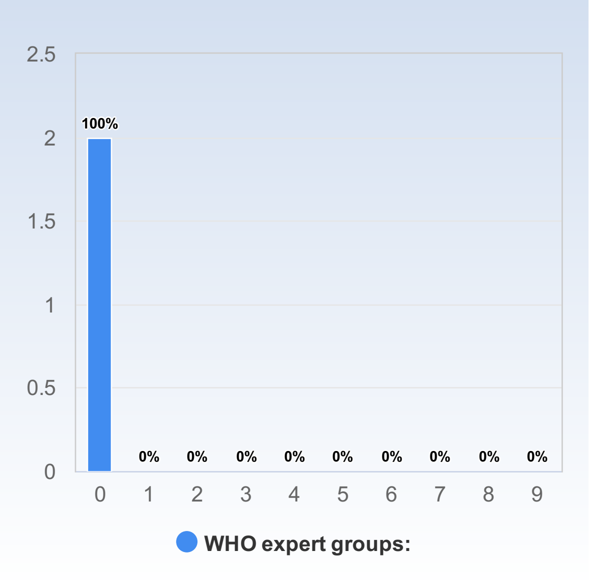

	Mean	Standard Deviation	Coefficient of Variation	Min	Lower Quartile	Median	Upper Quartile	Max
WHO expert groups:	0,0	0,0	NaN %	0,0	0,0	0,0	0,0	0,0

Other(s) (please comment below)

Other(s) (please comment below)	Number of responses
0	0 (0,0%)
1	1 (50,0%)
2	0 (0,0%)
3	1 (50,0%)
4	0 (0,0%)
5	0 (0,0%)
6	0 (0,0%)
7	0 (0,0%)
8	0 (0,0%)
9	0 (0,0%)
Total	2 (100,0%)

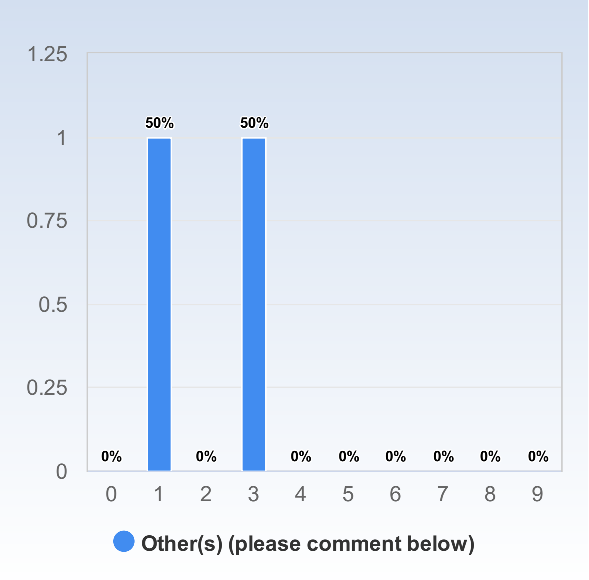

	Mean	Standard Deviation	Coefficient of Variation	Min	Lower Quartile	Median	Upper Quartile	Max
Other(s) (please comment below)	2,0	1,4	70,7 %	1,0	2,0	2,0	2,0	3,0

Comment
Since we are a industry far from the field, we are not involved.
None
"Other": 3 experts are members of EFSA panel working groups other than those part of EFSA panels.
SAICM - related working groups<br>UNEP - related worinkg groups on sustainable chemicals

Do you expect that the number of personnel involved in international assignments (c.f. previous question) will increase or decrease in the coming 5 years?

Do you expect that the number of personnel involved in international assignments (c.f. previous question) will increase or decrease in the coming 5 years?	Number of responses
Increase	4 (40,0%)
Decrease	1 (10,0%)
No change is expected	5 (50,0%)
Total	10 (100,0%)

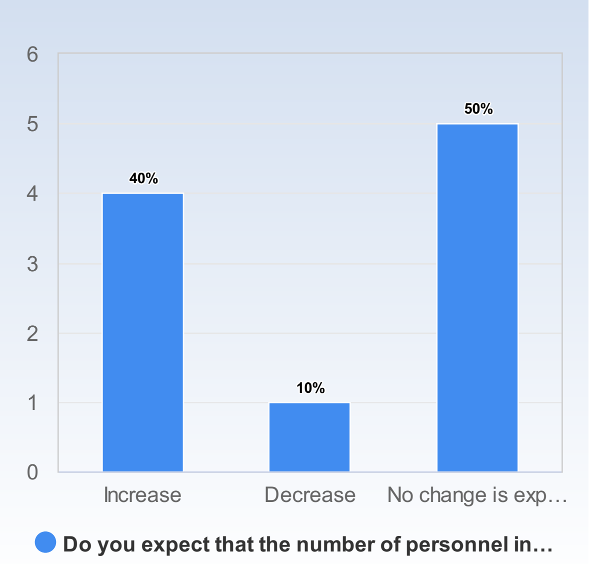

	Mean	Standard Deviation	Coefficient of Variation	Min	Lower Quartile	Median	Upper Quartile	Max
Do you expect that the number of personnel involved in international assignments (c.f. previous question) will increase or decrease in the coming 5 years?	2,1	1,0	47,4 %	1,0	1,0	2,5	3,0	3,0

Please comment.
We need to make sure risk assessment will be part of the decisions in our field.
Likely as we have fairly young experts currently

Any additional reflections or comments regarding competence provision needs in the area of risk assessment/communication?

Any additional reflections or comments regarding competence provision needs in the area of risk assessment/communication?
-
We are small in this context and are looking at how we can use risk assessment in our daily work. This is a learning process for us as well.
From our perspective (consultants) the need for help from companies and authorities is increasing together with more complex issues.<br>We are continously educating our customers regarding these matters, which is one reason for the meaningfulness of consultancy.
Since the EU-legislations nowdays often include risk assessment and risk management, the need for highly qualified people with this competence will be required.
I see two different kind of needs:<br><br>To strengthen universities by exchanging research / education at all levels. This to form specialists at a high level.<br>To create training that provides competent employees for other activities such as industry, municipalities, etc
The area is becomming more and more complex. The legal requirements very often require an expert to interpret and follow; this is very clear from a conultants horizon.

SI Part G

Merged analyses of the result of respondents from Denmark, Finland, Norway and Sweden

Result of the questionnaire responses as obtained from Denmark (DK), Finland (FI), Norway (NO) and Sweden (SE).

The results are put together into one document to give an overview of the results and to help in the interpretation of the results. Still it is valid to look into the national wise reports from the survey. This document does not contain any views from the project group.

Question 1: Please indicate your area of affiliation.

Total number of responses from DK, FI, NO and SE: 40. The responses are distributed as shown below

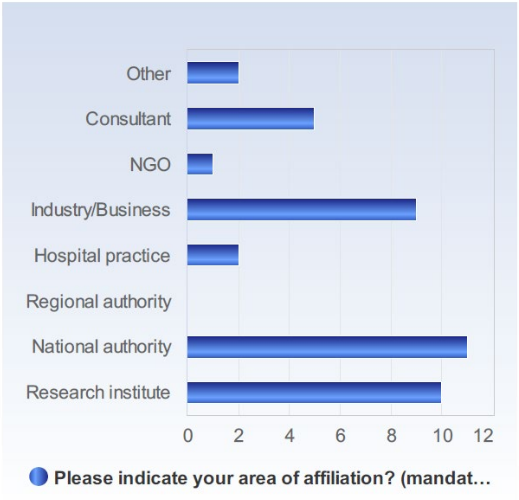

Question 2. What is the number of personnel in chemical risk assessment/risk communication in your organization?

Most responses indicate 1-10 personnel, then 10-20 and only occasionally more. Still the responses may include 99-109 personnel numbers (DK); FI indicate two with 55-65 personnel, NO has one with 33-43 and one with 55-65.

Question 3: What specialization, in chemical risk assessment /communication, do you have in your organization? Please estimate the number of personnel for each specialization.

In order to see the actual numbers, we need to consult the country wise responses. However, if merged, we can see that all professional areas are included among the answers i.e. Alternative (non-animal) in vitro methods, Animal testing, Bioinformatics, Chemical analysis, Chemistry/Environ-mental chemistry, Ecotoxicology, Epidemiology, Exposure assessment, QSAR and read-across, Risk assessment, Risk communication, Risk management, Statistics, Systematic literature reviews, Toxicology and some others were mentioned.

Response/Comments DK.

Toxicogenomics
The figure 30 is specifically those considered as "toxicologist". They are all involved in in vitro/in vivo methods, exposure and risk assessment.
The total number of personnel working in all these fields are large, but not all focus on toxicology as a discipline. Numbers of those working with testing are higher than given here, as toxicology studies are outsourced. In addition the number of chemical analysis, ADME and assays scientists are many (not counted here).
On the previous page only employees with research functions are indicated, not technicians
I have tried to indicate the person years allocated to each task.

Response/Comments FI.

One person part-time
Very difficult the address the exact numbers of the people working in the specific areas in a large research organisation. Please take these numbers as indicative. Risk assessment includes exposure assessment. Number of researchers involved in the field is 4, but chemical risk assessment mainly by 2 persons. One out of 2 conducts epidemiological assessments, 2 other persons are statisticians. Chemical risk assessment needs knowledge of toxicology. All the personnel is involved with communication.
We would like to point out that same persons work in different specializations. Also, most of our staff carry out work related to risk assessment only part-time. However, the risk assessment is linked to everybody's work more or less, and that is why we named all our researchers in question 2.
Our main task is chemical analysis, which takes about 3-person years in total (involved 5 persons). Exposure and risk assessment + communication and toxicology take nowadays only 1 – 1.5 person year in total. Four (4) persons have some knowledge on various disciplines of toxicology but are not toxicologists by education.
Actual work in some areas asked in the Authority is mainly other, but skills to do is available. Risk assessment in based on comparison to TDI or similar definitions made by EFSA or other specific institutes.
Finnish Poison Information Centre, we have a team of 15 people with pharmaceutical and medical background. We focus on clinical toxicology with expertise in risk assessment, management and medical toxicology.
Twenty-six (26) persons working in our chemistry lab (all marked to "chemical analyses" are involved in the analysing either air, surface or biomonitoring samples from the workplaces. These include also laboratory technicians. Exposure assessors (26) include industrial hygienists providing industrial hygienic services to Finnish workplaces and 4 industrial hygienist who are mainly involved in research projects (instead of expert services provided by our institute). Toxicologist performing toxicology research in the lab have been divided between in vitro/in vivo according to PM distribution/year (however, there are 4 toxicologist able to run animal studies). Additionally, there are toxicologists/chemists (7) who are more involved in risk assessment activities; these include regulatory risk assessment, limit value setting, risk assessment activities in research projects, toxicological support to our occupational health clinics, biomonitoring activities. These have been divided between "toxicology" and "Risk Assessment" but this is rather arbitrary division. Same person deals with several areas of specialization.
We have excluded chemistry because most of our personnel are chemists, as we are a chemical company.

Response/Comments NO.

Specialisation is a relative term. I have some experience within these topics. In addition to myself I have colleagues who are specialised in risk assessment methodology, and SEVESO risk. This is of course also chemical risk assessment competency, but I have not included them in the number here since the questionnaire seems to be focused on chemical hazard as such, not so much how it is used.

Veterinary toxicology, wild and farm animal poisonings it is very challenging to provide numbers since our institution is very big with many different specialities and not each speciality knows each other personally. The numbers might not reflect the real situation and must be seen as an rough estimate.

Response/Comments SE.

This is a new field in our industry and we are trying to make the best out of it.

Question 4: How many of the personnel in your organization, with the chemical risk assessment/communication expertise, holds a:

Bachelor degree: FI has most BS (65-81), followed by DK with 33-39 and SE with 5. None in NO

Master degree: FI has ≤138; SE ≤132; DK ≤124 and NO ≤39. The responses were given as ranges but this is the highest number presented by the respondents.

Doctoral degree (PhDs, MDs, Dtech (TkT, D.I.T), etc): SE has ≤108; DK ≤98; FI ≤84 and NO ≤33. The responses were given as ranges but this is the highest number presented by the respondents.

Question 5: European Registered Toxicologist (ERTs):

The total numbers of ERTs in the Nordic countries are: DK: 39; FI: 59; IS: to come; NO: 70; SE: 45. Please note that these are the total numbers of ERTs in each of the countries. ERTs engaged in risk assessment of environmental hazards are lower.

Question 6: What is the present age profile of the experts in your organization?

Question 6: **What is the present age profile of the experts in your organization?**

DK

< 40 years of age:	Number of responses
2 - 6	2 (50,0%)
7 - 11	1 (25,0%)
12 - 16	0 (0,0%)
17 - 21	0 (0,0%)
22 - 26	0 (0,0%)
27 - 31	0 (0,0%)
32 - 36	0 (0,0%)
37 - 41	0 (0,0%)
42 - 46	0 (0,0%)
47 - 51	1 (25,0%)
Total	4 (100,0%)

FI

< 40 years of age:	Number of responses
0 - 2	5 (50,0%)
3 - 5	1 (10,0%)
6 - 8	1 (10,0%)
9 - 11	1 (10,0%)
12 - 14	1 (10,0%)
15 - 17	0 (0,0%)
18 - 20	0 (0,0%)
21 - 23	1 (10,0%)
24 - 26	0 (0,0%)
27 - 29	0 (0,0%)
Total	10 (100,0%)

NO

< 40 years of age:	Number of responses
0 - 3	1 (25,0%)
4 - 7	0 (0,0%)
8 - 11	1 (25,0%)
12 - 15	0 (0,0%)
16 - 19	1 (25,0%)
20 - 23	0 (0,0%)
24 - 27	0 (0,0%)
28 - 31	0 (0,0%)
32 - 35	1 (25,0%)
36 - 39	0 (0,0%)
Total	4 (100,0%)

SE

< 40 years of age:	Number of responses
0 - 4	7 (77,8%)
5 - 9	1 (11,1%)
10 - 14	0 (0,0%)
15 - 19	0 (0,0%)
20 - 24	0 (0,0%)
25 - 29	0 (0,0%)
30 - 34	0 (0,0%)
35 - 39	0 (0,0%)
40 - 44	1 (11,1%)
45 - 49	0 (0,0%)
Total	9 (100,0%)

Question 6: **What is the present age profile of the experts in your organization?**

DK

40-50 years of age:	Number of responses
1 - 3	1 (16,7%)
4 - 6	1 (16,7%)
7 - 9	2 (33,3%)
10 - 12	1 (16,7%)
13 - 15	0 (0,0%)
16 - 18	0 (0,0%)
19 - 21	0 (0,0%)
22 - 24	0 (0,0%)
25 - 27	0 (0,0%)
28 - 30	1 (16,7%)
Total	6 (100,0%)

FI

40-50 years of age:	Number of responses
0 - 2	3 (27,3%)
3 - 5	4 (36,4%)
6 - 8	1 (9,1%)
9 - 11	0 (0,0%)
12 - 14	0 (0,0%)
15 - 17	0 (0,0%)
18 - 20	1 (9,1%)
21 - 23	1 (9,1%)
24 - 26	1 (9,1%)
27 - 29	0 (0,0%)
Total	11 (100,0%)

NO

40-50 years of age:	Number of responses
0 - 5	5 (71,4%)
6 - 11	0 (0,0%)
12 - 17	0 (0,0%)
18 - 23	0 (0,0%)
24 - 29	1 (14,3%)
30 - 35	0 (0,0%)
36 - 41	0 (0,0%)
42 - 47	0 (0,0%)
48 - 53	1 (14,3%)
54 - 59	0 (0,0%)
Total	7 (100,0%)

SE

40-50 years of age:	Number of responses
0 - 6	8 (72,7%)
7 - 13	2 (18,2%)
14 - 20	0 (0,0%)
21 - 27	0 (0,0%)
28 - 34	0 (0,0%)
35 - 41	0 (0,0%)
42 - 48	0 (0,0%)
49 - 55	0 (0,0%)
56 - 62	0 (0,0%)
63 - 69	1 (9,1%)
Total	11 (100,0%)

Question 6: **What is the present age profile of the experts in your organization?**

DK

50-60 years of age:	Number of responses
1	2 (33,3%)
2	0 (0,0%)
3	1 (16,7%)
4	0 (0,0%)
5	1 (16,7%)
6	0 (0,0%)
7	0 (0,0%)
8	0 (0,0%)
9	0 (0,0%)
10	2 (33,3%)
Total	6 (100,0%)

FI

50-60 years of age:	Number of responses
2 - 3	6 (60,0%)
4 - 5	1 (10,0%)
6 - 7	0 (0,0%)
8 - 9	1 (10,0%)
10 - 11	0 (0,0%)
12 - 13	1 (10,0%)
14 - 15	0 (0,0%)
16 - 17	0 (0,0%)
18 - 19	0 (0,0%)
20 - 21	1 (10,0%)
Total	10 (100,0%)

NO

50-60 years of age:	Number of responses
2 - 5	1 (16,7%)
6 - 9	1 (16,7%)
10 - 13	0 (0,0%)
14 - 17	2 (33,3%)
18 - 21	0 (0,0%)
22 - 25	0 (0,0%)
26 - 29	0 (0,0%)
30 - 33	1 (16,7%)
34 - 37	0 (0,0%)
38 - 41	1 (16,7%)
Total	6 (100,0%)

SE

50-60 years of age:	Number of responses
0 - 5	9 (90,0%)
6 - 11	0 (0,0%)
12 - 17	0 (0,0%)
18 - 23	0 (0,0%)
24 - 29	0 (0,0%)
30 - 35	0 (0,0%)
36 - 41	0 (0,0%)
42 - 47	0 (0,0%)
48 - 53	0 (0,0%)
54 - 59	1 (10,0%)
Total	10 (100,0%)

Question 6: **What is the present age profile of the experts in your organization?**

DK

60-65 years of age:	Number of responses
1	1 (33,3%)
2	0 (0,0%)
3	0 (0,0%)
4	0 (0,0%)
5	1 (33,3%)
6	0 (0,0%)
7	0 (0,0%)
8	0 (0,0%)
9	0 (0,0%)
10	1 (33,3%)
Total	3 (100,0%)

FI

60-65 years of age:	Number of responses
0	1 (16,7%)
1	1 (16,7%)
2	2 (33,3%)
3	0 (0,0%)
4	0 (0,0%)
5	0 (0,0%)
6	1 (16,7%)
7	0 (0,0%)
8	0 (0,0%)
9	1 (16,7%)
Total	6 (100,0%)

NO

60-65 years of age:	Number of responses
1 - 2	1 (25,0%)
3 - 4	1 (25,0%)
5 - 6	0 (0,0%)
7 - 8	1 (25,0%)
9 - 10	0 (0,0%)
11 - 12	0 (0,0%)
13 - 14	0 (0,0%)
15 - 16	0 (0,0%)
17 - 18	0 (0,0%)
19 - 20	1 (25,0%)
Total	4 (100,0%)

SE

60-65 years of age:	Number of responses
0 - 1	2 (40,0%)
2 - 3	2 (40,0%)
4 - 5	0 (0,0%)
6 - 7	0 (0,0%)
8 - 9	0 (0,0%)
10 - 11	0 (0,0%)
12 - 13	0 (0,0%)
14 - 15	0 (0,0%)
16 - 17	0 (0,0%)
18 - 19	1 (20,0%)
Total	5 (100,0%)

Question 6: **What is the present age profile of the experts in your organization?**

DK

> 65 years of age:	Number of responses
0	0 (0,0%)
1	1 (100,0%)
2	0 (0,0%)
3	0 (0,0%)
4	0 (0,0%)
5	0 (0,0%)
6	0 (0,0%)
7	0 (0,0%)
8	0 (0,0%)
9	0 (0,0%)
Total	1 (100,0%)

FI

> 65 years of age:	Number of responses
0	1 (50,0%)
1	1 (50,0%)
2	0 (0,0%)
3	0 (0,0%)
4	0 (0,0%)
5	0 (0,0%)
6	0 (0,0%)
7	0 (0,0%)
8	0 (0,0%)
9	0 (0,0%)
Total	2 (100,0%)

NO

> 65 years of age:	Number of responses
0	1 (33,3%)
1	0 (0,0%)
2	0 (0,0%)
3	0 (0,0%)
4	0 (0,0%)
5	1 (33,3%)
6	0 (0,0%)
7	0 (0,0%)
8	1 (33,3%)
9	0 (0,0%)
Total	3 (100,0%)

SE

> 65 years of age:	Number of responses
0	0 (0,0%)
1	0 (0,0%)
2	1 (50,0%)
3	0 (0,0%)
4	0 (0,0%)
5	1 (50,0%)
6	0 (0,0%)
7	0 (0,0%)
8	0 (0,0%)
9	0 (0,0%)
Total	2 (100,0%)

Comment: More than 65 years of age: FI and DK both only one person above 65. SE and NO have the largest number of older personnel, i.e. to be substituted.

Question 7 Is there a need for hiring of replacement/expanding the number of chemical risk assessment/communication personnel over the next 5-10 years in your organization, e.g. due to retirement or change in duties/deliverables within your organisation?

Response/Comments DK.

Probably, but entirely depending on funding
To a small degree based on natural changes.
No expected change in number of personnel. Continuous replacement.
We have vacant positions in especially exposure assessment, and additional recruitment might be relevant depending on potential increase in funding. Recruitment due to retirement will not be relevant the next 10 years.

Response/Comments FI.

Yes, with regulative experience.
In principle, we would need continuation in analytic epidemiology on cancer risks caused by occupational and environmental hazards, but our institute has decided that it will not be within a key function for its activities
There is need due the new directives from EU and retirement.
No retirements in the nearest future, and the turnover of the permanent personnel is low.
Project researchers will be recruited when needed.
A few persons (toxicologists) have already retired, and no one has been hired to replace them
Certain number expertise needed, due to retirement new experts needed.
Currently not foreseen.
Realistic needs, but it may be left out in authority's strategic planning
ERT will retire within next 5 years. Some other retirements within next few years.
Need to hire industrial hygienists able to measure and assess exposure at workplaces; we have currently two open positions. In addition, we have recently hired one new toxicologist and we might also need one additional (temporary contract) researcher in our genotoxicology laboratory this year or next year.
Due to retirement 3, 2-5 additional due to increasing workload --> if financially possible.

Response/Comments NO.

If possible, we would have had two persons with this type of skills in our organisation, but that is not possible to get - there are none in this area.
Retirements. However, future need is hard to decide, depending on tasks of the organization
We will have some people that are to retire. Possible that other people find positions outside the department, including managerial roles.
We need new expertise and replace / strengthen existing areas of risk assessment within the department.
Retirement and change in deliverables

Response/Comments SE.

We already have a need today, but there is no staff available in the field, especially challenging for a small, member organization in a niche industry area.
Due to retirement but also due to increasing demands from customers

Question 8: Which needs do you foresee you will have recruiting relevant personnel for the coming 4-7 years? Please address your needs for personnel with general toxicology and related science educations, versus highly specified education.

Response/Comments DK.

Expertise in probabilistic risk assessment based on artificial intelligence and novel approach methodologies (NAMs) and exposure modelling.
The primary need is for personnel with experience in regulatory (eco)toxicology. Both highly specialized and more general competences are needed for different tasks.
We expect to be able to recruit general scientists and provide education in toxicology. Challenge will be to include scientists strong in computational skills with insight in our field
Personnel in field exposure assessment/monitoring (science education) Epidemiologists with skills in register studies (public health education) Maybe toxicologists.

Response/Comments FI.

General toxicology, regulative toxicology, biocidal substances
Lack of funding and other resources will be the main problem. Another problem is that there will be lack of well capable specialists in the collaborating institutes due to the same problem in their resource allocation.
1. New regulations from EU i.e. Do Not Significant Harm principle and Single Use Plastic Directive; 2. Retirement; 3. New Biomaterials development; 4. Recirculation and utilization of industrial side streams.
We have a need for toxicology experts, but it is unlikely we will have the possibility to recruit permanent personnel.
We would need to hire person/persons with environmental toxicology and risk assessment educations
Need to assure to have in minimum one general toxicologist in the organisation.
There is likely a need to hire toxicologists, in particular general toxicologists. Risk assessors or exposure assessors are more challenging to hire, as rarely a general toxicologist has also experience in risk assessment or exposure assessment or e.g. a (food) chemist has experience in exposure assessment.
Basic training on understanding toxicity is urgently needed. This provides route to specific education
Personnel with general toxicology educations, with chemistry and pharmacy educations.
Finnish Poison Information Center has a continuous need for BScs in Pharmacy and MDs with special expertise in acute medicine, pharmacology and toxicology.
See the previous question. We need industrial hygienists and since it requires special expertise and education possibilities for it are limited in Finland we have had challenges in finding suitable candidates. In addition, in latest toxicology recruitment we had challenges to find suitable candidates; in two last recruitments the other one who was hired came outside Nordic countries (we were specifically searching some existing experience in genotoxicology and laboratory work). For risk assessment positions we have had open lately, it has been also really difficult to find toxicologists who would have had earlier experience/or any real knowledge of toxicological risk assessment or occupational toxicology. Since occupational toxicology is rather important field if we consider e.g. regulatory toxicology (e.g. under REACH) there is a real need to increase knowledge of this field. This should start already from the basic toxicology education.
General toxicology and risk assessment, environmental toxicology.
MSc in ecotoxicology/environmental chemistry, MSc in chemistry; MSc in toxicology; general toxicology.

Response/Comments NO.

Ecotoxicology/environmental management system skills. Due to retirement a general toxicologist will also be needed in about 7 years.

Chemistry, risk assessments, veterinary toxicology, feed toxicology, poisonings, we expect that we have 6-8 vacancies. We expect general Toxicology training and thereafter specialisation in a specific area of toxicology like inhalation, genetic, immunotoxicology and exposure sciences
General toxicology, computer science, exposure assessment, statistics, and NAMs
Regulatory toxicologist with competence in bioinformatics, new generation risk assessment methodologies
The department will need both personal with general toxicology with PhD degree, and more highly specialized personnel, especially within new methods (in vivo/in silico), bioinformatics, exposure assessments and environmental epidemiology. Areas as endocrine, immune and neurologic effects will also be areas of interest for the future.
Both, general toxicology/related science education preferably young recruits which are interested to become specialised in our field and also persons with highly specified education which can step in, continue and further develop our already started work.
No needs.

Response/Comments SE.

Recruitment of 5-10 general toxicologists over the next 7 years.
We need to recruit PhD competence with relevant scientific education in toxicology and chemistry.
Our needs will be general toxicology to be able to assess chemicals for safe substitution and risk assessment for air and dermal contact.
We foresee recruiting both tox and ecotox competence in general. Regarding highly specified education we see that both competence within environmental fate and ED is needed. When recruiting we see that few has regulatory knowledge and this is a competence we foresee is needed in combination with above.
The organisation has and will have sufficient staffing in these roles. Yet, if people decide to leave they are very difficult to replace because of lack of experience and training required for the chemical industry.
There will be needs.
We have a lack of experts with competence in food toxicology especially in area of natural toxins, radioactive contamination and food allergy.
General competence of more importance than specified.
We are currently recruiting a person specialized in monitoring of human exposure to hazardous substances in the everyday environment.
In general, students today seem to choose broad, non-specialized educations. This applies to the highest degree to education in the environmental field and means that there are not many with a good knowledge of chemistry and toxicology.
General tox, in combination with an understanding of legislation.
In general, we mainly need to recruit toxicologists, ecotoxicologists, chemists. Preferably with a PhD degree and with various specializations/competences. We are in the midst of a major change with both a new regulation (Market Control Regulation) and Sweden's presidency of the EU, which will affect the need for recruitment and the need for skill development of existing personnel for many years.

Question 9: Please indicate your experience related to how easy it is to recruit competencies in chemical risk assessment/ communication you want to have /hire. Please use a number between 1 (easy to recruit) and 6 (difficult to recruit).

The results shown in the diagram below are representing the merged results from DK, FI, NO and SE.

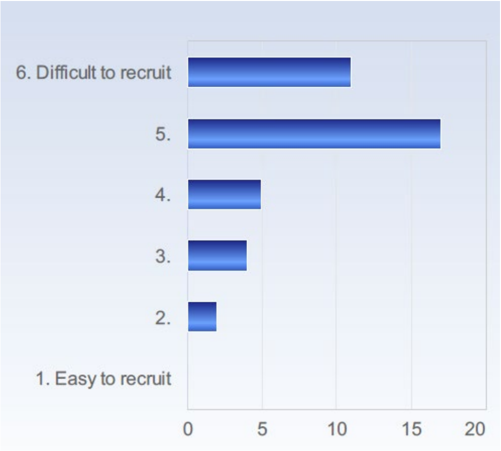

Question 10. Indicate if academia delivers sufficient number of candidates to fulfil your needs (master and/or PhD level)?

Response/Comments DK.

PhD level: Not (yet) with the expertise mentioned earlier (probabilistic risk assessment, AI, NAMs, exposure modelling). Not sufficient with regard to toxicologists. Difficult for public administration to compete with private companies.
Yes, as we can provide further competence development.
However, a general concern exists as there is strong competition in getting the best candidates.
For exposure assessment, there are not sufficient candidates. It is also difficult in other fields, maybe not so much in toxicology

Response/Comments FI.

Number of candidates may be sufficient, but the quality varies too much.
At least within the PhD level, there is not sufficient number of trainees on analytic epidemiology on chemical hazards.
There is no relevant education for new biomaterials risk evaluation thus the job requires additional training.
Risk assessment science is not taught in any Finnish university, neither are toxicologists easily available. Therefore, the candidates are hired according to their other competences.
Academia delivers sufficient number of toxicologists with master degrees every year, but usually they don't have the required experience for risk assessment.
Yes and no. It is not only getting newly graduated experts, but also have work experience. But in general: more academic MSc/PhDs are needed to provide sufficient pool of candidates.
At the moment not. MSc-level would suffice in order for these people to be available on the work markets faster.
In Helsinki area there enough training in chemistry, biology, biochemistry or pharmacy.
As regards chemistry/pharmacy educations, yes.
We have identified problems in recruiting medical doctors with interest in clinical toxicology. While there are several subspecialty training programs available for medical doctors, a subspecialty training in clinical toxicology does not exist. In Finland, there is not a professorship in clinical toxicology.

There might be a need for more experts especially in the field of industrial hygiene but possibly also in toxicology. In the case of toxicology, in toxicology education even more emphasis could be given for risk assessment skills and the students should be made more familiar also with the special field of occupational toxicology.
No, especially regulatory toxicology/ecotoxicology.
To our knowledge, toxicology can only be studied after a BSc degree in chemistry / pharmacy / medicine / biochemistry or equivalent; a BSc degree in toxicology is not available. The MSc in Toxicology is only offered as an international study program, in which there may not be enough experts who also speak Finnish fluently, which is necessary in order to work for the Finnish national authority. Recruitment challenges describe skills needed and job assignments in a way that reaches all potential applicants. Tukes may not be a sufficiently well-known employer and ECHA competes for staff.

Response/Comments NO.

We are not located in a central area around Oslo or Trondhjem, meaning that there are very few people with relevant background (master or higher) within human- and eco-toxicology. Local process industry would have hired more toxicologists if it had been possible to find them.
Very limited in feed science, veterinary toxicology, etc. limited in pathology,
On a master level there are a number of candidates but very much ecotoxicology focussed and not a lot with a human toxicology focus. PhD are much more difficult to recruit since there are not enough funded PhD positions available. Universities focus more on ecotoxicology than human toxicology
No
Not for human toxicologists
There is a growing need for personal within risk assessment with PhD degree. We are very concerned for future possibilities for employing toxicologists with required expertise/skills.
There is not a sufficient number of candidates available, both at Master and PhD level.
Yes

Response/Comments SE.

Do not know, but there is an obvious lack of Toxicologists in Sweden with any work experience within the area of Risk Assessment. Perhaps - the area is too small.
I believe they do, however we are new in the field and it is hard to attract people.
Yes, the number of candidates fulfils our need however we would like them to have a more regulatory knowledge within their education.
For chemical industry you need people with interdisciplinary knowledge which is underestimated in academia. Not just in order to better grasp the problems you are faced with but also in order to communicate with other specialists (process engineers, chemists, legal, communication team, business managers etc). The toxicology training used to be better in this but I feel that during the last 20 years the training has become more proned to develop very specialised students only suitable for further academic research.
Lack of experience is the problem
Academia does not deliver candidates fully trained i food toxicology in Sweden.
Not enough to fulfil our needs. We have very few good job candidates. We often need PhD level, with broad knowledge.
My experience is that fresh PhDs are not aware of the possibilities within local authorities. So it's not so much about sufficient number altogether, more that there is a lack of interested candidates.
See answer above - So, no they don't.
We need experienced competences, PhDs are great for instilling confidence and demonstrating the importance of knowledge
Yes, academia do deliver what we need, so far.

Q11: Do you find it necessary to train your new personnel in the areas of chemical risk assessment/communication due to limited/poor knowledge from academia or previous affiliation(s)/work experience? If YES, please comment on how you train the personnel.

The merged response from the four Nordic countries (DK, FI, NO and SE). Comments shown county wise below the diagram.

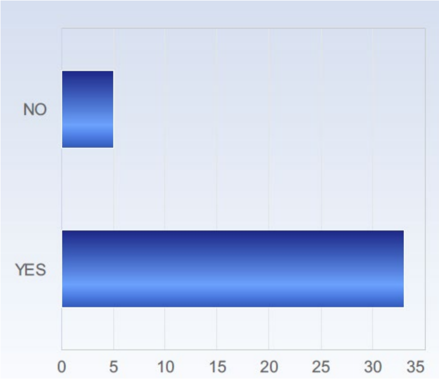

Response/Comments DK.

By job training, NIVA courses, Courses, webinars
Regulatory knowledge is often insufficient. Very different levels of scientific competences from newly recruited staff. Depends amongst others on the level of specialisation during the studies.
Task specific training needed.
Internal and external courses, "sidemandsoplæring"

Response/Comments FI.

Internal training and external courses.
Not possible for the moment, because of lack of research resources and prioritization
Hands on work i.e. research proposal preparation, occupational health regulations, monthly QMS announcements.
Mostly on the job learning.
Conferences, BTSF and other courses, and previous materials are utilized.
We don't have specific training resources, our way is to participate in relevant courses and learn from older, more experienced colleagues.
By sending them to external training programmes and courses - even basic level training sometimes needed.
Through internal and external training courses, and external webinars. Internal and external seminars/trainings or presentations are useful. Regulatory guidelines, internal SOPs, supervisor training, training courses.
A long self-tailored introduction/familiarization period of ~6 months
In the fields of industrial hygiene and occupational toxicology (and regulatory risk assessment related to e.g. limit value setting) it is not possible to find personnel who would have those skills when entering the institute. Therefore, we are currently considering if it would be possible to set up "trainee" positions in our institute to fulfil our own future needs for skilled personnel but also to increase the expertise in these fields in Finland in general. This is however only very early phases of the planning.
Experienced colleagues are training new personnel. BTSF trainings & risk assessment trainings organized by Wageningen University etc.
Work shop organized by Karolinska institutet, ECHA and EU member states.

Persons in enforcement have to carry our risk assessment and risk management tasks, but all of them do not have toxicology degree.

Response/Comments NO.

Almost a requirement to succeed.
On the job training being active in the consultancy work and in research projects.
Relevant workshops, courses.
We put them under traineeship of senior personnel.
Yes, new employs will be included in risk assessment teams and trained by experienced personal in the department. In addition, it is important that all personal participate in relevant courses and conferences.
Internal seminars, literature studies, participation on conferences/workshops.

Response/Comments SE.

Formal internal education. Informal education during work.
How to interpret regulation and guidance. We always need to educate for IUCLID and Chesar.
Yes if a newly examined person is hired, that would be necessary. But previous experience is preferred.
Academia does not deliver candidates fully trained i food toxicology in Sweden. Since the food toxicolgy area is a specialized area with demands on knowledge of food habits, consumption etc, this is something that needs to be covered "on the job" by courses by EFSA, or by learning from colleagues.
We try to train staff in basic knowledge of different groups of chemicals and their applications and the most common hazards associated with these chemicals. We encourage everyone to attend seminars, webinars, etc. to gain as much knowledge as possible. When possible and we find a suitable course, employees can take these, but the supply is poor.
We need people with competence and experience. We find it possible to teach in legal and other requirements (communication), but impossible for us to teach in deep knowledge in risk assessment.

Q12: Which areas of expertise areas are primarily lacking when you want to hire new personnel?

The merged results for DK, FI, NO and SE are presented in the graph below. It looks a bit different between the countries as shown for each of them as comments are show country wise below.

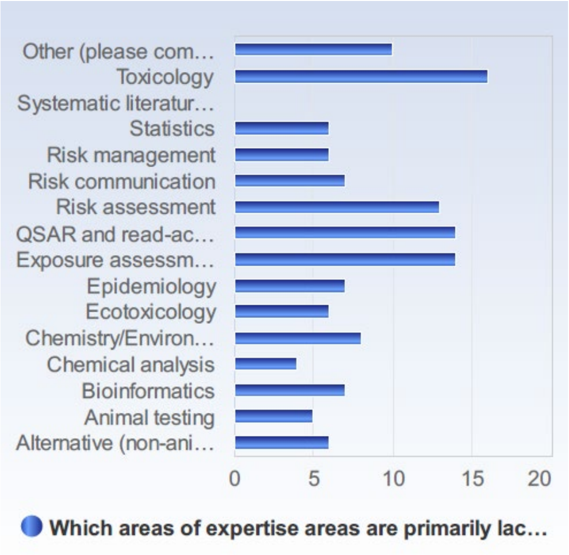

Results and Comments DK.

Which areas of expertise areas are primarily lacking when you want to hire new personnel?	Number of responses
Alternative (non-animal) in vitro methods	0 (0,0%)
Animal testing	0 (0,0%)
Bioinformatics	1 (16,7%)
Chemical analysis	1 (16,7%)
Chemistry/Environmental chemistry	1 (16,7%)
Ecotoxicology	1 (16,7%)
Epidemiology	1 (16,7%)
Exposure assessment	2 (33,3%)
QSAR and read-across	3 (50,0%)
Risk assessment	0 (0,0%)
Risk communication	0 (0,0%)
Risk management	0 (0,0%)
Statistics	0 (0,0%)
Systematic literature reviews	0 (0,0%)
Toxicology	0 (0,0%)
Other (please comment below)	2 (33,3%)
Total	12 (200,0%)

Comments: Probabilistic risk assessment, AI, NAMS and exposure modelling
Not possible to answer since it depends on the specific position.
Management is not always specifically looking for toxicology skills. General scientist/biological skills are not lacking currently.

Results and Comments FI.

Which areas of expertise areas are primarily lacking when you want to hire new personnel?	Number of responses
Alternative (non-animal) in vitro methods	2 (16,7%)
Animal testing	1 (8,3%)
Bioinformatics	2 (16,7%)
Chemical analysis	0 (0,0%)
Chemistry/Environmental chemistry	2 (16,7%)
Ecotoxicology	3 (25,0%)
Epidemiology	4 (33,3%)
Exposure assessment	7 (58,3%)
QSAR and read-across	5 (41,7%)
Risk assessment	8 (66,7%)
Risk communication	4 (33,3%)
Risk management	3 (25,0%)
Statistics	3 (25,0%)
Systematic literature reviews	0 (0,0%)
Toxicology	7 (58,3%)
Other (please comment below)	2 (16,7%)
Total	53 (441,7%)

Comments: Regulatory toxicology and ecotoxicology
Recirculation, industrial side streams, new biomaterials, new processes for waste treatment health hazards
As I mentioned in my previous answers, industrial hygiene and occupational toxicology/toxicological risk assessment expertise are the main areas we see the challenges. However, we have had some problems in finding also experts experienced in specific in vitro methods.
Regulatory expertise.

Results and Comments NO.

Which areas of expertise areas are primarily lacking when you want to hire new personnel?	Number of responses
Alternative (non-animal) in vitro methods	4 (57,1%)
Animal testing	3 (42,9%)
Bioinformatics	4 (57,1%)
Chemical analysis	3 (42,9%)
Chemistry/Environmental chemistry	2 (28,6%)
Ecotoxicology	1 (14,3%)
Epidemiology	1 (14,3%)
Exposure assessment	4 (57,1%)
QSAR and read-across	5 (71,4%)
Risk assessment	2 (28,6%)
Risk communication	0 (0,0%)
Risk management	0 (0,0%)
Statistics	3 (42,9%)
Systematic literature reviews	0 (0,0%)
Toxicology	6 (85,7%)
Other (please comment below)	0 (0,0%)
Total	38 (542,9%)

Comments: We are going to need personnel with toxicology background, that also have specialised in the areas noted above.

Results and Comments SE.

Which areas of expertise areas are primarily lacking when you want to hire new personnel?	Number of responses
Alternative (non-animal) in vitro methods	0 (0,0%)
Animal testing	1 (9,1%)
Bioinformatics	0 (0,0%)
Chemical analysis	0 (0,0%)
Chemistry/Environmental chemistry	3 (27,3%)
Ecotoxicology	1 (9,1%)
Epidemiology	1 (9,1%)
Exposure assessment	1 (9,1%)
QSAR and read-across	1 (9,1%)
Risk assessment	3 (27,3%)
Risk communication	3 (27,3%)
Risk management	3 (27,3%)
Statistics	0 (0,0%)
Systematic literature reviews	0 (0,0%)
Toxicology	3 (27,3%)
Other (please comment below)	6 (54,5%)
Total	26 (236,4%)

Comment: Connection to industry related topics further from the "normal" areas. Regulatory; IUCLID; Chesar

How to read guidance. If a hire know one of the topic above, all the others will be lacking. As a regulatory industry toxicologist you need to understand (not only grasp) all boxes above and more!

The combination of the above checked expertise is almost impossible to find in young people.

The mindset regarding finding and learning new information is the most important.

Food allergy; Radionuclear contamination; Immunology

Experience about how to give advice to end-users of chemicals/articles for avoiding/reducing chemical risks.

A combination of more or less everything above, several individuals can complete each other.

We generally find these competencies when we look for them.

Q13: Please suggest university courses related to chemical risk assessment/communication you would like to see offered by academia in a near future.

Response/Comments DK.

Bench mark dose modelling for quantitative risk assessment-
Probabilistic risk assessment based on AI, NAMs (including e.g., RASAR) and exposure modelling.
Regulatory toxicology.
Use of digital tools, computational toxicology (as we have difficulties in internal education).
Risk assessment of materials/impurities (requires multiple skills and experience in materials understanding and chemistry as well as biology).
Course in applied toxicology. There used to be a post graudate course at University of Copenhagen, but it does not seem to be available any more.

Response/Comments FI.

Regulatory toxicology
Waste management chemical and microbiological risks, thermochemical processing volatile gaseous components.
Chemical risk assessment on food safety (Codex Alimentarius Commission).
Risk communication on food safety.
In Finland there is only one university (far from capital area) that offers master's degree in toxicology. At least University of Helsinki should give such a possibility, too.
e.g. Evaluation of in vivo and in vitro studies for risk assessment, Mixture toxicity and cumulative risk assessment, Computational toxicology.
Assessment on food contact materials. Hazard evaluation. Modern toxicology techniques (omics) applied to risk assessments.
Any courses on risk assessment and risk communication would be welcomed. Also courses on toxicology (general) for non-toxicologists i.e. courses which would not aim at ERT but would help e.g. food chemists, nutritionists etc in their work within the area of risk assessment.
Consumer issues are challenging, because some chemical are in several authorities area - needs communication platform. Nanotehchnology is exellent example for this issue with Titanium dioxide and additve (E171).
In Denmark there is a kind of food safety issue discussion group, that also authorities follow and occasionally comment as well.
Fraunhofer ITEM provides good trainings in toxicology at least in food and feed area (provided by EFSA for EFSA connected people).
Regulatory toxicology-Risk assessment-medicinal products.
Ecotoxicology.
Medical / clinical acute toxicology
We have had discussions with UEF on the collaboration between our institute and UEF on the organisation of the occupational toxicology course. Whether this collaboration will happen is, however, still open. What comes to industrial hygiene, although there are courses related to
it at UEF, it seems that number of students specializing for it is currently not sufficient for our needs.
Global regulatory (eco)toxicology.
In silico methods in practice for Plant Protection Products, alternative in vitro methods in regulatory toxicology,
Toxicokinetics. Genotoxicology, Dermal absorption, Formulation chemistry

Response/Comments NO.

Basic toxicology both within human and eco. Regulatory toxicology.
More focus on regulatory framework and toxicologists with bioinformatics and biostatistics skills.
More hands-on experience with advanced.
laboratory models including in vitro, transcriptomics and proteomic data.
MSc in risk assessment.

Human toxicology, OMICS; New methods in risk assessment (in vitro/in silico) and exposure assessment.
Case related courses in order to train the students for future real life scenarios.

Response/Comments SE.

More courses focusing on guidelines and test methods for chemical risk assessment, both in silico, in vitro and in vivo testing.
Risk communication in connection to safe substitution and in the communication with suppliers to increase knowledge in the area.
Courses on IUCLID, Chesar.
Regulatory globally.
Practical case studies in risk assessment and communication.
There should be more courses on chemical legislation and application of the legislation.
CLP, REACH, Chemical risk assessment.
Food toxicology. Risk-Benefit assessments. Immunology. Epidemiology. Radionuclear contamination.
Risk assessment and risk management of the catchment areas for abstraction points of water intended for human consumption - to fulfil the new drinking water directive.
Chemical risk reduction in non-chemical contexts (i.e. outside the traditional laboratory/industrial context. Focussing on the use of materials, articles, products and communicating with suppliers and customers. How to get information from suppliers etc).
Courses that meet the need for staff with knowledge of chemicals, toxicology and legislation at a level that allows them to work in different industries. It is not always necessary to have knowledge at a master's level in, for example, toxicology. However, the broad educations that have become popular sometimes include only one semester in total of toxicology and chemistry. It is not enough to understand chemicals and how they work in different materials, different fractions, toxicological aspects and legislation.
Understanding legal requirements and legal processes. Reach, CLP, ...
Test methods (non-animal), Basic compare animal testing vs non-animal testing. How to integrate research info, useful regulatory actions and understanding how science and policy interact.
Developments in bioinformatics.

Q14: Please indicate, if you expect a lack of expertise in the area of risk assessment/ communication in your organisation, in the near future.

Diagram of the merged result for DK, FI, No and SE below.

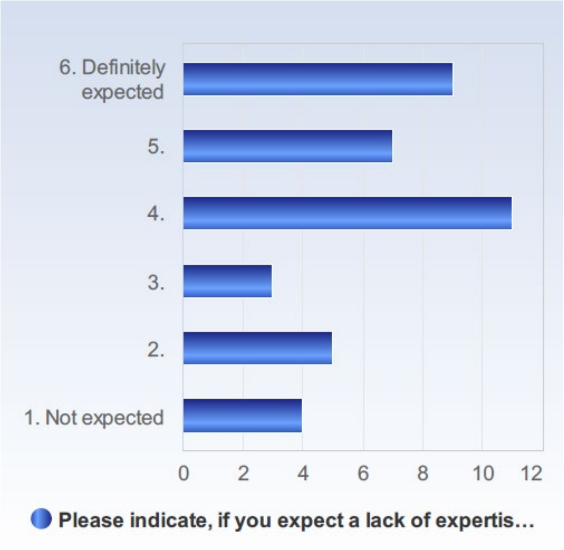

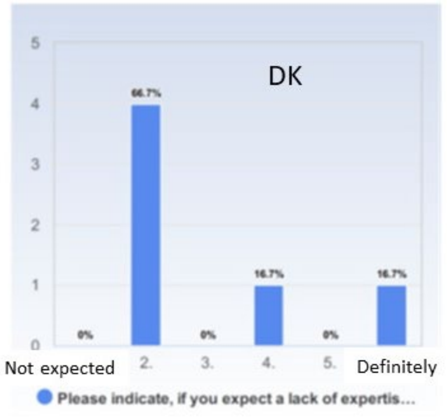

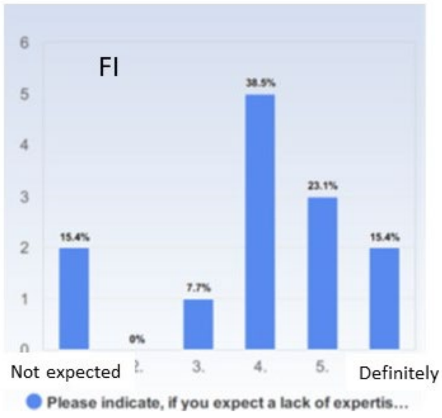

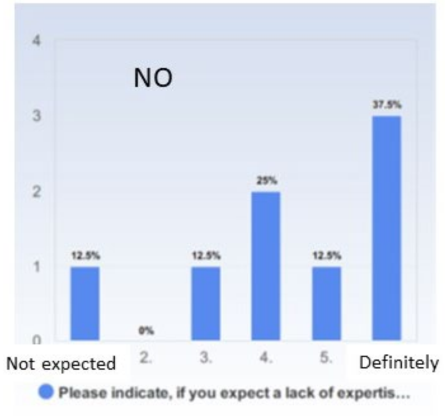

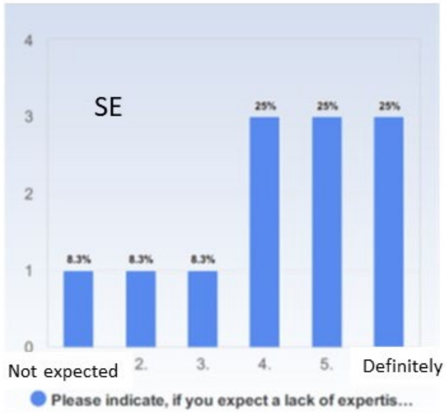

Question 15: Please explain how you foresee to meet the needs of highly competent/qualified personnel in chemical risk assessment/communication in your organization five years from now (e.g. external courses, workshops or internal courses).

Response/Comments DK.

We educate PhD students and post docs, but we also recruit from other institutions, i.e. universities.
Internal and external courses.
Internal training as mentioned earlier (courses, webinars, ...)
Internal training is most important. Availability of external courses is not meeting the demand currently.
Same expectations as now. Expect to do internal training mainly.
Participate in courses, e.g. online and physical courses.

Response/Comments FI.

Good working circumstances, larger team supporting the individual expert in his/her job. First of all, prioritization and funding should be made available for the key factors still requiering research (e.g. identifying carcinogenic risks) in the national research funding. If this will not be in the priority on the national funding it will not be in the budgets and resource allocation of the collaborative institutes and it will be very difficult to organize and perform appropriate research and training.
1. Hands on experience 2. Legislation development by the policy makers 3. QMS reporting at work when new processes are developed.
Internal training, BTSF and other external courses, conferences,
Participation in relevant research and external courses.
External courses and external training programmes and workshops.

Through internal and external training. Through keeping abreast of the new developments at EFSA, ECHA, EMA, EC, FDA, EPA, JECFA, IPCS, FAO/WHO, ILSI etc. Through training courses, workshops, guidance documents etc organised by the aforementioned bodies.
There is need for understanding and communicating of safety issues and explanations of withdrawals or bans to consumers. Currently EU and for example Horizon Europe programs asks in partnerships a collaboration and chemical safety issues are also included.
External courses, workshops.
Finnish Poison Information Center is a 60-year old center located within the largest hospital district of Finland (HUS). We rely on strong collaboration with the clinicians and good contacts with other Finnish authorities. We have our own training program for new employees as well as a biweekly own scientific meeting program. We also collaborate with Nordic Poison Centres and participate in external conferences and courses.
I already mentioned that trainee -program we have initially planned and discussions with UEF related to occupational toxicology course. We have also discussed with UEF on the possibility of our personnel to participate for some toxicology courses in UEF (as part of continued education). We are also following closely external courses available in Europe and for example some of our experts have participated in toxicology/risk assessment courses organised by Karoliska Institutet.
Internal/external training and utilizing external consultants.
Internal or external courses on specific topics, e.g. QSAR, in vitro methods; risk assessment in general. With continuous learning and updating education using external courses. Co-operation with European colleagues. ECHA workshops.

Response/Comments NO.

Recruitment, external courses, internal training in suspected animal poisonings,
Combination of external courses and internal workshops. Active participation in a number of EU projects might offer training opportunities.
Participating in workshops, courses, internal training. Maybe participation in the EU-FORA fellowship.
External course, internal courses and internal courses as well as practical training and internship.
Yes, new employs will be trained by experienced personal and participate in relevant courses and conferences.
External courses and workshops and also internal seminars/discussions.
External courses, certification.

Response/Comments SE.

External and internal courses, workshops etc.
Mostly with research projects and courses for personnel.
Internal education, collaboration with universities/consultants, workshop by authorities and international conferences.
If no staff leave us we have the right level om competence. If we need to hire and replace a lot of internal training is required. Hoping to find good people with the right mindset. Then internal education.
Via external courses, workshops or internal courses
If we cannot find a highly qualified person to hire, I guess we have to educate a less qualified person.
Most internal education (person to person), but also external 1-2 day courses.
We hope to further develop our cooperation with academia in terms of int. projects, master students, discussions about research findings and our questions etc.
We try to hire new personnel through search recruitments that unfortunately are time consuming and expensive.
Hire experienced and supplement with internal supervision.

Q16: Please give suggestions what can be done nationally to optimize the numbers of competent persons for your organization.

Response/Comments DK.

It would be great to have a proper toxicology education in DK e.g. a master of toxicology at one of the universities, and to have a PhD school that focusses on different aspects of toxicology.
Include more risk analysis courses in relevant educations (such as chemistry, biology etc.).
Organize courses and training that fit into New Generation Risk Assessment (i.e., without the use of animals).
Establishment of a toxicology education.
Course in Applied toxicology in Denmark

Response/Comments FI.

Good teachers, marketing, innovative learning methods, learning by doing
National policy makers should update legislation taking into consideration biodiversity and chemical burden for each new chemical compound (i.e. Do Not Significant Harm policy from EU).
Increase of toxicology education, especially in geographical areas where it is needed. ALSO risk assessment education! Resources to recruit needs to be increased.
There are plans to establish a toxicological centre of expertise in Finland that would develop toxicological expertise, in particular regarding risk assessment. This plan now waits decisions and resources from the government in order to be realized.
By providing enough basic training options for potential students (in toxicology, ecotoxicology) is essential starting point. Also providing options for Master of Science graduated (in chemistry, ecology, medicine, biology...) to further specialize to toxicology as a second graduations (up to PhD level).
Perhaps on-line training courses for self-learning could be developed.
Enough delivery of academia level educated candidates.
Need to attract more medical doctors to the field.
See also my previous replies. Increase collaboration between universities and research institutes and e.g. industry who also needs this expertise. Ensure funding; e.g. funding related to organising such kind of trainee positions in this field would be useful.
More co-operation with industry during studies.
To establish national toxicology centre or otherwise create a functioning and flexible network of experts.

Response/Comments NO.

Germany, France, UK all have consultancy companies with highly qualified toxicologists - often with different people specialising in different areas like literature review, follow up on animal testing, exposure assessments, QSAR, ecotox., epidemiology etc. We don't have any of this in Norway. It is some scattered knowledge within a few organisations like DNV and so on, but they aren't big enough to provide broad enough support. We are always dependent on international support. It is sad that we can't get that type of support in Norway. And for advanced chemical process industry outside Oslo, it is very difficult to find people with sufficient academic background within this field.
Toxicology training in a One Health perspective, collected compendium related to animal poisoning, causes and symptoms for poisonings.
Specific training program to recruit young toxicologists into the area of human toxicology. The research council should award some research projects within human toxicology so that we can hire PhD students and postdocs.
Increase the focus on human toxicology and methods for risk assessment. This should also be combined with methods for systematic review and evidence-based toxicology thinking.
Education of human toxicologists at the university level, provision of university courses in human toxicology.

We need more national funded research within human health and toxicology to ensure PhD candidates for future toxicology science and advisory work. More programs with focus on human health and toxicology issues in The Research Council of Norway is highly essential.
Recruitment and academic education of young people to guarantee a high enough number of competent persons in the future. Starting recruitment already at school level by demonstrating how interesting, useful, exiting and necessary this specific work is. Publishing more topic-related articles in social media as e.g. newspapers, to educate the general population and to catch attention and interest of especially younger people who are at a stage where deciding about their professional future pathway.
No suggestions, have no needs.

Response/Comments SE.

Adjust the current national courses in Toxicology to meet the current needs of Swedish organisations.
I think the Substitution centre is doing a great job, however the need is for all industries.
See earlier replies. We recruit globally and do not see a specific national need.
Networking
We find it efficient to train toxicologists "on the job" after recruitment. The most important prerequisite is that the basic training of toxicologists is kept at a high international level.
Long term: To educate more people. Short term: To form a network with people working in the area that can share competence and job opportunities.
Se answer on question 14
Networking, cooperation between consultants and other organisations. Support networking with like-minded abroad.
A good balance between academia/research, regulators/administration and industry.

Q17: Please suggest how the Nordic countries could act jointly to optimize the numbers of competent persons for your organization.

Response/Comments DK.

The master of toxicology and the PhD school could be a Nordic activity or be coordinated between key institutes in Nordic countries
Co-operate to establish a joint education (cf. ERT).
Start with talking and identifying gaps.
Relevant courses with selected topics.
Joint courses including industry focus.
Exchange between countries.
Common courses.

Response/Comments FI.

Co-operation like workshops, webinars/seminars, subgroups of special items,
Regulation is same in EU-level. Is Nordic countries right forum?
Please see the previous reply.
Food safety risk assessment courses in English given by the Nordic countries together.
Something like Nordiska Hälsovårdshögskolan should be founded again?
Nordic countries could organize joint training courses.
Maybe a joint Nordic programme in one of Nordic countries, or even splitting the expertise area between different Nordic counties (if resources cannot be put on one country only).
Perhaps creating a strong network of the expertise in the Nordic countries. A roster of experts and expertise could be established similar to JECFA or EFSA.
Common trainings and projects organized by HSSD or NIVA for example or other Nordic organisations.
Join training of academy students.

Student exchange between universities.
Nordic workshops.

Increase collaboration to provide training and education options.
We should consider organising post-graduate training course jointly.
Co-operation with different stakeholders, e.g. industry, organizations, authorities, universities.
Continue co-operation in trainings / courses on specific topics, ensure funding eg. via NKE (the Nordic Working Group for Chemicals, Enviroment an Health).
Nordic level co-operation in university level education DTU, KI, Göteborg?? Better co-operation network between Nordic universities offering ecotox and tox education. A list of Nordic persons with their expertise could be useful.

Response/Comments NO.

Nordic training and network, related to veterinary toxicology as the total need is limited,
Organise training courses (3-4 days) that focus on specific areas.
On-line courses that people can take in their own time.
A Nordic PhD program with research projects
Nordic training in relevant topics
Setting up common courses with common syllabus
Arranging joint training programs for the countries will be a valuable initiative. See also 16)
Education of compentent persons on a Nordic level by arranging Nordic courses/workshops for people who already have a relevant basic education - further development - and academic courses/schools for people being in an earlier career stage (as students or PhD-candidates).
No suggestions, have no needs

Response/Comments SE.

A more active joint toxicology organization.
The Nordic Expert Group for Criteria Documentation of Health Risks from Chemicals is an example of well-functioning Nordic joint work.
Other types of background documentation as well as impact assessments for specific chemical agents are fields that may be suitable for
Nordic collaboration.
Make sure there is a possibility to help all industries.
Nordic trainings and networks are always useful to increase the critical mass.
Collaboration and networking
The option to cooperate with other Nordic authorities, for example through the EFSA EU-fora mechanism with expert exchange.
Have no suggestion.
Can universities merge, use their common resource pool and develop different types of education? Some parts may be common to all students. Others could be aimed at those who want to continue in academia and others at those who want to enter, for example, industry.
Networking and common trainings and educations. Invite to seminars and presentations.

Q18: Do you see positive possibilities for a closer, formalized cooperation between the Nordic countries in the area of chemical risk analysis, including training?
If YES - How could this cooperation be accomplished? / If NO - Why would that be hard to accomplish?

The merged result for DK, FI, NO, SE shown in the diagram below. Comments country wise thereafter.

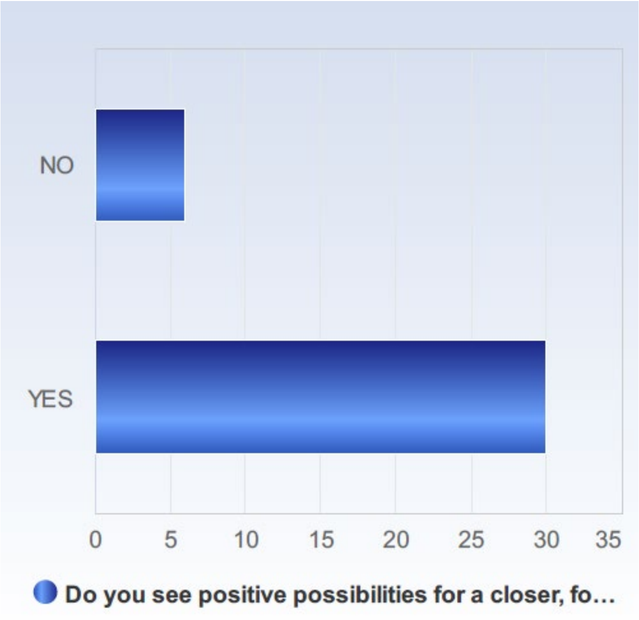

Response/Comments DK.

Yes, using NIVA courses and other Nordic inititatives. It would also be good if the Nordic countries could collaborate closer in risk assessment and regulation e.g. in occupational health.
Co-operate to establish a joint education (cf. ERT).
Agree to implement New generation Risk Assessment, create an understanding of what it implies and identify gaps in the current training and education.
Formalised cooperation already exists between authorities (Nordic Chemical Group).
Via the Nordic council of ministers; NIVA

Response/Comments FI.

EU-level is better.
In principle, many of the research requirements are quite the same in the Nordic countries. Still, it is not easy to organize such projects that would satisfy some standard definitions and harmonisation, because of lack of organization and resources for research collaboration, and heterogeneity between the research groups in their goals and projects.
Maybe in the field pharma, but in biotech (recycling, utilization of side streams) very difficult because of conflicting interests.
Co-organized training to found the basis for cooperation (e.g. mutual language on concepts is needed).
We already have some research cooperation, although its strengthening would benefit the quality of risk assessment.
NO, we suggest that European level would be better
I see this as a positive option but I cannot recognize the roadblocks. I guess the key is: how to finance this? Are there enough capacity and number of teachers / professors?
See above/look pervious page
See Q17.

We already collaborate with Nordic Poison Centres in terms of antidote use, for example.
For example, organising post-graduate training program in this field.
PPP Northern Zone cooperation is already strong but needs to be continued.
More unformalized Nordic cooperation would be needed. That cooperation could be regular meetings in risk assessment issues or Ad Hoc meetings when needed.

Response/Comments NO.

Nordic council should put some money on the table to develop training courses of sponsor PhD projects in the area of human toxicology.
For subjects that are of interest for risk managers in more Nordic countries, it is better to collaborate than to perform separate assessment.
Collaboration of toxicology societies, closer contact between regulatory authorities.
Nordic workshops, courses, meetings organised by national/Nordic authorities and together with e.g. national toxicological/chemical societies.
Courses/workshops with different hosts in the Nordic countries.

Response/Comments SE.

Workshops where knowledge can be shared.
Networking, collaboration between national authorities
see for example question 17
Do not know
From my own experience I know that one Nordic country are not big enough to be specialized in all disciplines
See no 17

Question 19: How many of your experts within chemical risk assessment/communication are involved in international assignments related to the organizations indicated below?

Numbers of engaged personnel from our four countries (DK, FI, NO and SE) are shown in the diagram below. The persons engaged may not do this full time.

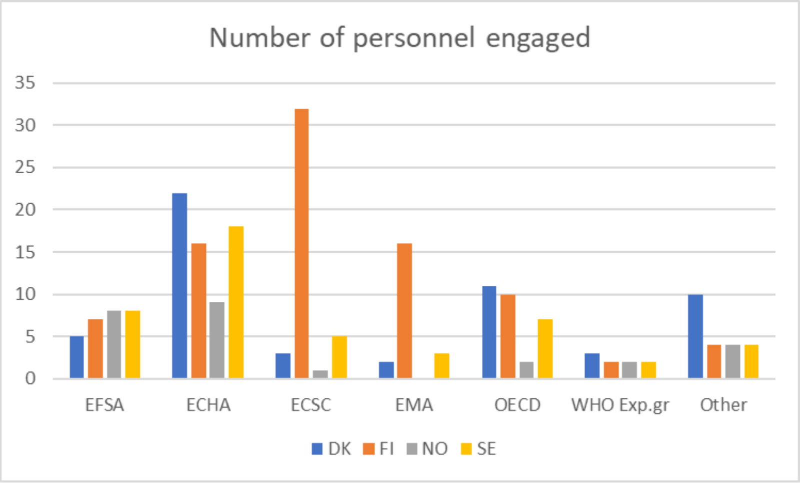

ECSC stands for EC Scientific Committees; EMA for European Medicines Agency committees; “Other groups” are specified below.

Comments DK.

Nordic Expert Group

Participation is supported/driven by the participation in the Horizon2020 ONTOX project and includes also the ASPIS cluster and (from May 2022) also PARC.
Others: Commission working groups.
Toxicologists not involved in above committees. Some give input to ICH guidelines etc. Several collaborate with other industries on giving input to regulatory improvements.
ISO working groups for standardization of methods.

Comments FI.

No personnel available for such assignments.
It would be desirable to participate, but our minimal resources are not enough to do so. At the moment only national risk assessment assignments could be carried out.
No one in that high-level forums.
One ex-EFSA staff member who coordinated EFSA CONTAM WGs and the PANEL and developed CONTAM risk assessments for 10 years works in our company. She has also some experience in risk communication and/or management from EC, EFSA and ECHA, as well as from national FSAs.
Nordic Expert Group for Criteria Documentation of Health Risks from Chemicals (NEG). Related to ECHA expert groups, one of our experts is a member of RAC but there are two others who have been assigned as member's advisors.
Industry experts from individual companies usually do not participating these panels.

Comments NO.

Internationally. I have represented industry in relevant industry organisations like Eurometaux and CEFIC.
Previously actively participated in relevant EFSA Working groups, EC scientific committees,
Participates in reference laboratory studies, and method developments and ILSI Working group on food allergens
IARC
NEG
None of the above

Comments SE.

None
"Other": 3 experts are members of EFSA panel working groups other than those part of EFSA panels.
SAICM - related working groups
UNEP - related working groups on sustainable chemicals

Question 20: Do you expect that the number of personnel involved in international assignments (c.f. previous question) will increase or decrease in the coming 5 years?

The summarised response shown in the diagram below. Comments given below as well (country wise).

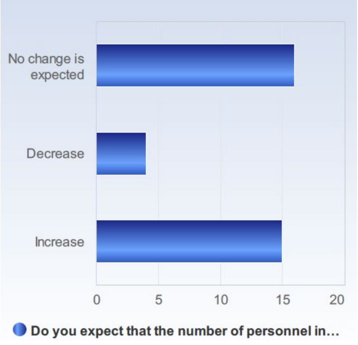

Comments DK.

These activities are difficult to cover by external funding, and we have to cover our research 120% by external funding.
Small increase.
One will retire, and the cost of participating in ISO working groups are high. Our institute is cutting down costs and focusing on our core tasks, so less and less time is available for participation in such tasks.

Comments FI.

May be some (?)
After the retirement of the part-time expert, there will be no substitutes available at our institute (other than possible participation by the retired experts).
Increasing research funding is coming from EU and national funding sources are aligning their requirements for funding with the EU.
We really hope that the answer 'Increase' would happen, but if thinking realistically, probably no change is expected.
Actually, no view to this question.
We are a CRO/consultant company and have therefore conflict of interest as regards to participation to the work of the above public bodies.
Representation in international organisations already at high level.

Response/Comments NO.

Increase because there will a high demand of these specialised experts. But not sure if we can prioritise participation in these international groups above national tasks.
Together with climate changes, biodiversity and pollution/chemical exposure is a global challenge, and international cooperation is higly nessesary.

Response/Comments SE.

We need to make sure risk assessment will be part of the decisions in our field.
Likely as we have fairly young experts currently.

Q21: Additional comments

Response/Comments DK.

There are a lot of EU initiatives aiming at chemical risk assessment without the use of animals. Analyse the needs and expectations of these initiatives and build relevant courses, workshops, webinars to train new risk assessors, managers, communicators for these new tasks and challenges.
Use of toxicological discipline could be better integrated part of several lines of education. Could benefit from industrial perspective also.

Response/Comments FI.

Please, take the number of people given in this questionnaire only indicative since it very difficult to assess the actual number of people involved in risk assessment / communication.
These answers reflect the situation in Finnish Food Authority's Risk Assessment Unit. There will be additional information in a separate form(s) from other units, where our answers are excluded.
No additional views.
Currently we do not have much solutions but we are open to discuss and we see a need for further co-operation.

Response/Comments NO.

It is timely that this is put on the Nordic agenda!

Response/Comments SE.

From our perspective (consultants) the need for help from companies and authorities is increasing together with more complex issues. We are continuously educating our customers regarding these matters, which is one reason for the meaningfulness of consultancy.
Since the EU-legislations nowadays often include risk assessment and risk management, the need for highly qualified people with this competence will be required.
I see two different kind of needs: To strengthen universities by exchanging research / education at all levels. This to form specialists at a high level. To create training that provides competent employees for other activities such as industry, municipalities, etc
The area is becoming more and more complex. The legal requirements very often require an expert to interpret and follow; this is very clear from a consultant's horizon.

Åke Bergman prepared the Word summary (ver. 22-11-21).
